# A phase II randomized trial of sodium oligomannate in Alzheimer’s dementia

**DOI:** 10.1186/s13195-020-00678-3

**Published:** 2020-09-14

**Authors:** Tao Wang, Weihong Kuang, Wei Chen, Wenwei Xu, Liming Zhang, Yingjie Li, Hailin Li, Ying Peng, Yangmei Chen, Baojun Wang, Jinsong Xiao, Honghua Li, Chuanzhu Yan, Yifeng Du, Mouni Tang, Zhiyi He, Haibo Chen, Wei Li, Hong Lin, Shugui Shi, Jianzhong Bi, Huadong Zhou, Yan Cheng, Xiaoping Gao, Yihui Guan, Qiu Huang, Kewei Chen, Xianliang Xin, Jian Ding, Meiyu Geng, Shifu Xiao

**Affiliations:** 1 Department of Geriatric Psychiatry, Shanghai Mental Health Center, Shanghai Jiaotong University School of Medicine, Shanghai, China; 2 Alzheimer’s Disease and Related Disorders Center of Shanghai Jiaotong University, 600 South Wan Ping Road, Shanghai, 200030 China; 3 Department of Psychiatry, West China Hospital of Sichuan University, Chengdu, Sichuan China; 4 Department of Neurology, Sir Run Run Shaw Hospital, Affiliated with the Zhejiang University School of Medicine, Hangzhou, Zhejiang China; 5 Department of Geriatric Psychiatry, Wuxi Mental Health Center, Wuxi, Jiangsu China; 6 Department of Neurology, First Affiliated Hospital of Harbin Medical University, Harbin, Heilongjiang China; 7 Department of Neurology, The Hospital of 81st Group Army PLA, Zhangjiakou, Hebei China; 8 Department of Geriatric Psychiatry, Nanjing Brain Hospital Affiliated to Nanjing Medical University, Nanjing, Jiangsu China; 9 Department of Neurology, Sun Yat-Sen Memorial Hospital, Sun Yat-Sen University, Guangzhou, Guangdong China; 10 Department of Neurology, The Second Affiliated Hospital of Chongqing Medical University, Chongqing, China; 11 Department of Neurology, Baotou Central Hospital, Baotou, Inner Mongolia Autonomous Region China; 12 Department of Neurology, Zhongnan Hospital of Wuhan University, Wuhan, Hubei China; 13 Department of Neurology, Central War Zone General Hospital of the Chinese People’s Liberation Army, Wuhan, Hubei China; 14 Department of Neurology, Qilu Hospital of Shandong University, Jinan, Shandong China; 15 Department of Neurology, Shandong Provincial Hospital, Jinan, Shandong China; 16 Department of Geriatric Psychiatry, Guangzhou Brian Hospital, Guangzhou, Guangdong China; 17 Department of Neurology, The First Hospital of China Medical University, Shenyang, Liaoning China; 18 Department of Neurology, Beijing Hospital, Beijing, China; 19 Department of Neurology, Shanghai Ninth People’s Hospital, Shanghai Jiao Tong University School of Medicine, Shanghai, China; 20 Department of Neurology, Tangdu Hospital, Air Force Military Medical University, Xi’an, Shanxi China; 21 Department of Neurology, The First Hospital Affiliated to AMU (Southwest Hospital), Chongqing, China; 22 Department of Neurology, The Second Hospital of Shandong University, Jinan, Shandong China; 23 Department of Neurology, Daping Hospital, Chongqing, China; 24 Department of Neurology, Tianjin Medical University general hospital, Tianjin, China; 25 Department of Neurology, Hunan Provincial People’s Hospital, Changsha, Hunan China; 26 PET Center Huashan Hospital Fudan University, Shanghai, China; 27 Med-X Research Institution, Shanghai Jiao Tong University, Shanghai, China; 28 Banner Alzheimer’s Institute, Phoenix, AZ USA; 29 Shanghai Green Valley Pharmaceutical Co Ltd, Shanghai, China; 30 State Key Laboratory of Drug Research, Shanghai Institute of Materia Medica, Chinese Academy of Sciences, 555 Zu Chong Zhi Road, Shanghai, 201203 China

**Keywords:** Sodium oligomannate, Efficacy, Safety, Alzheimer’s disease, Clinical trial

## Abstract

**Background:**

Sodium oligomannate (GV-971), a marine-derived oligosaccharide, is a novel agent that may improve cognition in AD patients.

**Methods:**

The 24-week multicenter, randomized, double-blind, placebo parallel controlled clinical trial was conducted in AD in China between 24 October 2011 and 10 July 2013. The study included a 4-week screening/washout period, followed by a 24-week treatment period. Patients were randomized in a 1:1:1 ratio to receive GV-971 900 mg, 600 mg, or placebo capsule in treatment period, respectively. The primary outcome was cognitive improvement as assessed by changes in Alzheimer’s Disease Assessment Scale-cognitive subscale 12-item (ADAS-cog12) scores from baseline to week 24. The secondary efficacy outcomes included CIBIC-Plus, ADCS-ADL, and NPI at 24 weeks after treatment compared with baseline. A subgroup study was assessment of the change in cerebral glucose metabolism by fluorodeoxyglucose positron emission tomography measurements.

**Results:**

Comparing with the placebo group (*n* = 83, change − 1.45), the ADAS-cog12 score change in the GV-971 600-mg group (*n* = 76) was − 1.39 (*p* = 0.89) and the GV-971 900-mg group (*n* = 83) was − 2.58 (*p* = 0.30). The treatment responders according to CIBIC-Plus assessment were significantly higher in the GV-971 900-mg group than the placebo group (92.77% vs. 79.52%, *p* < 0.05). The GV-971 900-mg subgroup showed a lower decline of cerebral metabolic rate for glucose than the placebo subgroup at the left precuneus, right posterior cingulate, bilateral hippocampus, and bilateral inferior orbital frontal at uncorrected *p* = 0.05. The respective rates of treatment-related AEs were 5.9%, 14.3%, and 3.5%.

**Conclusions:**

GV-971 was safe and well tolerated. GV-971 900 mg was chosen for phase III clinical study.

**Trial registration:**

ClinicalTrials.gov, NCT01453569. Registered on October 18, 2011.

## Background

Globally, approximately 35.6 million individuals live with dementia, and this number is predicted to double by 2030 and more than triple by 2050 [1]. The number of people with Alzheimer’s disease (AD) was estimated to be 5.69 million in 2010, with the incidence being 6.25 cases/1000 person-years [2]. The currently available drug treatments for AD target neurotransmitter pathways implicated in disease pathophysiology [3]. While agents such as acetylcholinesterase inhibitors and *N*-methyl-d-aspartic acid receptor antagonists, including memantine, may stabilize or slow the decline of cognition, function, and behavior in patients with AD, they do not slow down the pathological progression [4]. AD is pathologically characterized by senile plaques, neurofibrillary tangles, reactive astrocytosis, and neuronal cell loss. A major component of senile plaques, implicated in the pathophysiology of AD, is the aggregated β-amyloid (Aβ) peptide [5, 6]. Despite the urgent clinical need, in the past decade, disease-modifying therapies such as those targeting amyloid deposition and tau protein have failed to demonstrate clinically relevant efficacy [7–10].

Certain key amino acid residues and specific domains of the Aβ peptide reportedly play an important and unique role in Aβ aggregation [11, 12]. Sodium oligomannate (GV-971) is a marine-derived oligosaccharide [13]; by multitargeting various Aβ subregions, it inhibits Aβ aggregation and destabilizes Aβ aggregates into non-toxic conformers. Moreover, GV-971 can evidently reconstitute the dysbiosis of gut microbiota, reduce metabolite-driven peripheral infiltration of immune cells into the brain, and inhibit neuroinflammation [14]. These effects have been reported to protect synapse integrity and improve cognition in vitro and in a transgenic mouse model of AD [15–18].

Herein, we report the results of a phase II trial designed to investigate the optimal dose, efficacy, and safety of GV-971 capsules in patients with mild-to-moderate AD.

## Methods

### Study design

This 24-week, multicenter, randomized, double-blind, placebo-controlled, phase II trial was conducted in patients with mild-to-moderate AD in geriatric psychiatry, neurology, or geriatrics departments at 24 hospitals (i.e., centers) in China between 24 October 2011 and 10 July 2013. The study included a 4-week screening/washout period, followed by a 24-week treatment period. During the 4-week screening/washout period, all patients received placebo capsules, and during the 24-week treatment period, they underwent the following treatment: three 150-mg GV-971 capsules b.i.d. (900-mg group), two 150-mg GV-971 capsules plus one placebo capsule b.i.d. (600-mg group), or three placebo capsules b.i.d. The trial protocol had two versions: 1.1 and 2.1. The main amendment was the additional clarifications of hypothesis testing in version 2.1 than version 1.1.

The protocol was registered on https://clinicaltrials.gov/ct2/show/NCT01453569 and approved by the China Food and Drug Administration (approval nos. 2006L02492, 2011L00942). The trial was also approved by the Institutional Review Boards of all participating centers from which the approval of the protocol and all related documents was obtained. The trial protocol can be found in Additional file 1.

### Participants

#### Inclusion criteria

Patients aged between 50 and 85 years (inclusive) were eligible to participate in this study, regardless of their gender. They should have been educated to primary school level and above, with the ability to complete pertinent cognitive tests and other rating scales. To qualify for the trial, a subject was expected to fulfill the diagnostic criteria of probable AD according to the National Institute of Neurological and Communicative Disorders and Stroke and the Alzheimer’s Disease and Related Disorders Association (1984), with mild-to-moderate stages (10 ≤ total Mini-Mental State Examination score ≤ 24) [19, 20]. In addition, the total Hachinski Ischemic Scale [21] score had to be ≤ 4 and the total 17-item Hamilton Depression Scale [22] score had to be ≤ 10. Furthermore, it was mandatory for each participant to have stable, reliable caregivers who were expected to be with them for at least 4 days/week for at least 2 h each time while the patient was awake. The caregivers were required to provide valuable information on the Clinician Interview Based Impression of Change–Plus (CIBIC-Plus) [23], Alzheimer’s Disease Cooperative Study–Activities of Daily Living (ADCS-ADL) [24], and Neuropsychiatric Inventory (NPI) [25, 26] assessments. Prior to implementation of any protocol-related procedure or examination, the subjects were required to sign a written informed consent form. If they were unable to sign the form due to impaired cognition, their legal guardians were asked to sign on their behalf.

#### Exclusion criteria

Subjects were excluded if they had participated in any another clinical trial within 30 days prior to the initiation of this study, if they were pregnant or nursing, or if they had dementia due to non-AD causes. Furthermore, they were excluded if they had abnormal laboratory values, including glutamic pyruvic transaminase or glutamic oxaloacetic transaminase > 1.2 times the upper limit of normal; creatinine > 1.2 times the upper limit of normal; white blood cell count, platelet count, or hemoglobin level below the lower limit of normal; and blood glucose level > 1.5 times the upper limit of normal. Patients with systolic blood pressure ≥ 160 mmHg or diastolic blood pressure ≥ 100 mmHg during screening were also not enrolled. In addition, patients were excluded if they had unstable or severe cardiac, pulmonary, hepatic, renal, or hematopoietic disease; a visual or hearing disorder that prevented completion of the neuropsychological test and scale evaluation; and history of alcohol or drug abuse, psychosis (including severe depression), or use of drugs for AD that could not be stopped. Further details pertaining to the inclusion and exclusion criteria have been provided in Additional file 1.

### Randomization and masking

Patients were randomized in a 1:1:1 ratio to receive 900 mg GV-971, 600 mg GV-971 plus placebo, or placebo during the treatment period via an interactive web response system (IWRS) managed by GCP ClinPlus Co., Ltd. (China). All distributed trial drugs had corresponding numbers. Investigators logged on to IWRS for identifying a participant with the help of a center-specific subject ID and visit ID. The system then distributed the drugs to the designated subject using a system-generated drug number.

### Study assessments

Herein, the primary endpoint was cognitive improvement as assessed by changes in Alzheimer’s Disease Assessment Scale-cognitive subscale 12-item (ADAS-cog12) [27] scores from baseline to week 24. The decline of ADAS-cog12 score means the cognitive improvement of patient. The secondary efficacy outcomes included a global assessment based on CIBIC-Plus, along with improvements in ADCS-ADL and NPI scales at 24 weeks post-treatment as compared with baseline data. An assessment of changes in cerebral glucose metabolism, as evaluated by fluorodeoxyglucose positron emission tomography (FDG-PET) measurements of the cerebral metabolic rate for glucose in the brain regions preferentially affected by AD, was also performed in a subgroup of subjects at two sites in Shanghai and Chengdu; the same inclusion and exclusion criteria were used. Safety assessments included evaluations of adverse events (AEs), serious AEs (SAEs), laboratory test results (routine blood and coagulate function tests), electrocardiography data, and vital signs. The study assessment schedule is provided in Additional file 1.

### Statistical analysis

#### Sample size determination

Data collection and analysis were independently managed by GCP ClinPlus Co., Ltd. (China). Power Analysis and Sample Size software (NCSS, LLC, Utah, USA) was used to calculate the sample size. The assumed difference in ADAS-cog mean of − 2.0 and common standard deviation of 4 gave rise to the effect size of 0.5. With 80% power, type I error of 0.05, and computed effect size of 0.5 (∆/δ), we estimated that 70 subjects were needed per group to compare the difference in changes in ADAS-cog12 total scores from baseline to week 24 between the two treatment groups and the placebo group. Considering a drop-off rate of 20%, 84 subjects were expected to be present in each group (total = 252 in the three groups).

#### Efficacy endpoints and analyses

We finally enrolled 255 cases in this study (*n* = 85, 84, and 86 in the placebo, 600-mg, and 900-mg groups, respectively). Data were analyzed in the intention-to-treat population and are reported for the full analysis set and safety set after database lock on August 15, 2013. All data were analyzed using SAS version 9.2. The difference in changes in ADAS-cog12 total scores from baseline to week 24 between the two treatment groups was analyzed using the analysis of covariate model, with baseline ADAS-cog12 total scores serving as the covariate. All results have been expressed as least squares adjusted means and 95% CI.

#### FDG-PET

Subjects in the ^18^F-FDG-PET subgroup underwent PET scanning at baseline and week 24. Each participant was instructed to fast for at least 6 h prior to the PET scan and had serum glucose levels between 63 and 99 mg/100 mL prior to radiotracer administration. During the 45-min radiotracer uptake period after the patient received an intravenous bolus dose of 220–370 MBq (6–10 mCi) FDG, he/she rested quietly in a darkened room with eyes open, in the supine position, and with minimal ambient noise before transferred to the scanning room. FDG-PET scan was then performed using the Siemens Biograph PET/CT (Siemens, Germany), a 30-min emission scan, and a 5-min post-emission CT scan. PET images were reconstructed using filtered back projection, correction for radiation attenuation and scatter. Using SPM8 (https://www.fil.ion.ucl.ac.uk/spm/), the images were spatially normalized to the default MNI (Montreal Neurological Institute) brain template for voxel-wise statistical analysis-based general linear model. The relative cerebral metabolic rate for glucose (CMRgl) was calculated as the standardized uptake value ratio, with the whole brain serving as the reference region. For the brain regions known to be affected by AD, the temporal lobe, hippocampus, posterior cingulate, precuneus, and parietal lobe (all bilateral), we extracted the peak voxel values for each of these regions to examine the baseline–24-week CMRgl change differences between the placebo and 900-mg groups under the general linear model.

## Results

### Baseline characteristics and overview of study subjects

**Fig. 1:**
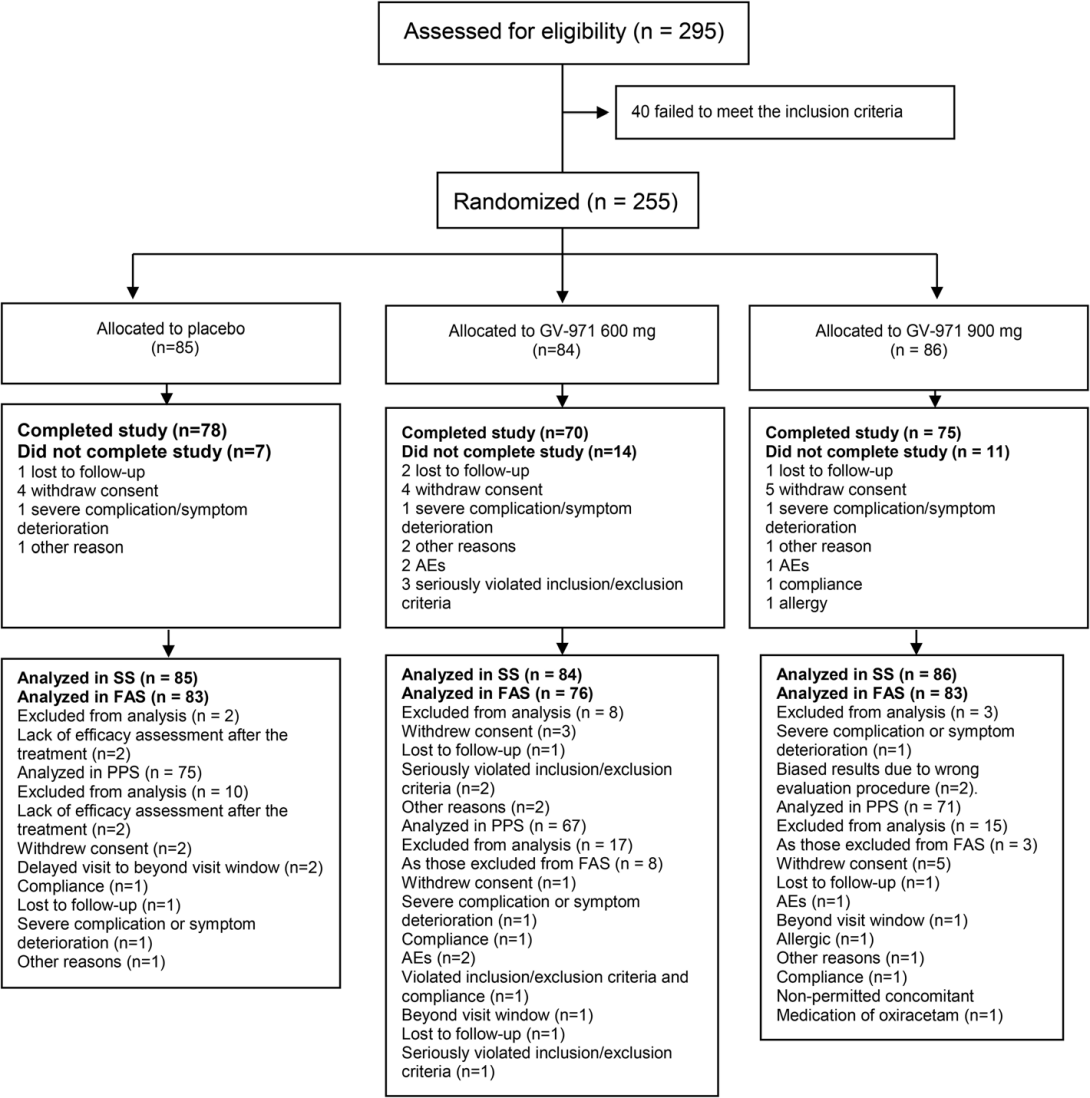
Patient disposition

In total, 295 subjects were assessed for eligibility; 255 patients were enrolled and randomly assigned into the placebo (*n* = 85), 600-mg (*n* = 84), and 900-mg (*n* = 86) groups. Patient disposition is shown in Fig. 1. There were 83 patients in the placebo group, 76 in the 600-mg group, and 83 in the 900-mg group who completed the study and were included in the full analysis set. Overall, 32 (12.5%) patients could not continue their participation as they were lost to follow-up, withdrew consent, showed severe complication/symptom deterioration, experienced AEs or allergic reactions, seriously violated the inclusion/exclusion criteria, or showed non-compliance, among other reasons (Fig. 1). There were 7, 9, and 9 patients in the placebo, 600-mg, and 900-mg groups, respectively, who underwent ^18^F-FDG-PET scans. Baseline demographics were not statistically different among the groups (*p* > 0.05) (Table 1), and baseline demographics for the FDG-PET subgroups were also not statistically different (*p* > 0.05).

**Table 1:** Baseline patient characteristics and demographics of full analysis set

	Placebo (***N*** = 83)	600 mg (***N*** = 76)	900 mg (***N*** = 83)	***p*** value
Gender				0.519^a^
Male	31 (37.4%)	35 (46.1%)	33 (39.8%)	
Female	52 (62.7%)	41 (54.0%)	50 (60.2%)	
Age (years)	70.3 (8.1)	70.3 (8.4)	70.4 (8.5)	1.00^b^
Ethnicity				0.76^a^
Han Chinese	83 (100.0%)	75 (98.7%)	82 (98.8%)	
Others	0 (0.0%)	1 (1.3%)	1 (1.2%)	
Height (cm)	161.7 (8.8)	163.1 (7.2)	162.5 (7.6)	0.52^b^
Weight (kg)	58 (10.4)	60 (9.9)	59 (10.0)	0.20^b^
Education				0.80^a^
6 years	31 (37.4%)	22 (29.0%)	27 (32.5%)	
More than 6 years	52 (62.7%)	54 (71.1%)	56 (67.5%)	
Breathing (times/minute)	18 (2.0)	18 (2.1)	18 (2.1)	0.54^b^
Diastolic blood pressure (mmHg)	126 (12.0)	124 (11.8)	126 (11.2)	0.65^b^
Systolic blood pressure (mmHg)	76 (7.0)	78 (7.4)	76 (8.1)	0.24^b^
Pulse (times/minute)	74 (10.0)	75 (8.9)	74 (8.7)	0.85^b^
HIS	1.3 (0.9)	1.5 (0.9)	1.3 (0.9)	0.88^b^
HAMD	3.3 (2.7)	3.4 (2.8)	3.1 (2.7)	0.28^b^
MMSE	17.5 (4.2)	18.3 (4.8)	18.1 (4.4)	0.20^b^

*HIS* Hachinski Ischemic Scale, *HAMD* Hamilton Depression Scale, *MMSE* Mini-Mental State Examination

^a^Exact Pearson’s chi-squared test

^b^*p* values were determined using two-tailed *t* tests

### Primary efficacy outcome

**Fig. 2:**
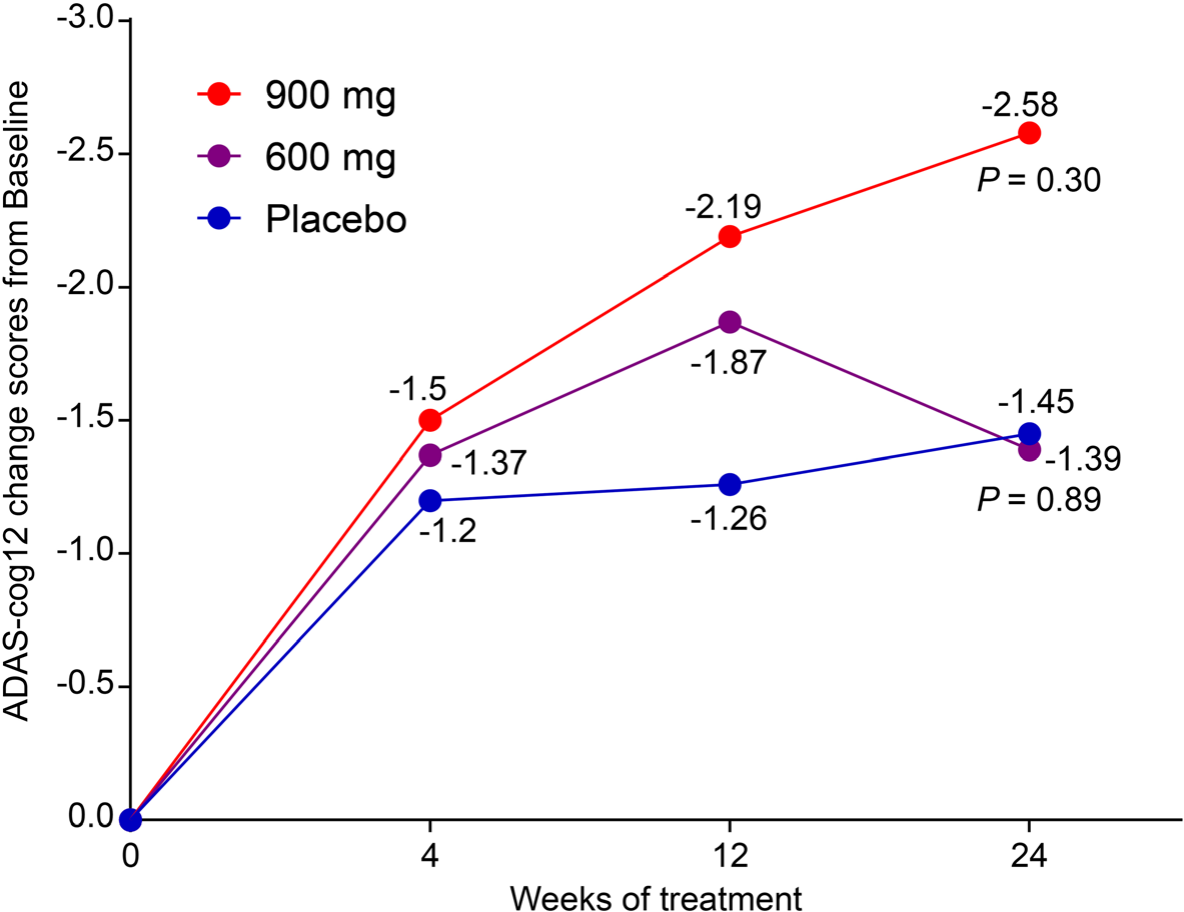
Full analysis set of the primary efficacy outcome of ADAS-cog12 change scores from baseline to week 24 among the three groups. *p* values were derived upon comparison of changes from week 24 to baseline with the placebo group

The effect of treatment on the primary outcomes in the three groups was not significantly different. For the primary efficacy outcome of cognitive improvement at week 24, as measured using ADAS-cog12 total scores, the mean change from the baseline value was − 1.45 in the placebo group (*n* = 83), − 1.39 in the 600-mg group (*n* = 76, *p* = 0.89 in comparison with the placebo group), and − 2.58 in the 900-mg group (*n* = 83, *p* = 0.30 in comparison with the placebo group) (Table 2 and Fig. 2). The least squares adjusted mean changes and 95% CI of ADAS-cog12 total scores from baseline to week 24 were as follows: 900-mg group = − 2.53, 95% CI, − 3.91 to − 1.15; 600-mg group = − 1.34, 95% CI, − 2.88 to 0.19; and placebo group = − 1.50, 95% CI, − 2.97 to − 0.03. Though not statistically different, change in ADAS-cog12 scores from baseline to week 24 in the 900-mg group was numerically greater than that in the placebo group.

**Table 2:** Efficacy analyses for the primary and secondary outcomes with the covariate model of full analysis set

	Placebo (***N*** = 83), mean (SD)	600 mg (***N*** = 76), mean (SD)	900 mg (***N*** = 83), mean (SD)
**ADAS-cog12**			
Baseline	28.1 (12.0)	26.1 (12.4)	26.16 (12.00)
24 Ws	26.7 (14.5)	24.7 (14.4)	23.6 (13.7)
Change	− 1.5 (7.0)	− 1.4 (6.5)	− 2.6 (5.7)
*p* value^a^	–	*p* = 0.89	*p* = 0.30
**ADCS-ADL**			
Baseline	50.9 (17.4)	50.0 (17.4)	53.7 (16.4)
24 Ws	49.8 (17.5)	49.5 (18.7)	53.2 (16.7)
Change	− 1.1 (7.8)	− 0.5 (7.7)	− 0.5 (8.3)
*p* value^a^	–	0.66	0.48
**NPI**			
Baseline	9.9 (13.7)	7.3 (10.9)	7.4 (12.2)
24 Ws	7.8 (11.7)	7.5 (12.2)	6.2 (10.8)
Change	− 2.1 (9.0)	0.2 (7.2)	− 1.1 (11.2)
*p* value^a^	–	0.17	0.94
**CIBIC+**	Cases (%)	Cases (%)	Cases (%)
Significant improvement	3 (3.6)	2 (2.6)	0 (0.0)
Moderate improvement	6 (7.2)	9 (11.8)	13 (15.7)
Slight improvement	32 (38.6)	18 (23.7)	29 (34.9)
No change	25 (30.1)	23 (30.3)	35 (42.2)
Slight deterioration	17 (20.5)	21 (27.6)	4 (4.8)
Moderate deterioration	0 (0.0)	3 (4.0)	2 (2.4)
Significant deterioration	0 (0.0)	0 (0.0)	0 (0.0)
*p* value^b,^*	–	0.11	0.01

*Ws* weeks, *ADAS-cog* Alzheimer’s Disease Assessment Scale-cognitive, *ADCS-ADL* Alzheimer’s Disease Collaborative Study–Activity of Daily Living Scale, *NPI* Neuropsychiatric Inventory, *CIBIC* Clinician’s Interview-Based Impression of Change

^a^Exact Pearson’s chi-squared test

^b^*p* values were determined using two-tailed *t* tests

*Comparison of the percentage of effectiveness in the overall efficacy evaluation (significant improvement + moderate improvement + slight improvement + no change) on the CIBIC-Plus scale of sodium oligomannate capsule and placebo at week 24 of treatment

### Secondary efficacy outcomes

**Fig. 3:**
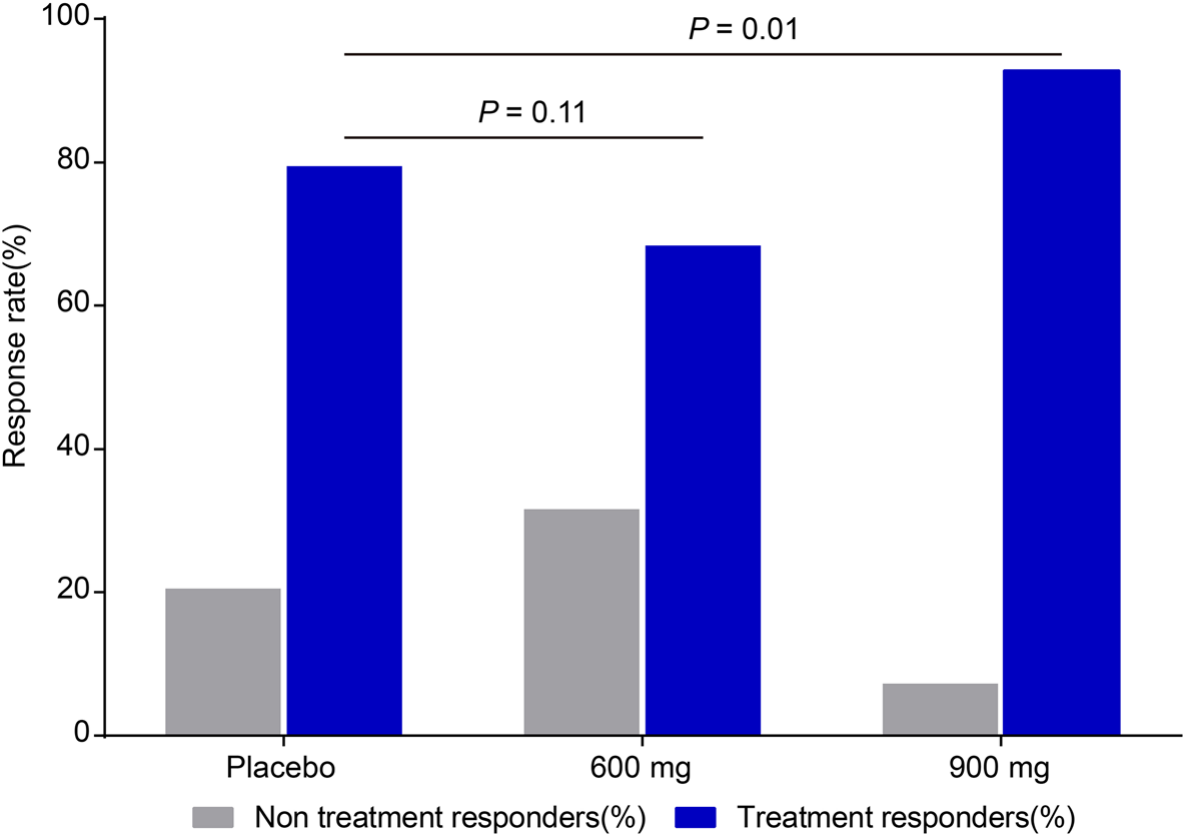
Full analysis set of the secondary efficacy outcome of CIBIC-Plus change score from baseline to week 24 among groups

The assessment of the secondary efficacy outcomes showed that significantly more patients were classified as treatment responders based on CIBIC-Plus at week 24 in the 900-mg group than in the placebo group (Fig. 3). Treatment responders were defined as those with marked improvement, moderate improvement, minimum improvement, or no change on CIBIC-Plus. The difference in the percentage of treatment responders was significant between the 900-mg and placebo groups (92.8% vs. 79.5%; *p* = 0.01), but insignificant between the 600-mg and placebo groups (68.4% vs. 79.5%; *p* = 0.11). For other secondary outcome measures, i.e., the effect of the study drug at week 24 on ADCS-ADL and NPI scales, no significant differences were found between the two treatment groups and the placebo group.

### ^18^F-FDG-PET scan subgroup exploratory analysis

**Fig. 4:**
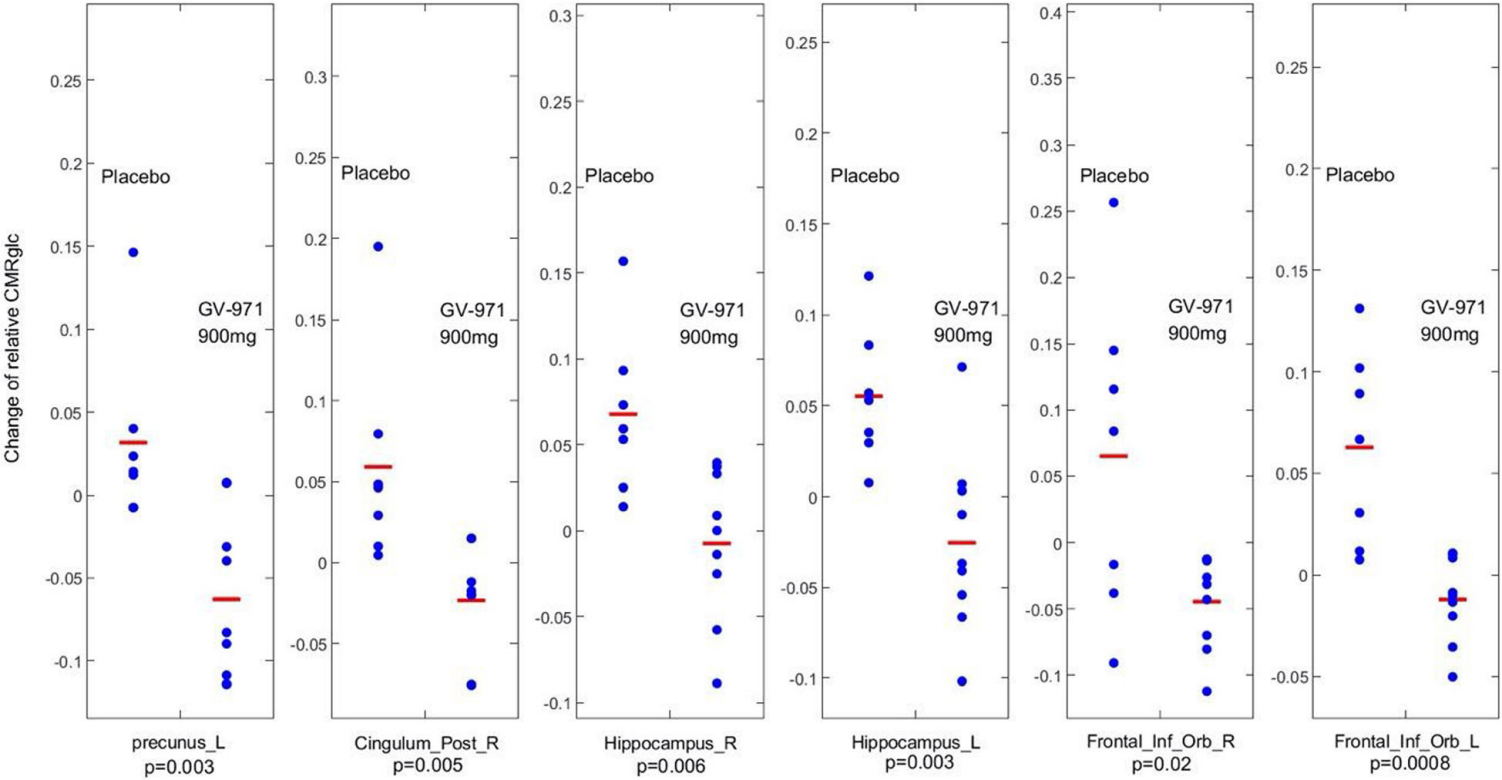
^18^F-FDG-PET measured changes of cerebral glucose metabolism (baseline – follow-up) in several AD related brain regions, revealing an improvement or slower decline in the 900-mg group than in the placebo group. *p* values displayed in the graph are not corrected for multiple comparison, and none survived multiple comparison correction

The effect of GV-971 on cerebral glucose metabolism, as measured using ^18^F-FDG-PET, was evaluated in 7, 9, and 9 patients from the placebo, 600-mg, and 900-mg groups, respectively. CMRgl change differences, not corrected for multiple comparisons, before and after treatment were noted in the brain regions known to be affected by AD between the placebo and the 900-mg groups which were shown in Fig. 4, with the left precuneus (*p* = 0.003), right posterior cingulate cortex (*p* = 0.005), right hippocampus region (*p* = 0.006), left hippocampus (*p* = 0.003), right inferior orbital frontal (*p* = 0.02), and left inferior orbital frontal (*p* = 0.0008) uncorrected for multiple comparisons. However, none of them is significant after multiple comparison corrections. Also, we did not find the group differences in the right precuneus, left posterior cingulate, and bilateral temporal lobe and parietal lobe even with uncorrected *p* = 0.05. Finally, there was no baseline CMRgl difference.

### Safety analysis

The AE profile of GV-971 is summarized in Table 3 and Additional file 2. Most AEs were mild to moderate and needed no treatment. The total rate of AEs was 77.6%, 76.2%, and 59.3% in the placebo, 600-mg, and 900-mg groups, respectively, while the rates of treatment-related AEs were 5.9%, 14.3%, and 3.5% in the aforementioned groups, respectively. The discontinuation rates due to AEs were 1.2% in the placebo group, 3.6% in the 600-mg group, and 3.5% in the 900-mg group. There were 14 reported SAEs (seen as 15 case-events), including six SAEs in the placebo group (six case-events, 7.1%), five in the 600-mg group (six case-events, 6.0%), and three in the 900-mg group (three case-events, 3.5%). Thirteen SAEs, including four in the 600-mg group, three in the 900-mg group, and six in the placebo group, were evaluated by investigators to be definitely unrelated to the study drug. One SAE, behavioral and psychiatric symptoms of dementia, in the 600-mg group was possibly related to the study drug. The assessment of vital signs and physical examination results yielded essentially no abnormalities or intergroup differences. Laboratory test results and ECG findings were similar between the groups at 24 weeks post-treatment as compared with the baseline data.

**Table 3:** Summary of adverse events, serious AEs of all groups

Items	Placebo (***N*** = 85)	600 mg (***N*** = 84)	900 mg (***N*** = 86)	***p*** value
	Cases (%)	Cases (%)	Cases (%)	
AEs	35 (41.2)	31 (36.9)	24 (27.9)	0.18
NR Med	32 (37.7)	26 (30.9)	21 (24.4)	0.17
RE Med	3 (3.5)	5 (6.0)	3 (3.5)	0.67
SAEs	6 (7.1)	5 (6.0)	3 (3.5)	0.58
NR Med	6 (7.1)	4 (4.8)	3 (3.5)	0.56
RE Med	0 (0.0)	1 (1.2)	0 (0.0)	0.36
Drop-off due to AE	1 (1.2)	3 (3.6)	3 (3.5)	0.56

*AEs* adverse events, *SAEs* serious AEs, *AEs NR Med* non-medication-related AEs, *AEs RE Med* medication-related AEs (definitely, probable, possible, and suspiciously)

*p* values related to AE and SAE were analyzed using the chi-squared test

## Discussion

Oligomannurarate, a marine-derived oligosaccharide, has been identified as a multi-region binder of Aβ. Through its multitargeting of various subregions of Aβ, GV-971 inhibits Aβ aggregation and destabilizes Aβ aggregates into non-toxic conformers [15–17]. Other important mechanism of GV-971 is that it can reconstitute the dysbiosis of gut microbiota, reduce metabolite-driven peripheral infiltration of immune cells into the brain, and inhibit neuroinflammation [14]. Studies have shown that GV-971 crosses the blood-brain barrier and appears to have a good tolerability profile. In the phase 1 study in healthy volunteers, the compound can be detected in blood. Gender and age have no effects on the pharmacokinetic (PK) profiles of GV-971. GV-971 was shown to have a dose linear PK profiles in the blood, with a half-life of ~ 11 h and a good safety profile. In this phase II study, as compared with placebo, the oral administration of GV-971 at 600 mg or 900 mg/day for 24 weeks did not have any significant positive effects on the primary endpoint in patients with mild-to-moderate AD. However, the trial results provided useful information pertaining to the clinical efficacy of GV-971. No significant effect was detected on the primary endpoint of ADAS-cog12 changes at 24 weeks in the 900-mg group as compared to those in the placebo group. The secondary efficacy outcome based on CIBIC-Plus also suggested that administering 900 mg GV-971 improved the global function in patients with mild-to-moderate AD. AD dementia is well known as a progressive disease [1]. We referred to the data processing methods of CIBIC+ in other AD clinical research [28] and considered the no change in the CIBIC+ scale at endpoint as treatment response. The percentage of treatment responders was significantly higher for those in the 900-mg group as compared to those in the placebo group. The placebo effect seemed to appear in nervous system drugs, such as drugs for dementia and depression [29–31]. There have been studies reporting the variation of placebo effects among different racial and ethnic groups in the East Asia [32, 33]. In our case, the unmet cares given by the physicians, in the aspects of the clinical encounter, including the tone and emotional presence of the clinician, and the support and engagement involved in a consultation, have been found to directly shape clinical outcomes. As in the reports, the placebo effect appeared in all of the groups.

AD is clinically characterized by progressive cognitive impairment, which is associated with impaired cerebral glucose metabolism [34]. In fact, cerebral glucose hypometabolism occurs during early AD, and ^18^F-FDG-PET studies have consistently reported progressive reductions in cerebral glucose metabolism, the extent and topography of which correlate with symptom severity [35]. In this 24-week, double-blind, randomized, placebo-controlled trial, we employed FDG-PET to explore the effects of GV-971 on regional neuronal activity in patients with mild-to-moderate AD in a small subcohort of only 7, 9, and 9 subjects from the 3 groups. We acknowledge that the FDG-PET measure was exploratory, so was our analysis which did not survive multiple comparison correction; thus, our findings should be interpreted with caution. ^18^F-FDG-PET data showed that treatment with GV-971 at 900 mg/day slowed the impairment of cerebral glucose metabolism in several AD-associated brain regions. Given the exploratory nature (including not corrected for multiple comparisons), our results seemed to imply that the effects of GV-971 on neuronal function are not simply compensatory in the brain regions affected by AD.

Treatment with GV-971 was safe and well tolerated. Most AEs were either infections, gastrointestinal disorders, or nervous system disorders. Moreover, as compared with the placebo group, none of the AEs was more prevalent in the active treatment groups. Although the primary outcome measure did not reach statistical significance, findings from this phase II trial are still informative. First, our results suggest that GV-971 dosage should be 900 mg/day whenever a new phase III study is being conducted. Second, no difference of the ADAS-cog change between the placebo and 900-mg groups suggests that the trial duration should be > 24 weeks in the future to comprehensively assess the efficacy of GV-971.

According to the results of this phase II study, a future phase III trial of GV-971 could be able to reduce the ADAS-cog12 score from baseline by at least 1.4, which, together with variability information, can be converted to an effect size (∆/δ) of 0.23. Assuming the two-arm design (placebo vs. 900 mg GV-971), such a study can be conducted with 315 subjects/group with 80% power to detect differences in the change between the GV-971 and placebo groups for 36-week treatment duration phase III trial, with a two-sided α level of 0.05.

This phase II study has three important limitations. First, biomarkers associated with AD were not included as a part of the inclusion/exclusion criteria for AD diagnosis at the protocol design and to serve as possible additional output measures. The lack of biomarkers may thus lead to some bias in AD diagnosis. Second, there could have been an evaluation bias at few sites as investigators were not continuously trained to ensure consistency in assessing cognitive test data. Third, there was a potential of continuous improvement in the 900-mg group at endpoint, so the 24-week treatment duration may not be long enough to witness the effectiveness of GV-971. Therefore, it is necessary to increase the treatment duration in future phase III trials of GV-971. Last, the cholinesterase inhibitors (ChEIs) have been shown to be effective for treating mild-to-moderate AD. We shall consider the use of ChEIs as a “standard of care” in future GV-971 trial.

## Conclusions

In conclusion, this phase II trial provided the evidence that GV-971 was safe and well tolerated. A decision was made to carry out a phase III clinical trial for GV-971 with the chosen dosage 900 mg.

## Supplementary information

**Additional file 1.**

**Additional file 2.**

## Data Availability

The datasets used and/or analyzed during the present study are available from the corresponding author on reasonable request.

## Abbreviations

GV-971: Sodium oligomannate
AD: Alzheimer’s disease
ADAS-cog12: Alzheimer’s Disease Assessment Scale-cognitive subscale 12-item
CIBIC-Plus: Clinician Interview Based Impression of Change–Plus
ADCS-ADL: Alzheimer’s Disease Cooperative Study–Activities of Daily Living
NPI: Neuropsychiatric Inventory
Aβ: β-Amyloid
b.i.d.: Bis in die
IWRS: Interactive web response system
ID: Identity document
FDG-PET: Fluorodeoxyglucose positron emission tomography
AE: Adverse event
SAE: Serious AE
CI: Confidence interval
