# Supplementary material for: A phase II randomized trial of sodium oligomannate in Alzheimer’s dementia
Source: Alzheimers Res Ther. 2020 Sep 14;12:110. doi: 10.1186/s13195-020-00678-3 (PMC7489025; doi:10.1186/s13195-020-00678-3)
Supplement: Supplementary file 1 — Additional file 1. [file 13195_2020_678_MOESM1_ESM.doc]

Clinical Study Approval No.: 2006L02492

**A Multi-Center, Randomized, Double-Blind, Placebo Parallel Controlled, Phase II Clinical Trial to Evaluate the Dosage, Efficacy and Safety of** **Sodium oligomannate Capsules (971) in Treatment of Mild to Moderate Alzheimer’s Disease**

| **Leading Clinical Research Institute：** | Shanghai Mental Health Center, Shanghai Jiao Tong University School of Medicine | |
| --- | --- | --- |
| **Principal Investigator (PI):** | Prof. Shi-fu Xiao |  |
| **Statistical Analysis Company:** | GCP ClinPlus Co.，Ltd. |  |
| **Sponsor:** | Shanghai Green Valley Pharmaceutical Co., Ltd. |  |
| **CRO:** | GCP ClinPlus Co.，Ltd. |  |
| **Protocol No.:** | 9712011-1 |  |
| **Version No.：** | V2.1 |  |
| **Date:** | 12-Dec-2011 |  |

This clinical trial protocol is a confidential document and is only used by investigators and Ethics Committee of study center involving in the clinical trial with Sodium oligomannate capsules (971capsules). Disclosure and publication are prohibited.

# Signature of Sponsor/Clinical Monitoring/Statistical Analysis Companys

**Sponsor**

I have read this protocol (Protocol No.: 9712011-1, version No.: 2.1, version date: 12-Dec-2011), agreed upon the contents to conduct the clinical study accordingly. I will follow the GCP regulations with rigor to perform the sponsor’s duties with highest standarnd. I will be responsible to originate, organize and support this clinical study, especially purchase insurance for all participants for possible study related injury or death in the trial, provide economic compensation and legal guarantee for investigators

I will keep this protocol and all relevant materials confidential.

**Sponsor:** **Shanghai Green Valley Pharmaceutical Co., Ltd.**

Director (Singature): Date YYYY MMM DD

**CRA**

I will fulfill the duties as a monitor in accordance with the GCP regulations.

I have read this protocol. I will carefully perform the monitor’s duties according to the GCP regulations to ensure that the rights of the subjects in the clinical study are protected, the data of the study records and reports are true, accurate and complete, and the study follows approved protocols, drug clinical study management practices, and relevant regulations.

**Clinical Monitoring Company:GCP ClinPlus Co.，Ltd.**

Director (Singature): Date YYYY MMM DD

**Biostatistical Analysis**

I will carefully perform the duties of statisticians according to the GCP regulations.

**Statistical Company：GCP ClinPlus Co.，Ltd.**

Statistician (Singature): Date YYYY MMM DD

# Investigator’s signature

I have received the Investigator’s Brochure, understood the process of this drug study and been informed that updated Investigator’s Brochure will be provided in time.

I have read this protocol (Protocol No.: 9712011-1, version No.: 2.1, version date: 12-Dec-2011). The study will be conducted according to moral, ethical and scientific principles specified in the Declaration of Heisinki and China GCP. I agree to conduct this clinical study according to this protocol design and regulations.

I will be responsible to make clinically related medical decisions to ensure subjects receive timely appropriate treatment in case of adverse events during the study. I understand requirements on correct reporting of serious adverse events and will record and report these events according to requirements.

I promise to truly, accurately, completely, timely and legally record data in case report. I will accept monitoring, audit and inspection by monitors or auditors and drug supervision and administration department to ensure quality of this clinical study.

I agree that study results will be used for drug registration and publication.

I will submit a copy of curriculum vitae to the Ethics Committee before initiation of the study, which may be also submitted to drug administration department for review.

Clinical Research Institute：

Principal investigator (signature): Date YYYY MMM DD

**Contents**

[Signature of Sponsor/Clinical Monitoring/Statistical Analysis Companys 2](#__RefHeading___Toc4777587)

[Investigator’s signature 3](#__RefHeading___Toc4777588)

[Abbreviations 8](#__RefHeading___Toc4777589)

[Protocol Synopsis 10](#__RefHeading___Toc4777590)

[1. Study Background 16](#__RefHeading___Toc4777591)

[2. Theoretical Basis 17](#__RefHeading___Toc4777592)

[3. Overview of Preclinical Study 24](#__RefHeading___Toc4777593)

[3.1 Therapeutic Effect of Sodium oligomannate on Learning and Memory Function in Senile Dementia 24](#__RefHeading___Toc4777594)

[3.1.1 Effect of Sodium oligomannate on Learning and Memory Function of Animals with Alzheimer's Disease Induced by Intracerebral Injection of Aβ_1-40_ 24](#__RefHeading___Toc4777595)

[3.1.2 Effect of Sodium oligomannate on Learning and Memory Function of Animals with Alzheimer's Disease Induced by Intracerebral Injection of Aβ_25-35_ 25](#__RefHeading___Toc4777596)

[3.1.3 Effect of Sodium oligomannate on Learning and Memory Function of Animals with Alzheimer's Disease Induced by Subcutaneous Injection of D-galactose 26](#__RefHeading___Toc4777597)

[3.1.4 Effect of Sodium oligomannate on Learning and Memory Function of Animals with Alzheimer's Disease Induced by Intraperitoneal Injection of Scopolamine 27](#__RefHeading___Toc4777598)

[3.1.5 Protective Effect of Sodium oligomannate on Nerve Cell Impairment induced by Aβ in vitro 28](#__RefHeading___Toc4777599)

[3.2 Study on the Mechanism of Sodium oligomannate to Improve the Learning and Memory Function of Alzheimer's Disease 29](#__RefHeading___Toc4777600)

[4. Study Objectives 30](#__RefHeading___Toc4777601)

[4.1 Primary Objective 30](#__RefHeading___Toc4777602)

[4.2 Secondary Objectives 30](#__RefHeading___Toc4777603)

[5. Study Design 30](#__RefHeading___Toc4777604)

[5.1 Study Rationale 30](#__RefHeading___Toc4777605)

[5.2 Overview of Study Design 31](#__RefHeading___Toc4777606)

[5.3 Multi-center 31](#__RefHeading___Toc4777607)

[5.4 Randomization 31](#__RefHeading___Toc4777608)

[5.5 Schedule of Events 32](#__RefHeading___Toc4777609)

[6. Selection of Subjects 35](#__RefHeading___Toc4777610)

[6.1 Study Population 35](#__RefHeading___Toc4777611)

[6.1.1 Diagnostic Criteria 35](#__RefHeading___Toc4777612)

[6.1.1.1 AD Diagnostic Criteria 35](#__RefHeading___Toc4777613)

[6.1.2 Grading of AD severity 36](#__RefHeading___Toc4777614)

[6.1.2.1 Mild 36](#__RefHeading___Toc4777615)

[6.1.2.2 Moderate 36](#__RefHeading___Toc4777616)

[6.1.2.3 Severe 37](#__RefHeading___Toc4777617)

[6.2 Inclusion Criteria 37](#__RefHeading___Toc4777618)

[6.3 Exclusion Criteria 38](#__RefHeading___Toc4777619)

[6.4 Uncompleted Cases 39](#__RefHeading___Toc4777620)

[6.4.1 Drop-off Criteria 39](#__RefHeading___Toc4777621)

[6.4.2 Withdrawal Criteria 39](#__RefHeading___Toc4777622)

[6.4.3 Termination Criteria 40](#__RefHeading___Toc4777623)

[7. Study Drug 40](#__RefHeading___Toc4777624)

[7.1 Strength, Dosage Form and Manufacturer 40](#__RefHeading___Toc4777625)

[7.2 Drug Package 40](#__RefHeading___Toc4777626)

[7.3 Dosage and Administration 41](#__RefHeading___Toc4777627)

[7.3.1 Drug in the run-in period 41](#__RefHeading___Toc4777628)

[7.3.2 Treatment Period 41](#__RefHeading___Toc4777629)

[7.4 Management and Storage of Study Drugs 41](#__RefHeading___Toc4777630)

[7.5 Drug Distribution and Record 42](#__RefHeading___Toc4777631)

[7.6 Evaluation of Drug Compliance 42](#__RefHeading___Toc4777632)

[7.7 Drug Accountability 43](#__RefHeading___Toc4777633)

[7.8 Concomitant Medication 43](#__RefHeading___Toc4777634)

[7.8.1 Prohibited Drugs Throughout the Study 43](#__RefHeading___Toc4777635)

[7.8.2 Permitted Drugs in the Trial 44](#__RefHeading___Toc4777636)

[8. Study Evaluations and Procedures 44](#__RefHeading___Toc4777637)

[8.1 Screening/Run-in Evaluation——Visit 0（Week-4） 44](#__RefHeading___Toc4777638)

[8.2 Baseline Evaluation – Visit 1 (Day 0) 45](#__RefHeading___Toc4777639)

[8.3 Week-4 after Treatment – Visits 2 (4 weeks±3 days) 46](#__RefHeading___Toc4777640)

[8.4 Week-12 after Treatment – Visits 3 (12 weeks±7 days) 47](#__RefHeading___Toc4777641)

[8.5 Week-24 after Treatment – Visits 4 (24 weeks±7 days) 48](#__RefHeading___Toc4777642)

[8.6 Phone Follow-up 49](#__RefHeading___Toc4777643)

[8.7 Unscheduled Visits (study required or premature withdrawal from the trial) 49](#__RefHeading___Toc4777644)

[8.8 Relevant Treatments after the End of Trial 49](#__RefHeading___Toc4777645)

[8.9 Central Laboratory Tests 49](#__RefHeading___Toc4777646)

[8.10 PET Imaging Examination 50](#__RefHeading___Toc4777647)

[8.11 Safety Assessment 50](#__RefHeading___Toc4777648)

[9. Endponints 50](#__RefHeading___Toc4777649)

[9.1 Efficacy Endpoints 50](#__RefHeading___Toc4777650)

[9.1.1 Primary Efficacy Endpoint 50](#__RefHeading___Toc4777651)

[9.1.2 Secondary Efficacy Endpoints 51](#__RefHeading___Toc4777652)

[9.1.3 Subgroup Efficacy Endpoints 51](#__RefHeading___Toc4777653)

[9.1.4 Efficacy Evaluation Scales 51](#__RefHeading___Toc4777654)

[9.1.4.1 ADAS-Cog 51](#__RefHeading___Toc4777655)

[9.1.4.2 CIBIC-plus 51](#__RefHeading___Toc4777656)

[9.1.4.3 ADCS-ADL 52](#__RefHeading___Toc4777657)

[9.1.4.4 NPI 52](#__RefHeading___Toc4777658)

[9.2 Observation of Drug Concentration 52](#__RefHeading___Toc4777659)

[9.3 Safety Evaluation 52](#__RefHeading___Toc4777660)

[9.3.1 Adverse Event (AE) 53](#__RefHeading___Toc4777661)

[9.3.1.1 Definition of AE 53](#__RefHeading___Toc4777662)

[9.3.1.2 Relationship with the Study Drug 53](#__RefHeading___Toc4777663)

[9.3.1.3 Severity Grading of AE 54](#__RefHeading___Toc4777664)

[9.3.2 Adverse Drug Reactions (ADR) 54](#__RefHeading___Toc4777665)

[9.3.3 Clinical Laboratory Abnormalities 54](#__RefHeading___Toc4777666)

[9.3.4 Serious Adverse Event (SAE) 55](#__RefHeading___Toc4777667)

[9.3.5 Actions Taken for AE 55](#__RefHeading___Toc4777668)

[9.3.5.1 Record and Report 55](#__RefHeading___Toc4777669)

[9.3.5.2 Emergent Unblinding 56](#__RefHeading___Toc4777670)

[9.3.5.3 Treatment of Patients 56](#__RefHeading___Toc4777671)

[9.3.5.4 Follow-up of Adverse Events 57](#__RefHeading___Toc4777672)

[10. Regulations of Blinding, Preservation of Blind Codes and Unblinding 57](#__RefHeading___Toc4777673)

[10.1 Blinding and Preservation of Blind Codes 57](#__RefHeading___Toc4777674)

[10.2 Emergent Unblinding 58](#__RefHeading___Toc4777675)

[10.3 Regulations of Unblinding 58](#__RefHeading___Toc4777676)

[11. Data Management 58](#__RefHeading___Toc4777677)

[11.1 Data Collection and Revision 58](#__RefHeading___Toc4777678)

[11.2 Verification of Source Records 58](#__RefHeading___Toc4777679)

[11.3 Blind Review and Unblinding 58](#__RefHeading___Toc4777680)

[11.4 Data Lock 59](#__RefHeading___Toc4777681)

[12. Statistical Analysis Plan 59](#__RefHeading___Toc4777682)

[12.1 Sample Size 59](#__RefHeading___Toc4777683)

[12.2 Study Parameters 60](#__RefHeading___Toc4777684)

[12.3 Analysis Sets 60](#__RefHeading___Toc4777685)

[12.3.1 Full analysis set (FAS) 60](#__RefHeading___Toc4777686)

[12.3.2 Per-protocol (PP) set 60](#__RefHeading___Toc4777687)

[12.3.3 Safety set (SS) 60](#__RefHeading___Toc4777688)

[12.4 General Considerations of Statistical Analysis 60](#__RefHeading___Toc4777689)

[12.5 Analysis of Statistical Methods 61](#__RefHeading___Toc4777690)

[12.5.1 Efficacy Analysis 61](#__RefHeading___Toc4777691)

[12.5.2 Primary Efficacy Endpoint 61](#__RefHeading___Toc4777692)

[12.5.3 Secondary Efficacy Endpoints 62](#__RefHeading___Toc4777693)

[12.6 Safety Analysis 62](#__RefHeading___Toc4777694)

[13. Quality Control of the Trial 62](#__RefHeading___Toc4777695)

[13.1 Consistent Training of Scale Rating 62](#__RefHeading___Toc4777696)

[13.2 Data Monitoring committee (DMC) 63](#__RefHeading___Toc4777697)

[13.3 Clinical Monitoring 63](#__RefHeading___Toc4777698)

[13.4 Collaborative Monitoring and Audit 63](#__RefHeading___Toc4777699)

[14. Ethical Requirements 64](#__RefHeading___Toc4777700)

[15. Informed Consent Form/Data Protection Agreement 64](#__RefHeading___Toc4777701)

[16. Withdrawal from the Clinical Study 65](#__RefHeading___Toc4777702)

[17. Patients’ Privacy 65](#__RefHeading___Toc4777703)

[18. Revision of the Clinical Protocol 65](#__RefHeading___Toc4777704)

[19. Case Report Form (CRF) 65](#__RefHeading___Toc4777705)

[20. Verification of Source Data 66](#__RefHeading___Toc4777706)

[21. Publication 66](#__RefHeading___Toc4777707)

[22. Preservation of Study Data 66](#__RefHeading___Toc4777708)

[23. Responsibilities of Each Party 66](#__RefHeading___Toc4777709)

[24. Clinical Research Institution 66](#__RefHeading___Toc4777710)

[25.Reference 67](#__RefHeading___Toc4777711)

[26. Appendix 69](#__RefHeading___Toc4777712)

[Appendix 1 Mini-Mental State Examination (MMSE) 69](#__RefHeading___Toc4777713)

[Appendix 2 Hachinski Ischemic Scale score (HIS) 71](#__RefHeading___Toc4777714)

[Appendix 3 Hamilton Depression Scale (HAMD) 72](#__RefHeading___Toc4777715)

[Appendix 4 Alzheimer's Disease Assessment Scale - cognitive portion (ADAS-cog) 75](#__RefHeading___Toc4777716)

[Appendix 5 Clinician impression to changes through interviews (CIBIC-plus) 80](#__RefHeading___Toc4777717)

[Appendix 6 Alzheimer's Disease Cooperative Study—Activities of Daily Living (ADCS-ADL) 95](#__RefHeading___Toc4777718)

[Appendix 7 Neuropsychiatric Inventory - clinician rating scale (NPI) 102](#__RefHeading___Toc4777719)

[Summary 124](#__RefHeading___Toc4777720)

| PS1 | Presenilin 1 |
| --- | --- |
| P-tau | Phosphorylated tau protein |
| T-tau | Total tau protein |
| QOL-AD | Quality of Life Scale - Alzheimer's Disease |
| RBC | Red blood cel |
| SAE | Serious adverse event |
| SD | Standard deviation |
| SFDA | State Food and Drug Administration |
| SOD | Superoxide dismutase |
| SP | Senile plaques |
| SS | Safety set |
| t1/2 | Half life |
| TBIL | Total bilirubin |
| Tmax | Time to maximum concentration |
| TNF－α | Tumor necrosis factor-α |
| TP | Total protein |
| TSH | Thyrotropin stimulating hormone |
| VitB_12_ | Vitamin B_12_ |
| WBC | White blood cell |
| LOCF | Last observation carried forward |
| LY% | Percentage of lymphocytes |
| HIS | Hachinski Ischemic Scale |
| MMSE | Mini Mental State Examination |
| MPV | Mean platelet volume |
| MRI | Magnetic resonance imaging |
| NFT | Neurofibrillary tangle |
| NINCDS-ADRDA | National Institute of Neurological and Communicative Disorders and Stroke and the Alzheimer's Disease and Related Disorders Association |
| NPI-C | The Neuropsychiatric Inventory-Clinician rating scale |
| ATP | Adenosine triphosphate |
| AUC | Area under the curve |
| PLT | Platelet |

# Abbreviations

| FIB | Fibrinogen |
| --- | --- |
| FT3 | Free triiodothyronine |
| FT4 | Free tetraiodothyronine |
| GAGS | Glycosaminoglycan |
| GGT | γ-glutamyl transpeptidase |
| PPS | Per Protocol Set |
| GR% | Neutrophil percentage |
| GSH-PX | Glutathione peroxidase |
| HAMD | Hamilton Depression Scale |
| Hb | Hemoglobin |
| HIV | Human immunodeficiency virus |
| ITT | Intention-to-treat analysis |
| Cmax | Maximum concentration |
| Cr | Creatinine |
| CRF | Case report form |
| CSF | Cerebrospinal fluid |
| DBIL | Direct bilirubin |
| ECG | Electrocardiogram |
| BUN | Blood urea nitrogen |
| AchE | Acetylcholinesterase |
| AD | Alzheimer's disease |
| ADAS-Cog | Alzheimer's Disease Assessment Scale-Cognitive |
| ADCS-ADL | Alzheimer’s Disease Collaborative Study – Activity of Daily Living Scale |
| AE | Adverse event |
| ALB | Albumin |
| ALT | Alanine aminotransferase |
| ALP | Alkaline phosphatase |
| ANCOVA | Analysis of covariance |
| APP | Amyloid precursor protein |
| AST | Aspartate aminotransferase |
| FAS | Full analysis set |
| Aβ | Amyloid-β |
| IWRS | Interactive Web Response System |
| EDC | Electronic data capture |

# Protocol Synopsis

| **Study Title** | A Multi-center, Randomized, Double-blind, Placebo parallel controlled, Dose-finding Phase II Clinical Trial to Evaluate the Efficacy and Safety of Sodium oligomannate capsules (971 capsules) in Treatment of Mild to Moderate Alzheimer’s Disease。 |
| --- | --- |
| **Study objectives** | **Primary objective：**To explore the optimal therapeutic dose of Sodium oligomannate capsules (971 capsules) for the treatment of mild to moderate Alzheimer's disease and to evaluate its effectiveness and safety  **Secondary objectives:**  1．To evaluate the effects of Sodium oligomannate capsules on biomarkers (Aβ40, Aβ42, T-tau, P-tau) in cerebrospinal fluid  2．To determine the concentration of Sodium oligomannate in cerebrospinal fluid and blood at steady state. |
| **Clinical trial approval No.** | 2006L02492 |
| **Study design** | Multicenter, randomized, double-blind, placebo-controlled, dose-exploration |
| **Test and Reference drug** | **Test drug:** Sodium oligomannate capsules (971 capsules), each capsule contains 150 mg Sodium oligomannate .  **Reference drug:** placebo, identical to Sodium oligomannate capsule in appearance, package, label and other characteristics. |
| **Study subjects** | Patients with mild to moderate Alzheimer’s disease |
| **Sample size** | It is planned to enroll approximately 252 subjects who will be randomized into 3 groups (at 600mg, n=84; at 900mg, n=84, Placebo, n=84). Each center will compete for subjects’ enrolment. |
| **Duration** | The study includes a screening/run-in period and a double-blind treatment period. |
| **Treatment Regimen** | This study includes 3 dose groups, i.e. the low-dose group (Sodium oligomannate capsules at 600 mg), or the high-dose group(Sodium oligomannate capsules at 900 mg) and the control group (Placebo). Dose regimen at screenin/run-in period: 3 capsules a time, twice a day in the morning and evening, respectively. On the last day of the 4-week screening/ run-in period, drug compliance of the subject entering the run-in period will be evaluated, and the inclusion/exclusion criteria will be reviewed. Only subjects with drug compliance within 80%～120% and eligible after review can be randomized to a 24-week treatment period. For subjects entering the treatment period, they will be randomized into different dose groups according to the time of visit at baseline. The low-dose group (600 mg): for each dose, 2 Sodium oligomannate capsules (971 capsules) + 1 placebo. The high-dose group (900 mg): for each dose, 3 Sodium oligomannate capsules (971 capsules).The control group: for each dose, 3 placebo capsules. For each dose group, the dose regimen is twice a day, oral administration using warm water, in the morning and evening respectively. |
| **Inclusiong Criteria** | 1．Male and female subjects aged 50-85 years (inclusive).  2．Education: ≥ 6 years.  3．Meet diagnostic criteria of probable AD according to National Institute of Neurological and Communicative Disorders and Stroke and the Alzheimer's Disease and Related Disorders Association (NINCDS-ADRDA) (1984).  4．Patients with mild to moderate disease, i.e. 10points ≤ MMSE total score ≤24 points.  5．Hachinski Ischemia Scale (HIS) total score ≤4 points.  6．Hamilton Depression Scale/17-item (HAMD) total score ≤10 points.  7．Subjects should have stable, reliable caregivers, or at least have frequent contact with caregivers (at least 4 days every week, at least 2 hours every day), and caregivers will help patients in participation in this study. Caregivers must accompany subjects to participate in study visits and have sufficient interaction and communication with subjects, so as to provide valuable information on scales including CIBIC-plus(see Appendix 6) , ADCS-ADL(see Appendix 7) and NPI(see Appendix8).  8．The patient agrees to participate in the trial and provides the ICF by him/her or his/her guardian prior to the study. |
| **Exclusion Criteria** | 1．Subjects with previous participation in any other clinical trial within 30 days prior to this study.  2．Pregnant and lactating women.  3．Paitents with allergic constitution.  4．Dementia due to other reasons: vascular dementia, central nervous system infection (e.g. AIDS, syphilis), Creutzfeldt-Jakob disease, Huntington's chorea, Parkinson’s disease, dementia with Lewy bodies, traumatic dementia, other physical and chemical factors (e.g. drug poisoning, alcoholism, carbon monoxide poisoning), significant physical illness (e.g. hepatic encephalopathy, pulmonary encephalopathy), intracranial occupying lesion (e.g. subdural hematoma, brain tumor), endocrine disorders (e.g. thyroid disease, parathyroid disease) and dementia caused by vitamin deficiency or other factors.  5．Previous nervous system disorders (including stroke, optic neuromyelitis, Parkinson’s disease, epilepsy).  6．Abnormal laboratory indicators: liver function (ALT, AST) > 1.2× ULN, Cr > 1.2× ULN, WBC count＜4.0×10^9^, platelet＜100×10^9^, haemoglobin <100g/L, random blood glucose >1.2× ULN.  7．Uncontrolled hypertension: systolic blood pressure ≥160 mmHg or diastolic blood pressure ≥100 mmHg.  8．Unstable or severe cardiac, pulmonary, hepatic, renal or hematopoietic disease (including unstable angina, uncontrolled asthma, active gastric bleeding and cancer).  9．Visual or hearing disorder to prevent completion of neuropsychological test and scale evaluation.  10．CT or MRI examination within 1 year prior to enrollment showed clinically significant focal lesions.  11．Alcohol or drug abuse.  12．Psychiatric patients, including those with severe depression  13．Subjects with ongoing AD treatment which can not be discontinued.  14．Use of heparin, polysaccharide sulfate or mannose ester within 3 weeks prior to screening  15．Upon determination by investigators, subjects can not complete this study  16． Subjects are investigators, staff at study center involving in this study, staff of GCP ClinPlus Co.，Ltd or Shanghai Greenvalley Pharmaceutical Co., Ltd. or their direct relatives. |
| **Drop-off Criteria** | 1．ICF withdrwal by either the subject or his/her guardian  2．Individual cases with unexpected unblinding in the trial  3．Lost to follow-up；  4．Allergic to any ingredient of Sodium oligomannate capsules  5．Subjects with unacceptable AE/SAE  6．Subjects with serious complications or deteriorated conditions who are in need of emergent intervention throughout the study.  7．Get pregnant during the trial  8．Other reasons. |
| **Withdrawal Criteria** | 1．Major violation of inclusion/exclusion criteria  2．Poor protocol compliance, use of prohibited medication during the study |
| **Termination Criteria** | 1. If serious safety issues occur throughout the study, the test should be terminated in time.  2. With poor efficacy, it is unnecessary to continue the trial.  3. Major errors of study protocol are found throughout the trial leading to un-evaluable efficacy or major deviation occurred in the operation leading to un-evaluable efficacy  4. Termination upon request by sponsor (due to funding and management reasons, etc.)  5. Cancelled by administrative regulators |
| **Concomitant**  **Medication** | The following drugs are prohibited througout the trial:  1．Adrenal cortical hormone, central stimulants, Chinese medicine supplements and various TCM and western medicines that can improve memory or cognition(hydergine, duxil, piracetam, Kangnaoshuai, aniracetam, etc.)  2．N-methyl-D-aspartate (NMDA) antagonists, e.g. amantadine, ketamine and dextromethorphan; antipsychotics (except risperidone, quetiapine and olanzapine stably used for more than 4 weeks before randomization); antidepressants (except sertraline, citalopram and escitalopram stably used for more than 30 days before randomization); sedative hypnotics (when necessary, zopiclone, alprazolam and estazolam can be temporarily used). For patients using the above antipsychotics for a long time, dose should be maintained stable throughout the study as far as possible.  3．Heparin, polysaccharide sulfate and mannose ester.  All used drugs rather than trial drug must be recorded in concomitant drugs form (including drug name, dose, frequency of administration and time), so as to evaluate possible influence on trial for analysis and reporting. |
| **Observation Endpoints** | **Primary Efficacy Endpoint：**  Improvement of cognitive function: Alzheimer’s Disease Assessment Scale – cognition (ADAS-cog/12-item).  **Secondary Efficacy Endpoints:**  1．Overall evaluation: Clinician's Interview-Based Impression of Change (CIBIC-plus scale)  2．Improvement of activities of daily living: Activity of Daily Living Scale (ADCS-ADL)  3．Improvement of neuropsychiatric behaviour: Neuropsychiatric Inventory (NPI)  **Subgroup Efficacy Endpoints:**  1．CSF biomarkers: Aβ40, Aβ42, T-tau, P-tau (For some patients who provide the written informed consent for CSF examination, lumbar puncture will be performed, and cerebrospinal fluid will be taken before and after treatment for drug detection ).  2． Glucose metabolism of bilateral temporoparietal cortex: observation by PET imaging (only in some centers)  3．Changes in the above endpoints of patients with different blood APOEε4 genotypes  **Observation Endpoints:** The concentration of Sodium oligomannate in blood and cerebrospinal fluid after reaching steady state (Week 24 after treatment) (patients with signed ICF of lumbar puncture). |
| **Safety Endpoints** | Incidence of adverse reactions, vital signs (blood pressure, heart rate), laboratory tests (hematology and urinalysis, biochemical indicators), ECG and premature withdrawal from the study. |
| **Number of visits** | There are totally 5 on-site visits and 3 phone visits. On-site visits occur in Week -4, Day 0 and Weeks 4, 12, and 24. Phone visits occur in Weeks 2, 8 and 16. |
| **Statistical analysis** | SAS statistical software will be used for all statistical analysis. Contents of analyses will be described in detail in statistical analysis plan (SAP), which will be reviewed at blind data review meeting and finalized before unblinding. All statistical tests will use two-sided test. P≤0.05 indicates statistically significant difference.  For description of quantitative variables, mean, standard deviation, median, minimum and maximum will be calculated; for description of categorical variables, number and percentage will be calculated. Changes from baseline will be calculated as (visit – baseline).  Multiplicity adjustment: verifying dose-response relationship of the primary endpoint is the main content of analysis in this study. Other further analyses are all exploratory. No multiplicity adjustment will be performed for class I errors. |
| **Study Timeline** | Estimated time of the first patients in (FPI): before Jul. 30^th^ ,2011  Estimated time of last patients in (LPI): before Jan. 30^th^, 2012 |

**A Multi-center, Randomized, Double-blind, Placebo parallel controlled, Dose-finding Phase II Clinical Trial to Evaluate the Efficacy and Safety of Sodium oligomannate capsules (971) in Treatment of Mild to Moderate Alzheimer’s Disease**

# 1. Study Background

Alzheimer disease (AD), one of the common diseases in the elderly population, is a degenerative disease of the nervous system. It is characterized by progressive memory loss and finally loss of life ability, dealing a great toll to families and the society. The main pathological features include extracellular senile plaques (SP), intracellular neurofibrillary tangle (NFT), and selective neuronal and synaptic loss. Currently, the etiology and pathogenesis are not yet clear, and thus there is no effective treatment.

With aging of the population, the incidence of AD has increased year by year. According to the 2009 report of the World Health Organization, there are about 35.6 million patients with AD worldwide, and it is expected to reach more than 115.4 million by 2050. In China, there are up to 7 million patients with AD and increase by 300,000 per year. It is predicted that there will be 12 million AD patients in China by 2030. In developed countries, AD has become the fourth leading cause of death after cardiovascular disease, cancer and cerebrovascular disease. The cost of treatment and care for AD is high, $11 billion a year in the UK and $83.9 billion in the United States. AD has become a serious social and health problem.

At present, drugs used in clinical treatment of AD mainly include cholinesterase inhibitors and N-methyl-D-aspartate (NMDA) receptor antagonists. Cholinesterase inhibitors mainly include donepezil, rivastigmine, galantamine and huperzine. These drugs can increase acetylcholine in synaptic cleft and are used in current first-line treatment of mild to moderate AD. Memantine is the representative of NMDA receptor antagonists. It acts on glutamine system in brain, improving glutamine delivery required in memory improvement. It is used in moderate to severe AD. However, all of these medications are symptomatic treatments, which can not effectively prevent the rapid development of AD, instead, have shortcomings such as large side effects and poor oral absorption. The anti-AD pipeline under development such as lipid-lowering drugs, anti-inflammatory drugs, hormonal substances and other antioxidants are difficult to achieve good application prospects due to the lack of specificity or challenge in penetrating the blood-brain barrier. Therefore, the development of anti-AD drug with special effects, low toxicity, easy to pass the blood-brain barrier is currently a hot spot of common concern in the medical community.

The main pathological manifestations of AD are as follows: ① A large number of extracellular inflammatory senile plaques, i.e. SP, with core and main components of β-amyloid (Aβ). ② NFT in the neurons. NFT is formed by highly phosphorylated tau protein. ③ large loss of cholinergic neurons and synapses.

The most important theory in the pathogenesis of AD is the amyloid cascade hypothesis, suggesting that the imbalance of Aβ production and clearance in the brain leads to disease. Since Masters et al. discovered that Aβ was the main structural substance of neurofibrillary tangles in 1985, there have been a lot of research on Aβ which plays a key role in the pathogenesis of AD. The deposition of Aβ is the originating factor and central link of AD pathology as well as the common pathway of AD development caused by various factors. Therefore, inhibition of Aβ production, promotion of Aβ clearance, reduction of toxicity and repair after neuronal damage have become important targets for AD drug development.

Sodium oligomannate is an oligosaccharide compound obtained by degradation of polymannuronic acid derived by degradation, separation and purification using alginic acid, extract of marine brown algae, as the raw material. It acts on Aβ pathway to treat Alzheimer’s disease. It is a new class 1.2 chemical drug.

State Food and Drug Administration new drug clinical trial approval No. is 2006L02492, 2011L00942.

# 2. Theoretical Basis

Aβ is a short peptide generated from its precursor protein amyloid precursor protein (APP) under effects of β secretase and γ secretase, with a molecular weight of approximately 4.2 KD, consisting of 39-43 amino acids. In physiological state, soluble Aβ can protect neurons. However, in pathological state, Aβ in α helix can be transformed to β-pleated sheet, which further links and aggregates to form insoluble Aβ aggregate. Aggregated Aβ deposits in nerve tissues, causing cytotoxicity, resulting in intracellular Ca^2+^ overload and increased free radical. These stimulate glial cells to release various inflammatory cytokines, damaging cholinergic signalling and reducing energy metabolism, causing cellular dysfunction and even death, finally manifested as dementia. Thus, inhibiting generation of Aβ and preventing formation of Aβ fibril are feasible ways to prevent development of AD. Aβ generation from APP through decomposition is a normal physiological process. Though inhibiting activity of β secretase and γ secretase may significantly reduce generation of Aβ, this will also influence other normal physiological functions of β secretase and γ secretase (e.g. γ secretase also plays a role in E-cadherin decomposition). Therefore, research and development of these drugs have certain limitations. It should be particularly noted that unlike effect on Aβ generation, as Aβ aggregation is an important pathological basis causing onset of AD and preventing Aβ aggregation will not influence normal physiological functions, design and development of anti-AD drugs by inhibiting various steps of Aβ fibril formation has become a hot spot.

Recent studies find endogenous polyanionic carbohydrates play an essential role in Aβ aggregation. Aβ molecule has heparin binding site (13-16 residues, HHQK). As a scaffold, endogenous polyanionic glycosaminoglycans (GAGs) can act on heparin binding sites of two adjacent Aβ molecules, promoting early formation of β-pleated sheet and advanced fibril aggregation of Aβ molecules, thus providing important “nucleation” basis for Aβ fibril formation, while stabilizing 3-D structure of Aβ fibril and preventing degradation of Aβ fibril. This suggests that any polyanionic compound competitively binding to heparin binding site on Aβ molecule may prevent binding between endogenous GAGs and Aβ, thus inhibiting formation of Aβ fibril. GAG has developed into an exciting anti-Aβ fibril aggregation.molecular template. Based on the fact that preventing Aβ fibril formation wil not influence normal physiological functions, finding saccharide anti-AD drugs that can bind to Aβ molecule, inhibit Aβ fibril formation, antagonize toxicity of Aβ fibril and easily pass blood-brain barrier has become the most promising direction in research and development of anti-AD drugs.

Sodium oligomannate is an oligosaccharide compound jointly developed by Shanghai Institute of Materia Medica of Chinese Academy of Medical Sciences and Ocean University of China. It is an oligosaccharide compound with high affinity to Aβ and specific molecular skeleton obtained through rational drug design, specific degradation technique and Aβ biochip screening based on structure-activity relationship between Aβ and carbohydrate. Through target SNK, HHQK heparin binding site, it competes with endogenous GAGs to bind to Aβ, inhibits transformation of Aβ from α helix to β-pleated sheet and promotes disaggregation of oligomerized and fibrillated Aβ, thus inhibiting toxicity of Aβ on neurons and exerting anti-AD effect. It has the potential to be an ideal therapeutic drug for AD.

**Results of pharmacological studies of Sodium oligomannate**  showed that Sodium oligomannate can significantly improve the learning and memory function of dementia animal models induced by intracerebral injection of Aβ, subcutaneous injection of D-Galactose and intraperitoneal injection of scopolamine and was characterized by high BBB peneration and low toxic side effects. Preliminary exploration on mechanism of action found that the effect of Sodium oligomannate on Alzheimer's disease is related to Aβ molecule binding, inhibition of Aβ fibril formation, promotion of Aβ fibril depolymerization and antagonism of Aβ fibril toxicity. In thise sense, Sodium oligomannate is a new drug with unique MOA in prevention and treatment of Alzheimer’s disease.

**The acute toxicity study of Sodium oligomannate**: the toxic symptoms after oral gavage and intraperitoneal administration of a single dose in mice were basically the same. The animals were slightly excited 10-15 minutes after dosing, mainly characterized by sensitive touch and jump. At 15-20 minutes after dosing, the animals gradually turned into a state of inhibition, mainly characterized by lying down, closed eyes, respiratory inhibition, gait instability, and purple mouth, etc. The number of animals with toxic reactions and the severity were positively correlated with the dose administered. The earliest death of oral gavage in mice occurred 2 hours after dosing, and the earliest death of intraperitoneal administration in mice occurred 1 hour after dosing, and all deaths occurred within 18 hours. The necropsy of the dead animals revealed bleeding duodenal and jejunal. The median lethal dose (Lethal Dose, 50', LD50) was calculated using the Bliss method. The LD50 values of Sodium oligomannate in mice was 21.1g/kg for oral gavage and 6.1g/kg for intraperitoneal injection. After oral gavage of Sodium oligomannate at 16g/kg in rats, reduced activities were observed at 90 min after dosing and Some (6/20) animals developed prone symptoms but returned to normal at 24 hours after dosing. Neither deaths nor significant effect on the weight gain were observed in rats during the 14-day observation period. The LD50 for oral gavage in rats was greater than 16 g/kg.

**The chronic toxicity study of** **Sodium oligomannate :** rats continuously received oral administration of Sodium oligomannate 120 mg/kg, 480 mg/kg and 1920 mg/kg for 180 days. Animals in each dose group were compared with control group. There was no abnormal change in mental status, behavior, activity, body weight or appetite. Histopathological examination showed no drug related lesion in vital organs in each group. Safe dose was 120 mg/kg. In 480 mg/kg and 1920 mg/kg dose groups, the animals had significant decrease in hemoglobin; in anatomical examination, adrenal gland, spleen and thyroid gland weight and coefficient in 480 mg/kg and 1920 mg/kg groups significantly increased compared with those in control group, and showed certain relationship with administered dose; after 30-day recovery following discontinuation, indicators in each dose group returned to normal compared with control group. In 120 mg/kg dose group, rats received oral administration for 180 days. There was no abnormal change in general observation, hematology, blood biochemistry, urine biochemistry, gross necropsy or histological indicators compared with control group. This dose was safe dose for oral administration in the 180-day chronic toxicity test in rats.

Beagles received continuous oral administration of Sodium oligomannate 60 mg/kg, 240 mg/kg and 960 mg/kg for 270 days. In 240 mg/kg and 960 mg/kg dose groups, the animals had slower eating speed and decreased food consumption after administration. In addition, there was soft stool, loose stool and positive stool occult blood. With prolonged administration time, stool abnormalities gradually disappeared. These changes were correlated with administered dose. In 960 mg/kg dose group, animals’ body weight was lower than that in control group. With prolonged administration time, body weight gradually returned to normal. In 960 mg/kg dose group, hematological examination found increased platelet (PLT) and decreased mean platelet volume (MPV); blood biochemical test showed increased cholesterol (CHO). Histopathological examination found some animals had unclear morphology of gall bladder mucosal epithelial cells, and local epithelial degeneration and shedding in 240 mg/kg and 960 mg/kg dose groups. These changes were essentially recovered after discontinuation for 30 days. After continuous oral administration of Sodium oligomannate for 270 days in beagles, there was no abnormal change in mental status, activity, anal temperature, body weight or urine in 60 mg/kg dose group;。there was no significant change in hematology, blood biochemistry or ECG examination compared with control group; this dose was no observed adverse effect level in beagles. hough Sodium oligomannate caused toxicity in animals, like other drugs, there was significant relationship with administered dose. Therefore, occurrence of toxicity can be controlled by controlling dose.

The dose of Sodium oligomannate leading to toxic reactions in rats and Beagle dogs was calculated as 9.1 times and 14.1 times of the recommended human dose (600 mg/person•day, 65 kg/person) , respectively, according to the body surface area. The safe dose in rats and Beagle dogs were 120 mg/kg and 60 mg/kg, respectively, which were 2.3 and 3.5 times of the equivalent dose in human (converted by body surface area).

**Genetic toxicity study of Sodium oligomannate** suggests Sodium oligomannate has no mutagenicity.

**Reproductive toxicity study of Sodium oligomannate:** in sensitive period of teratogenesis (gestational days 6-15), rats received oral gavage administration of Sodium oligomannate 120 mg/kg, 480 mg/kg and 1920 mg/kg/d. Negative control group was set. Necropsy was performed on gestational day 21. Growth of pregnant rats, and survival, development, appearance, bone and internal organs of fetus were examined to evaluate teratogenic toxicity of Sodium oligomannate . The results found in Sodium oligomannate 120 mg/kg, 480 mg/kg and 1920 mg/kg/d dose groups, weight gain of pregnant rats during administration was normal, without significant difference from control group; there was no significant difference in number of stillborn fetus, number of absorbed fetus, number of live fetus or loss rate after implantation between the three treatment groups and negative control group; there was no significant difference in mean body length, mean tail length or mean body weight of fetus between the three treatment groups and negative control group. In examination of fetal appearance, internal organs and skeletal system, no drug related teratogenesis or abnormal fetus was observed in Sodium oligomannate 120 mg/kg, 480 mg/kg or 1920 mg/kg/d dose group or negative control group. At 120 mg/kg, 480 mg/kg and 1920 mg/kg/d, Sodium oligomannate had no effect on growth of pregnant rats, no toxic effect on embryo, no significant effect on growth of fetus and no teratogenic effect on fetus. 1920 mg/kg/d can be considered as safe dose of Sodium oligomannate for reproductive toxicity in sensitive period of teratogenesis in rats.

Results of toxicity study indicate sensitive period of teratogenesis has low toxicity.

**Pharmacokinetic study:** Non-clinical pharmacokinetic study indicated after intravenous injection of Sodium oligomannate in rats, concentration-time curve was in line with three-compartment model, with T_1/2_(α) 0.80 h and T_1/2_(β) 6.84 h. After oral administration of Sodium oligomannate in rats, concentration-time curve was in line with two-compartment model, with T_1/2_(α) 1.25-1.83 h and T_1/2_(β) 8.25-11.85 h. Bioavailability was approximately 25-32%.

Tissue distribution study found that in 13 tested tissues and organs, except thymus, testis and adipose tissue where it was undetectable, it had high distribution in brain, spleen, heart, lung, stomach and intestine. High concentration was persistently maintained in brain, spleen, heart and lung. Certain concentration was maintained in kidney, liver and ovary. There was trace concentration in muscles.

Excretion study found that 72 h after oral administration of Sodium oligomannate 75 mg/kg in rats, dose excreted in urine was 24.56% and 61.93% in feces. Within 3 days, dose excreted in urine and feces was 86.48% of administered dose. Excretion was the highest within 1 day after administration, exceeding 75%. Peak excretion was achieved at 10-24 h, reaching 57.39%. At 5 μg/ml and 10 μg/ml, binding of Sodium oligomannate to rat plasma protein was about 50%.

Chemical method, ^3^H labelling method and immunological method were used to investigate penetration of Sodium oligomannate through blood-brain barrier and form of in vivo existence. The results found that Sodium oligomannate passed blood-brain barrier in unchanged form and existed in cerebrospinal fluid (CSF) as unchanged form. Additionally, brain microvascular endothelial cell glucose transporter-1 (GLUT-1) system was used to determine GLUT-1 was the main carrier protein mediating penetration of Sodium oligomannate through blood-brain barrier.

The above study results suggest Sodium oligomannate has established efficacy in improving learning and memory of dementia animals. Moreover, it has unique action mechanism, easily passes blood-brain barrier and has low toxicity. Therefore, it is a very promising drug to improve learning and memory in dementia.。

**Phase Ⅰ Clinical Results:**

（1）Sodium oligomannate capsules (971 capsules) are safe and well-tolerated in a single dose of 100-1200 mg and continuous administration at 900 mg/day and 1200 mg/day.

（2）Sodium oligomannate capsules (971 capsules) are taken orally, linearly absorbed in a single dose of 300 mg, 600 mg, 900 mg, and t1/2 is 9-11 hours.

（3）Food intake has no significant effect on the in vivo process of Sodium oligomannate capsules (971 capsules).

（4）Sodium oligomannate capsules (971 capsules) at 300 mg/day, 600 mg/day, twice a day, reach the steady state after continuous administration of 7 times and no significant accumulation in the body.

（5）There is no significant difference in the in vivo process of Sodium oligomannate capsules (971 capsules) between the healthy elderly population and young adults.

（6）Gender had no significant effect on the in vivo process of Sodium oligomannate capsules (971 capsules), indicating that Sodium oligomannate capsules (971 capsules) are safe and well-tolerated, has low toxic and side effects, and is easily absorbed by oral administration..

**Pharmacokinetic characteristics are：**

（1）After oral administration of Sodium oligomannate , plasma drug concentration was low, apparent volume of distribution was high and metabolism was slow. In 300 mg, 600 mg and 900 mg groups, mean elimination half life (t_1/2_) was 10.41±1.47 h, 11.00±3.32 h and 11.07±2.54 h, and time to maximum concentration (T_max_) was 0.73±0.13 h, 0.75±0.30 h and 0.78±0.38 h respectively.

（2）After single oral administration of 300 mg, 600 mg and 900 mg Sodium oligomannate in fasting state, maximum concentration (C_max_) and area under curve (AUC) increased with dose, showing linear pharmacokinetic characteristics.

（3）Comparing single oral administration of 300 mg Sodium oligomannate in subjects in fed and fasting state, there was no statistical difference in T_max_, C_max_, t_1/2_, mean residence time (MRT), AUC_0-48_ or AUC_0-∞_. This indicates food has no effect on in vivo process of Sodium oligomannate administered in healthy subjects.

（4）After steady state was achieved by multiple oral doses of Sodium oligomannate 300 mg and 600 mg in subjects, there was no change in t_1/2_, MRT or T_max_ compared with those after single oral administration of 300 mg and 600 mg; concerning ratio between AUC_ss_/dose and AUC_0-48_/dose, 300 mg (multiple)/300 mg (single) and 600 mg (multiple)/600 mg (single) ratios were 1.811 and 1.640 respectively, indicating multiple oral doses of Sodium oligomannate did not cause significant accumulation.

（5）Comparing pharmacokinetic parameters after single oral dose of Sodium oligomannate and multiple oral doses of Sodium oligomannate in male and female subjects, gender had no significant effect on in vivo process of Sodium oligomannate after oral administration in healthy subjects.

（6）After single oral dose of 300 mg and continuous oral doses of 300 mg Sodium oligomannate in healthy elderly subjects, t_1/2_, MRT, T_max_ and C_max_ were compared with those in young subjects. The results showed there was no significant difference in in vivo process of Sodium oligomannate after single-dose and multiple-dose oral administration between healthy young and elderly subjects.

（7）After steady state was achieved by oral administration of 300 mg Sodium oligomannate in healthy elderly subjects, ratio between AUC_ss_/dose and AUC_0-48_/dose showed 300 mg (multiple)/300 mg (single) ratio was 1.26, indicating multiple oral doses of Sodium oligomannate did not cause accumulation in elderly subjects.

# 3. Overview of Preclinical Study

## 3.1 Therapeutic Effect of Sodium oligomannate on Learning and Memory Function in Senile Dementia

Sodium oligomannate significantly improves learning and memory in APP/PS1 double transgenic dementia animals and dementia animal models caused by intracerebral injection of Aβ, subcutaneous injection of D-galactose and intraperitoneal injection of scopolamine. It significantly decreases latent period of water maze in animals with memory disorders, prolongs swimming time in the original platform quadrant, improves learning and memory, significantly reduces soluble and insoluble Aβ40 and Aβ42 in brain tissue of APP/PS1 double transgenic dementia animals, reduces Aβ plaques in brain tissue, and significantlly improves activity of choline acetyltransferase (ChAT), superoxide dismutase (SOD), glutathione peroxidase (GSH-PX) and adenosine triphosphatase (ATPase). It is suggested that Sodium oligomannate has a certain improvement effect on learning and memory impairment in senile dementia. At the same time, the protective effect of Sodium oligomannate on nerve cells damaged by Aβ was observed in vitro. It is found that Sodium oligomannate can inhibit the neurotoxicity of Aβ through multiple links.

### 3.1.1 Effect of Sodium oligomannate on Learning and Memory Function of Animals with Alzheimer's Disease Induced by Intracerebral Injection of Aβ_1-40_

Aβ1-40 aged for 72 hours was injected into the lateral ventricle of Balb/c mice to prepare mice with learning and memory dysfunction. The mice were subjected to step through test and Morris water maze behavior test to evaluate the effects of Sodium oligomannate at 15 mg/kg, 30mg/kg and 60mg/kg on learning and memory function of dementia animals. The results showed that Aβ_1-40_ lateral ventricle injection significantly prolonged the latency and swimming distance of the Morris water maze in mice, indicating that the mouse dementia model was successfully established. The oral gavage of Sodium oligomannate could significantly shorten the escape latency and swimming distance of the mice, and had quicker effect than the positive drug Huperzine A. The space exploration experiment showed that the lateral ventricle injection of Aβ_1-40_ significantly reduced the number of times the mouse passed through the original platform and prolonged the time to reach the original platform at the first time. For mice with treatment of Sodium oligomannate and huperzine A, the time to original platform for the first time were significantly shortened, and the number of crossing the original platform was significantly increased. The results of the step through test showed that the Aβ1-40 lateral ventricle injection significantly shortened the latency of the mice, and the number of errors increased significantly, suggesting that the mouse dementia model was successfully established. However, for mice with treatment of Sodium oligomannate and huperzine , the latency was prolonged and the number of errors was decreased significantly, indicating that Sodium oligomannate can significantly improve the learning and memory function of animals with Alzheimer's Disease induced by intracerebral injection of Aβ_1-40_.

### 3.1.2 Effect of Sodium oligomannate on Learning and Memory Function of Animals with Alzheimer's Disease Induced by Intracerebral Injection of Aβ_25-35_

Aβ_25-35_ aged for 72 hours was injected into the lateral ventricle of Balb/C mice to prepare mice with learning and memory dysfunction, and the Morris water maze behavior test was performed. Following the behavioral experiment, the mice were decapitated, the brain was taken, the cortex and hippocampus were separated, to determine the activity of ChAT, acetylcholinesterase (AchE), SOD, GSH-PX, monoamine oxidase B (MAO-B), Na+, K+ and the contents of malondial chehyche (MDA). The effects of mannooligosaccharide at 15 mg/kg, 30 mg/kg and 60 mg/kg on learning and memory function of dementia animals were evaluated. The results showed that injection of Aβ_25-35_ at lateral ventricle significantly prolonged the latency and swimming distance of the Morris water maze in mice, indicating that the mouse dementia model was successfully established. The dosing of Sodium oligomannate could significantly shorten the escape latency and swimming distance of the mice, and had quicker effect than the positive drug huperzine A. Exploratory experiments showed that Aβ25-35 lateral ventricle injection significantly reduced the percentage of time in the quadrant of the mouse, and the administration of Sodium oligomannate significantly increased the percentage of time in the quadrant of the original station. The results of biochemistry determination showed that Sodium oligomannate could significantly increase the activity of ChAT in the cerebral cortex of dementia mice, significantly increase the activity of SOD in the cerebral cortex and hippocampus, decrease the content of MDA, increase the activity of GSH-PX, and significantly increase the activity of Na+, K+-ATP enzyme in hippocampus. Meanwhile, it was found that Sodium oligomannate could significantly change the expression of cerebral cortex-related genes in mice with dementia induced by Aβ_25-35_ injection, indicating that Sodium oligomannate can significantly improve the learning and memory function of animals with Alzheimer's Disease induced by intracerebral injection of Aβ_25-35_.

### 3.1.3 Effect of Sodium oligomannate on Learning and Memory Function of Animals with Alzheimer's Disease Induced by Subcutaneous Injection of D-galactose

Male C57BL/6 mice were randomly assigned into the control group, the model group, Sodium oligomannate at 15, 30, 60 mg/kg group and positive control ligustrazine group and huperzine A group. Except the control group, animals were injected subcutaneously with D-galactose once a day for 6 weeks. Medication intervention was started from week 3 for 4 weeks. The Morris water maze test was started from week 5 for 3 days. After the Morris water-maze test, the animals were rested for one day, and the square labyrinth was started the next day for 3 days. Following the behavioral experiment, the mice were decapitated, the brain was taken, the cortex and hippocampus were separated, to determine the activity ofCuZn-SOD、GSH-PX、Na^+^K^+^-ATPase、AchE and ChAT and the content of malondial chehyche (MDA). Results of Morris water maze test showed that the escape latency of mice was significantly prolonged after injection of D-galactose, indicating that the aging model has been established, and the escape latency in the Sodium oligomannate group was significantly shorter than the model group, and the Sodium oligomannate group had quicker effect than the positive drug huperzine A. The square water maze test results showed that escape latency in the model group was significantly longer than the control group, indicating that the model was still established, and the latency in the Sodium oligomannate group was significantly shorter than that of the model group, indicating that Sodium oligomannate can significantly improve the learning and memory function of D-galactose-induced dementia animals. The results of biochemical indicators showed that the content of MDA in hippocampus and cortex of D-galactose aging model group increased significantly, CuZn-SOD, GSH-PX, AchE, ChAT active enzyme decreased significantly, Na+K+-ATPase activity increased significantly. Therefore, Sodium oligomannate can significantly reduce the content of MDA in hippocampus and cortex, increase the activity of CuZn-SOD, AchE and CHAT in cortex and inhibit the activity of Na+K+-ATP, indicating that the effect of Sodium oligomannate on improving memory function is related to reducing the formation and accumulation of lipid peroxidation products, improving brain energy metabolism and enhancing the function of acetylcholine system.

### 3.1.4 Effect of Sodium oligomannate on Learning and Memory Function of Animals with Alzheimer's Disease Induced by Intraperitoneal Injection of Scopolamine

Rats were randomly assigned into the control group, the model group, Sodium oligomannate at 12.5 mg/kg, 25 mg/kg, 50 mg/kg group and positive control huperzine A group. Except the control group and the model group were intragastrically administered with normal saline, the other groups were given corresponding drugs for 37 days. The Morris water maze behavioral test was started on day 31 and tested continuously for 5 days. The scopolamine at 0.75 mg/kg was intraperitoneally injected at 30 min before each test, once a day, and the rats were placed into the water from the two fixed points, and the time for searching for the platform (escape latency) and swimming distance were recorded within 2 min. After the end of the navigation test and at one-day interval, the platform was removed, and the rats were placed into the water from a water inlet point to measure the swimming time in the quadrant of the original platform within 2 minutes. The results showed that with the prolongation of training time, the escape latency and swimming distance of rats gradually shortened, and from the second day, compared with the normal group, the escape latency and swimming distance of the scopolamine model group were significantly prolonged. On Day 5 of training, compared with the model group, the Sodium oligomannate group and the huperzine A group had significantly shorter escape latency and swimming distance. The results of space exploration test showed that the swimming time of rats in the model group in the quadrant of the original platform was significantly shortened, while the swimming time of the Sodium oligomannate group and the positive drug huperzine A group in the quadrant of the original platform was significantly prolonged, indicating that Sodium oligomannate has a significant improvement effect on the learning and memory function of scopolamine-induced dementia animals. The results of biochemical test showed that Sodium oligomannate could significantly increase the activity of Na+, K+-ATPase and Ca2+-ATPase in hippocampus ,SOD and GSH –PX in rats with learning and memory impairment induced by scopolamin, indicating that the improvement of learning and memory function of Sodium oligomannate is related to antioxidation and improvement of brain energy metabolism.

### 3.1.5 Protective Effect of Sodium oligomannate on Nerve Cell Impairment induced by Aβ in vitro

Methylthiazoletetrazolium (MTT) was used to observe the effects of Sodium oligomannate on the damage of primary cortical neurons and neuroblastoma SH-SY5Y induced by Aβ_25-35_ and Aβ_1-40_. The results showed that Aβ_25-35_ and Aβ_1-40_ could significantly damage nerve cells, leading to reduced cells, shortened neurites, rounded cell bodies, and increase in suspended cells. However, when 10 μg/ml, 50 μg/ml, 100 μg/ml of Sodium oligomannate was added, neuronal damage induced by Aβ was significantly inhibited including increase in the number of cells , decrease in the number of suspended cells. The improvement effect incrased with the dose suggesting that Sodium oligomannate can significantly inhibit the neurocytotoxicity of Aβ. In addition, the effects of Sodium oligomannate on cytotoxic damage of Aβ at different stage (ie, different incubation time) were also observed. The results showed that Aβ with different degrees of aging had different damage to nerve cells. As the Aβ aging increased, the toxicity was stronger (Aβ_1-40_ at 12, 24, 48 hours of aging reduced cell viability to 84.2%, 73.5%, 68.7% respectively), However, if 100 μg/ml of Sodium oligomannate was added during the aging process, the cytotoxicity of Aβ was significantly reduced, suggesting that the anti-Aβ neurotoxicity of Sodium oligomannate may be related to the inhibition of fibril formation. In addition, the study also observed the survival of nerve cells under 3 senario, i.e. the treatment of Sodium oligomannate followed by Aβ induced damage, Sodium oligomannate and Aβ simultaneously added to cells, and Aβ damage followed by the treatment of Sodium oligomannate to determine the role of Sodium oligomannate in anti-Aβ neurotoxicity. The results showed that Aβ-induced damage to cells was different under these three treatments, but the MTT reduction ability of the Sodium oligomannate group was higher than that of the Aβ model group, suggesting that the Sodium oligomannate could inhibit Aβ neurotoxicity in mulitple links.

## 3.2 Study on the Mechanism of Sodium oligomannate to Improve the Learning and Memory Function of Alzheimer's Disease

At the cellular, molecular and genetic level, the mechanism of action of Sodium oligomannate was studied. It was found that Sodium oligomannate had obvious protective effects on nerve cell damage induced by Aβ and H2O2 by inhibiting the neuronal apoptosis and the increase in intracellular free calcium concentration and cellular lipid peroxidatio. It can sinificantly increase mitochondrial membrane potential, inhibit the contents of P53, caspase-3 and decrease the Bcl-2 protein expression, indicating that the mechanism of action of Sodium oligomannate is related to antagonizing Aβ toxicity, anti-oxidation and inhibition of apoptosis. Further studies revealed that the heparin-binding site His13-Lys16 of Aβ full-length molecule is the main site mediating interaction with sodium Sodium oligomannate , while Ser26-Lys28 is an important region for the binding of Aβ_25-35_ fragment to Sodium oligomannate which can inhibits Aβ conformational change, prevents fibril formation , promotes fibrillar depolymerization, and antagonizes Aβ neurocytotoxicity. Moreover, it was shown that the protective effect of Sodium oligomannate on SH-SY5Y cells injured by Aβ after 24 hours of PMA was abolished, suggesting that Sodium oligomannate antagonizing Aβ toxicity may be achieved through activation of protein kinase (Protein Kinase C, PKC). The results of Western blotting further confirm this inference. In addition, Sodium oligomannate can also up-regulate the content of mitogen-activated protein kinases (MAPK) in SH-SY5Y cells damaged by Aβ, and down-regulate expression of polysialic acid cell adhesion molecule (PSA-NCAM), indicating that Sodium oligomannate may antagonize the damage of SH-SY5Y cells induced by Aβ_25-35_ by activating PKC and further affecting MAPK activity. The mechanism of action of Sodium oligomannate on learning and memory function of dementia animals is related to its binding to Aβ molecule, inhibition of Aβ fibril formation, promotion of fibril depolymerization, antagonism of Aβ neurocytotoxicity and affecting cell signal transduction pathway.

Given the results of the pharmacodynamics, safety, and pharmacokinetic studies, the recommended indication for clinical trials is mild to moderate Alzheimer's disease. In order to explore the optimal dose, efficacy and safety of (971 capsules) in the treatment of mild to moderate Alzheimer's disease, a phase II clinical trial is proposed to implement.

Considering the presence or absence of the APOEε4 genotype may affect the efficacy of Sodium oligomannate capsules, all enrolled patients will receive the determination for APOEε4 genotype prior to the trial, and subgroup analysis will performed for APOEε4 genotype.

# 4. Study Objectives

## 4.1 Primary Objective

To explore the optimal therapeutic dose of Sodium oligomannate capsules (971 capsules) for the treatment of mild to moderate Alzheimer's disease and to evaluate its effectiveness and safety.

## 4.2 Secondary Objectives

(1) To evaluate the effects of Sodium oligomannate capsules on biomarkers (Aβ40, Aβ42, T-tau, P-tau) in cerebrospinal fluid.

(2) To determine the concentration of Sodium oligomannate in cerebrospinal fluid and blood at steady state.

# 5. Study Design

## 5.1 Study Rationale

（1）Drug clinical study approval document from China Food and Drug Administration (approval document No.: 2006L02492)

（2）Good Clinical Practice (GCP) 2003

（3）China Food and Drug Administration Technical Guideline on Clinical Trials of Drugs in Treatment of Alzheimer’s Disease 2007

（4）Drug Registration Management Regulations 2007

（5）Guideline of European Committee for Proprietary Medicinal Products to improve symptoms for anti-dementia trials

（6）Declaration of Helsinki 2000

（7）Data from preclinical pharmacodynamics, acute and chronic toxicology, phase I and phase II clinical studies of Sodium oligomannate (971)

## 5.2 Overview of Study Design

This drug is a class 1.2 chemical drug. According to Drug Registration Management Regulations, a multi-center, randomized, double-blind, placebo parallel controlled, dose-finding phase II clinical trial will be conducted.

252 eligible patients with mild to moderate AD will enter into the 4-week run-in period. For patients fulfilling the run-in period, they will receive a 24-week treatment. After double-blind randomization, patients will be assigned into the low-dose group (Sodium oligomannate capsules at 600 mg), or the high-dose group(Sodium oligomannate capsules at 900 mg) and the control group (Placebo). Each group will have 84 subjects and each center will compete for enrolment. For some patients with signed ICF of lumbar puncture, the biomarkers in cerebrospinal fluid will be observed and the concentrations in cerebrospinal fluid and blood at steady state will be determined.

## 5.3 Multi-center

This trial will be conducted at 20-30 study centers. Shanghai Mental Health Center, Affiliated to Shanghai Jiao Tong University School of Medicine is the leading unit for clincial study.

## 5.4 Randomization

This study will use interactive web response system (IWRS) to ensure dynamic stratified randomization. The stratification factors include study center, education level (6years vs. more than 6 years), with/without the CSF lumbar puncture, and with/without PET imaging examination.All the distributed study drugs have corresponding numbers. Doctors will log on IWRS system in the order of visit for each subject and distribute study drugs to designated subject according to drug number provided by the system. Doctors can not select drug number. IWRS system randomly distributes drugs for treatment period (24 weeks). Investigators should provide drugs to designated subject in several times according to the protocol. Each subject will receive one of the three treatments according to drug number. Treatment has to be initiated within 3 days after randomization.

## 5.5 Schedule of Events

| Events | Screening/  Run-in Period | Baseline ^1^ | 2 weeks after treatment | 4 weeks after treatment | 8 weeks after treatment | 12 weeks after treatment | 16 weeks after treatment | 24 weeks after treatment | Unscheduled visit |
| --- | --- | --- | --- | --- | --- | --- | --- | --- | --- |
| Visit | Visit 0  (Week -4) | Visit 1  ( D0 ) | PhoneVisit 1  (2weeks±3d) | Visit 2  (4weeks±3d) | PhoneVisit 2  (8weeks±7d) | Visit 3  (12weeks±7d) | PhoneVisit 3  (16weeks±7d) | Visit 4  (24weeks±7d) | Study required or premature withdrawal from trial |
| Written ICF | × |  |  |  |  |  |  |  |  |
| Inclusion/exclusion criteria | × | ×^2^ |  |  |  |  |  |  |  |
| Medical history | × |  |  |  |  |  |  |  |  |
| Physical examination | × | × |  |  |  |  |  | × | × |
| Vital signs | × | × |  | × |  | × |  | × | × |
| Urine pregnancy test ^3^ | × |  |  |  |  |  |  | × |  |
| Hematology and urinalysis | × |  |  | × |  | × |  | × | × |
| Blood biochemistry | × |  |  | × |  | × |  | × | × |
| Coagulation ^4^ | × |  |  | × |  | × |  | × | × |
| Folic acid, VitB12, thyroid function, syphilis test | × |  |  |  |  |  |  |  |  |
| Sample collection for APOE genotype determination | × |  |  |  |  |  |  |  |  |
| Cerebrospinal fluid sample retention (Aβ40, Aβ42, T-tau, P-tau, drug concentration detection) **^5^** |  | × |  |  |  |  |  | × |  |
| Determination of concention in blood ^6^ |  |  |  |  |  |  |  | × |  |
| ECG | × |  |  | × |  | × |  | × | × |
| Cranal MRI | × |  |  |  |  |  |  |  |  |
| PET imaging ^7^ |  | × |  |  |  |  |  | × |  |
| MMSE | × | × |  |  |  |  |  |  |  |
| HIS | × | × |  |  |  |  |  |  |  |
| HAMD | × | × |  |  |  |  |  |  |  |
| ADAS-Cog |  | × |  | × |  | × |  | × |  |
| CIBIC-plus |  | × |  | × |  | × |  | × |  |
| ADCS-ADL |  | × |  | × |  | × |  | × |  |
| NPI questionnaire |  | × |  | × |  | × |  | × |  |
| Concomitant medication | × | × | × | × | × | × | × | × | × |
| AE |  | × | × | × | × | × | × | × | × |
| Dispense/recover study drugs |  | × |  | × |  | × |  | × |  |
| Schedule visit time | × | × |  | × |  | × |  |  |  |

Note:1 Urine pregnancy test, hematology, urinalysis, biochemical examination, head MRI results at screening are acceptable as baseline result.

2 Inclusion/exclusion criteria verification is required at the end of run-in period.

3 Urine pregnancy tests are conducted in women capable of children bearing.

4 Coagulation: including calculation of prothrombin time, partial thromboplastin time, and international normalized ratio

5 Cerebrospinal fluid examinations will be performed in patients who signed ICF of lumbar puncture. Short-term hospitalization for observations are permitted. Cerebrospinal fluid biomarkers, APOE genotype testing, and drug concentration testing in cerebrospinal fluid will be performed at a central laboratory.

6 Determination of drug concentration in cerebrospinal fluid and blood will be performed to subjects who signed ICF of lumbar puncture at a central laboratory.

7 PET imaging will be conducted at some centers.

# 6. Selection of Subjects

## 6.1 Study Population

A total of 252 female/male patients with mild to moderate AD.

### 6.1.1 Diagnostic Criteria

#### 6.1.1.1 AD Diagnostic Criteria

Diagnostic criteria are those of probable AD according to National Institute of Neurological and Communicative Disorders and Stroke and the Alzheimer's disease and Related Disorders Association (NINCDS-ADRDA).

I Diagnostic criteria of probable AD:

（1）Dementia confirmed by clinical examination, dementia scale examination and neuropsychological test

（2）Cognitive deficits in at least 2 aspects

（3）Progressive deterioration of memory and other cognitive functions

（4）Without consciousness disorder

（5）Age of onset between 40 and 90 years, mostly after 65 years

（6）Without systemic or cerebral diseases that may induce progressive damage of memory and cognitive function.

Ⅱ After excluding other causes of dementia, clinical features supporting possible AD diagnosis include:

（1）Characteristic progressive deterioration of cognitive function, e.g. language (aphasia), motor skill (apraxia) and perceptual ability (agnosia)

（2）Damaged activities of daily living, change of behavior

（3）Family history of similar disease, especially pathologically confirmed family history

（4）Laboratory tests:

①Normal routine cerebrospinal fluid test

②Normal EEG or no specific change

③CT follow-up observation shows evidence of cerebral atrophy

### 6.1.2 Grading of AD severity

#### 6.1.2.1 Mild

Recent memory impairment is usually the initial and most significant symptom, e.g. frequent loss of objects, forgetting important appointment or promise and inability to recall name of a new colleague; difficulty in learning new knowledge and inability to remember contents after reading books or newspapers. There is often time disorientation in that the patient can not remember the specific date. Calculation ability is impaired. It is very difficult to complete simple calculations, e.g. continuous calculation of 100 minus 7 and minus another 7. Thinking is slow. There is difficulty in thinking, particularly inability to understand new things. Patients at early stage have certain insight in own memory problem and try to make up and mask, e.g. maintaining a record to avoid adverse effect of memory impairment on work and life, such as carefully managing money and preparing foods for family. Patients are able to complete familiar daily activities or usual works. Patients can essentially take care of themselves.

Personality change usually occurs at early stage. Patients are less active, have decreased activity, become lonely, selfish, less interested in surrounding environment, chilly to surrounding people, even indifferent to relatives, emotionally unstable, irritable and difficult to adapt to a new environment.

#### 6.1.2.2 Moderate

At this stage, patients can not live independently. Manifestation includes increasingly serious memory disorder. Patients may forget objects just used, lose household supplies and even valuables; forget things just occurring, forget own home address and name of relatives, but can still remember own name; sometimes may have falsification and fiction due to memory loss. Distant memory is also impaired. Patients can not remember own work experience and even forget own birthday. In addition to time disorientation, there is also place disorientation. Patients tend to get lost and even can not distinguish places, e.g. school or hospital. There is significant speech dysfunction. Patients have speech disorder and say meaningless words. They can not name similar objects. Subsequently, there is anomia. In naming test, patients can not name rare objects, followed by difficulty in naming common objects. There is agnosia, mostly face agnosia. Patients can not recognize relatives and friends, even own image in a mirror. There is apraxia. Patients can not correctly express by gesture or perform continuous movements, e.g. tooth brushing. Patients can not work or complete housework. Even basic daily activities such as washing and dressing require family’s supervision or assistance. Patients also have outstanding mental and behavior problems. Emotional fluctuation is unstable. Patients may be not able to find objects they have placed and suspect others of stealing, or suspect infidelity of spouse due to strong jealousy with fragments of illusion. There may be sleep disorder. Some patients are drowsy in daytime and have insomnia at night. There is behavior disorder. Patients often pick wastes and collect garbages; take others' belongings; there may also be excessive instinctive action, manifested as public nudity and sometimes aggressive behavior.

#### 6.1.2.3 Severe

Memory, thinking and other coginitive functions are all impaired. Patients forget own name and age and can not recognize relatives. Language ability is further deteriorated. Patients only have spontaneous speech with monotonous contents or repeately make incomprehensible sounds, and finally lose language function. Activities gradually decrease. Patients gradually lose walking ability and even can not stand up. They are finally bed ridden, with urinary and fecal incontinence. At late stage, patients may have primitive reflex.

The most significant neurological signs are increased muscle tension and body flexion. Disease course is progressive and usually lasts for 8-10 years. Spontaneous relief or cure is rare. Finally, it develops into severe dementia. Patients often die of secondary physical disease or failure, e.g. decubitus, bone fracture, pneumonia and malnutrition.

## 6.2 Inclusion Criteria

（1）Male and female subjects aged 50-85 years (inclusive)

（2）Education level: ≥ 6 years

（3）Meet diagnostic criteria of probable AD according to National Institute of Neurological and Communicative Disorders and Stroke and the Alzheimer's Disease and Related Disorders Association (NINCDS-ADRDA) (1984)

（4）Patients with mild to moderate disease, i.e. 10 points ≤ MMSE total score ≤24 points (see Appendix 1)

（5）Hachinski Ischemia Scale (HIS) total score ≤4 points (see Appendix 2)

（6）Hamilton Depression Scale/17-item (HAMD) total score ≤10 points (see Appendix 3)

（7）Subjects should have stable, reliable caregivers, or at least have frequent contact with caregivers (at least 4 days every week, at least 2 hours every day), and caregivers will help patients in participation in this study. Caregivers must accompany subjects to participate in study visits and have sufficient interaction and communication with subjects, so as to provide valuable information on scales including CIBIC-plus, ADCS-ADL and NPI

（8）The patient agrees to participate in the trial and provides the ICF by him/her or his/her guardian.

## 6.3 Exclusion Criteria

（1）Subjects with previous participation in any other clinical trial within 30 days prior to this study

（2）Pregnant and lactating women

（3）Paitents with allergic constitution

（4）Dementia due to other reasons: vascular dementia, central nervous system infection (e.g. AIDS, syphilis), Creutzfeldt-Jakob disease, Huntington's chorea, Parkinson’s disease, dementia with Lewy bodies, traumatic dementia, other physical and chemical factors (e.g. drug poisoning, alcoholism, carbon monoxide poisoning), significant physical illness (e.g. hepatic encephalopathy, pulmonary encephalopathy), intracranial occupying lesion (e.g. subdural hematoma, brain tumor), endocrine disorders (e.g. thyroid disease, parathyroid disease) and dementia caused by vitamin deficiency or other factors

（5）Previous nervous system disorders (including stroke, optic neuromyelitis, Parkinson’s disease, epilepsy)

（6）Abnormal laboratory indicators: liver function (ALT, AST) > 1.2× ULN, Cr > 1.2× ULN, WBC count＜4.0×10^9^, platelet＜100×10^9^, haemoglobin <100g/L, random blood glucose >1.2× ULN

（7）Uncontrolled hypertension: systolic blood pressure ≥160 mmHg or diastolic blood pressure ≥100 mmHg

（8）Unstable or severe cardiac, pulmonary, hepatic, renal or hematopoietic disease (including unstable angina, uncontrolled asthma, active gastric bleeding and cancer)

（9）Visual or hearing disorder to prevent completion of neuropsychological test and scale evaluation

（10）CT or MRI examination within 1 year prior to enrollment showed clinically significant focal lesion

（11）Alcohol or drug abuse

（12）Psychiatric patients, including those with severe depression

（13）Subjects with ongoing AD treatment which can not be discontinued

（14）Use of heparin, polysaccharide sulfate or mannose ester within 3 weeks prior to screening

（15）Upon determination by investigators, subjects can not complete this study

（16）Subjects are investigators, staff at study center involving in this study, staff of GCP ClinPlus Co.，Ltd or Shanghai Greenvalley Pharmaceutical Co., Ltd. or their direct relatives.

## 6.4 Uncompleted Cases

### 6.4.1 Drop-off Criteria

（1）ICF withdrwal by either the subject or his/her guardian

（2）Individual cases with unexpected unblinding in the trial

（3）Lost to follow-up

（4）Allergic to any ingredient of Sodium oligomannate capsules

（5）Subjects with unacceptable AE/SAE

（6）Subjects with serious complications or deteriorated conditions who are in need of emergent intervention throughout the study

（7）Get pregnant during the trial

（8）Other reasons.

Subjects have the right to withdraw from the trial at any time without discrimination or revenge. If a subject decides to withdraw from the trial, the investigator should obtain the reason as far as possible, and record the reason on case report form. Investigators may ask subjects to discountiue the trial in the following circumstances: there are adverse events or other administrative reasons, and investigators consider it is inappropriate to continue the clinical trial. When a subject prematurely withdraw from the trial, evaluation must be performed. If a subject withdraw from the the trial due to adverse event or abnormal laboratory value, these should also be recorded in case report form and end of trial page should be completed.

### 6.4.2 Withdrawal Criteria

（1）Major violation of inclusion/exclusion criteria

（2）Major violation of inclusion/exclusion criteria

At the blind review meeting, the principle investigator and statistician will decide whether the case is excluded. For excluded cases, the reason for exclusion should be given and their CRF should be kept for future review.

### 6.4.3 Termination Criteria

The termination of study refers to that the clinical study has not been completed according to the protocol, and all procedures have been stopped midway. The main objective is to protect the rights of subjects, ensure the quality of study, and avoid unnecessary economic losses.

（1）If serious safety issues occur throughout the study, the test should be terminated in time.

（2）With poor efficacy, it is unnecessary to continue the trial

（3）Major errors of study protocol are found throughout the trial leading to un-evaluable efficacy or major deviation occurred in the operation leading to un-evaluable efficacy

（4）Termination upon request by sponsor (due to funding and management reasons, etc.)

（5）Cancelled by administrative regulators.

# 7. Study Drug

## 7.1 Strength, Dosage Form and Manufacturer

Study drug: Sodium oligomannate capsules (971)

Dosage form：capsule

Strength: 150 mg/capsule

Manufacturer: Shanghai Greenvalley Pharmaceutical Co., Ltd

Shelf life: 2 years

Storage condition: room temperature, protected against light, dry, sealed

Run-in period drug and reference drug: placebo without any active component, provided by Shanghai Greenvalley Pharmaceutical Co., Ltd. Strength: 150 mg/capsule; shelf life: 2 years.

Placebo is identical to Sodium oligomannate capsule in taste, odor, appearance and design, and acceptable in quality standard inspection approved by China Food and Drug Administration. Drug production date and shelf life are provided at time of use. Please check carefully.

The above study drugs are all manufactured in plants meeting GMP conditions and obtain drug test certificate.

## 7.2 Drug Package

Before initiation of the clinical trial, all study drugs are packaged in the same way. Sodium oligomannate capsules are packaged in bottles. Each bottle contains 42 capsules (spare drugs include 18 or 42 capsules per bottle). Outer package of study drugs contains the following contents: clinical study batch No., number of contained drugs, drug strength, storage condition, administration route, shelf life, drug supplier and “Only for clinical study”.

## 7.3 Dosage and Administration

This study includes 3 dose groups, i.e. the low-dose group (Sodium oligomannate at 600mg), the high-dose group (Sodium oligomannate at 900mg ) and the placebo group. The study duration includes a 4-week screening/run-in period and a 24-week treatment period.

### 7.3.1 Drug in the run-in period

Placebo, oral, b.i.d, 3 capsules once a time.

### 7.3.2 Treatment Period

The low-dose group (600 mg): for each dose, 2 Sodium oligomannate capsules(971 capsules)+ 1 placebo

The high-dose group (900 mg): for each dose, 3 Sodium oligomannate capsules(971 capsules)

The placebo group: for each dose, 3 placebo capsules.

For each dose group, the dose regimen is twice a day in the morning and evening for 24 weeks.

## 7.4 Management and Storage of Study Drugs

Study drugs have to be stored in a safe, dry place at room temperature, protected against light. There should be a specifically designated person responsible for drug management in each study center. Only investigators, personnel designated by investigators and clinical monitors can have access to drugs.。

Drugs can only be used for eligible patients enrolled in this clinical study. Eligible enrolled patients receive Study drugs strictly according to principle of randomization. In drug distribution, the pharmacist or clinician has to carefully check enrollment number and ensure to distribute drugs according to the designated number. Clinical trial monitors will regularly retrieve remaining drugs and drug packages. Investigators should receive, store, distribute and retrieve drugs according to relevant regulations, and maintain corresponding record. Investigators must return unused drugs to the sponsor. Study centers are obligated to maintain a drug list for drug monitoring, audit and inspection by clinical monitors, auditors and CFDA.

## 7.5 Drug Distribution and Record

Study drugs will be directly delivered to study centers by the sponsor. According to drug number generated by interactive web response system (IWRS), trial drug managers in study centers will distribute drugs with corresponding number to each clinical investigator, who will distribute drugs to subjects.

After subjects are eligible in screening follow-up, investigators will log on IWRS system to obtain randomization number and drug number for each subject. In 36-week treatment period, subjects will be randomized to each trial group at 1:1 ratio. In each visit, investigators should record reception and return of study drugs, and accurately complete drug distribution registration form in time.

Investigators are responsible for distribution, inventory and tracking of all clinical drugs, and record distribution and counting of all clinical drugs in time. After the study is terminated, investigators have to hand all remaining drugs and inventory record to monitors.

Study drugs can not be distributed to patients rather than subjects.

Investigators should record each batch of received drugs and drugs distributed to each patient in drug storage form.

## 7.6 Evaluation of Drug Compliance

Investigators should emphasize importance of compliance to subjects.

In return visits, subjects have to return packages of used drugs, e.g. package bottle, and unused drugs and packages. Investigators or designated personnel will count number of returned drugs, calculate number of used drugs and compare with number of drugs that should be taken. Lost and unreturned drugs will be recorded.

Drug compliance = number of actually used drugs/number of drugs that should be used×100%.

criterion of good drug compliance: 80%≤compliance≤120%; poor compliance: compliance <80% or >120%.

If subjects have poor compliance, reasons should be found and recorded.

Investigators will record number of distributed and returned drugs in corresponding part of case report form.

## 7.7 Drug Accountability

Investigators are responsible to count, check and record study drugs. Investigators or designated personnel have to maintain a record of drug number throughout the study. Clinical study monitors will regularly check stored drugs, returned drugs and distribution record form in study centers. At the end of study, all remaining drugs, returned drugs and packages will be counted by investigators and clinical monitors, and then retrieved by the sponsor.

## 7.8 Concomitant Medication

For other concomitant diseases, symptomatic drugs can be used as long as trial drug efficacy evaluation is not influenced. Except etiological treatment, concomitant drugs should be minimized.

### 7.8.1 Prohibited Drugs Throughout the Study

If subjects receive any of the listed “prohibited drugs” during the study, they should quit the study. However, if treatment duration is <7 days, investigators can immediately stop the prohibited drugs according to subjects’ safety and necessity of new treatment. In that case, subjects may continue the study. Use of prohibited drugs must be recorded in the section of “concomitant drugs” in CRF.

**Table 1 Prohibited Drugs**1

| 1. Taking medication now or within the last 30 days or 5 half-life periods (whichever is longer) | |
| --- | --- |
| 1a Within 30 days before Visit 1 or 5 half-lives (whichever is longer).  2a Pro re nata (PRN), defined to be use as needed in the past 8 weeks, less than 4 times weekly (more frequent administration is defined to be chronic short-term or long-term administration).  Long-term administration is defined as chronic administration ≥2 months at fixed dose before Visit 1. | |
| Category of prohibited drugs | Description |
| Adrenal cortical hormone ^1a^ |  |
| Central stimulants ^1a^ |  |
| 中成药补剂^1a^ |  |
| Various Chinese and western nootropics influencing memory ^1a^ | e.g. hydergine, duxil, piracetam, Kangnaoshuai, aniracetam, etc. |
| N-methyl-D-aspartate (NMDA) inhibitors^1a^ | e.g. memantine, amantadine, ketamine and dextromethorphan |
| Antipsychotics | Except risperidone, quetiapine and olanzapine which have been stably used before randomization and dose is maintained stable during the study. |
| Antidepressants | Except sertraline, citalopram and escitalopram which have been stably used for more than 4 weeks before randomization and dose is maintained stable during the study |
| Sedative hypnotics ^2a^ | Sedative hypnotics (zopiclone, alprazolam and estazolam can be temporarily used when necessary). For patients receiving sedative hypnotics for a long time, dose should have been stable for at least 4 weeks before randomization and is maintained stable as far as possible during the study. |
| Heparin, polysaccharide sulfate and mannose ester |  |

### 7.8.2 Permitted Drugs in the Trial

All medication other than prohibited drugs in Table 1. If drugs which have a negative impact on cognitive function are used such as anti-cholinergic drugs (including drugs with significant anticholinergic activity, e.g. amitriptyline) and most sedatives( typical or atypical antipsychotics), dose should be stable for at least 4 weeks before baseline and maintained unchanged as far as possible during the study。

# 8. Study Evaluations and Procedures

## 8.1 Screening/Run-in Evaluation——Visit 0（Week-4）

Investigators have to record eligibility of each patient against inclusion criteria and whether he/she is finally included or excluded in screening form and submit to the sponsor. After all patients are eligible in screening, 4-week single-blind, placebo run-in period will be entered. In screening/run-in evaluation, the following evaluations or procedures should be completed：

（1）Sign ICF prior to the screening

（2）Fill in general information

（3）Inquire of medical history, including previous history, present illness, treatment history and history of other concomitant diseases and medcation

（4）Physical examination, vital signes (body temperature, resting heart rate, respiratory rate and blood pressure after resting for 10 minutes)

（5）Cranial magnetic resonance image(MRI）examination（能提供筛选前一年内结果的可免做）；

（6）Scale evaluation: including Hachinski ischemia scale (HIS), HAMD, and MMSE

（7）Laboratory tests ( results within 7 days prior to the screening are acceptablemed in screening period)

① Haematology : red blood cell (RBC), hemoglobin (HGB), white blood cell (WBC), percentage of neutrophil, percentage of lymphocyte, percentage of monocyte, percentage of eosinophil, percentage of basophil and platelet (PLT)；

② Urinalysis: Urine pH (pH), urine protein, leukocyte (LEU) and red blood cell；

③ Blood biochemistry: alanine transaminase (ALT), glutamic-oxalacetic transaminase (AST), total bilirubin (TBiL), direct bilirubin (DBiL), alkaline phosphatase (ALP), serum albumin (ALB), blood urea nitrogen (BUN), creatinine (Cr), gamma-glutamyl transferase (GGT), and blood glucose；

④ Coagulation: including calculation of prothrombin time, partial thromboplastin time, and international normalized ratio

⑤ Folic acid, VB_12_, thyroid function (T3, T4, FT3, FT4 and TSH), rapid plasma reagin circle card test (RPR)

⑥ Blood sample collection: determinnation of APOE genotype

⑦ Urine pregnancy test (women of child bearing potential)

（8）ECG examination (results within 7 days prior to the screening are acceptable.)

（9）Record adverse events

（10）Record drug compliance

（11）Dispense run-in drugs and inform patients of administration method: 3 capsules each time, twice daily, oral

（12）Schedule the next visit

## 8.2 Baseline Evaluation – Visit 1 (Day 0)

After the end of run-in period, investigators will evaluate patients’ compliance. For patients with good compliance (placebo administration complaince is 80%-120%), evaluation against inclusion/exclusion criteria is performed again. Eligible patients will enter randomization and the study drug will be dispensed. The efficacy data of baseline assessment should be obtained within 3 days (72 hours) prior to the first dosing. For Laboratory tests (hematology, urinalysis, blood biochemistry, coagulation and urine pregnancy test), ECG and cranial MRI, screening results are acceptable. The following assessments or procedures should be completed at baseline:

（1）Review inclusion/exclusion criteria and evaluate run-in period compliance；

（2）Vital signs: body temperature, heart rate, respiratory rate and blood pressure after resting for 10 minutes

（3）Physical examination

（4）Scale assessment: including HIS, HAMD, MMSE, Alzheimer’s Disease Assessment Scale – cognition (ADAS-Cog/12), Alzheimer’s Disease Collaborative Study – Activity of Daily Living Scale (ADCS-ADL), Neuropsychiatric Inventory – Clinician Rating Scale (NPI) and Clinician's Interview-Based Impression of Change (CIBIC-plus)

（5）Cerebrospinal fluid sample collection: Aβ40, Aβ42, T-tau, P-tau. For subjects with lumba puncture, short-term hospitalization observations can be scheduled depending on the situation

（6）PET imaging examination: glucose metabolism of bilateral temporoparietal cortex (only in some centers)

（7）Record adverse events

（8）Record concomitant medication

（9）Dispense run-in drugs and inform patients of administration method: 3 capsules each time, twice daily, oral

（10）Schedule the next visit

## 8.3 Week-4 after Treatment – Visit 2 (4 weeks±3 days)

（1）Vital signs: body temperature, resting heart rate, respiratory rate and blood pressure after resting for 10 minutes

（2）Scale evaluation: including ADAS-Cog scale, CIBIC-plus scale, ADCS-ADL scale and NPI questionnaire；

（3）Laboratory tests:

① Hematology: red blood cell (RBC), hemoglobin (HGB), white blood cell (WBC), percentage of neutrophil, percentage of lymphocyte, percentage of monocyte, percentage of eosinophil, percentage of basophil and platelet (PLT)

② Urinalysis: urine pH, urine protein, leukocyte (LEU) and red blood cell；

③ Blood biochemistry: alanine transaminase (ALT), glutamic-oxalacetic transaminase (AST), total bilirubin (TBiL), direct bilirubin (DBiL), alkaline phosphatase (ALP), serum albumin (ALB), blood urea nitrogen (BUN), creatinine (Cr), gamma-glutamyl transferase (GGT), and blood glucose

④ Coagulation: including calculation of prothrombin time, partial thromboplastin time, and international normalized ratio

（4）ECG

（5）Record adverse events

（6）Record concomitant medication

（7）Dispense/recover run-in drugs and inform patients of administration method: 3 capsules each time, twice daily, oral

（8）Schedule the next visit

## 8.4 Week-12 after Treatment – Visit 3 (12 weeks±7 days)

（1）Vital signs: body temperature, heart rate, respiratory rate and blood pressure after resting for 10 minutes

（2）Scale assessment: including ADAS-Cog, ADCS-ADL, NPI and CIBIC-plus

（3）Laboratory tests：

①Haematology : red blood cell (RBC), hemoglobin (HGB), white blood cell (WBC), percentage of neutrophil, percentage of lymphocyte, percentage of monocyte, percentage of eosinophil, percentage of basophil and platelet (PLT)

② Urinalysis: Urine pH (pH), urine protein, leukocyte (LEU) and red blood cell

③ Blood biochemistry: alanine transaminase (ALT), glutamic-oxalacetic transaminase (AST), total bilirubin (TBiL), direct bilirubin (DBiL), alkaline phosphatase (ALP), serum albumin (ALB), blood urea nitrogen (BUN), creatinine (Cr), gamma-glutamyl transferase (GGT), and blood glucose

④ Coagulation: including calculation of prothrombin time, partial thromboplastin time, and international normalized ratio

（4）ECG

（5）Record adverse events

（6）Record concomitant mediction

（7）Dispense/recover run-in drugs and inform patients of administration method: 3 capsules each time, twice daily, oral

## 8.5 Week-24 after Treatment – Visit 4 (24 weeks±7 days)

（1）Vital signs: body temperature, heart rate, respiratory rate and blood pressure after resting for 10 minutes

（2）Scale assessment: including ADAS-Cog, ADCS-ADL, NPI and CIBIC-plus

（3）Laboratory tests:

① Haematology : red blood cell (RBC), hemoglobin (HGB), white blood cell (WBC), percentage of neutrophil, percentage of lymphocyte, percentage of monocyte, percentage of eosinophil, percentage of basophil and platelet (PLT)

② Urinalysis: Urine pH (pH), urine protein, leukocyte (LEU) and red blood cell

③ Blood biochemistry: alanine transaminase (ALT), glutamic-oxalacetic transaminase (AST), total bilirubin (TBiL), direct bilirubin (DBiL), alkaline phosphatase (ALP), serum albumin (ALB), blood urea nitrogen (BUN), creatinine (Cr), gamma-glutamyl transferase (GGT), and blood glucose

④ Coagulation: including calculation of prothrombin time, partial thromboplastin time, and international normalized ratio

（4）Cerebrospinal fluid sample collection: determination of Aβ40, Aβ42, T-tau, P-tau, and concentration of Sodium oligomannate (for subjects with signed ICF of lumbar puncture ) . For subjects with lumbar puncture, short-term hospitalization observations can be scheduled depending on the situation

（5）Blood sample collection: determination of concentration of Sodium oligomannate in blood (for subjects with signed ICF of lumbar puncture)

（6） PET imaging examination: glucose metabolism of bilateral temporoparietal cortex (only in some centers)

（7）ECG

（8）Record adverse events

（9）Record concomitant medication

（10）Recover the study drug

## 8.6 Phone Follow-up

At weeks 2, 8 and 16 after treatment, the subjects will be followed up by telephone to obtain information about adverse events, mental state, diet, urination, defecation, dry mouth and concomitant medication.

## 8.7 Unscheduled Visits (study required or premature withdrawal from the trial)

If subjects require additional visits or prematurely withdraw from the trial, investigators should record measures taken (including results of laboratory tests) on original medical record and unscheduled visit page of case report form。

In addition, when subjects prematurely withdraw from the trial before end of trial, safety evaluation should be performed as far as possible and investigators should complete end of trial page.

## 8.8 Relevant Treatments after the End of Trial

Upon the end of study, if the subject is willing to continue taking the study drug, the sponsor can provide the study drug free of charge for 6 months, but will not continue to pay the cost of examination. Subjects may also choose the currently available anti-AD treatments according to physician’s advice, such as cholinesterase inhibitors (donepezil, rivastigmine, galantamine, and huperzine A) or N-methyl-D-aspartate (NMDA) receptor antagonist (Memantine).

## 8.9 Central Laboratory Tests

（1）CSF samples: It will be used for the determination of drug concentration and biomarkers (Aβ40, Aβ42, T-tau, P-tau). For subjects with signed ICF of lumbar puncture, the CSF samples will be collected at Visit 1 (baseline) and Visit 4 (Week 24). Lumbar puncture will be performed using the routine method. A 3 ml of CSF sample will be collected and stored in the sterile polypropylene tube and placed on the ice. Following that, the sample will be centrifuged at 4000 rpm for 10 min. The supernatant is dispensed in 0.5 ml/tube, frozen and stored in a -80 degree refrigerator for testing, avoiding repeated freezing and thawing. The time from collecting samples to storing in the refrigerator should be within 2 hours.

（2）Blood samples: It will be used for the determination of APOE genotypes. Ater the blood is collected, it will be anticoagulated with ethylenediaminetetraacetic acid (EDTA) or ACD and dispensed to 2.5 ml/tube (at least 4 tubes for each patient at each collection). Following that , the sample will be centrifuged at 3000 rpm for 10 min, and divided into supernatant (plasma) and sediment (cell). The supernatant will be taken separately to another tube, and both the supernatant and the cell should be frozen in an ultra-low temperature freezer (-80 degrees). The samples for APOE genotype determination will be collected for all subjects at Visit 0 (screening/run-in period) while those for drug concentration determination will be collected for subjects with lumbar puncture at Visit 4 (Week 24).

After a certain amount of sample is collected, samples will be transported using the cold-chain logistics to the central laboratory for uniform determination.

## 8.10 PET Imaging Examination

PET imaging will be performed only in some centers. A total of 30 subjects (10 subjects/group) will receive the PET imaging examination. The examination results will be assessed in uniform by professionals to observe the glucose metabolism in the bilateral temporoparietal cortex.

## 8.11 Safety Assessment

Safety evaluation will be performed at baseline and after treatment. In premature withdrawal, safety evaluation should be performed before withdrawal. For clinically significant laboratory abnormalities, determination should be repeated within 1 week as far as possible. Results are recorded in the section of “unscheduled visit” in CRF. Necessary clinical treatment will be provided till recovery or stable.

Safety evaluation includes adverse events, vital signs, laboratory examination and premature withdrawal due to safety or tolerability issues. All adverse events and corresponding treatments should be recorded in CRF.

# 9. Endponints

## 9.1 Efficacy Endpoints

### 9.1.1 Primary Efficacy Endpoint

Improvement of cognitive function: Alzheimer’s Disease Assessment Scale – cognition (ADAS-cog/12-item)

### 9.1.2 Secondary Efficacy Endpoints

（1）Overall evaluation: Clinician's Interview-Based Impression of Change (CIBIC-plus scale)

（2）Improvement of activities of daily living: Alzheimer’s Disease Collaborative Study – Activity of Daily Living Scale (ADCS-ADL)

（3）Improvement of neuropsychiatric behavior: Neuropsychiatric Inventory (NPI)

### 9.1.3 Subgroup Efficacy Endpoints

（1）CSF biomarkers: Aβ40, Aβ42, T-tau, P-tau (For some patients who provide the written informed consent for CSF examination, lumbar puncture will be performed, and cerebrospinal fluid will be taken before and after treatment for detection)

（2）Glucose metabolism of bilateral temporoparietal cortex: observation by PET imaging (only in some centers)

（3）Changes in the above endpoints of patients with different blood APOE genotypes.

### 9.1.4 Efficacy Evaluation Scales

#### 9.1.4.1 ADAS-Cog

ADAS-Cog/12-item is the primary efficacy endpoint. It is a tool for comprehensive cognitive measurement. ADAS-Cog evaluation of cognitive functions includes 12 items, e.g. memory, understanding, orientation, language, structure, use of concept, immediate words recall and words recognition. It takes about 30-40 min. Score range is 0-75. Generally, 4 points of improvement in ADAS-Cog/12 is considered as clinically sigificant for antidementia drugs.

The assessment of ADAS-Cog/12 is scheduled on visits 1 – 4 and the scale is detailed in Appendix 4.

#### 9.1.4.2 CIBIC-plus

Clinician interview based impression change is a tool mainly used to evaluate overall change of AD patients, including symptoms in 3 aspects: cognition, function and behavior. Behavior part of CIBIC-plus uses corrected version of Behavioural Pathology in Alzheimer Disease Rating Scale (BEHAVE-AD). In caregiver questionnaire, severity of behavior symptoms in 7 main fields and severity of 25 specific symptoms (or items) in corresponding fields are evaluated.

The assessment of CIBIC-plus is scheduled on visits 1 – 4 and the scale is detailed in Appendix 5.

#### 9.1.4.3 ADCS-ADL

ADCS-ADL is a secondary efficacy endpoint evaluating activities of daily living. It includes 23 items confirming effectiveness step by step. Total score is 78. Higher score indicates better ability of daily living and lower score indicates poorer ability of daily living.

The assessment of ADCS-ADL is scheduled on visits 1 – 4 and the scale is detailed in Appendix 6.

#### 9.1.4.4 NPI

NPI is a secondary efficacy endpoint, evaluating 14 common mental behavior symptoms in dementia. Each subitem contains screening questions reflecting core symptoms. If answer to a screening question is “No”, the patient proceeds to the next screening question. If answer is “Yes”, severity of symptoms in the past 4 weeks and degree of caregiver distress should be evaluated and the clinician will evaluate clinical impression. Change in clinical impression score in each field of NPI can be used to compare drug efficacy.

The assessment of NPI is scheduled on visits 1 – 4 and the scale is detailed in Appendix 7.

## 9.2 Observation of Drug Concentration

The concentration of Sodium oligomannate in cerebrospinal fluid and blood at steady state (Week-24).

## 9.3 Safety Evaluation

Safety evaluation includes vital signs, adverse events, laboratory tests and physical examination. The follow-up of subjects will be conducted by investigators as per the schedule including the vital signs, clinical presentations, and laboratory tests. The quality control certificate of each laboratory and the normal value range of the test items shall be provided to the monitor. For abnomal laboratory test values that are difficult to interpret, the re-testing should be peformed immediately until normal and/or well-explained.

All adverse events occurring during drug treatment will be recorded in CRF. Evaluation of adverse events includes classification, grade, relationship with the drug, actions taken and outcome.

### 9.3.1 Adverse Event (AE)

#### 9.3.1.1 Definition of AE

An adverse event refers to an untoward adverse medical event occurring in a subject after administration of study drug, not necessarily related to the drug, including any new event or event with worsening in severity and frequency from baseline, including laboratory abnormalities.

Adverse events that occurred during the trial must be recorded on the case report form (CRF). For treatment emergent adverse events (TEAE), information such as symptoms, severity, onset time, duration, actions taken, and duration should be recorded in the observation form and the realtionship with the study durg should be assessed on the comprehensive consideration of comorbidities and concomitant medication by the investigator and relevant records should be made accordingly. Adverse events should be coded using the medical terms and the disease diagnosis should be given as much as possible instead of listing symptoms and signs (e.g. the symptoms of cough, runny, sneezing and sore throat should be reported as upper respiratory tract infection).

#### 9.3.1.2 Relationship with the Study Drug

The investigators should assess the association between adverse events and the study drug and concomitant medications. The relationship between adverse events and the treatment can be classified accoording to the following 5-level criteria.

**Definitely related:** follows a reasonable temporal sequence from the time of drug administration, corresponds with a known response pattern to the investigational product, improvement on discontinuation or dose reduction, reappearance on rechallenge.

**Probably related:** follows a reasonable temporal sequence from the time of drug administration, corresponds with a known response pattern to the investigational product, improvement or disappearance on dose reduction or withdrawal, however, the subject's clinical status or other factors may also cause this response；

**Possibly related:** follows a reasonable temporal sequence from the time of drug administration, corresponds with a known response pattern to the investigational product, however, the subject's clinical status or other factors may also explain this response.

**Unlikely related:** without a reasonable temporal sequence from the time of drug administration , not consistent with a known response pattern to the investigational product, the subject's clinical status or other factors may also cause this response

**Not related:** without a reasonable temporal sequence from the time of drug administration, consistent with a known response pattern of medication other than the investigational product, the subject's clinical status or other factors may also cause this response, improvement or disappearance after excluding clinical symptoms or other causes, reappearance after recovery of other treatments.

#### 9.3.1.3 Severity Grading of AE

(1) Mild: mild adverse reaction, without the development of symptoms, with slight discomfort, disppearance without the treatment and withdrawal.

(2)Moderation: obvious adverse reactions, with significant discomfort and moderate damage of important organs or systems

(3)Severe: serious damage to vital organs or systems, life-threatening.

### 9.3.2 Adverse Drug Reactions (ADR)

An adverse drug reaction (ADR) is an unwanted, undesirable effect of a medication that occurs during usual clinical use. In a clinical trial of a new drug or new drug use, when the therapeutic dose has not been determined, all harmful, rather than expected, causal reactions with the drug application should also be considered adverse drug reactions.

### 9.3.3 Clinical Laboratory Abnormalities

When laboratory abnormalities are not accompanied by clinical signs and symptoms, they are not recorded as adverse events, and such changes will be considered in data analysis. However, if the investigator believes that the necessary treatment should be given for the laboratory abnormalities, such laboratory abnormalities should be reported as adverse events and recorded together with concomitant treatment.

For abnomal laboratory test values that are difficult to interpret, the re-testing should be peformed immediately until normal and/or well-explained.

### 9.3.4 Serious Adverse Event (SAE)

Any unfavorable medical event should be defined as a SAE if it met one or more description stated below:

（1）Results in death

（2）Teratogenic, carcinogenic or congenital defects

（3）life-threatening and results in persistent or significant disability

（4）Permanent damage to organ function

（5）Requires hospitalization or prolongation of existing hospitalization

If the subject experiences a serious adverse event during the trial, regardless of whether it is related to the study drug, the investigator should immediately take appropriate treatment measures to protect the subject's safety and report to the Medical Ethics Committee of the study center and the Drug Registration Department of the State Food and Drug Administration (Tel: 010-68313344, Fax: 010-88363228), health administrative department and clinical study project manager (Yue Chen, mobile: 18911101026, Tel: 010-51298908-200, Fax: 010-68716972), contact of the sponsor ( Hai-yin Piao, mobile: 13811299202, Tel: 010-88217796), the PI (Shi-fu Xiao, mobile: 13818246156) and the contact of GCP center (Hua-fang Li, mobile: 13641625395, Tel: 021-64387250-3128) within 24 hours after the event occurs. Meanwhile, the SAE report should be completed in time and the relevant information should be recorded in detail as far as possible.

Cerebrospinal fluid will be collected at either the out-patient clinic or the in-patient department. Short-term hospitalization due to study requirements (such as the collection of cerebrospinal fluid) will be not considered as a serious adverse event.

The investigator should evaluate and record the serious adverse events in the CRF as follows: severity, relationship with the study drug, measures taken with the study drug, and outcomes. The sponsor should promptly notify hospitals involving in the study and ensure that the reporting procedures required by all laws and regulations are met.

### 9.3.5 Actions Taken for AE

#### 9.3.5.1 Record and Report

The investigator should explain to the subject that he/she is required to truthfully feedback the change of desease after administration. Physicians should avoid inducing subjects when asking questions, pay attention to adverse reactions or unexpected side effects when observing the efficacy (including symptoms, signs, laboratory tests). Regardless of whether the adverse reactions or adverse events are related to the study drug, it should be recorded in detail, including the onset time, symptoms and signs, severity, duration, laboratory test indicators, treatment methods and results, follow-up time, etc. The use of concomitant medication is also required to be recorded in detail to analyze the relationship of adverse reactions with the study drug. All records should be signed and dated.

#### 9.3.5.2 Emergent Unblinding

For the emergency unblinding envelopes dispensed together with the study drug, they can only be opened by the principle investigator of the study center when the serious adverse event occurs in the subject and it is necessary to know what drug is used, i.e. emergent unblinding. Once unblinded, the subject will withraw from the trial and treated as a drop-off case. The results will be notified to the monitor and the reason will be detailed in the case report form which should be signed and dated.

#### 9.3.5.3 Treatment of Patients

When an adverse reaction is observed, the investigator may take necessary measures depending on the condition such as continuous observation without treatment interruption ( If the reaction disappears, the drug can be used until the end of treatment period), continuous observation with drug suspension ( If the reaction disappears or is not worsenning , the drug can be used until the end of treatment period), treatment discontinuation and concomitant medication,etc. In case that the patient is found not tolerable, the treatment must be discontinued even if the adverse reaction is mild.

In the event of a serious adverse event, the site undertaking the clinical study must immediately take the necessary measures to protect the safety of the subject. All adverse events should be followed up, the treatment and results should be recorded in detail until resolved or the condition is stable. If the laboratory indicators are normal before the treatment but turns abnormal after treatment, the patient should be followed up until returning to normal. Follow-up methods may include hospitalization, outpatient visit, home visit, communication and other forms according to the severity of adverse reactions.

The treatment and outcome of adverse events should be recorded on the CRF. All adverse events should be followed up until relief or stabilization. The study monitor will check contents of the CRF at any time.

#### 9.3.5.4 Follow-up of Adverse Events

The follow-up is not limited to the duration of study. For some subjects, the drug-related AE may still persist after the end of study, such AE should be followed up until any of the following condition is met:

（1）Resolved

（2）Stable

（3）Return to the baseline level

（4）Attributable to medication other than the study drug or not related to the study behavior;

（5）More information is not available (the patient refused to provide more information, or there is evidence that the patient has been lost to follow-up after the best efforts).

Some events requiring hospitalization or prolongation of existing hospitalization may not be considered serious adverse events, including hospitalization for other reasons rather than adverse events, scheduled surgery before the study or other treatment or examination purposes.

# 10. Regulations of Blinding, Preservation of Blind Codes and Unblinding

## 10.1 Blinding and Preservation of Blind Codes

This trial is a randomized, double-blind, multi-center clinical trial. Statistician is responsible for random blinding of the study drug and generation of a random number table. The random number table is generated using simple randomization with proc plan process of SAS 9.1.3 software. Random seed number and random number table generated by two randomizations will be separately preserved as drug blind codes. A corresponding emergency letter will be generated for each random number while the random table is being generated. The drug is blinded by a biostatistician according to the above-mentioned blind codes. Each subject will be assigned a kit containing all medications during the study. The label of the kit is numbered, and the same number is also written on the label of all the medicines in the box. After the drug are dispensed, the blind codes are sealed. The blind codes of drug are stored in triplicate in Shanghai Mental Health Center affiliated to Shanghai Jiaotong University School of Medicine, Purisheng (Beijing) Pharmaceutical Technology Development Co., Ltd. and Shanghai Green Valley Pharmaceutical Co., Ltd., the blind codes may not be opened during the trial.

The emergency letter is distributed to the study sites along with the study drugs with the corresponding number and is kept by the principle investigator.

## 10.2 Emergent Unblinding

When there are serious adverse events or patients require rescue and it is necessary to know what drug is used, the trial director at the study center will report to the clinical study director and emergency unblinding can be performed once agreement is obtained. The principal investigator in each study center can obtain information on specific treatment assignment via IWRS system . Once emergency unblinding is performed, the subject is treated as drop out case. Time, place, reason and participants in unblinding should be recorded promptly. These will be reported to the sponsor, CFDA and clinical study director in a timely manner.

## 10.3 Regulations of Unblinding

Since the study will adopt a 1:1:1 design, the second unblinding method will used for unblinding.Unblinding is performed after blind review, confirmation of statistical analysis plan and data lock.

# 11. Data Management

## 11.1 Data Collection and Revision

Electronic data capture (EDC) is used for data collection. Data capture interface is designed according to the protocol. Investigators perform data collection and record. Data managers review data submitted by monitors. If there is any question, investigators will be inquired in EDC system. Investigators will check, revise or confirm data. All data revisions will be recorded in detail in EDC. The system will set corresponding authorization according to user’s role.

## 11.2 Verification of Source Records

Investigators must carefully handle all data obtained in the clinical study, ensuring rights and privacy of patients participating in the clinical study. Investigators must allow access to and review of all required clinical study data by monitors to verify accuracy of source data and get updated on progress of the study. If source records can not be verified, investigators should allow further confirmation of data quality by monitors/auditors/inspectors.

## 11.3 Blind Review and Unblinding

Blind review refers to check and evaluation of data in the database after the last case report form is entered into the database and before the first unblinding.

When all case report forms are entered into the database and confirmed to be correct, the data manager will develop the database examination report, including completion of the trial (including a list of drop out subjects), verification of inclusion/exclusion criteria, integrity logical consistency, outlier data, time window, concomitant medication and adverse events.

In blind review meeting, principal investigator, the sponsor, monitors, data manager and biostatisticians will review the informed consent form signed by subjects, maintenance of blinding during the trial and emergency unblinding in the trial, make decisions on questions proposed in database examination report, complete final definition and judgment of analysis population and write blind review report. The database will be locked at the same time。

## 11.4 Data Lock

Data can be locked when the following conditions are met:

（1）All data are collected

（2）All queries are resolved

（3）Analysis population is defined.

Data lock will be completed by the data manager. Locked data are sent to statisticians for analysis.

# 12. Statistical Analysis Plan

## 12.1 Sample Size

A total of 252 subjects (including 20% drop-out cases) are required to be enrolled in about 20 sites across the country. It is estimated to enrol 210 eligible subjects (70 subjects for each group). Enrollment period is about 6 months. The software of PASS is used to calculate sample size. If 70 subjects are included in each group, there is 80% power to detect effect size of at least 0.5 (effect size, ∆/δ) between test group and placebo control group at two-sided alpha level of 0.05. Considering 20% drop out cases, approximately 84 subjects will be enrolled in each group, totaling 252 subjects in all 3 groups.

## 12.2 Study Parameters

All parameters and study factors will be analysed using SAS software.

## 12.3 Analysis Sets

### 12.3.1 Full analysis set (FAS)

Subjects will be excluded in a minimal and rational manner based on the principle of Intention-To-Treat (ITT). The full analysis set (FAS) will contain all subjects in the RND set who received at least one dose of double-blind study drug, and have a baseline value and at least one post baseline efficacy assessment on treatment. For missing values related to the efficacy of FAS, they will be imputed using the method of last observation carry forward (LOCF).

### 12.3.2 Per-protocol (PP) set

FAS subjects without major protocol deviation will be included in the Per-protocol set. Major protocol deviations will be defined before unblinding. Generally, subjects meeting the following criteria will compose the PP population: ① compliance between 80-120%; and ② no prohibited drugs are used during the trial and ③ meet all inclusion criteria but do not meet any exclusion criteria and ④ complete all scheduled visits and contents specified in CRF.

In this set, missing data will not be imputed using the LOCF.

### 12.3.3 Safety set (SS)

Safety set includes all enrolled subjects who receive at least one dose and have appropriate subsequent information for safety analysis. All safety information of subjects will be evaluated, including adverse events and laboratory examination results.

## 12.4 General Considerations of Statistical Analysis

SAS statistical software will be used for all statistical analysis. Contents of analyses will be described in detail in statistical analysis plan (SAP), which will be reviewed at blind data review meeting and finalized before unblinding. All statistical tests will use two-sided test. P≤0.05 indicates statistically significant difference. For description of quantitative variables, mean, standard deviation, median, minimum and maximum will be calculated; for description of categorical variables, number and percentage will be calculated. Changes from baseline will be calculated as (visit – baseline).

Multiplicity adjustment: verifying dose-response relationship of the primary endpoint is the main content of analysis in this study. Other further analyses are all exploratory. No multiplicity adjustment will be performed for class I errors.

## 12.5 Analysis of Statistical Methods

Baseline parameters (gender, age, race, and course of Alzheimer’s disease, body weight, height and vital signs at visits) will be summarized using descriptive statistics. The drug-placebo comparison: analysis of variance is performed for continuous data with normal distribution. Kruskal-Wallis rank sum test is performed for data with non-normal distribution. χ2 test is used for discontinuous data. For ranked data, Kruskal-Wallis rank sum test will be used.

### 12.5.1 Efficacy Analysis

All are quantitative variables. Paired t test or Wilcoxon one-sample test is used for comparison before and after treatment in each group. For drug-placebo comparison after treatment, the difference before and after treatment is used in analysis of covariance and the corresponding variable before treatment is used as the covariance. When necessary, sensitivity analysis and subgroup analysis will be performed.

Hypothetical test：H_0_: The change of primary endpoint,i.e ADAS-Cog score at 24 weeks from the baseline is the same for all 3 groups, i.e. the low-dose group (Sodium oligomannate at 600mg), the high-dose group (Sodium oligomannate at 900mg ) and the control group (placebo).

H_1_: The change of primary endpoint,i.e ADAS-Cog score at 24 weeks from the baseline is not the same for all 3 groups, i.e. the low-dose group (Sodium oligomannate at 600mg), the high-dose group (Sodium oligomannate at 900mg ) and the control group (placebo).

### 12.5.2 Primary Efficacy Endpoint

The efficacy analysis will be performed to both the FAS and PPS population.

ADAS-Cog score will be summarized for the scheduled visit and treatment group using descriptive statistics. Covariance analysis model (ANCOVA) is used to compare change before and after treatment (24weeks – baseline) in each group, using baseline value as the covariates in model, while treatment group and study center are used as fixed effect. Based on this model, change of each endpoint after 24-week treatment from baseline in each group, and least squares mean difference in change between the two groups and 95% confidence interval are calculated and stratified according to different blood APOEε4 genotypes. The mixed model for repeated measure (MMRM) will be used as reference analysis for the primary endpoint of ADAS-Cog12 before and after treatment (24weeks – baseline) in each group.

### 12.5.3 Secondary Efficacy Endpoints

The efficacy analysis will be performed to both the FAS and PPS population.

Secondary efficacy endpoints including CIBIC-plus, ADCS-ADL, NPI questionnaire, CSF biomarkers (Aβ_40_, Aβ_42_, T-tau, P-tau) abd glucose metablism of PET imaging will be summarized for the scheduled visit and each treatment group using descriptive statistics. The same analysis method with primary endpoint will be adopted to analyse the change of each group from baseline at 24 weeks in secondary endpoints which will be stratified according to different blood APOEε4 genotypes.

## 12.6 Safety Analysis

The safety analysis will be performed for the safety set (SS).

After the AE and ADR are coded, all events will be analyzed for treatment group, the number of subjects, incidence, and the number of envents by severity.

For continuous viariales of vital signs and ECG, the observed values at each time point and change from the baseline will be summerized using descriptive statistics.

For laboratory tests, listings will be given as per the determination by investigators by normal, abnormal without clinical significance, and abnormal with clinical significance.

# 13. Quality Control of the Trial

## 13.1 Consistent Training of Scale Rating

To enture consistency of clinical evaluation results provided by different investigators and to avoid difference in results from different evaluation personnel, consistent training on clinical evaluation will be provided for investigators in all centers before formal initiation of the trial:。

## 13.2 Data Monitoring committee (DMC)

This study will establish the independent Data Monitoring Committee (DMC). DMC will consist of professionals who are not involved in this clinical trial and responsible for the review and assessment of the safety data to protect the safety of the subjects and comply with the requirements of protocol and related regulations.

## 13.3 Clinical Monitoring

During the trial, monitors designated by the sponsor will conduct on-site monitoring visits to the study center on reglar basis to ensure that the study protocol is fully and strictly implemented and the study data is accurately completed.

（1）Personnel participating in the trial must have central training to unify recording method and judgment criteria.

（2）The entire clinical trial is conducted under blind state.

（3）Clinical study monitors are responsible to be familiar with all investigators and personnel in all clinical study centers involved in each step of the clinical trial (including distribution of study drugs).

（4）Monitors must regularly monitor works in clinical study centers and inspect clinical study centers at least once before enrollment of the first subject, during the clinical study and after end of the clinical study.

（5）Monitors and current investigators jointly verify whether the clinical trial strictly follows the clinical study protocol and solve any issue emergent in the clinical study with investigators. Investigators should truly, carefully record each item in CRF in detail according to requirements to ensure contents in case report form are true and reliable.

（6）Reference ranges in testing lab are used as judgment criteria for laboratory abnormalities.

（7）In the clinical trial, all observation results and findings should be checked and informed consent form must be reviewed for all enrolled subjects. This is to ensure data are reliable and conclusions of the clinical trial are based on original data. In the clinical trial and data processing stage, there should be corresponding data management measures.

（8）For potential drop-off cases, active measures should be taken to control drop-off rate within 20%.

## 13.4 Collaborative Monitoring and Audit

All drugs and materials used in the clinical study are subject to quality control. The sponsor, personnel authorized by the sponsor or relevant medical management departments have the right to perform systemic inspection on clinical study related activities and documents, to ensure the trial is conducted according to the study protocol, standard operating procedures and relevant regulations, and trial data are truly, accurately and completely recorded in time. Subjects in the clinical study will be informed of clinical study audit. However, subjects’ privacy and data will be strictly protected.

During the trial, project manager will assist each clinical monitor in collaborative monitoring, so as to find and solve issues in time. Independent quality control department will select several centers for on-site autit and study documents of all centers will be subject to audit.

# 14. Ethical Requirements

This trial will be conducted according to requirements in Declaration of Helsinki（2008）, GCP and relevant regulations in China. Before initiation of the trial, the clinical trial can only be conducted after the study protocol is approved by the Ethics Committee in clinical research institution.

Investigators are responsible to provide the clinical study protocol, specific patient information page and a copy of the informed consent form to the Ethics Committee, so as to obtain independent approval document for this clinical study.

Before initiation of the clinical study, approval document must be obtained from the Ethics Committee. Approval document from the Ethics Committee must be provided in written to investigators, who will then provide the sponsor with a copy of the approval document. Approval document provided by the Ethics Committee should have a list of committee members participating in discussion on approval document and corresponding responsibilities.

In the clinical study, any issues related to clinical study safety must be reported to the Ethics Committee in time, such as revision of the clinical study protocol or patient information page and serious adverse events in the clinical study. End or premature termination of the clinical study must also be reported to the Ethics Committee.

# 15. Informed Consent Form/Data Protection Agreement

When obtaining and signing the informed consent form, investigators must comply with requirements of GCP, Declaration of Helsinki and relevant regulations. Before trial related activities, investigators must inform subjects of trial related information verbally and in written as subjects can read and understand. Investigators must ensure subjects have sufficient time to determine whether they will participate in this trial. Before any trial related activity, the informed consent form voluntarily signed and dated by subjects must be obtained.

Informed consent can only be performed by qualified medical professionals designated by investigators. The written informed consent form must be signed and dated by the personnel performing informed consent.

# 16. Withdrawal from the Clinical Study

Investigators have the right to ask the subject to withdraw from the clinical trial for any reason related to his/her rights, including other concomitant diseases, adverse events or treatment failure. Investigators must specify the reason for withdrawal, regardless of when and why the subject withdraws from the clinical trial. For subjects withdrawing due to concomitant diseases or adverse events, detailed explanation must be recorded in case report form, along with other relevant data on concomitant diseases or adverse events.

# 17. Patients’ Privacy

Study personnel must protect privacy of subjects in the clinical trial. In all documents submitted to the sponsor, subjects are only identified with numbers, rather than names or inpatient numbers. Investigators must carefully keep subjects' name and address, and enrollment forms corresponding to subject numbers. These enrollment forms are strictly kept by investigators confidentially and not submitted to the sponsor.。

# 18. Revision of the Clinical Protocol

Revision of the clinical protocol must be jointly developed by the sponsor and investigators. Major revision should be submitted to the Ethics Committee/Institutional Review Board. These procedures should be completed before revison comes into effect.

# 19. Case Report Form (CRF)

This study will use EDC system. Investigators must ensure to completely, accurately fill electronic case report form(eCRF). Each CRF is only used for data records of one subject.

# 20. Verification of Source Data

Investigators must properly handle all data obtained in the clinical study, ensuring the protection of rights and privacy of patients participating in the clinical study. Investigators must allow access to and review of all required clinical study data by monitors/auditors/inspectors to verify accuracy of source data and learn progress of the study. If source records can not be verified, investigators should cooperate monitors/auditors/inspectors to further confirm the quality of data quality.

# 21. Publication

For a multi-center clinical trial, data of each center can not be publlished separately before the publication of the overall study results and the sponsor reserves the right to review the draft publications.

# 22. Preservation of Study Data

Investigators must agree to archive all study related source documents according to the regulatory requirements. A copy of all clinical research data must be kept for at least 5 years after the end of the trial. The ownership of all information in this clinical trial belongs to the sponsor. Except as required by the CFDA, the investigator may not provide it to the third party in any way without the written consent of the sponsor.

# 23. Responsibilities of Each Party

The sponsor, investigators, leading research institution and participant institutions must assume corresponding responsibilities according to Good Clinical Practice (2003) and this protocol. The sponsor will provide clinical trial insurance to subjects participating in this clinical study.

# 24. Clinical Research Institution

（1）Clinical Study Centers

Leading Study Center: Shanghai Mental Health Center, Shanghai Jiao Tong University School of Medicine

Address: 600 Wanping South Road, Xuhui District, Shanghai Postcode: 200030

Tel: 021-64387250

Study Centers: 20-30 study centers across the country

（2）Statistical Analysis

GCP ClinPlus Co.，Ltd.

Address: 9th Floor, Qingdong Business District, No. 1 Lane Gully, Haidian District, Beijing Postcode:100089

Tel: 010-51298908

（3）Sponsor

Shanghai Green Valley Pharmaceutical Co., Ltd.

Address: 421 Newton Road, Pudong New Area, Shanghai Postcode: 201203

Tel: 021-50504988

（4）Contract Research Organization（CRO）

GCP ClinPlus Co.，Ltd.

Address: 9th Floor, Qingdong Business District, No. 1 Lane Gully, Haidian District, Beijing Postcode:100089

Tel: 010-51298908

（5）Archiving Unit of Study Data

The source data of the trial is kept by each study center and preserved at least 5 years after the drug is marketed.

# 25.Reference

（1）Alzheimer's Association.2010 Alzheimer's disease facts and figures.Alzheimers Dement,*2*010,6:158–194.

（2）Masters CL,Cappai R,Barnham KJ,et al.Molecular mechanisms for Alzheimer's disease:implications for

neuroimaging and therapeutics.J Neurochem,2006,97(6):1700-1725.

（3）Rodriguez J,Ferri C,Acosta D,et al.Prevalence of dementia in Latin America,India, and China:a pop-

ulation-based cross-sectional survey.Lancet,2008,online first on July 28,2008.

（4）Hebert LE,Beckett LA,Scherr PA,et al.Annual incidence of Alzheimer disease in the United States

projected to the years 2000 through 2050.Alzheimer Disease & Associated Disorders,2001,15:169–173.

（5）Personal Social Services Research Unit (PSSRU).A report to the Alzheimer's Society on the prevalence and economic cost of dementia in the UK produced by King's College London and London School of Economics. London: Alzheimer's Society,2007.

（6）Leung GM,Yeung RY,Chi I,et al.The economics of Alzheimer disease.Dement Geriatr Cogn Disord,2003,15:34–43.

（7）[Masters CL](http://www.ncbi.nlm.nih.gov/pubmed?term="Masters CL"%5BAuthor%5D), [Multhaup G](http://www.ncbi.nlm.nih.gov/pubmed?term="Multhaup G"%5BAuthor%5D), [Simms G](http://www.ncbi.nlm.nih.gov/pubmed?term="Simms G"%5BAuthor%5D)，et al.Neuronal origin of a cerebral amyloid: neurofibrillary tangles of Alzheimer's disease contain the same protein as the amyloid of plaque cores and blood vessels.[EMBO J,](javascript:AL_get(this, 'jour', 'EMBO J.');)1985,4(11):2757-2763.

（8）GoIlg Y,Chang L,Viola KL,et a1.Alzheimer's disease-affected brain：presence of oligomeric A beta ligands(ADDls) suggests a molecular basis for reversible memory loss.Prec Nail Acad Sci USA, 2003,

100(8):10417-10422．

（9）Barrow CJ,Small BJ.Aβ Peptide and Alzheimer's Disease. London, England:Springer-Verlag,2006:5-32.

（10）Ling-na Kong, Mei-yu Geng, Liang Miao, et al. Effect of acid oligosaccharide on gene expression in brain of Alzheimer's disease model mic. Journal of Pharmacy, 2005，40(12)：1105-1109.

（11）Fan Ying,Hu Jinfeng,Li Jing,et al.Effect of acidic oligosaccharide sugar chain on scopolamine-induced memory impairment in rats and its related mechanisms.Neurosci Lett.2005,374(3):222-226.

（12）Hu Jinfeng,Geng Meiyu,Li Jing,et al.Acidic oligosaccharide sugar chain,a marine-derived acidic oligosaccharide,inhibits the cytotoxicity and aggregation of amyloid beta protein.J Pharmacol Sci，2004,95(2):248-255.

（13）CFDA. Technical guidelines for the Clinical Trials of anti-AD Drugs.2007.

（14）European Medicines Agency.Guideline on medicine products for the treatment of Alzheimer disease and other dementias.2009.

（15）Qi-dong Yang, Lin Zhou, The treatment of Alzheimer's disease with β - amyloid peptide as a target.

Internal medicine; its theory and practice, 2007, 2(2):94-97.

（16）Chun-xi Liu, Yao-de Huang. Progressive study of β-amyloid therapy in Alzheimer's disease. Chinese

clinical rehabilitation, 2003,7（28）:3867-3869.

（17）Jia Liu, Wen-xin Dong. Development of innovative drugs for Alzheimer's disease. China Pharmaceutical

Industry Journal, 2007, 38(7):528-532.

（18）McKhann G,Drachman D,Folstein M,et al.Clinical diagnosis of Alzheimer's disease: report of the

NINCDS-ADRDA Work Group under the auspices of Department of Health and Human Services Task

Force on Alzheimer's Disease.Neurology,1984,34(7):939-944.

# 26. Appendix

## Appendix 1 Mini-Mental State Examination (MMSE)

INSTRUCTION: Now I want to ask you some questions, most are simple, please carefully answer.

| Evaluation items | Correct | Wrong | Score |
| --- | --- | --- | --- |
| 1 Please tell me: |  |  |  |
| What year is it now? | 1 | 0 | □ |
| What season is it now? | 1 | 0 | □ |
| What month is it now? | 1 | 0 | □ |
| What's the date today? | 1 | 0 | □ |
| What day is today? | 1 | 0 | □ |
| What city is this (city name)? | 1 | 0 | □ |
| What area is this (district name)? | 1 | 0 | □ |
| What street is this? | 1 | 0 | □ |
| Which floor is this? | 1 | 0 | □ |
| What place is this? | 1 | 0 | □ |
| 2 Now I tell you the names of three things, after I have finished you repeat and remember them, then I will ask you later. "Ball", "flag", "trees." Please repeat (careful state it clearly, say everything with a second, if the patient can not completely say, the words can be repeated up to six times, but remember the first pass score). | | | |
| Rubber ball | 1 | 0 | □ |
| National flag | 1 | 0 | □ |
| Trees | 1 | 0 | □ |
| 3 Now, if you do the math, subtract 7 from 100, and the resulting number minus 7, keep counting down, tell me the answer after each minus 7, until I say "stop" so far (Each correct answer 1 point, if one is wrong, such as 100-7 = 90, the next is right, such as 90-7 = 83, and the second still scores). | | | |
| 100-7=93 | 1 | 0 | □ |
| 93-7=86 | 1 | 0 | □ |
| 86-7=79 | 1 | 0 | □ |
| 79-7=72 | 1 | 0 | □ |
| 72-7=65 | 1 | 0 | □ |
| 4 Now please tell me what three things are those that I asked you to remember one moment ago? | | | |
| Rubber ball | 1 | 0 | □ |
| National flag | 1 | 0 | □ |
| Trees | 1 | 0 | □ |
| 5 (examiner shows a watch) What is this? | 1 | 0 | □ |
| (Examiner shows a pencil) what is this? | 1 | 0 | □ |
| 6. Please repeat after me "we work together to pull tight rope" | 1 | 0 | □ |
| 7. "Please close your eyes," please read these words, and follow the meaning of this sentence. | 1 | 0 | □ |
| 8. I give you a piece of paper, please do what I said: "Pick up the paper with the right hand, fold it up with both hands, and put it on your left leg". | | | |
| Pick up the paper with the right hand | 1 | 0 | □ |
| Fold it up with both hands | 1 | 0 | □ |
| Put it on the leg | 1 | 0 | □ |
| 9. Would you please write a sentence (written by patients themselves, must have a subject, predicate, and certain content. Grammar, punctuation, and spelling errors can be ignored) 　　　　　　　 　　　 1 0 □ | | | |
| 10. Would you please draw it down according to this way (must draw 10 angles, must draw two pentagons crossed, cross graphics must be quadrilateral to score, uneven lines can be ignored) | | | |
| 1 0 □ 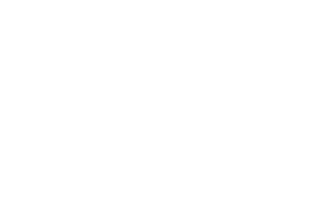 | | | |
| Total score | | | □□ |

## Appendix 2 Hachinski Ischemic Scale score (HIS)

| Items | Yes | No |
| --- | --- | --- |
| 1 An acute onset | 2 points | 0 point |
| 2 Ladder aggravated | 1 point | 0 point |
| 3 Volatility course | 2 points | 0 point |
| 4 Night confusion | 1 point | 0 point |
| 5 Relatively intact personality | 1 point | 0 point |
| 6 Depression | 1 point | 0 point |
| 7 Somatic discomfort complaints | 1 point | 0 point |
| 8. Emotional instability | 1 point | 0 point |
| 9. Previous history of hypertension | 1 point | 0 point |
| 10. Previous history of stroke | 2 points | 0 point |
| 11 With atherosclerosis | 1 point | 0 point |
| 12 Focal neurological symptoms | 2 points | 0 point |
| 13 Focal neurological signs | 2 points | 0 point |

Note: The total score ≥ 7 points is vascular dementia, ≤ 4 points is Alzheimer's disease, score between them is mixed dementia.

## Appendix 3 Hamilton Depression Scale (HAMD)

INSTRUCTION: Ask the patient himself, evaluate the patient for performance of nearly one month.

| Items | 0 | 1 | 2 | 3 | 4 | Score |
| --- | --- | --- | --- | --- | --- | --- |
| 1 Depression | None | State only when asked | Express spontaneously in conversation | Showing emotions not with words but from facial expressions, gestures, sounds or desire to cry | The patient's verbal and non-verbal (facial expressions, movements) expressed this sentiment almost entirely | □ |
| 2 Guilt | None | Blame themselves, feel that they have hurt others | Think they committed a crime, or ruminating past mistakes and errors | The disease is currently considered punishment to themselves, or evil delusions | Evil delusions accompanied by accusations or hallucinations threatening | □ |
| 3 Suicide | None | Feel being alive does not make sense | I hope I have died, or often think about things about death | Negative perceptions, thoughts of suicide | There are serious suicidal behavior | □ |
| 4Difficulty falling asleep | None | Sometimes complained of difficulty falling asleep, half an hour after going to bed still can not sleep | Complained of nightly difficulty falling asleep | / | / | □ |
| 5 Sleep is not deep | None | Shallow sleep, many nightmares | Midnight (before 12:00 am) had to wake up (not including using the toilet) | / | / | □ |
| 6 Waking up early | None | Waking up early, waking one hour earlier than usual, but can go back to sleep | Can not go back to sleep after waking | / | / | □ |
| 7 Work and interest | Normal | States when asked | Spontaneous expression of , directly or indirectly, loss of interest in the activity, work or study, for example you feel listless, indecisive, can not adhere to or need to force yourself to go to work or activities | Time of activity reduced or effectiveness reduced, inpatient to participate in work or entertainment no more than three hours a day | Stops working due to the current diseases, inpatients do not participate in any activities or can not complete the daily affairs of the ward without help from others | □ |
| 8 Slow: slow thinking and speech, difficulty concentrating, decreased initiative | None | Psychiatric examination revealed mild mental retardation | Psychiatric examination found significant mental retardation | Psychiatric examination conducted with difficulties | Completely unable to answer questions (stupor) | □ |
| 9 Agitation | None | A bit distracted during examination | Obviously distracted or multi-trick | Can not sit, had to stand once during examination | Hand-wringing, biting, pulling hair, biting lips | □ |
| 10 Mental anxiety | None | States when asked | Spontaneous expression | Expression and speech reveals significant concerns | Obvious panic | □ |
| 11 Somatic anxiety: dry mouth, bloating, diarrhea, belching, abdominal cramps, palpitations, headache, hyperventilation and sigh, as well as frequent urination and sweating. | None | Mild | Moderate, there is definitely the above symptoms | Severe, above symptoms are severe, affect the life, need to be handled | Seriously affect the life and activities | □ |
| 12 Gastrointestinal symptoms | Normal | Loss of appetite, but they do not need others to encourage , self-feeding | Need others to urge or to request to eat, or need use laxatives or digestants | / | / | □ |
| 13 Systemic symptoms | None | Limbs, back of the neck heaviness, backache, headache, muscle pain, malaise | Symptoms obvious | / | / | □ |
| 14 Sexual symptoms: loss of libido, menstrual disorders, etc. | Normal | Mild | Severe | Subjects were not sure or do not fit, are not included in the total score |  | □ |
| 15 Hypochondriacal | None | Too much attention to the body | Mulled over health problems | There are hypochondriacal delusions | Hypochondriacal delusions with hallucinations | □ |
| 16 Weight loss | None | More than half kg weight loss over the week | More than 1 kg weight loss over the week |  |  | □ |
| 17 Insight | Yes | Knew he was sick, but attributed to poor food, the environment, busy with work, etc. | Deny sick |  |  | □ |
| 18 Total score | -- | -- | -- | -- | -- | □□ |

Reference values: <7 no depression; 7～17 mild depression; 18～24 moderate depression; > 24 severe depression.

## Appendix 4 Alzheimer's Disease Assessment Scale - cognitive portion (ADAS-cog)

| **1. Word memory** | | | |
| --- | --- | --- | --- |
| **Words** | **First time** | **Second time** | **Third time** |
|  | **Recalled not recalled** | **Recalled not recalled** | **Recalled not recalled** |
| Family  Coins  Railroad  Child  Army  Flag  Skin  Library  Wheat  Ocean | □ □  □ □  □ □  □ □  □ □  □ □  □ □  □ □  □ □  □ □ | □ □  □ □  □ □  □ □  □ □  □ □  □ □  □ □  □ □  □ □ | □ □  □ □  □ □  □ □  □ □  □ □  □ □  □ □  □ □  □ □ |
| Score |  |  |  |

| **2. Naming objects** | **Right** | **Wrong** |  | **Right** | **Wrong** |
| --- | --- | --- | --- | --- | --- |
| Flower  Sofa  Whistle  Pencil  Shuttlecock  Mask  Scissors  Comb  Wallet  Harmonica  Stethoscope  Pliers | □  □  □  □  □  □  □  □  □  □  □  □ | □  □  □  □  □  □  □  □  □  □  □  □ | The thumb  The index finger  The middle finger  The ring finger  pinkie | □  □  □  □  □ | □  □  □  □  □ |

| 3 Command | **Right** | **Wrong** |
| --- | --- | --- |
| Make a fist | □ | □ |
| Point to the ceiling, and then point to the ground | □ | □ |
| Put the pencil on the card, then put it back | □ | □ |
| Put the watch on the other side of the pencil, and then turn over the cards | □ | □ |
| Tap on each side of the shoulder twice with two fingers of one hand, but should have been closed eyes | □ | □ |

| **4. Structured exercises** | **Right** | | **Wrong** |
| --- | --- | --- | --- |
| Circle  Stacked rectangular  Diamond  Cube | □  □  □  □ | | □  □  □  □ |
| **5. Intention exercises** | **Right** | **Wrong** | |
| Stack letters  Put the letter into the envelope  Seal the envelope  Write addresses on envelopes  Marking the place of stamp | □  □  □  □  □ | □  □  □  □  □ | |

| **6. Orientation** | **Right** | **Wrong** |
| --- | --- | --- |
| Full name  Week  Date (can be different by one day)  Month  Years  Season (one week before / 2 weeks after season changes)  Hour of the day ( error within one hour)  Location (part naming are also acceptable) | □  □  □  □  □  □  □  □ | □  □  □  □  □  □  □  □ |

Score

1 word recall tasks: 2 naming objects or fingers

3 the command: 4 structural exercises:

5 intention exercise: 6 orientation:

| **7. Words identification task** | | | | | | | | |
| --- | --- | --- | --- | --- | --- | --- | --- | --- |
|  | **Yes**  **（old）** | **No**  **（new）** |  | **Yes**  **（old）** | **No**  **（new）** |  | **Yes**  **（old）** | **No**  **（new）** |
| **Silence**  **Elbow**  **Daughter**  **Powder**  **Canal**  **Forehead**  **Tiger**  **Dawn**  **Dragon**  **Bedroom**  **Sister**  **Beggar**  **Echo**  **Nephew**  **Obligation**  **Village**  **Corner**  **Olive tree**  **Music**  **Courage**  **Container**  **Ribbon**  **Object**  **Necklace**  **Score** | □ |  | Bubble  Corner  Jewelry  Shower  Village  Forehead  Silence  Tiger  Meeting  Container  Car  Onion  Beggar  Alert  Echo  Courage  Daughter  Object  Organ  Beverages  Basin  Jacket  Dawn  Mayor  Score |  | □ | Monkey  Silence  Islands  Season  Dawn  Needle  Echo  Cow  Corner  Kingdom  Tiger  Object  Beggar  Fountain  Village  People  Hunter  Forehead  Pitcher  Container  Daughter  Courage  Shell  Lily  Score |  | □ |
|  |  | □ |  | □ |  |  | □ |  |
|  | □ |  |  |  | □ |  |  | □ |
|  |  | □ |  |  | □ |  |  | □ |
|  |  | □ |  | □ |  |  | □ |  |
|  | □ |  |  | □ |  |  |  | □ |
|  | □ |  |  | □ |  |  | □ |  |
|  | □ |  |  | □ |  |  |  | □ |
|  |  | □ |  |  | □ |  | □ |  |
|  |  | □ |  | □ |  |  |  | □ |
|  |  | □ |  |  | □ |  | □ |  |
|  | □ |  |  |  | □ |  | □ |  |
|  | □ |  |  | □ |  |  | □ |  |
|  |  | □ |  |  | □ |  |  | □ |
|  |  | □ |  | □ |  |  | □ |  |
|  | □ |  |  | □ |  |  |  | □ |
|  | □ |  |  | □ |  |  |  | □ |
|  |  | □ |  | □ |  |  | □ |  |
|  |  | □ |  |  | □ |  |  | □ |
|  | □ |  |  |  | □ |  | □ |  |
|  | □ |  |  |  | □ |  | □ |  |
|  |  | □ |  |  | □ |  | □ |  |
|  | □ |  |  | □ |  |  |  | □ |
|  |  | □ |  |  | □ |  |  | □ |
|  |  |  |  |  |  |  |  |  |

1. **Words identification task：**

|  | **Score** | **Score criteria** |
| --- | --- | --- |
| 8. Recall test instructions | □ | 0 = never need additional Tips instructions  1 = very mild, forget once  2 = mild, must be reminded twice  3 = moderate, 3 or 4 times to remind  4 = moderately severe, 5 or 6 times to remind  5 = severe, must be reminded 7 times or more than 7 times... |
| 9. Spoken language ability | □ | 0 = no situation difficult to understand  1 = very mild, there is one case of lack of comprehensibility  2 = mild, for less than 25% of time there are difficulties in speech intelligibility  3 = moderate, for 25% -50% of the time there are difficulties in speech intelligibility  4 = moderate to severe, for more than 50% of the time there are difficulties in speech intelligibility  5 = severe, say one or two words then interrupt; speak fluently, but lacking in substance; silence |
| 10. (in the process of spontaneous speech ) difficulty in finding words | □ | 0 = no evidence of difficulties in finding words during spontaneous speech process  1 = very mild, difficulty finding the word once or twice, the clinical significance is not obvious  2 = mild, obvious repeat or replaced with synonyms  3 = moderate, and sometimes lack of the word, and no alternative words  4 = moderate to severe, frequent lack of words, and no alternative words  5 = severe, almost complete loss of words with contents; speech sounds hollow; say one or two words then interrupt |
| 11. The spoken language understanding ability | □ | 0 = no evidence of poor ability to understand  1 = very mild, there is one case of wrong understanding  2 = mild, 3-5 cases of the wrong understanding  3 = moderate, require repeating and repeatedly explaining phrases to be understood  4 = moderate to severe, only occasionally respond correctly; namely: "yes" or "no" questions  5 = severe, subjects rarely make appropriate response to the question, and not caused by poor speech |
| 12. Attention | □ | 0 = no evidence of poor attention or distractibility  1 = very mild, there is one time of inattention  2 = mild, 2-3 times inattention or distractibility; signs of irritability and distracted appeared  3 = moderate, 4-5 times of inattention during the interview process  4 = moderate to severe, often inattention and / or frequent distractibility during the interview process  5 = severe and extremely difficult to concentrate, distractibility appeared numerous times, unable to complete the test task |

Score

8. Recall test instructions ：

9. Spoken language ability：

1. difficulty in finding words :

11. The spoken language understanding ability:

12. Attention:

## Appendix 5 Clinician impression to changes through interviews (CIBIC-plus)

| Evaluator's Guide  CIBIC + consists of three parts:  The first part - the baseline assessment (including information from patients and caregivers both).  The second part - the scheduled visit periods and premature discontinuation test visit evaluation form (including information from patients and caregivers both).  The third part - clinicians’ impression to changes (done through evaluation of scheduled visits and premature discontinuation test visit).  Overall objective of CIBIC + is to provide a means for the reliable assessment of overall change from baseline in the clinical trials. It provides a semi-structured form, so that clinicians gather the necessary clinical information from patients and caregivers, in order to form an overall impression of clinical change.  The first part is used to record baseline information as a reference for future assessments.  The second part is a set of evaluation forms, and the purpose of design is to help clinicians gather the necessary information to assess changes since the beginning of the baseline assessment.  The third part is a case evaluation form of clinicians’ impression of change. The purpose of this form is for clinicians on a seven-point Likert-type scale to assess their impression to changes beginning from baseline.  Usage  The first part - the baseline assessment. At baseline assessment part, clinicians interview with patients and caregivers, record the baseline conditions in the recording field of the first part for later reference. Only in the part of the baseline assessment, clinical information about the patient can adopt information from the screening interview, medical history, physical and neurological examination.  The first, the second part adopt similar format in recording relevant clinical information. Column labeled with "Range" lists the various ranges may taken into account by clinician in assessing patients’ potential changes. Column labeled with "tips" provides some examples may be useful for clinicians in assessing a certain range. They are only used as a guide to collect information. The last column provides the space for use as a record. The form provides the corresponding blanks used to fill interviews records with caregivers and interviews records with patients.  Time for completing baseline form is not specified.  The second and third parts - the follow-up visits. The second and third part are used for scheduled visits period and the premature discontinuation visits. Order of visits with patients and caregivers is not required by the study.  After completing the second part of the evaluation form, in the third part, i.e. "clinicians impression to changes" section, clinicians in a seven-point Likert-type scale (from significantly improved to a significant deterioration) record his / her impressions of clinical change.  Clinicians’ impression to changes is the assessment of changes beginning from baseline, rather than an assessment of severity. In making change assessment, clinicians can refer to the baseline information pooled by him / herself in the first part. Then they shall copy clinicians’ evaluation of impression to changes into the master study case report form.  Clinicians need to make individually conclusions about change without consultation with other colleagues. In the beginning of the interview, clinicians should remind the interviewee to avoid mentioning any side effects he / she may be going through. |
| --- |

CIBIC PLUS—Baseline interviews

| Research Project No.: | Case No.: | | Interview window: | | | Date of visit: |
| --- | --- | --- | --- | --- | --- | --- |
| Interview start time:  Brief Description: See description sheet. Use this form to record baseline information for future assessment of change. Please refer to the guide page in the instructions on how to obtain related information. A brief assessment should be made on the patient's mental state . No specific requirements on the form or order of interview. | | | | | | |
| **Range** | | **Tips** | | | **Record** | |
| Relevant medical history | | 22 Recent relevant clinical events and diseases | | | Patient | |
|  |  |  |  |  | Caregiver | |
| Observation / evaluation | | 23 Appearance | | | Patient | |
|  |  |  |  |  | Caregiver | |
| **Mental / cognitive status** | | | | | | |
| Awakening /  Vigilance /  Attention / concentration level | | 24 Confusion / clarity  25 excitatory / reactivity  26 state of consciousness | | Patient | | |
|  |  |  |  | Caregiver | | |

**CIBIC PLUS—Ba**seline interview (continued)

| **Range** | **Tips** | **Record** | |
| --- | --- | --- | --- |
| **Mental / cognitive status (continued)** | | | |
| Orientation | 27 Time  28 Location  29 Character  30 Time relation  31 Travel  32 Reorientation  33 Identify yourself / others / objects  34 Reflect appropriate | | Patient |
|  |  |  | Caregiver |
| Memory | 35 Keep in mind  36 Recall  37 Long/far away  38 Memories of the past | | Patient |
|  |  |  | Caregiver |
| Language / speech | 39 Fluency degree/ expressive language, receptive language  40 Comprehension  41 heterophasia / difficulty finding the word  42 names identification, the number of words  43 Repeat  44 Follow the instructions | | Patient |
|  |  |  | Caregiver |

CIBIC PLUS—Baseline interview (continued)

| **Range** | **Tips** | | **Record** |
| --- | --- | --- | --- |
| **Mental / cognitive status (continued)** | | | |
| Practical ability | 45 Structural capacity  46 Ideation  47 Ability to mimic the action / imitation | Patient | |
|  |  | Caregiver | |
| Judgment /  Problem-solving skills /  Insight | 48 Behavior of patients in the environment requiring the use of judgment | Patient | |
|  |  | Caregiver | |
| **Behavior** | | | |
| Ideological content | 49 Organization  50 Appropriateness | | Patient |
|  |  |  | Caregiver |

CIBIC PLUS—Baseline interview (continued)

| **Range** | **Tips** | | **Record** |
| --- | --- | --- | --- |
| **Behavior (continued)** | | | |
| Hallucinations /  Delusion /  Illusion | 51 Audible / visual  52 Misperception  53 Systematic / developed | Patient | |
|  |  | Caregiver | |
| Behavior / mood | 54 Affective / emotional instability  55 Unusual / weird  56 Bohemian / inappropriate sexual behavior  57 Motivation / energy  58 Wandering / Lost  59 Excited / offensive  60 Hostility  61 Depression-related  62 Anxiety-related  63 Appropriateness  64 Cooperative  65 Demanding / dependence | Patient | |
|  |  | Caregiver | |
| Sleep / appetite | 66 Sleep Disorders  67 Insomnia (type?)  68 Nocturnal  69 Too much sleep - lack of sleep  70 Appetite / weight change | Patient | |
|  |  | Caregiver | |

CIBIC PLUS—Baseline interview (continued)

| **Range** | **Tips** | **Record** |
| --- | --- | --- |
| **Behavior (continued)** | | |
| Neurological / psychomotor activity | 71 Overall muscle activity  72 Posture / gait  73 Dyskinesia  74 Abnormal muscle activity  75 Daily behavior patterns  76 Trot | Patient |
|  |  | Caregiver |
| **Activities of daily living** | | |
| Basic and complex (appliance use) activities of daily living | 77 Ability to move  78 Hygiene / dress  79 Wearing  80 Feed themselves  81 Shopping  82 Homework / hobby  83 Money management  84 Driving / Cycling | Patient |
|  |  | Caregiver |
| Social skills | 85 Participate: social interaction  Community Activities  86 Independence  87 Powerless | Patient |
|  |  | Caregiver |

CIBIC PLUS—Baseline interview (continued)

| Record, review, summary statement: |
| --- |
| Information from other sources: |
| To complete this form information from the following sources are used :   1. Interviews with the patient / patient inspection 2. Interviews with caregiver, describe the relationship with the patient 3. Information about the neuropsychological test results 4. General information obtained from the medical staff meeting held targeting the patient 5. Other: |

CIBIC PLUS—Caregiver and patient interviews

| Research Project No.: | Case No.: | | Interview window: | Date of visit: | |
| --- | --- | --- | --- | --- | --- |
| Interview start time:  Brief Description: See description sheet. After the interview with both caregivers and patients, using this form to record information for CIBIC + assessment. Only refer to baseline evaluation form. A brief assessment of the mental state of the patient is required. No specific requirements on the form or the order of the interview. | | | | | |
| **Range** | | **Tips** | | | **Record** |
| Relevant medical history | | 93 Clinical events change and disease since the baseline interview | | | Patient |
|  |  |  |  |  | Caregiver |
| Observation / evaluation | | 94 Appearance | | | Patient |
|  |  |  |  |  | Caregiver |
| **Mental / cognitive status** | | | | | |
| Awakening /  Vigilance /  Attention / concentration level | | 95 Confusion / clarity  96 excitatory / reactivity  97 state of consciousness | | | Patient |
|  |  |  |  |  | Caregiver |

**CIBIC PLUS—Caregiver and patient interviews (continue**d)

| **Range** | **Tips** | **Record** | |
| --- | --- | --- | --- |
| **Mental / cognitive status (continued)** | | | |
| Orientation | 98 Time  99 Location  100 Character  101 Time relation  102 Travel  103 Reorientation  104 Identify yourself / others / objects  105 Reflect appropriate | | Patient |
|  |  |  | Caregiver |
| Memory | 106 Keep in mind  107 Recall  108 Long/far away  109 Memories of the past | | Patient |
|  |  |  | Caregiver |
| Language / speech | 110 Fluency degree/ expressive language, receptive language  111 Comprehension  112 heterophasia / difficulty finding the word  113 Name identification, the number of words  114 Repeat  115 Follow the instructions | | Patient |
|  |  |  | Caregiver |

CIBIC PLUS—Caregiver and patient interviews (continued)

| **Range** | **Tips** | **Record** |
| --- | --- | --- |
| **Mental / cognitive status (continued)** | | |
| Practical ability | 116 Structural capacity  117 Ideation  118 Ability to mimic the action / imitation | Patient |
|  |  | Caregiver |
| Judgment /  Problem-solving skills /  Insight | 119 Behavior of patients in the environment requiring the use of judgment | Patient |
|  |  | Caregiver |
| **Behavior** | | |
| Ideological content | 120 Organization  121 Appropriateness  122 Expressing hostility | Patient |
|  |  | Caregiver |

CIBIC PLUS—Caregiver and patient interviews (continued)

| **Range** | **Tips** | **Record** |
| --- | --- | --- |
| **Behavior (continued)** | | |
| Hallucinations /  Delusion /  Illusion | 123 Audible / visual  124 Misperception  125 Systematic / developed | Patient |
|  |  | Caregiver |
| Behavior / mood | 126 Affective / emotional instability  127 Unusual / weird  128 Bohemian / inappropriate sexual behavior  129 Motivation / energy  130 Wandering / Lost  131 Excited / offensive  132 Hostility  133 Depression-related  134 Anxiety-related  135 Appropriateness  136 Cooperative  137 Demanding / dependence | Patient |
|  |  | Caregiver |
| Sleep / appetite | 138 Sleep Disorders  139 Insomnia (type?)  140 Nocturnal  141 Too much sleep - lack of sleep  142 Appetite / weight change | Patient |
|  |  | Caregiver |

CIBIC PLUS—Caregiver and patient interviews (continued)

| **Range** | **Tips** | **Record** |
| --- | --- | --- |
| **Behavior (continued)** | | |
| Neurological / psychomotor activity | 143 Overall muscle activity  144 Posture / gait  145 Dyskinesia  146 Abnormal muscle activity  147 Daily behavior patterns  148 Trot | Patient |
|  |  | Caregiver |
| **Activities of daily living** | | |
| Basic and complex (appliance use) activities of daily living | 149 Ability to move  150 Hygiene / dress  151 Wearing  152 Feed themselves  153 Shopping  154 Homework / hobby  155 Money management  156 Driving / Cycling | Patient |
|  |  | Caregiver |
| Social skills | 157 Participate: social interaction  Community Activities  158 Independence  159 Powerless | Patient |
|  |  | Caregiver |

CIBIC PLUS—Caregiver and patient interviews (continued)

| Record, review, summary statement: |
| --- |
| Information from other sources: |

**CIBIC PLUS—Clinician impression on the cha**nges

| Research Project No.: | Case No.: | Interview window: | Date of visit: |
| --- | --- | --- | --- |
| To complete this form information from the following sources are used:  1. 🞏 Significant improvement  2. 🞏 Moderate improvement  3. 🞏 Slight improvement  4. 🞏 No change  5. 🞏 Slight deterioration  6. 🞏 Moderate deterioration  7. 🞏 Significant deterioration | | | |

## Appendix 6 Alzheimer's Disease Cooperative Study—Activities of Daily Living (ADCS-ADL)

Caregivers please answer patient’s performance in the last four weeks.

|  | Items | | | | | | | | | | Please check | | | | | | | | | | | | | | | | | | | | | | | | | | | | | | | |
| --- | --- | --- | --- | --- | --- | --- | --- | --- | --- | --- | --- | --- | --- | --- | --- | --- | --- | --- | --- | --- | --- | --- | --- | --- | --- | --- | --- | --- | --- | --- | --- | --- | --- | --- | --- | --- | --- | --- | --- | --- | --- | --- |
| 1 | Eating meals, which of the following is the best description | | | | | | | | | | 3 Do not need help, without any difficulties  2 Do not need help, with some difficulties  1 Do not need help, with difficulties  0 Patient needs help with feedings | | | | | | | | | | | | | | | | | | | | | | | | | | | | | | | □ |
| 2 | Walking (or moving on the wheel chair) which of the following is the best description | | | | | | | | | | 3 Do not need physical help. Able to walk outdoor independently  2 Do not need physical help. Able to walk indoor independently  1 Able to move from a bed to a chair  0 Need help with walking or moving | | | | | | | | | | | | | | | | | | | | | | | | | | | | | | | □ |
| 3 | Toilet capabilities, which of the following is the best description | | | | | | | | | | 3 Full toilet capabilities without any supervision or help  2 Need supervision but no need of physical help  1 Need physical help but able to perform toilet duties  0 Need physical and not able to perform toilet duties | | | | | | | | | | | | | | | | | | | | | | | | | | | | | | | □ |
| 4 | Taking a bath, which of the following is the best description | | | | | | | | | | 3 No need reminding or physical help to take a full bath  2 No need physical help, but need supervision/reminding to perform a full bath  1 Need a little physical help(e.g. washing the hair) to perform a full bath  0 Need full help | | | | | | | | | | | | | | | | | | | | | | | | | | | | | | | □ |
| 5 | **Personal Hygiene, w**hich of the following is the best description | | | | | | | | | | 3 No need physical help to perform nail care  2 No need physical help to perform hair care  1 No need help to perform hand washing  0 Need help to perform all of above activities | | | | | | | | | | | | | | | | | | | | | | | | | | | | | | | □ |
| 6 | Get dressed | | | | | | | | | | | | | | | | | | | | | | | | | | | | | | | | | | | | | | | | | |
| 6A | Does patient pick up a day coat himself/herself | | | | | | | | | | | Yes | | | | | | | | | No  0 | | | | | | | | | Unknown0 | | | | | | | | | | | | □ |
|  | If yes, please check | | 3 No need supervision or help  2 Need supervision  1 Need physical help | | | | | | | | | | | | | | | | | | | | | | | | | | | | | | | | | | | | | | |  |
| 6B | Which of the following is the best description of his/her daily performances | | 4 No need supervision or physical help to perform dressing himself/herself  3 Need supervision to perform self dressing but no need physical help  2Only need physical help when tightening buttons, zip, and shoe tie  1No need help if there is no buttons  0Always need help of self dressing | | | | | | | | | | | | | | | | | | | | | | | | | | | | | | | | | | | | | | | □ |
| 7 | Does patient use telephone? | | | | | | | | | | | | Yes | | | | | | | | | No  0 | | | | | | | | | Unknown  0 | | | | | | | | | | | □ |
|  | If yes, which of the following is the best description | | 5Able to find a number in the phone book or to call 411 to get the number.  4Only dial the familiar phone number when not looking at the phone book  3Only dial the familiar number when looking at he phone book  2 Able to pick up phone but not able to dial a phone  1Not able to pick up the phone, but able to answer the phone | | | | | | | | | | | | | | | | | | | | | | | | | | | | | | | | | | | | | | |  |
| 8 | Does patient watch TV?  If yes, answer the following questions | | | | | | | | | | | | | | | YES | | | | | | | No  0 | | | | | | | | | Unknown0 | | | | | | | | | | □ |
|  |  | | **a)** Does patient choose different channels or his/her favorites? | | | | | | | | | | | | | 1 | | | | | | | 0 | | | | | | | | | 0 | | | | | | | | | | □ |
|  |  | | **b)** Does patient make any comments on TV show when watching? | | | | | | | | | | | | | 1 | | | | | | | 0 | | | | | | | | | 0 | | | | | | | | | | □ |
|  |  | | **c)** Does Patient make any comments 24 hours after watching? | | | | | | | | | | | | | 1 | | | | | | | 0 | | | | | | | | | 0 | | | | | | | | | | □ |
| 9 | Does patient participate a conversation at least 5 minutes? Please note: patient does not need to initiate the conversation | | | | | | | | | | | | | | Yes | | | | | | | | No  0 | | | | | | | | | Unknown0 | | | | | | | | | □ | |
|  | If yes, which of the following is the best description | | | | | | 3 Normally relevant with the conversation  2 Normally not relevant with the conversation  1 Rarly saying anything | | | | | | | | | | | | | | | | | | | | | | | | | | | | | | | | | | | |
| 10 | Does patient clean up the table after meal? | | | | | | | | | | | | | Yes | | | | | | | | | | No  0 | | | | | | | | | Unknown0 | | | | | | | | | □ |
|  | **If yes, w**hich of the following is the best description | | | 3 No need supervision or help  2 Need supervision  1 Need physical help | | | | | | | | | | | | | | | | | | | | | | | | | | | | | | | | | | | | | |  |
| 11 | Is patient able to find his/her own personal items? | | | | | | | | | | | | | | | Yes | | | | | | | | | NO  0 | | | | | | | | | Unknown0 | | | | | | | □ | |
|  | **If yes, w**hich of the following is the best description， | | | 3 No need supervision or help  2 Need supervision  1 Need physical help | | | | | | | | | | | | | | | | | | | | | | | | | | | | | | | | | | | | |  |  |
| 12 | Does patient ever get hot/cold drinks for himself/herself? | | | | | | | | | | | | | | | | Yes | | | | | | | | | NO  0 | | | | | | | | | Unknown0 | | | | | | □ | |
|  | If yes, which of the following is the best description | | | | 3 Normally no need physical help to get hot drink  2 Normally can get hot drink if there is hot water  1Normally no need physical help to ger cold drink | | | | | | | | | | | | | | | | | | | | | | | | | | | | | | | | | | | |  |  |
| 13 | Does patient make his/her own meal? | | | | | | | | | | | | | | | | | Yes | | | | | | | | | No  0 | | | | | | | | | Unknown  0 | | | | | □ | |
|  | If yes, which of the following is the best description | | | | | 4 Almost no need help to cook food or heat food with microwave  3 need great help to cook food or heat food with microwave  2 Not able to cook or microwave food, able to mix different kinds of food to make a meal (i.e. sandwich)  1No mix or cook food, able to obtain food self | | | | | | | | | | | | | | | | | | | | | | | | | | | | | | | | | | |  |  |
| 14 | Does patient put home garbage or miscellaneous stuff in a garbage bin or a proper container? | | | | | | | | | | | | | | | | | Yes | | | | | | | | | No  0 | | | | | | | | | Unknown0 | | | | | □ | |
|  | If yes, which of the following is the best description | | | | | 3No need supervision or help  2Need supervision  1Need physical help | | | | | | | | | | | | | | | | | | | | | | | | | | | | | | | | | | |  |  |
| 15 | Does patient have outdoor activities? | | | | | | | | | | | | | | | | | | Yes | | | | | | | | | No  0 | | | | | | | | | Unknown0 | | | □ | | |
|  | If yes, which of the following is the best description | | | | | | | 4 Out door activities alone, at least 1 KM away from home  3 Out door activities alone, only within 1 KM away from home  2Out door activities with company or supervision only, no matter the distance away from home  1Out door activities only with physical help, no matter the distance away from home | | | | | | | | | | | | | | | | | | | | | | | | | | | | | | | |  |  |  |
| 16 | Does patient go shopping?  **If yes, answer A and B:** | | | | | | | | | | | | | | | | | | | Yes | | | | | | | | | No  0 | | | | | | | | | Unknown0 | □ | | | |
|  | **A）Which of following is the best description of the way of shopping** | | | | | | | | 3No need supervision or physical help  2Need some supervision or physical help  1Does know how to shop or pick up merchandiser improperly | | | | | | | | | | | | | | | | | | | | | | | | | | | | | | □ | | | |
|  | **B)** Does patient pay for the shopping without supervision or physical help? | | | | | | | | | | | | | | | | | | | Yes  1 | | | | | | | | | No  0 | | | | | | | | | Unknown0 | □ | | | |
| 17 | | Does patient memorize an appointment or a meeting with other people? (e.g. relatives, friends, physicians, or hairstylist, etc) | | | | | | | | | | | | | | | | | | Yes | | | | | | | | | No  0 | | | | | | | | | Unknown0 | □ | | | |
|  |  | If yes, which of the following is the best description | | | | | | | | 3Normally able to memorize, but need written notice  2Able to memorize only with oral reminding on same day  1Even with oral reminding, normally forgot the appointment | | | | | | | | | | | | | | | | | | | | | | | | | | | | |  |  |  |  |

|  | **Note:**If patient lives in a nursing home please skip question 18. Go directly to question 19. □ | | | | | | | | | | | | | | | |
| --- | --- | --- | --- | --- | --- | --- | --- | --- | --- | --- | --- | --- | --- | --- | --- | --- |
|  | 18 | | Has patient ever been left alone? (Exclude the time patient with family memebers)  If yes, answer the following questions | | | | | | | yes | | No  0 | | Unknown  0 | □ | |
|  |  |  |  | | | **a) Has** patient ever been left alone outside home more than 15 minutes during the day time? | | | | 1 | | 0 | | 0 | □ | |
|  |  |  |  | | | **b) Has** patient ever been left alone home more than one hour during the day time? | | | | 1 | | 0 | | 0 | □ | |
|  |  |  |  | | | **c) Has** patient ever been left alone home less than one hour during the day time? | | | | 1 | | 0 | | 0 | □ | |
|  | 19 | | Does patient discuss recent events? (Something happened during the past month)  If yes, answer the following questions | | | | | | | Yes | | No  0 | | Unknown0 | □ | |
|  |  |  |  | | | **a)** Is it something patient ever heard, read, or watched on TV, but not involved in personally | | | | 1 | | 0 | | 0 | □ | |
|  |  |  |  | | | **b) Is it something patient involved in out door with her/his family, friends, or neighbors?** | | | | 1 | | 0 | | 0 | □ | |
|  |  |  |  | | | **c)** Is it something patient involved in domestic affairs? | | | | 1 | | 0 | | 0 | □ | |
|  | 20 | | Does patient read written materials more than 5 minutes continuously ?If yes, answer the following questions | | | | | | | Yes | | No  0 | | Unknown  0 | □ | |
|  |  |  |  | | | **a)** Does patient discuss the reading details promptly, within one hour? | | | | 1 | | 0 | | 0 | □ | |
|  |  |  |  | | | **b)** Does patient discuss the reading details after one hour or even more? | | | | 1 | | 0 | | 0 | □ | |
|  | 21 | | Does patient ever compose an essay ?  Note :If patient only compose an essay after encouragement and help the answer is still‘Yes ‘ | | | | | | | Yes | | No  0 | | Unknown0 | □ | |
|  |  |  | If yes, which of the following is the best description of most complicated essay patient ever did. | | | 3A letter or a note other people can understand  2A simple note or words other people can understand  1His/her signature or name | | | | | | | | |  |  |
|  | 22 | | Does patient have any hobbies, favorite games, or vacations | | | | | | Yes | | No  0 | | Unknown  0 | | □ | |
|  |  |  | **If yes, which one(s) he/she ever had.**  **Note: Exclude walking in this scale table.**  **Please check all that apply** | | | | □ | A Chess games (including Majang, poker, chess)） | | | | | | |  |  |
|  |  |  |  |  |  |  | □ | B Ta Chi | | | | | | |  |  |
|  |  |  |  |  |  |  | □ | C Musical instruments | | | | | | |  |  |
|  |  |  |  |  |  |  | □ | D reading | | | | | | |  |  |
|  |  |  |  |  |  |  | □ | E Swimming | | | | | | |  |  |
|  |  |  |  |  |  |  | □ | F Guessing games | | | | | | |  |  |
|  |  |  |  |  |  |  | □ | G knitting | | | | | | |  |  |
|  |  |  |  |  |  |  | □ | H Gardening( planting flower, watering) | | | | | | |  |  |
|  |  |  |  |  |  |  | □ | I House repairing | | | | | | |  |  |
|  |  |  |  |  |  |  | □ | J Art (drawing, handwriting, dancing etc) | | | | | | |  |  |
|  |  |  |  |  |  |  | □ | K Cloth alteration | | | | | | |  |  |
|  |  |  |  |  |  |  | □ | L Cricket | | | | | | |  |  |
|  |  |  |  |  |  |  | □ | M Fishing | | | | | | |  |  |
|  |  |  |  |  |  |  | □ | N Others ____________________ | | | | | | |  |  |
|  |  |  | If yes, normally how does patient carrying out | | | | 3 No need supervision or help  2 Need supervision  1 Need help | | | | | | | | □ | |
|  | 22a | | If patient carries out above activities only under care, please check here | | | | | | | | | | | | □ | |
|  | 23 | | Does patient ever use electrical home appliance ? | | | | | | Yes | | No  0 | | Unknown0 | | □ | |
|  |  |  | If yes, please check which been used | | | | □ | A wash machines | | | | | | |  |  |
|  |  |  |  |  |  |  | □ | B Iron set | | | | | | |  |  |
|  |  |  |  |  |  |  | □ | C Gas oven | | | | | | |  |  |
|  |  |  |  |  |  |  | □ | D Mosquito repeller | | | | | | |  |  |
|  |  |  |  |  |  |  | □ | E Drinking water dispenser | | | | | | |  |  |
|  |  |  |  |  |  |  | □ | F Microwave | | | | | | |  |  |
|  |  |  |  |  |  |  | □ | G Vacuum machine | | | | | | |  |  |
|  |  |  |  |  |  |  | □ | H Electrical cooker | | | | | | |  |  |
|  |  |  |  |  |  |  | □ | I Food processors | | | | | | |  |  |
|  |  |  |  |  |  |  | □ | J Others____________________ | | | | | | |  |  |
|  |  |  | If yes, which of the following is the best description when patient operates a common appliance | | | | 4 No need help. If patient need to operate, not just the on/off switch  3 No need help, but only operate on/off switch  2 Need supervision, but no need physical help  1 Need physical help | | | | | | | | □ | |
| 24 | |  | |  | Total score (0-78) | | | | | | | | | | |  |
| 25 | |  | |  | Total times of answering “Unknown” | | | | | | | | | | |  |

## Appendix 7 Neuropsychiatric Inventory - clinician rating scale (NPI)

I. Neuropsychiatric Inventory (NPI)

The purpose of Neuropsychiatric Inventory (NPI) is to get the existing pathological information in the patients with brain dysfunction. NPI is developed to be used in patients with Alzheimer’s disease and other dementia, but also can be used to assess patient behavior change under other conditions. This inventory includes 10 behavior areas and two autonomic nervous system areas:

delusion

illusion

excited/offensive

depression

anxiety

high spirits/euphoria

cold/cool

derepression

irritability

abnormal behavior

Sleep and behavior disorders at night

Appetite and eating disorders

II. The usage of the Neuropsychiatric Inventory (NPI)

A. Neuropsychiatric Inventory (NPI) interview

NPI is based on answers from informed caregivers, and the caregiver is better the one to live together with patients. If there is no informed caregivers, the inventory cannot be used or can be used only after modification. Interview with caregivers better is in a time when the patient is not present, the purpose is to facilitate public discussion of those behavior inconvenient to describe when the patient is present. When you introduce to caregivers NPI interview, the following should be noted:

The purpose of the interview.

Assessment content - frequency, severity, and psychological pressure (described below).

Answer applies to those behavior emergent since the onset of and have been present in the past four weeks or for a period of time.

Usually can be answered using "yes" or "no", and answer should be brief.

When beginning to use inventory, say to caregivers: "these questions are designed to evaluate the behaviors of your husband/wife/etc. They usually can be answered using a 'yes' or' no ', so please try to answer briefly." If caregivers get into a detailed answer and answer content can hardly provide useful information, they can be reminded they need short answer. Some questions put forward in the process of interview might make caregivers feel emotionally disturbed, and interviewer should assure them after the end of the interview they will discuss these issues in detail.

Questions asked should be exactly the same as that of written. If caregivers don't understand the question, explanations should be provided, and retelling problems in alternative terms are acceptable.

B. behavior change

The questions are associated with the behavioural change of patients after illness. Behaviors that have been existing in the patient's life and have not changed in the process of illness, even abnormal (such as anxiety, depression) don’t score. Behaviors that have been present in the patient's life, but have changed in the process of the disease should score (such as the patient has always been very cold, in our survey there has been a significant increase in the cold degree).

NPI is usually used to assess change of behavior of patients in a specific period (e.g., over the past four weeks or some other time period) . In some studies, NPI is used to investigate changes due to treatment since the last time of treatment. It should be emphasized to the caregiver that these questions are related to the patient's performance or a change in behavior since the onset of the disease. For example, use such phrase to ask: "since he/she started with a new drug treatment..." or “since the _____ dose increase...”.

C. Screening questions

Screening questions are used to determine whether the patient is undergoing behavior change. If the answer to a screening question is "no", it is marked with "no", and subordinate questions included under the screening questions will not be asked any more, directly go to the next screening question. If the answer to a screening question is "yes" or caregivers’ answer has any uncertain component or answer does not match the information clinicians know(for example, caregivers’ answer to euphoria screening question is no, but patients’ manifestation in front of the clinician is euphoric), in the category label "yes", and further explore through the subordinate problems. If the content of the subordinate problems confirmed screening question, then judge the severity and frequency according to the standard provided with each behavior.

In some cases, the caregivers give positive answers to screening questions while giving negative answers to all of its subordinate questions. If this happens, caregivers are required to explain why they give positive answers to screening questions. If they provide information related to behavior and expression is different, it should be rated as usual to the severity and frequency of the behavior. If the first answer was a mistake so that it does not support any subordinate questions, change into “no” for the behavior on the screening question.

Some parts such as the questions related to the appetite are designed to capture behavior (appetite or weight increase or decrease) increase or decrease. If the caregiver gives “yes” to the former of a pair of questions (such as: is the patient's weight lost?) , you don't need to ask a second question again (is the patient's weight increased?). Because the answer to the first question already contains the answer to the second question. If caregivers give "no" to the former question, so be sure to ask the second question.

D. the frequency and severity rating

When determining the frequency and severity, behavior identified by the subordinate questions are rated as the most abnormal condition. For example, when you ask the subordinate questions in the exciting part, if caregivers say resistance behavior are particularly problematic, then use resistance behavior to hint evaluation to the excited frequency and severity. If there are two behaviors which are problematic, then the frequency and severity of the two behaviors are used to give a rating to this item. For example, if patient has two or more delusion, use the severity and frequency of all the delusional behavior (all types) to express these questions about the severity and frequency.

When judging the frequency, say to the people surveyed: "now I want to know the frequency of.these things (elaborate with description of behavior considered to be of the most problem in response to subordinate questions)" Can you tell me the frequency of them: less than once a week, about once a week, a few times a week but not every day, or once a day or more than once? "Certain behaviors, such as cold, will exist in a row, then use "almost continuous existence "instead of “once a day or more than once”.

When determining severity, tell interviewee: "now I want to know the severity of these behaviors. In terms of severity, I mean: how much bother these actions caused the patient or the degree of their disability. Would you please say [these behaviors] are mild, moderate, or severe?" In every part there is another description to help the interviewee to clarify the level of severity. In each case, make sure that caregivers give you definite answers on the frequency and severity of behavior. Don't guess what you think caregivers will say based on your discussion.

We found that will it helps to give caregivers a tips card. On this card there are the description of the frequency and severity (less than once a week, about once a week, a few times a week but not every day and once a day or more than once, or almost continuous to describe frequency; with mild, moderate and severe to describe severity), and allow them to visually see the optional answers. This also avoids for the testers to reiterate these options when asking every question.

E. markup is not applicable

In critically ill patients or patients in special medical condition, a set of questions may not apply. Bedridden patients may show the illusion, for example, or excited, but can't show the abnormal behavior. If clinicians or caregivers think these problems not suitable, then the parts should be marked as "not applicable" (in the top right-hand corner of the each part), also do not record more information for this part. Likewise, if the clinical doctors think the answer is invalid (for example: caregivers didn't seem to understand the set of questions asked), then this section should also be marked with "not applicable".

F. the autonomic nervous system changes

Item 11 (sleep) and 12 (eating) is added after the publishing of the Neuropsychiatric Inventory (NPI) (Cummings et al, 1994). The reason for adding is that they are the common problems areas in Alzheimer’s disease and other dementia. These items are part of depression syndrome of some patients, specially omit them from the irritable table of Neuropsychiatric Inventory (NPI) with the purpose to make the table focus on the mood symptoms. Both of these symptoms are usually not included in the Neuropsychiatric Inventory (NPI) total score nor are included in all study protocols.

G. caregivers’ psychological pressure (NPI - D)

When every field has been completed and caregivers completed the assessment of the frequency and severity of activities, if your protocol includes the psychological stress evaluation, then ask that question of caregivers psychological pressure related to it. To do this, must ask caregivers with a 5 points rating: 0 - no, 1 - slight, 2 - mild, 3 - moderate, 4 - serious, 5 - very serious or extremely serious. The psychological stress scale in this tool is developed by MD Daniel Kaufer (Daniel Kaufer).

III. the NPI score

Frequency scoring criteria:

1 - very few - less than once a week

2 - sometimes - about once a week

3 - often - a few times a week but not every day

4 - very frequently - once or more a day, or almost appear in succession

Severity scoring criteria:

1 - mild - rarely create psychological pressure to the patient

2 - moderate, cause much worry to patients, but can be distracted by caregivers through other activities

3 - serious, cause extreme worry to patients, and difficult to be distracted by other activities

Score of each field is: field score = frequency x severity

Psychological stress scoring criteria

0 - no

1 - slight

2 - mild

3 - moderate

4 - serious

5 - very serious or extremely serious

Therefore, there are four scores in each behavior field:

frequency

severity

total score (frequency x severity)

caregivers psychological pressure

NPI total score can be calculated by adding scores of all the 10 behavior fields. In most cases, Neuropsychiatric Inventory (NPI) total score does not include the autonomic nervous system item. In case of being included, it should be explained that a 12 points instead of 10 points is used. Mental pressure points are not included in the Neuropsychiatric Inventory (NPI) total score. The psychological pressure total score is created through the summary of scores of the first 10 or all 12 NPI psychological stress questions; be sure to specify it is 10 or 12 points that is adopted.

IV. Neuropsychiatric Inventory - nursing home version (NPI - NH) and Neuropsychiatric Inventory - Q version (NPI - Q)

Neuropsychiatric Inventory - nursing home version (NPI - NH) is developed to be used by the professional caregivers in the organization. This tool is the same as the original Neuropsychiatric Inventory (NPI) , just the format of question is changed, to adapt to the situation that professional caregivers don't know the patient before the patient is ill, and therefore could not know whether the patient's current behavior is on behalf of the change on the basis of behavior before the onset of disease. Caregivers psychological stress questions have been rephrased, to evaluate the "function interference" caused by various behaviors.

Neuropsychiatric Inventory - Q version (NPI - Q) is developed with standard Neuropsychiatric Inventory (NPI) and under cross validation, so as to provide a brief evaluation of the neuropsychological symptoms in the clinical environment.

Neuropsychiatric Inventory - nursing home version (NPI - NH) and Neuropsychiatric Inventory - Q version (NPI - Q) are provided by the University of California, Los Angeles, Alzheimer’s disease center, reed neurological research center. Address: 710 Westwood Plaza, Los Angeles, Califomia, 90095-1769.

V. usage video tape

Usage video tape (English) is provided by the University of California, Los Angeles, Alzheimer’s disease center, reed neurological research center. Address: 710 Westwood Plaza, Los Angeles, Califomia, 90095-1769. Each video tape costs $25 (will adjust according to actual situation). If these tools are used for research purposes, these tapes were strongly recommended training users in order to obtain consistency in using Neuropsychiatric Inventory (NPI) and Neuropsychiatric Inventory - nursing home edition (NPI - NH).

VI. Translation

Neuropsychiatric Inventory (NPI) has various language version for the use in Asia, Europe and America. Now more language versions are under development. Please use the address provided in the part VIII to contact with Dr Cummings consulting about language versions. All translation are completed through translation and reverse translation by bilingual clinicians - scientists with the version language as their first language. When providing translation version please also indicate the translator for contact.

VII. The electronic version

Neuropsychiatric Inventory (NPI), Neuropsychiatric Inventory - nursing home version (NPI - NH) and Neuropsychiatric Inventory - Q version (NPI - Q) have cd-rom version of apple computer format (no electronic score or usage version), and address provided in the part VIII below can be used to contact with Dr Cummings to ask for.

VIII. The copyright and use

Copyrights of Neuropsychiatric Inventory (NPI), Neuropsychiatric Inventory - nursing home version (NPI - NH) and Neuropsychiatric Inventory - Q version (NPI - Q) and the translation version and the derived version belong to Dr. Jeffrey l. Cummings. Free for all non-commercial research and clinical use. The Neuropsychiatric Inventory (NPI), Neuropsychiatric Inventory - nursing home version (NPI - NH) and Neuropsychiatric Inventory - Q version (NPI - Q) used for commercial purposes (including clinical trials, commercial project screening, for-profit health care service application, etc.) have to pay, and to use the tool you must negotiate with Dr Cummings. Address: University of California, Los Angeles, alzheimer's disease center, reed neurological research center, 710 Westwood Plaza, Califomia, Los Angeles, USA, 90095-1769 (telephone 310/206-5287, E-mail: jcummings@mednet.ucla.edu).

All papers and abstracts using Neuropsychiatric Inventory (NPI), Neuropsychiatric Inventory - nursing home version (NPI - NH) and Neuropsychiatric Inventory - Q version (NPI - Q) must be provided to Dr Cummings through the above address, in order to set up a comprehensive reference list for the studies using these tools and the survey people.

Reference

Cumming JL, Mega M, Gray K, Rosenberg-Thompson S, Carusi DA, Gornbein J. The Neuropsychiatric Inventory: comprehensive assessment of psychopathology in dementia. Neurology 1994;44:2308-2314.

Cummings JL. The Neuropsychiatric Inventory: Assessing psychopathology in dementia patients. Neurology 1997;48(Supp1.6):S10-S16.

Kaufer DI, Cummings JL, Christine D, Bray T, Castellon S, Masterman D, MacMillan A, Ketchel P, Dekosky ST. Assessing the impact of neuropsychiatric symptoms in Alzheimer’s disease: the Neuropsychiatric Inventory Caregiver Distress Scale. J Am Geriatric Soc 1998; 46:210-215.

Wood S, Cummings JL, Hsu M-A, Barclay T, Wheatley MV, Yarema KT, Schnelle JF. The use of the Neuropsychiatric Inventory in nursing home residents: characterization and measurement. Am J Geriatr Psychiatry 1999; 8:75-83.

Acknowledgements：UCLA Alzheimer’s Disease Center, Academic Geriatric Resource Program, UCLA Center on Aging and the Irving and Helga Cooper Geriatric Research Award; Sidell-Kagan Research Foundation.

**A. Delusion (NA)**

Dose the patient have some untrue beliefs you know? For example: He / she is convinced that someone intends to hurt him / her or to steal his / her stuff. Did he / she say that family members are not claiming their true identity or the house is not their home? What I want to ask are not things purely skeptical, but what I'm interested in is whether the patient is convinced that these things occur to him / her.

No (If the answer is “no”, go to the next item for the next screening question).

Yes (If the answer is “yes”, go on to ask the subordinate questions below the item).

1. Whether the patient thinks he / she is in danger - the other person intends to harm him / her? ________
2. Whether the patient thinks someone is trying to steal his / her stuff? ________
3. Whether the patient thinks his / her spouse is having an affair? _______
4. Whether the patient thinks unwelcome guests are staying in his / her home? ________
5. Whether the patient thinks his / her spouse or other person is not the person they claim? ________
6. Whether the patient thinks his / her house is not his / her home? ________
7. Whether the patient thinks the family members intend to abandon him / her? ________
8. Whether the patient thinks the characters on the television or magazine are actually at home? ________

[Does he / she not try to talk to or communicate with them?]

1. Are there any other unusual things he / she believes I have not asked? ________

If the screening questions are confirmed, the frequency and severity of delusion can be determined.

Frequency:

1. Rarely - less than once a week
2. Sometimes - about once a week
3. Regularly - several times a week but not every day
4. Very frequently - once or more times daily

Severity:

1. Mild - Delusion was present, but seems harmless and rarely brings psychological stress to the patient.
2. Moderate - Delusion brings psychological stress and annoyance.
3. Severe - Delusion brings extreme annoyance and is a major source of annoyance behavior. [If a doctor to prescribe drugs as needed (PRN), the administration of these drugs signals that delusion achieves significant severity.]

Psychological stress: Do you think how much stress has this behavior placed on your mood?

1. No
2. Slight
3. Mild
4. Moderate
5. Severe
6. Very severe or extremely severe

B. Illusion (NA)

Does the patient have illusions, such as unreal sight or sound? Does he / she seem to have seen, heard or experienced some things that do not exist? On this issue, we not only refer to some false beliefs, such as claiming that a dead person is still alive; but rather are asking whether the patient actually had the experiences like a sound or sight.

No (If the answer is “no”, go to the next item for the next screening question).

Yes (If the answer is “yes”, go on to ask the subordinate questions below the item).

1. Will the patient describe he / she heard some sound or behaved as he / she had heard some sounds?
2. Will the patient speak to the person who is not in front of him / her?
3. Will the patient describe he / she saw things others do not see or behaved as he / she saw things other do not see (people, animals, light, etc.)?
4. Does the patient say he / she smelled the odor others do not smell?
5. Will the patient describe he / she felt there was something on his / her skin or seemed to feel there was something crawling into or touching his / her body?
6. Will the patient describe he / she tasted some unprovoked taste?
7. Will the patient describe any other abnormal sensations and experiences?

If the screening questions are confirmed, the frequency and severity of illusion can be determined.

Frequency:

1. Rarely - less than once a week
2. Sometimes - about once a week
3. Regularly - several times a week but not every day
4. Very frequently - once or more times daily

Severity:

1. Mild - Illusion was present, but seems harmless and rarely brings psychological stress to the patient.
2. Moderate - Illusion brings psychological stress and annoyance.
3. Severe - Illusion brings extreme annoyance and is a major source of annoyance behavior.

It may be controlled by administering the as needed (PRN) class of drugs.

Psychological stress: Do you think how much stress has this behavior placed on your mood?

1. No
2. Slight
3. Mild
4. Moderate
5. Severe
6. Very severe or extremely severe

**C. Agitation / Aggression (NA)**

Will the patient have some periods during which he / she refuses to cooperate or refuses other’s help? Is he / she very difficult to cope with such conditions?

No (If the answer is “no”, go to the next item for the next screening question).

Yes (If the answer is “yes”, go on to ask the subordinate questions below the item).

1. Will the patient feel uneasy for those who want to try to take care of him / her, or resist some activities such as bathing or changing clothes?
2. Is the patient stubborn at his / her own way of acting?
3. Does the patient refuse to cooperate or refuse other’s help?
4. Does the patient have some other behaviors so that others find it difficult to cope with him / her?
5. Will the patient make a noise or make a curse angrily?
6. Does the patient have the behaviors of closing the door violently, kicking the furniture and throwing things?
7. Does the patient have the behavior of attempting to harm or hit someone else?
8. Does the patient have other aggressive or agitated behaviors?

If the screening questions are confirmed, the frequency and severity of agitation / aggression can be determined.

Frequency:

1. Rarely - less than once a week
2. Sometimes - about once a week
3. Regularly - several times a week but not every day
4. Very frequently - once or more times daily

Severity:

1. Mild - Behavior may bring annoyance, which can be controlled by diverting attention and consoling.
2. Moderate - Behavior may bring annoyance, which is difficult to be diverted and controlled.
3. Severe - Agitation brings extreme annoyance and is a major source of annoyance behavior. It may cause personal injury, and usually requires medication.

Psychological stress: Do you think how much stress has this behavior placed on your mood?

1. No
2. Slight
3. Mild
4. Moderate
5. Severe
6. Very severe or extremely severe

D. Depression (NA)

Does the patient look sad or depressed? Did he / she say he / she felt sad or depressed?

No (If the answer is “no”, go to the next item for the next screening question).

Yes (If the answer is “yes”, go on to ask the subordinate questions below the item).

1. Does the patient have tears or sob when he / she seems sad?
2. Does the patient appear in speech or behavior that he / she seems unhappy or depressed?
3. Does the patient belittle him / herself or feel that he / she is just like a loser?
4. Does the patient say that he / she is a bad person or should be punished?
5. Does the patient seem discouraged or say that his / her future is uncertain?
6. Does the patient say that he / she is a burden to the family or it will be better if he / she is not at home?
7. Have the patient expressed his desire to die or talked about suicide?
8. Did the patient have any other signs of depression or sadness?

If the screening questions are confirmed, the frequency and severity of depression can be determined.

Frequency:

1. Rarely - less than once a week
2. Sometimes - about once a week
3. Regularly - several times a week but not every day
4. Very frequently - almost consecutively

Severity:

1. Mild - Depression may bring annoyance, which can be controlled by diverting attention and consoling.
2. Moderate - Depression brings annoyance so that patient is present with symptoms of depression, which are difficult to alleviate.
3. Severe - Depression brings extreme annoyance and is a major source of suffering for the patient.

Psychological stress: Do you think how much stress has this behavior placed on your mood?

1. No
2. Slight
3. Mild
4. Moderate
5. Severe
6. Very severe or extremely severe

E. Anxiety (NA)

Does the patient appear very nervous, worried, or scared for no apparent reasons? Does he / she look very nervous or upset? Is the patient afraid that you are not around him / her?

No (If the answer is “no”, go to the next item for the next screening question).

Yes (If the answer is “yes”, go on to ask the subordinate questions below the item).

1. Does the patient say he / she is worried about planned things?
2. Did the patient have the feeling of be trembling, uneasy, or particularly stressful?
3. Did the patient have (or complain of) shortness of breath, wheezing, or sigh for no other apparent reasons except tension?
4. Will the patient complain of psychological upset (some nausea) or have rapid heart beats because of tension? [The symptoms are not caused by poor health.]
5. Will the patient evade from some locations or conditions making him / her more nervous, such as going by car, meeting friends, or staying in the crowd?
6. Will the patient become nervous and upset when he / she is separated from you (or his / her caregiver)?
7. Did the patient show any signs of anxiety?

If the screening questions are confirmed, the frequency and severity of anxiety can be determined.

Frequency:

1. Rarely - less than once a week
2. Sometimes - about once a week
3. Regularly - several times a week but not every day
4. Very frequently - once or more times daily24

Severity:

1. Mild - Anxiety may bring annoyance, which can be controlled by diverting attention and consoling.
2. Moderate - Anxiety brings annoyance so that patient is present with symptoms of depression, which are difficult to alleviate.
3. Severe - Anxiety brings extreme annoyance and is a major source of suffering for the patient.

Psychological stress: Do you think how much stress has this behavior placed on your mood?

1. No
2. Slight
3. Mild
4. Moderate
5. Severe
6. Very severe or extremely severe

F. Elation / Excitement (NA)

Does the patient seem too happy or too excited for no reasons? I do not mean that happiness comes from normal routes when the patient sees a friend, receives a gift, or stays with his / her family members. I mean whether the patient has persistently and abnormally good mood, or will feel things are humorous while other people do not feel.

No (If the answer is “no”, go to the next item for the next screening question).

Yes (If the answer is “yes”, go on to ask the subordinate questions below the item).

1. Does the patient feel too good or too happy and behave not same as his / her own usual performance?
2. Will the patient feel some other people do not find things interesting or funny humorous?
3. Does the patient seem to have a childish sense of humor, and not tend to giggle or laugh properly (for example when he / she saw unfortunate things happened to someone)?
4. Is the patient telling jokes or saying some words that other people think not humorous, but he / she feels it funny?
5. Will the patient play some childish tricks for fun, such as pinching others or playing “hide and seek”?
6. Will the patient “brag” or falsely exaggerate his / her ability or wealth?
7. Does the patient have the signs of feeling too good or too happy?

If the screening questions are confirmed, the frequency and severity of elation / excitement can be determined.

Frequency:

1. Rarely - less than once a week
2. Sometimes - about once a week
3. Regularly - several times a week but not every day
4. Very frequently - almost consecutively

Severity:

1. Mild - Elated behavior is obvious for his / her friends and family members, but brings annoyance.
2. Moderate - Elated behavior is significantly abnormal.
3. Severe - Elated behavior is very obvious. The patient has excited performance and almost feels everything is very funny.

Psychological stress: Do you think how much stress has this behavior placed on your mood?

1. No
2. Slight
3. Mild
4. Moderate
5. Severe
6. Very severe or extremely severe

G. Apathy / Indifference (NA)

Doe the patient lose interest in the world around him / her? Does he / she lose interest in doing things, or lack of motivation for launching new activities? Is he / she more difficult to participate in the exchange or do housework? Does the patient show indifference or apathy?

No (If the answer is “no”, go to the next item for the next screening question).

Yes (If the answer is “yes”, go on to ask the subordinate questions below the item).

1. Does the patient seem to have less self-motivation and less activity than usual?
2. Is the patient unlikely to take the initiative to talk to other people?
3. Does the patient lack of some warmth or affection as compared with his / her own usual behavior?
4. Does the patient do less housework than the normal conditions?
5. Does the patient seem to be less interested in someone else’s activities or plans?
6. Is the patient no longer concerned about his / her friends or family members?
7. Is the patient no longer so enthusiastic about the things he / she is usually interested in?
8. Does the patient have other signs of being not interested in doing new things?

If the screening questions are confirmed, the frequency and severity of apathy / indifference can be determined.

Frequency: 1. rarely - less than once a week

2. Sometimes - about once a week

3. Regularly - several times a week but not every day

4. Very frequently - almost consecutively

Severity:

1. Mild - Indifference is evident, but rarely affects daily life. The patient’s present behavior is slightly different from his / her usual behavior. The patient will respond to the recommendation of participation in activities.

2. Moderate - Indifference is very evident, which can be overcome by the coaxing and encouragement from the caregiver. The patient has only a spontaneous reaction to major events such as visitation by the close relatives or family members.

3. Severe - Indifference is very evident. The patient usually has no reaction to any encouragement or outside things.

Psychological stress: Do you think how much stress has this behavior placed on your mood?

1. No
2. Slight
3. Mild
4. Moderate
5. Severe
6. Very severe or extremely severe

H. Disinhibition (NA)

Does the patient seem to have impulsive behaviors without thinking? Will he / she do or say something he / she does not do or say usually in public? Will he / she do something that makes you or someone else embarrassing?

No (If the answer is “no”, go to the next item for the next screening question).

Yes (If the answer is “yes”, go on to ask the subordinate questions below the item).

1. Does the patient seem to have impulsive behaviors without considering the consequences?
2. Does the patient talk to some complete strangers as if he / she knows them?
3. Does the patient say some words without taking into account the feelings of others or some words hurting their feelings?
4. Does the patient speak foul language he / she does not do usually or talk about sex-related topics?
5. Does the patient talk openly about some issues that are usually inconvenient to be discussed in public places and are of highly personal privacy?
6. Does the patient have a disadvantageous behavior, or touch or hug someone else in a manner inconsistent with his / her personality?
7. Does the patient have the signs of other impulsiveness and lose of control?

If the screening questions are confirmed, the frequency and severity of disinhibition can be determined.

Frequency:

1. rarely - less than once a week

2. Sometimes - about once a week

3. Regularly - several times a week but not every day

4. Very frequently - almost consecutively

Severity:

1. Mild - Disinhibition is evident, but diverting attention and guidance will produce results.

2. Moderate - Disinhibition is very evident and is difficult to be overcome by the caregiver.

3. Severe - Any interference by the caregiver is to no avail for disinhibitory behavior, which has become the root cause of embarrassment or social distress.

Psychological stress: Do you think how much stress has this behavior placed on your mood?

1. No
2. Slight
3. Mild
4. Moderate
5. Severe
6. Very severe or extremely severe

I. Irritability (NA)

Does the patient have the performances of being irritable and easily annoy? Is his / her mood variable? Will he / she be extremely impatient? We do not mean the patient is frustrated by amnesia or inability to complete doing the usual things. What we are interested in is whether the patient has abnormal performances of irritability, impatience, or rapid mood changes that are different from his / her usual behavior?

No (If the answer is “no”, go to the next item for the next screening question).

Yes (If the answer is “yes”, go on to ask the subordinate questions below the item).

1. Is the patient ill-tempered and easy to get angry at trivia?
2. Is the patient’s mood changing rapidly and volatile. Is he / she may in good mood at this moment and getting angry after a while?
3. Will the patient get angry suddenly and unexpectedly?
4. Does the patient lack of patience, and have difficulty in coping with time delays or waiting for planned activities?
5. Is the patient grumpy and irritable?
6. Is the patient argumentative and difficult to get along with?
7. Does the patient have other signs of irritability?

If the screening questions are confirmed, the frequency and severity of irritability / volatility can be determined.

- - - 1. Rarely - less than once a week
      2. Sometimes - about once a week
      3. Regularly - several times a week but not every day
      4. Very frequently - almost consecutively

Severity: 1. Mild - Irritability / volatility is evident. But the patient usually responds to diverting attention or consoling.

2. Moderate - Irritability / volatility is very evident and is difficult to be overcome by the caregiver.

3. Severe - Irritability / volatility is very evident. Any interference by the caregiver is to no avail for irritable / volatile behavior, which has become the root cause of psychological stress.

Psychological stress: Do you think how much stress has this behavior placed on your mood?

1. No
2. Slight
3. Mild
4. Moderate
5. Severe
6. Very severe or extremely severe

J. Abnormal action / behavior (NA)

Will the patient pace about and do something repeatedly, such as open the cabinet or drawer, or repeatedly pick something, or repeatedly wind the string or wire?

No (If the answer is “no”, go to the next item for the next screening question).

Yes (If the answer is “yes”, go on to ask the subordinate questions below the item).

1. Does the patient pace about in the house aimlessly?
2. Is the patient rummaging and searching around?
3. Does the patient repeatedly put on and put off his / her clothes?
4. Does the patient have some activities or “habits” that he / she will repeat over and over again?
5. Does the patient focus his / her attention on repeated actions, such as buttoning, grasping and picking up something like thin thread or winding the rope, and so on?
6. Does the patient appear excessively restless, look like not being able to sit quietly, or frequently raise feet, or tap with fingers?
7. Does the patient repeat doing other things over and over again?

If the screening questions are confirmed, the frequency and severity of abnormal action / behavior can be determined.

- - - 1. Rarely - less than once a week
      2. Sometimes - about once a week
      3. Regularly - several times a week but not every day
      4. Very frequently - almost consecutively

Severity: 1. Mild - Abnormal action / behavior is evident, but almost does not interfere with daily life.

2. Moderate - Abnormal action / behavior is very evident and is difficult to be overcome by the caregiver.

3. Severe - Abnormal action / behavior is very evident. Any interference by the caregiver is to no avail for abnormal action / behavior, which has become the root cause of psychological stress.

Psychological stress: Do you think how much stress has this behavior placed on your mood?

1. No
2. Slight
3. Mild
4. Moderate
5. Severe
6. Very severe or extremely severe

K. Nighttime sleep and behavioral disorders (NA)

Is the patient unable to sleep (except that the patient only gets off from bed to the toilet once or twice and then gets to sleep at night)? Will he \ she get up at night? Will he \ she walk at night or disturb your sleep?

No (If the answer is “no”, go to the next item for the next screening question).

Yes (If the answer is “yes”, go on to ask the subordinate questions below the item).

1. Does the patient have difficulty falling asleep?
2. Will the patient get up at night (except that the patient only gets off from bed to the toilet once or twice and then gets to sleep at night)?
3. Will the patient walk at night, pace about, or engage in inappropriate activities?
4. Will the patient wake you up at night?
5. Will the patient wake up, dress himself / herself and intend to go out at night because he / she mistakenly thinks it was in the morning and at the beginning of the day?
6. Does the patient wake up too early in the morning (earlier than his / her previous habitual waking time)?
7. Will the patient sleep too much at daytime?
8. Does the patient have other nocturnal behaviors that make you worry and we do not talk about?

If the screening questions are confirmed, the frequency and severity of nocturnal behavior disturbance can be determined.

- - - 1. Rarely - less than once a week
      2. Sometimes - about once a week
      3. Regularly - several times a week but not every day
      4. Very frequently - once or more times daily (every night)

Severity: 1. Mild - Nocturnal behaviors occur and will not bring disturbance.

2. Moderate - Nocturnal behaviors occur and disturb the sleep of the patient and the caregiver.

3. Severe - Nocturnal behaviors occur and may have some different forms. The patient is very distressed at night and the caregiver’s sleep is severely disturbed.

Psychological stress: Do you think how much stress has this behavior placed on your mood?

1. No
2. Slight
3. Mild
4. Moderate
5. Severe
6. Very severe or extremely severe

L. Appetite and eating disorders (NA)

Does he / she have any changes in appetite, weight and eating habits (if the patient is unable to take care of himself / herself and needs to be fed by other people, it will fall under the inapplicable class)? Are there any changes in his / her favorite types of food?

No (If the answer is “no”, go to the next item for the next screening question).

Yes (If the answer is “yes”, go on to ask the subordinate questions below the item).

1. Does his / her appetite decrease?
2. Does his / her appetite increase?
3. Does his / her weight decrease?
4. Does his / her weight increase?
5. Are there any changes in his / her eating behavior? For example, he / she puts too much food into his mouth at once.
6. Are there any changes in his / her favorite types of food? For example, he / she eats too much candy or some other food.
7. Does he / she make the habits of eating exactly the same food or eating in a constant order every day?
8. Are there any change in other appetite or eating aspects of the patient we have not talked about?

If the screening questions are confirmed, the frequency and severity of appetite and eating disorders can be determined.

- - - 1. Frequency: 1. Rarely - less than once a week
      2. Sometimes - about once a week
      3. Regularly - several times a week but not every day
      4. Very frequently - once or more times daily or almost consecutively

Severity: 1. Mild - Appetite and eating disorders have some changes, but do not result in changes in body weight and bring disturbances.

2. Moderate - Appetite and eating disorders have some changes and result in slight fluctuations of body weight.

3. Severe - Appetite and eating disorders have significant changes and result in fluctuations of body weight, which makes people embarrassing or disturbs the patient.

Psychological stress: Do you think how much stress has this behavior placed on your mood?

1. No
2. Slight
3. Mild
4. Moderate
5. Severe
6. Very severe or extremely severe

## Summary

| Symptom | Y/N | Frequency | Severity | Frequency*Severity | Psychological stress |
| --- | --- | --- | --- | --- | --- |
| A. Delusion | □ □ |  |  |  |  |
| B. Illusion | □ □ |  |  |  |  |
| C. Excited/offensive | □ □ |  |  |  |  |
| D. Depression | □ □ |  |  |  |  |
| E. Anxiety | □ □ |  |  |  |  |
| F.High spirits/euphoria | □ □ |  |  |  |  |
| G.Cold | □ □ |  |  |  |  |
| H.Derepression | □ □ |  |  |  |  |
| I.Irritability | □ □ |  |  |  |  |
| J.Abnormal behavior | □ □ |  |  |  |  |
| K. Sleep and behavior disorders | □ □ |  |  |  |  |
| L. Appetite disorders | □ □ |  |  |  |  |
| Total Score |  | | |  |  |

Rater: Date:
