# Supplementary material for: A phase II randomized trial of sodium oligomannate in Alzheimer’s dementia
Source: Alzheimers Res Ther. 2020 Sep 14;12:110. doi: 10.1186/s13195-020-00678-3 (PMC7489025; doi:10.1186/s13195-020-00678-3)
Supplement: Supplementary file 2 — Additional file 2. [file 13195_2020_678_MOESM2_ESM.doc]

**A Multi-Center, Randomized, Double-Blind, Placebo Parallel Controlled, Dose-Finding Phase II Clinical Trial to Evaluate the Efficacy and Safety of** **Sodium Oligomannate Capsules (971) in Treatment of Mild to Moderate Alzheimer’s Disease**

**-**

**Safety Report**

| **Document category:** | **Safety Report** |
| --- | --- |
| **Study phase:** | **Phase II** |
| **Principal Investigator:** | **Professor Xiao Shifu** |

Confidential

This document shall not be used, cited, published or disclosed by any means without the consent of Shanghai Green Valley Pharmaceutical Co., Ltd.

**Safety Report**

| **Study title:** | A Multi-Center, Randomized, Double-Blind, Placebo Parallel Controlled, Dose-Finding Phase II Clinical Trial to Evaluate the Efficacy and Safety of Sodium Oligomannate Capsules (971) in Treatment of Mild to Moderate Alzheimer’s Disease | | |
| --- | --- | --- | --- |
| **Study drug:** | Sodium Oligomannate capsule (971 capsule) | | |
| **Clinical trial approval document number:** | 2006L02492, 2011L00942 | | |
| **Study design:** | Multi-center, randomized double-blind, placebo-controlled, parallel-group, dose-finding phase | | |
| **Study phase:** | Phase II | | |
| **Drug registration applicant:** | Shanghai Green Valley Pharmaceutical Co., Ltd. (affix seal) | | |
| **Enrollment start date:** | 10-24-2011 (enrollment date of the first subject) | | |
| **Withdrawal end date:** | 07-10-2013 (withdrawal date of the last subject) | | |
| **Principal investigator:** | Professor Xiao Shifu | Signature: __________  Date: __________ | |
| **Main study site:** | Mental Health Center Affiliated with Shanghai Jiao Tong University School of Medicine  (Shanghai Mental Health Center) (affix seal) | | |
| **Contact person of the reporting unit:** | Xin Xianliang | Contact number: |  |
| **Statistics leader:** | Liang Suping | Signature: __________  Date: __________ | |
| **Statistics unit:** | GCP ClinPlus Co., Ltd. (affix seal) | | |
|  | (Source: Beijing DMS Pharma Ltd.) | | |
| **Storage location of raw data:** | National drug clinical trial institution archives of all centers. | | |
| **Report date:** | 2013-11-02 | | |

**Study Report** Summary

| **Drug registration applicant** | Shanghai Green Valley Pharmaceutical Co., Ltd. |
| --- | --- |
| **Name of study drug** | Sodium Oligomannate capsule (971 capsule) |
| **Study title**: A Multi-center, Randomized Double-blind, Placebo-controlled, Parallel-group, Dose-finding Phase II Clinical Trial on the Efficacy and Safety of Sodium Oligomannate Capsule (971 Capsule) in the Treatment of Mild to Moderate Alzheimer's Disease | |
| **Study staff**: Xiao Shifu, Chen Yangmei, Tang Mouni, Zhang Liming, Gao Xiaoping, Li Hailin, Li Wei, Kuang Weihong, Cheng Yan, Xiao Jinsong, Chen Haibo, Chen Wei, Li Yingjie, Zhou Huang, Shi Shugui, Li Honghua, He Zhiyi, Peng Ying, Du Yifeng, Lin Hong, Wang Baojun, Xu Wenwei, Bi Jianzhong, Yan Chuanzhu, Lang Suping, Sun Haiquan, Peng Yanfen, Wu Ting | |
| **Study dates**: Enrollment start date: 10-24-2011  Withdrawal end date: 07-10-2013 | |
| **Study objectives**:  **Primary objective:** To investigate the optimal therapeutic dose and perform an initial evaluation of the efficacy and safety of Sodium Oligomannate capsule (971 capsule) in the treatment of mild to moderate Alzheimer's disease.  **Secondary objectives:**  1．To evaluate the effect of Sodium Oligomannate capsule on the cerebrospinal fluid biomarkers (Aβ_40_, Aβ_42_, T-tau, P-tau);  2．To determine the concentrations of Sodium Oligomannate in the cerebrospinal fluid and blood after reaching steady state. | |
| **Study method**: Multi-center, randomized double-blind, placebo-controlled, parallel-group, dose-finding. | |
| **Number of subjects**: The planned enrollment was 252 subjects, and the actual enrollment was 255 subjects, with 85, 84 and 86 subjects in the placebo group, 600mg group and 900mg group, respectively. | |
| **Inclusion and exclusion criteria**  **Inclusion criteria**  1. Aged 50-85 years, male or female;  2．Primary school and higher education level;  3．Meets the diagnostic criteria of possible Alzheimer's disease (AD) in the U.S. National Institute of Neurological and Communicative Diseases and Stroke-Alzheimer's Disease and Related Disorders Association (NINCDS-ADRDA) (1984);  4．Patient with mild to moderate condition, that is, 10 ≤Total Mini-Mental State Examination (MMSE) score ≤24;  5．Total score on the Hachinski Ischemic Scale (HIS) <4 points;  6．Total score on the Hamilton Depression Scale/17-item version ≤10 points;  7．The subject should have a stable and reliable caregiver, or can at least have frequent contact with the caregiver (at least 4 days in a week, at least 2 hours per day), and the caregiver will assist the patient in participating in the entire study process.The caregiver must accompany the subject in participating the study visits, and must have sufficient interaction and exchange with the patient, so as to provide valuable information for scoring on the Neuropsychiatric Inventory (NPI), Activities of Daily Living Scale (ADCS-ADL), and Clinician Interview-Based Impression of Change (CIBIC-plus);  8．The patient agrees to participate in this trial, and he/she or his/her guardian signs the informed consent form before the trial.  **Exclusion criteria**  1. The patient has participated in another clinical trial within 30 days before the start of this study;  2．The patient is pregnant or breastfeeding;  3．The patient has an allergic constitution;  4．The patient has dementia caused by other reasons: vascular dementia, central nervous system infection (such as: AIDS, syphilis, etc.), Creutzfeldt-Jakob disease, Huntington's disease and Parkinson's disease, Lewy body dementia, dementia resulting from traumatic brain injury, other physical and chemical factors (such as: drug poisoning, alcohol poisoning, carbon monoxide poisoning, etc.), major physical illnesses (such as hepatic encephalopathy, pulmonary encephalopathy, hypoxic encephalopathy, etc.), intracranial lesions (such as: subdural hematoma, communicating hydrocephalus, brain tumor), endocrine system diseases (such as thyroid disease and parathyroid disease), and dementia caused by a lack of vitamins or other elements;  5．The patient has had nervous system disorders before (including stroke, neuromyelitis optica, Parkinson's disease, epilepsy, etc.);  6．The patient has abnormal laboratory test indicators: liver function (alanine aminotransferase (ALT) and aspartate aminotransferase (AST)) exceeds the upper limit of the normal range by 1.2 times, Cr exceeds the upper limit of the normal range by 1.2 times, white blood cell count ＜4.0×10^9^ or platelet count ＜100×10^9^, hemoglobin ＜100g/L, random blood glucose level exceeds the upper limit of the normal range by 1.2 times;  7．The patient has uncontrolled high blood pressure: systolic BP≥160mmHg or diastolic BP≥100mmHg;  8．The patient has unstable or serious cardiac, pulmonary, hepatic, renal or hematopoietic system diseases (including unstable angina pectoris, uncontrollable asthma, active bleeding, cancer, etc.);  9．The patient has vision and hearing disorders and is unable to complete the neuropsychological tests and rating scale;  10．The patient's computer tomography (CT) scan or magnetic resonance imaging (MRI) examination performed within one year before enrollment shows focal lesions that are clinically significant;  11．The patient is an alcohol abuser or drug abuser;  12．The patient has mental disorders, including serious depression;  13．The patient is currently receiving treatment for Alzheimer's disease, and the treatment cannot be discontinued;  14．The patient has taken heparin, sodium double-ester alginic acid and propylene glycol mannate sulfate within three weeks before screening;  15．The study staff believes that the subject is unable to complete this study for whatever reasons;  16．The subject is an employee or a close relative of personnel related to the investigator, staff of the study center, GCP ClinPlus Co., Ltd. or Shanghai Green Valley Pharmaceutical Co., Ltd. | |
| **Specifications, batch number, administration and dosage of drugs in the trial**  **Study drug:**  Name: Sodium Oligomannate capsule (971 capsule);  Specifications: 150mg/capsule;  Validity: 2 years.  **Drug for the run-in phase and control drug**:  Placebo that does not contain any effective treatment ingredients and is provided by Shanghai Green Valley Pharmaceutical Co., Ltd.;  Specifications: 150mg/capsule;  Validity: 2 years.  **Administration and dosage:**  **Run-in phase**: Placebo, 3 capsules/time, taken orally twice a day.  **Treatment phase**:  Low-dose group (600mg group): 2 Sodium Oligomannate capsules (971 capsule) + 1 placebo capsule to be taken each time;  High-dose group (900mg group): 3 Sodium Oligomannate capsules (971 capsule) to be taken each time;  Placebo group: 3 placebo capsules to be taken each time;  All the groups will take the capsules twice a day, in the morning and at night, for 24 weeks in total. | |
| **Course:** A trial cycle includes 4 weeks of screening/run-in phase and 24 weeks of double-blind treatment phase. | |
| **Evaluation criteria:**  **1) Primary efficacy endpoint**  Improvement in cognitive functions: Evaluated using the Alzheimer's Disease Assessment Scale-Cognitive (ADAS-cog/12) scale  **2) Secondary efficacy endpoints**  Overall evaluation: Evaluated using the CIBIC-plus scale;  Improvement in daily living functions: Evaluated using the ADCS-ADL scale;  Improvement in neuropsychiatric behavior: Evaluated using the NPI scale.  **3) Safety indicators:** Including adverse events, laboratory tests (blood routine test and urinalysis, blood biochemistry and coagulation function), ECG examination, vital signs (blood pressure and heart rate) and physical examination. | |
| **Number of visits:**  There are five onsite visits and three telephone visits in total. Onsite visits are carried out at Week -4, baseline, Week 4, Week 12 and Week 24.Telephone visits are carried out at Week 2, Week 8 and Week 16. | |
| **Analysis population**  **Full analysis set (FAS)**: Based on the intent to treat (ITT) principle, subjects have been excluded in the smallest and most reasonable manner. The FAS population refers to all patients who have been randomly assigned to the groups, have entered the double-blind treatment phase, and have been administered with at least one drug therapy and assessed for corresponding efficacy. Some of the missing data related to efficacy of the FAS population is added using the last observation carried forward (LOCF) method.  The FAS will be used for the analysis of efficacy indicators and balanced analysis of general information and basic indicators, and it is the primary data set for the efficacy evaluation under this study.  **Per protocol analysis set (PPS):** Those who fulfill the following criteria shall form the PPS population: ① compliance within 80%-120%; ② have not taken any prohibited drugs during the trial; ③ meet the indicators in the inclusion criteria; ④ have completed all the planned visits and the CRF has been completed as per requirement. No carryforward will be made for the missing data in this data set, and it will still be treated as missing data.  PPS is the secondary data set for the efficacy evaluation under this study.  **Safety analysis set (SS)**: Refers to all the populations that have been enrolled into the trial and taken one medication, and have suitable subsequent information for safety analysis. All safety information and data obtained from subjects will be assessed, including adverse events and laboratory test results.  Safety analysis will be conducted based on the actual groupings that received treatment. SS is the main data set for safety evaluation. | |
| **Statistical methods**  **General principles**  The SAS v9.2 statistical analysis software will be used for all statistical analyses.  All the statistical tests will be performed using two-sided test, and when the P value is 0.05 or less, the tested difference is deemed to be statistically significant (unless otherwise specified).  Quantitative indicators are described by calculating the mean, standard deviation, median, minimum value, maximum value and interquartile range.  Qualitative indicators are described using different number of cases and percentages.  Definition of baseline: The last observation before taking the study drug for the first time.  Unless otherwise specified, the data list will include all the randomized population.  **Safety evaluation**  The safety analysis set population is analyzed.  The adverse events are coded using the classification method in the universal MEDDRA terminology. The contents of all events are analyzed based on the groupings and the number of cases, incidence rate and frequency are analyzed based on the severity.  For laboratory tests conducted before and after treatment, the completed examination items and descriptive statistics are presented in the form of cross tabulation; missing values are separately categorized as unchecked items. The examination items conducted after treatment that show abnormalities (including abnormalities without clinical significance and abnormalities with clinical significance) will be listed out and their respective measured values before treatment will be attached. The ECG examination and physical examination are summarized with reference to the laboratory tests.  The vital signs at Week 4, Week 12 and Week 24 of treatment, and their changes as compared to baseline are described statistically by calculating the total number of cases, missing data, mean, standard deviation, minimum value, maximum value, median and interquartile range, and the differences between groups are compared using variance analysis. | |
| **Results**  **1. Characteristics of Subjects**  In this study, 255 subjects were enrolled in reality, with 85 subjects, 84 subjects and 86 subjects in the placebo group, Sodium Oligomannate capsule 600mg group and Sodium Oligomannate capsule 900mg group, respectively. Among the enrolled subjects, 32 (12.55%) of them dropped out and 223 (87.45%) of them completed the trial. The number of subjects entering FAS in the placebo group, 600mg group and 900mg group was 83, 76 and 83, respectively, the number of subjects entering PPS was 75, 67 and 71, respectively, and the number of subjects entering SS was 85, 84 and 86, respectively. At baseline, the demographic characteristics, vital signs, physical examination of all systems, Alzheimer's disease history, past history or comorbid diseases, history of drug allergy and abuse, history of alcohol abuse and other such clinical conditions of the three groups of subjects were not statistically significant. The clinical features of all indicators of laboratory tests and ECG examination were essentially balanced, and there was no significant difference between the groups. The subjects in all three groups had good drug compliance during the study.  **2. Safety results**  In this study, the overall incidence rate of adverse events in the placebo group, 600mg group and 900mg group was 41.18%, 36.90% and 27.91%, respectively. The incidence rate of adverse events related to the study drug was 3.53%, 5.59% and 3.49%, respectively. The incidence rate of adverse events leading to dropouts was 1.18%, 3.57% and 3.49%, respectively. The drug-related adverse events mainly manifested as mild to moderate psychiatric symptoms (such as insomnia and irritation) and skin symptoms (rash).  There were 14 cases (15 incidents) of serious adverse events (SAEs) in the entire study. Upon evaluation by the clinical investigator, ten of the SAEs were definitely unrelated to the drug, four SAEs were possibly unrelated to the drug, and one SAE (600mg group, 01028) was possibly related to the drug. See Table below.  Summary of adverse events (SS)   |  | **Placebo**  **(N=85)** | | |  | **Sodium Oligomannate capsule 600mg (N=84)** | | |  | **Sodium Oligomannate capsule**  **900mg (N=86)** | | | | --- | --- | --- | --- | --- | --- | --- | --- | --- | --- | --- | --- | | **Item** | Number of cases | Number of incidents | Incidence rate (%) |  | Number of cases | Number of incidents | Incidence rate (%) |  | Number of cases | Number of incidents | Incidence rate (%) | | All adverse events | 35 | 66 | 41.18 |  | 3 | 64 | 36.90 |  | 24 | 51 | 27.91 | | Adverse events related to the study drug* | 3 | 6 | 3.53 |  | 5 | 12 | 5.95 |  | 3 | 3 | 3.49 | | Serious adverse events | 6 | 6 | 7.06 |  | 5 | 6 | 5.95 |  | 3 | 3 | 3.49 | | Serious adverse events related to the study drug* | 0 | 0 | 0.00 |  | 1 | 1 | 1.19 |  | 0 | 0 | 0.00 | | Adverse events leading to dropouts | 1 | 1 | 1.18 |  | 3 | 5 | 3.57 |  | 3 | 3 | 3.49 |   Note: *Related to the study drug: Includes those who are "definitely related", "possibly related", "probably related" and "suspected to be related" to the study drug.  During the study, there was no abnormal changes in the temperature, respiration and systolic blood pressure of subjects in the three groups. There was also no intergroup differences. In terms of heart rate, there was a reduction of two beats in the 600mg group at Week 12 as compared to baseline (P<0.05).In terms diastolic blood pressure, there were significant differences (P<0.05) among the three groups at Week 4, as well as significant differences (P<0.05) in the changes as compared to baseline of the three groups at Week 24. The change in the placebo group, 600mg group and 900mg group as compared to baseline was 0.22mmHg, -2.03mmHg and 2.00mmHg, respectively, and there was an increment of 2mmHg (P<0.05) in the 900mg group at Week 24 of treatment as compared to baseline.  In terms of physical examination, there were no abnormal changes in the general condition, lymph nodes, neck, ear, nose, throat, lungs and spine of subjects in the three groups. The abnormal changes that occurred were:  Placebo group: three cases at the eyes and one case at the heart;  600mg group: one case on the skin, one case at the abdomen and one case at the limbs;  900mg group: one case on the skin and one case at the nervous system.  In terms of the laboratory test and ECG examination, the percentages of abnormal indicators of clinical significance appearing in the various examinations in the final visit of subjects in the three groups as compared to baseline were similar. | |
| **Conclusion:**  The use of 600mg and 900mg Sodium Oligomannate capsules for the treatment of patients with mild to moderate Alzheimer's disease for 24 weeks is safe with good tolerability. | |
| **Report date: November 2, 2013** | |

Table of Contents for Full Text

[**Main members of the study** 15](#__RefHeading___Toc48384944)

[**Main members of the study (continued)** 17](#__RefHeading___Toc48384945)

[**List of Abbreviations** 18](#__RefHeading___Toc48384946)

[**1.** **Introduction** 21](#__RefHeading___Toc48384947)

[2. Objectives of the study 21](#__RefHeading___Toc48384948)

[3. Management of the study 22](#__RefHeading___Toc48384949)

[3.1 Management organization of the study 22](#__RefHeading___Toc48384950)

[3.2 Training of study staff 23](#__RefHeading___Toc48384951)

[3.3 Monitoring of the clinical study 23](#__RefHeading___Toc48384952)

[3.4 Reporting system for serious adverse events 24](#__RefHeading___Toc48384953)

[3.5 Laboratory quality control 25](#__RefHeading___Toc48384954)

[3.6 Data management 25](#__RefHeading___Toc48384955)

[3.7 Withdrawal from the clinical study 25](#__RefHeading___Toc48384956)

[4. Study design and study process 26](#__RefHeading___Toc48384957)

[4.1 Overall study plan 26](#__RefHeading___Toc48384958)

[4.2 Study design and considerations for the selection of control group 26](#__RefHeading___Toc48384959)

[4.3 Sample size 27](#__RefHeading___Toc48384960)

[4.4 Selection of study subjects 27](#__RefHeading___Toc48384961)

[4.5 Study process 31](#__RefHeading___Toc48384962)

[4.6 Safety evaluation 46](#__RefHeading___Toc48384963)

[5. Data quality assurance 49](#__RefHeading___Toc48384964)

[5.1 Data collection and revision 49](#__RefHeading___Toc48384965)

[5.2 Verification of original records 49](#__RefHeading___Toc48384966)

[5.3 Blind review and unblinding 49](#__RefHeading___Toc48384967)

[5.4 Data lock 50](#__RefHeading___Toc48384968)

[6. Statistical processing plan 51](#__RefHeading___Toc48384969)

[6.1 General principles 51](#__RefHeading___Toc48384970)

[6.2 Data set for statistical analysis 51](#__RefHeading___Toc48384971)

[6.3 Characteristics of subjects 52](#__RefHeading___Toc48384972)

[6.4 Safety evaluation 53](#__RefHeading___Toc48384973)

[6.5 Concomitant medication 54](#__RefHeading___Toc48384974)

[7. Amendments made during the study 55](#__RefHeading___Toc48384975)

[7.1 Protocol amendments made during the study 55](#__RefHeading___Toc48384976)

[7.2 Change of clinical centers 56](#__RefHeading___Toc48384977)

[8. Interim analysis 57](#__RefHeading___Toc48384978)

[9. Characteristics of subjects 57](#__RefHeading___Toc48384979)

[9.1 Distribution of subjects 57](#__RefHeading___Toc48384980)

[9.2 Serious deviations from the study protocol 67](#__RefHeading___Toc48384981)

[**9.3 Demographics and other baseline characteristics** 68](#__RefHeading___Toc48384982)

[9.4 Drug exposure situation and medication compliance 70](#__RefHeading___Toc48384983)

[9.5 Concomitant medication 71](#__RefHeading___Toc48384984)

[10. Safety evaluation results 72](#__RefHeading___Toc48384985)

[**10.1 Overall safety evaluation results** 72](#__RefHeading___Toc48384986)

[10.2 Sub-group safety analysis 88](#__RefHeading___Toc48384987)

[10.3 Safety analysis of exploratory indicators 90](#__RefHeading___Toc48384988)

[**11. Discussion and conclusion** 91](#__RefHeading___Toc48384989)

**Ethics Statement**

This study is conducted in accordance with the Good Clinical Practice (GCP, CFDA Order No. 3, promulgated on August 6, 2003) promulgated by China Food and Drug Administration, and it complies with the Declaration of Helsinki of the World Medical Assembly (18th World Medical Association General Assembly, Helsinki, Finland, June 1964) and the latest revised version) (59th World Medical Association General Assembly, Seoul, South Korea, October 2008).The study protocol is in compliance with local regulations, and the informed consent form file has been submitted to the ethics committee for review and approval before obtaining the ethics approval document.

The amendment of protocol for this study has also been approved in writing by the ethics committee.

A hard copy of the approval document of the ethics committee has been submitted to the investigator, who then provides a duplicate copy of the approval document to the study sponsor. The approval document of the ethics committee contains the list of all committee members involved in the document discussion and their respective duties (refer to appendix).

Before the start of screening, the investigator has understood the objectives, methods, benefits and potential risks of this clinical study, and has obtained the informed consent form signed by the subjects of the clinical trial. The subject of the clinical trial shall personally (or through a lawful agent) sign and date the informed consent form, which is then kept properly by the investigator. The subject shall keep a duplicate copy.

During the clinical study, all issues related to the safety of clinical study, such as serious adverse events in the clinical study protocol and clinical study, shall be reported promptly to the ethics committee.

Upon conclusion of the clinical study, the conclusion shall be reported concurrently to the ethics committee.

Main members of the study

| **Participating unit** | **Name** | **Job title** | **Department or institution** | **Division of research work** |
| --- | --- | --- | --- | --- |
| Mental Health Center Affiliated with Shanghai Jiao Tong University School of Medicine  (Shanghai Mental Health Center) | Xiao Shifu | Department Head | Psychogeriatric Department | Principal investigator |
| Second Affiliated Hospital of Chongqing Medical University | Chen Yangmei | Department Head | Department of Neurology | Principal investigator |
| Guangzhou Brain Hospital (Guangzhou Psychiatric Hospital) | Tang Mouni | Superintendent | Department of Neurology | Principal investigator |
| First Affiliated Hospital of Harbin Medical University | Zhang Liming | Department Head | Department of Neurology | Principal investigator |
| Hunan Provincial People's Hospital | Gao Xiaoping | Department Head | Department of Neurology | Principal investigator |
| Brain Hospital Affiliated with Nanjing Medical University (Nanjing Brain Hospital) | Li Hailin | Department Head | Psychogeriatric Department | Principal investigator |
| Ninth People's Hospital Affiliated with Shanghai Jiao Tong University School of Medicine | Li Wei | Department Head | Department of Neurology | Principal investigator |
| West China Hospital of Sichuan Hospital | Kuang Weihong | Department Head | Psychogeriatric Department | Principal investigator |
| Tianjin Medical University General Hospital | Cheng Yan | Department Head | Department of Neurology | Principal investigator |
| Zhongnan Hospital of Wuhan University | Xiao Jingsong | Deputy Department Head | Department of Neurology | Principal investigator |
| Beijing Hospital of the Ministry of Health | Chen Haibo | Department Head | Department of Neurology | Principal investigator |
| Sir Run Run Shaw Hospital Affiliated with Zhejiang University School of Medicine | Chen Wei | Deputy Department Head | Psychiatric Department | Principal investigator |
| No. 251 Hospital of the People's Liberation Army | Li Yingjie | Department Head | Department of Neurology | Principal investigator |
| Third Affiliated Hospital, Third Military Medical University, People's Liberation Army (Daping Hospital, Third Military Medical University) | Zhou Huadong | Department Head | Department of Neurology | Principal investigator |
| First Affiliated Hospital, Third Military Medical University, People's Liberation Army | Shi Shugui | Department Head | Department of Neurology | Principal investigator |
| Wuhan General Hospital of Guangzhou Military Region, People's Liberation Army  (Wuhan General Hospital of Guangzhou Military Region) | Li Honghua | Department Head | Department of Neurology | Principal investigator |
| First Affiliated Hospital of China Medical University | He Zhiyi | Department Head | Department of Neurology | Principal investigator |
| Sun Yat-sen Memorial Hospital, Sun Yat-sen University  (Source: Second Affiliated Hospital of Sun Yat-Sen University) | Peng Ying | Department Head | Department of Neurology | Principal investigator |
| Shandong Provincial Hospital | Du Yifeng | Department Head | Department of Neurology | Principal investigator |
| Tangdu Hospital, Fourth Military Medical University, People's Liberation Army | Li Hong | Department Head | Department of Neurology | Principal investigator |

**Main members of the study (continued)**

| **Participating unit** | **Name** | **Job title** | **Department or institution** | **Division of research work** |
| --- | --- | --- | --- | --- |
| Baotou Central Hospital | Wang Baojun | Department Head | Department of Neurology | Principal investigator |
| Wuxi Mental Health Center | Xu Wenwei | Department Head | Psychogeriatric Department | Principal investigator |
| Second Hospital of Shandong University | Bi Jianzhong | Superintendent | Department of Neurology | Principal investigator |
| Qilu Hospital of Shandong University | Yan Chuanzhu | Department Head | Department of Neurology | Principal investigator |
| GCP ClinPlus Co., Ltd. | Liang Suping |  | Department of Biostatistics | Biostatistician |
|  | Sun Haiquan |  | Department of Biostatistics | Biostatistician |
|  | Peng Yanfen |  | Data Management Department | Data Management |
|  | Wu Ting |  | Data Management Department | Data Management |

**List of Abbreviations**

Explanation of the abbreviations and statistics (in English) used in this text:

| **English abbreviation** | **Chinese name** | **English abbreviation** | **Chinese name** |
| --- | --- | --- | --- |
| Aβ | β-amyloid peptide | GGT | γ-glutamyl transferase |
| AD | Alzheimer's disease | GLUT-1 | Glucose transporter protein-1 |
| ADAS-cog | Alzheimer's Disease Assessment Scale  -cognitive component | GMP | Good Manufacturing Practice |
| ADCS-ADL | Activities of Daily Living Scale | GSH-PX | Glutathione peroxidase |
| ALB | Serum albumin | HAMD | Hamilton Depression Scale |
| ALP | Alkaline phosphatase | HGB | Hemoglobin |
| ALT | Alanine aminotransferase | HIS | Hachinski Ischemic Scale |
| ATPase | Adenosine triphosphatase | IL-6 | Interleukin-6 |
| APOE | Apolipoprotein E | INR | International normalized ratio |
| APTT | Activated partial thromboplastin time | IWRS | Interactive Web Response System |
| AST | Aspartate aminotransferase | LD50 | Median lethal dose |
| BEHAVE-AD | Pathological behavior rating scale for Alzheimer's disease | LEU | Urinary leucocytes |
| BMI | Body mass index | LOCF | Last observation carried forward |
| BUN | Blood urea nitrogen | LSMEAN | Least-squares means |
| CFDA | China Food and Drug Administration  - | LY% | Percentage of lymphocytes |
| ChAT | Choline acetyltransferase | LSMEAN | Least-squares means |
| CHO | Cholesterol | Max | Maximum value |
| CIBIC-plus | Clinician Interview-Based Impression of Change  Rating Scale | Md(Q3-Q1) | Median (interquartile range) |
| Cr | Creatinine | Mean | Mean |
| CRA | Clinical monitor | Min | Minimum value |
| CRF | Case report form | MMSE | Mini Mental State Examination |
| CSF | Cerebrospinal fluid | MPV | Mean platelet volume |
| CT | Computer tomography | MRI | Magnetic resonance imaging |
| DBIL | Direct bilirubin | NFT | Neurofibrillary tangle |
| EDC | Electronic data capture | NINCDS-ADRDA | National Institute of Neurological and Communicative Disorders and Stroke-Alzheimer's Disease and Related Disorders Association |
| FAS | Full analysis set | NMDA | N-methyl-D-aspartate |
| FT_3_ | Serum free triiodothyronine | NPI | Neuropsychiatric questionnaire - clinician rating scale |
| FT_4_ | Serum free tetraiodothyronine | NPI-C | Neuropsychiatric symptoms questionnaire - based on caregiver |
| GAGs | Endogenous glycosaminoglycans | PET | Positron emission tomography |

**List of Abbreviations (continued)**

| **English abbreviation** | **Chinese name** | **English abbreviation** | **Chinese name** |
| --- | --- | --- | --- |
| PLT | Platelets | T_3_ | Triiodothyronine |
| PPS | Per-protocol set | T_4_ | Tetraiodothyronine |
| PS1 | Presenilin-1 | TBIL | Total bilirubin |
| PT | Prothrombin time | T_max_ | Peak time |
| P-tau | Phosphorylated tau protein | TNF-α | Tumor necrosis factor-α |
| QOL-AD | Quality of Life Scale - Alzheimer's Disease | TP | Total protein |
| RBC | Red blood cell | TSH | Thyroid stimulating hormone |
| SAE | Serious adverse events | T-tau | Total tau protein |
| SD | Standard deviation | VitB12 | Vitamin B12 |
| SOD | Superoxide dismutase | WBC | White blood cell |
| SP | Senile plaques | ∆/δ | Effect size |
| SS | Safety set |  |  |

1. **Introduction**

Sodium Oligomannate capsule (971 capsule) is a chemical drug under registration category 1.2; a multi-center, randomized, double-blind, placebo-controlled, parallel-group, dose-finding phase II clinical study was conducted in accordance with the "Measures for the Administration of Drug Registration".

This study used placebo as a control to investigate the optimal therapeutic dose and performed our initial evaluation of the efficacy and safety of Sodium Oligomannate capsule (971 capsule) in the treatment of mild to moderate Alzheimer's disease. The approval document number of China Food and Drug Administration for clinical study on new drug was: 2006L02492, 2011L00942.The Mental Health Center Affiliated with Shanghai Jiao Tong University School of Medicine (Shanghai Mental Health Center) was chosen as the lead unit for this clinical study, and Professor Xiao Shifu was appointed as the principal investigator. The clinical study was approved by the Mental Health Center Affiliated with Shanghai Jiao Tong University School of Medicine (Shanghai Mental Health Center on August 17, 2011, the first subject was enrolled on October 24, 2011, and the treatment and observation of all subjects were completed on July 10, 2013.

# 2. Objectives of the study

**Primary objective:** To investigate the optimal therapeutic dose and perform an initial evaluation of the efficacy and safety of Sodium Oligomannate capsule (971 capsule) in the treatment of mild to moderate Alzheimer's disease.

**Secondary objectives:**

1．To evaluate the effect of Sodium Oligomannate capsule on the cerebrospinal fluid biomarkers (Aβ_40_, Aβ_42_, T-tau, P-tau);

2．to determine the concentrations of Sodium Oligomannate in the cerebrospinal fluid and blood after reaching steady state.

# 3. Management of the study

## 3.1 Management organization of the study

The drug registration applicant was Shanghai Green Valley Pharmaceutical Co., Ltd. The monitoring, data management and statistical analysis of the study were completed by GCP ClinPlus Co., Ltd.

The original plan was to have 26 clinical study institutions participating in this study. Thereafter, due to the screening failure of Huashan Hospital Affiliated with Fudan University (Center 03) and the early closing of Ruijin Hospital Affiliated with Shanghai Jiao Tong University School of Medicine (Center 08) with enrollment difficulties, 24 clinical study institutions managed to complete this study in the end. The lead unit for the clinical study was the Mental Health Center Affiliated with Shanghai Jiao Tong University School of Medicine (Shanghai Mental Health Center), and the participating units included: Second Affiliated Hospital of Chongqing Medical University, Guangzhou Brain Hospital (Guangzhou Psychiatric Hospital), First Affiliated Hospital of Harbin Medical University, People's Hospital of Hunan Province, Brain Hospital Affiliated with Nanjing Medical University (Nanjing Brain Hospital), Ninth People's Hospital Affiliated with Shanghai Jiao Tong University School of Medicine, Tianjin Medical University General Hospital, Zhongnan Hospital of Wuhan University, Beijing Hospital of the Ministry of Health, Sir Run Run Shaw Hospital Affiliated with the School of Medicine of Zhejiang University, No. 251 Hospital of the People's Liberation Army, First Affiliated Hospital, Third Military Medical University, People's Liberation Army (Daping Hospital, Third Military Medical University), First Affiliated Hospital, Third Military Medical University, People's Liberation Army, Wuhan General Hospital of Guangzhou Military Region, People's Liberation Army (Wuhan General Hospital of Guangzhou Military Region), First Affiliated Hospital of China Medical University, Sun Yat-sen University of Sun Yat-Sen Memorial Hospital (Original: Second Affiliated Hospital of Sun Yat-Sen University), Shandong Provincial Hospital, Tangdu Hospital, Fourth Military Medical University, People's Liberation Army, Baotou Central Hospital, Wuxi Mental Health Center, Second Hospital of Shandong University, Qilu Hospital of Shandong University.

Ethics committee: Seven study centers (Ninth People's Hospital Affiliated with Shanghai Jiao Tong University School of Medicine, West China Hospital of Sichuan Hospital, , Beijing Hospital of the Ministry of Health, First Affiliated Hospital, Third Military Medical University, People's Liberation Army, Shandong Provincial Hospital, Tangdu Hospital, Fourth Military Medical University, People's Liberation Army, and Baotou Central Hospital) conducted the study according to the ethical principles of the ethics committee of Shanghai Jiao Tong University School of Medicine (Shanghai Mental Health Center), which was the lead unit. The other 16 study centers conducted the study according to the ethics approval document of the respective hospital centers.

## 3.2 Training of study staff

Investigators who were involved in the clinical study must have the necessary expertise, qualifications and skills for clinical studies. Before the start of the trial, all staff involved in the trial were trained on the study protocol, the current GCP and the current standard operating procedures related to this trial. In order to maintain the consistency of scale assessment, a standard training on the scales of this study was conducted, so that the study staff had full understanding and knowledge of the clinical study protocol and detailed meaning of all indicators. The recording method and judgment criteria were also standardized, and the quality of complete data was ensured. Units participating in the study had to appoint specialized personnel to store and keep all the study drugs in accordance with the national regulations on drug clinical studies, and to dispense the drugs based on the investigator's instructions. The process of dispensing and returning drugs was recorded correctly, and the recording forms were archived accordingly.

## 3.3 Monitoring of the clinical study

This study was monitored through the contact kept by the clinical research associate (CRA) of GCP ClinPlus Co., Ltd. with the study staff through regular visits, telephone and/or email.

The clinical research associate was familiar with all the investigators and the members of all clinical study centers involved in the various processes (including the dispensing of study drugs) in this clinical study. The CRA regularly inspected the work situation of the clinical study centers and conducted onsite or telephone monitoring based on the monitoring plan and actual needs before each center enrolled the first subject, during the conduct of the clinical study and after the conclusion of the clinical study.

The CRA provided a detailed explanation of the results of raw data review based on the standard operating procedures. The standard operating procedures for monitoring were kept confidential by the CRA. The CRA checked the original medical records of patients, drug dispensing records and archived documents (study files), and compared the clinical study records with the original medical records of subjects, so as to ensure the authenticity of clinical data. At the same time, the CRA also observed the study procedures and discussed about the existing problems with the investigators. The CRA and current investigators verified whether the clinical study was conducted in strict accordance with the clinical study protocol, and resolved any existing problems in the clinical study together. All the informed consent forms of all the screened and enrolled patients of the clinical study were checked.

For such visits, the investigators provided sufficient time and ensured that the CRA had access to the source data documents of data entered into the case report forms.

## 3.4 Reporting system for serious adverse events

Prior to the start of the study, the reporting system for serious adverse events was established, and all serious adverse events occurring during the observation period were to be reported according to the requirements of the corresponding national regulations regardless of whether they were related to the study drug. The corresponding information was then promptly provided to Shanghai Green Valley Pharmaceutical Co., Ltd. The serious adverse events (SAEs) were to be reported to the clinical research associate, sponsor's contact person, principal investigator, medical ethics committee of the lead unit, Safety Supervision Department and Registration Department of China Food and Drug Administration, and the provincial drug administration at the locale of the clinical study unit within 24 hours after having been made known. The evaluation and records of serious adverse events included: time of occurrence, severity, relationship with the study drug, measures taken in relation to the study drug, and the current conclusion.

## 3.5 Laboratory quality control

The laboratories of all centers provided internal and external quality control certificates and the list of range of normal laboratory values, so as to ensure the reliability of reported data. All the laboratory indicators in this study were tested in the laboratories of all centers. The APOE genotype and cerebrospinal fluid biomarkers were tested in the central laboratory.

## 3.6 Data management

The data management work was completed by GCP ClinPlus Co., Ltd.

The electronic data capture (EDC) system was used to collect data; this system can set the corresponding rights based on the different roles of the users. The data collection interface was designed based on the protocol, and the investigator carried out data collection and recording. The data management staff completed the online query management of data. The queries were verified by two integrated approaches: the first approach was the automatic verification of the system before the submission of data by the investigator, and the other approach was the manual verification by the data management staff after the submission of data. Using the SAS procedures, the data was verified, and the data management staff posted a query on the system for doubtful data. The investigator then verified, revised or confirmed the data, and all the revision of data was recorded in detail in the EDC.

## 3.7 Withdrawal from the clinical study

The subjects had the right to withdraw from the study at any time and for any reason. If a subject withdrew on his or her own initiative, the investigator would contact the patient through a phone call or visit, or find out the full reasons for the withdrawal as much as possible from the patient's relatives.

In the event of an aggravation of the original disease, adverse event, allergic reaction occurring after the use of study drug, violation of treatment program, or other reasons due to management and others, the investigator also had the right to decide whether the subject should discontinue the drug administration or withdraw from the study based on the actual circumstances. If a patient withdrew from the study due to adverse events or abnormal laboratory test results, this was recorded in the case report.

Patients who withdrew early from the study cannot be replaced by other subjects. For patients who withdrew from the study for whatever reasons, their effectiveness and safety data were obtained as much as possible, and all the assessment at the end of treatment was conducted with the subject's willingness and compliance. For subjects who lost contact (that is, those who did not participate in the study visits and did not produce any statement on their intention to withdraw from the study, such that their status became unknown), the investigator recorded the steps of attempts to establish contact with these subjects in the raw data, such as the date of phone calls and registered mail, etc.

# 4. Study design and study process

## 4.1 Overall study plan

This study was a multi-center, randomized, double-blind, placebo-controlled, parallel-group, dose-finding phase Ⅱ clinical study.

252 patients with mild to moderate Alzheimer's disease passed the screening. They first entered the 4-week run-in phase, followed by the 24-week treatment period if they passed the run-in phase. They were randomly assigned in a double-blind manner to the low dose group (Sodium Oligomannate capsule 600mg group), high dose group (Sodium Oligomannate capsule 900mg group) and control group (placebo group). There were 84 subjects in each group, and patients were competitively enrolled into the groups in all centers.

## 4.2 Study design and considerations for the selection of control group

### 4.2.1 Study design

This drug is a chemical drug under registration category 1.2; a multi-center, randomized, double-blind, placebo-controlled, parallel-group, dose-finding phase II clinical study was conducted in accordance with the "Measures for the Administration of Drug Registration". This study investigated the optimal therapeutic dose and performed an initial evaluation of the efficacy and safety of Sodium Oligomannate capsule (971 capsule) in the treatment of mild to moderate Alzheimer's disease. The design of this study was completed by drawing reference from local and overseas research progress on Alzheimer's disease, as well as the "Technical Guidelines for Clinical Studies on Drugs to Treat Alzheimer's Disease" promulgated by China Food and Drug Administration, EMEA's "Evaluation Guidelines for Clinical Studies on Drugs to Treat Alzheimer's Disease and Other Dementia", as well as data on the pharmacodynamics and chronic toxicology of 971 in preclinical studies, and data of the phase I clinical study.

### 4.2.2 Design of the placebo

The placebo did not contain any effective treatment ingredients and was provided by Shanghai Green Valley Pharmaceutical Co., Ltd.; specifications: 150 mg/capsule; validity period: 2 years.

The placebo and Sodium Oligomannate capsules were exactly the same in terms of taste, smell and appearance, as well as design, and both had passed the quality standard inspection of China Food and Drug Administration. The manufacturing date and expiry date of the drugs were provided during use.

All the study drugs were produced in workshops that met the quality management requirements for drug production (GMP), and drug approval certificates had been obtained.

## 4.3 Sample size

The sample size in this study was calculated using the PASS software. When there were 70 subjects in each group, an effect size (∆/δ) of at least 0.5 can be detected in the comparison between the study group and the placebo control group under a power of 80% and an inspection standard of 0.05, and taking the dropout rate of 20% into account, each group was expected to enroll 84 subjects, amounting to a total of 252 cases for the three groups. The actual enrollment was 255 subjects. There were 85, 84 and 86 subjects in the placebo group, 600mg group and 900mg group, respectively.

## 4.4 Selection of study subjects

Male and female patients with mild to moderate Alzheimer's disease who met the enrollment criteria were shortlisted.

### 4.4.1 Inclusion criteria

1) Aged 50-85 years, male or female;

2) Primary school and higher education level;

3) Met the diagnostic criteria of possible Alzheimer's disease (AD) in the National Institute of Neurological and Communicative Disorders and Stroke-Alzheimer's Disease and Related Disorders Association (NINCDS-ADRDA) (1984);

4) Patients with mild to moderate condition, that is, 10 points ≤Total MMSE score ≤24 points;

5) Total HIS score <4 points;

6) Total HAMD score ≤10 points;

7) The subject should have a stable and reliable caregiver, or can at least have frequent contact with the caregiver (at least 4 days in a week, at least 2 hours per day), and the caregiver will assist the patient in participating in the entire study process. The caregiver must accompany the subject in participating the study visits, and must have sufficient interaction and exchange with the patient, so as to provide valuable information for scoring on the NPI, ADCS-ADL and CIBIC**-**plus scales.

8) The patient has agreed to participate in this study, and he/she or his/her guardian has signed the informed consent form before the study.

### 4.4.2 Exclusion criteria

1) The patient participated in another clinical study within 30 days before the start of this study;

2) The patient is pregnant or breastfeeding;

3) The patient has an allergic constitution;

4) The patient has dementia caused by other reasons: vascular dementia, central nervous system infection (such as: AIDS, syphilis, etc.), Creutzfeldt-Jakob disease, Huntington's disease and Parkinson's disease, Lewy body dementia, dementia resulting from traumatic brain injury, other physical and chemical factors (such as: drug poisoning, alcohol poisoning, carbon monoxide poisoning, etc.), major physical illnesses (such as hepatic encephalopathy, pulmonary encephalopathy, hypoxic encephalopathy, etc.), intracranial lesions (such as: subdural hematoma, communicating hydrocephalus, brain tumor), endocrine system diseases (such as thyroid disease and parathyroid disease), and dementia caused by a lack of vitamins or other elements;

5) The patient has had nervous system disorders before (including stroke, neuromyelitis optica, Parkinson's disease, epilepsy, etc.);

6) The patient has abnormal laboratory test indicators: liver function (ALT and AST) exceeded the upper limit of the normal range by 1.2 times, Cr exceeded the upper limit of the normal range by 1.2 times, white blood cell count <4.0×10^9^ or platelet count <100×10^9^, hemoglobin <100 g/L, random blood glucose level exceeded the upper limit of the normal range by 1.2 times;

7) The patient has uncontrolled high blood pressure: systolic BP≥160mmHg or diastolic BP≥100mmHg;

8) The patient has unstable or serious cardiac, pulmonary, hepatic, renal or hematopoietic system diseases (including unstable angina pectoris, uncontrollable asthma, active bleeding, cancer, etc.);

9) The patient has vision and hearing disorders and is unable to complete the neuropsychological tests and rating scale;

10) The patient's CT or MRI examination performed within one year before enrollment show focal lesions that are clinically significant;

11) The patient is an alcohol abuser or drug abuser;

12) The patient has mental disorders, including serious depression;

13) The patient is currently receiving treatment for Alzheimer's disease, and the treatment cannot be discontinued;

14) The patient took heparin, sodium double-ester alginic acid and propylene glycol mannate sulfate within three weeks before screening;

15) The study staff believe that the subject is unable to complete this study for whatever reasons;

16) The subject is an employee or a close relative of personnel related to the investigator, staff of the study center, GCP ClinPlus Co., Ltd. or Shanghai Green Valley Pharmaceutical Co., Ltd.

### 4.4.3 Situations of non-completion of study

#### 4.4.3.1 Dropout criteria

1) The subject or his/her caregiver withdraws the informed consent form;

2) Individual cases of abnormal unblinding during the study;

3) Lose to follow-up;

4) Allergy to any component of Sodium Oligomannate capsule;

5) Occurrence of unacceptable adverse reaction/serious adverse event;

6) The subject develops serious complications or experiences a worsening of condition during the study period, which require emergency measures to be taken;

7) The patient becomes pregnant during the study period;

8) Other reasons.

Subjects have the right to withdraw from the study at any time without being subject to any discrimination or retaliation. If a subject decides to withdraw from the study, the investigator has to find out the reasons as much as possible and record the reasons on the case report form. The investigator has the right to terminate a subject's continual participation in the study in the event of the following situations: occurrence of adverse events or other management issues, and the clinical study cannot be continued based on the investigator's judgment. When a subject withdraws early, that subject must be assessed. If the subject withdraws from the study due to adverse drug events or abnormal laboratory test values, these must be recorded on the case report form, and the study conclusion page must be filled in.

#### 4.4.3.2 Elimination Criteria

1) Serious violation of the inclusion and exclusion criteria;

2) Poor protocol compliance, use of prohibited drugs during the study.

In the blind review meeting, the principal investigator and statistician decides whether the patient is to be eliminated. For eliminated patients, the reasons must be explained and the CRF has to be retained for future reference.

#### 4.4.3.3. Criteria for termination of study

Study termination refers to the discontinuation of the entire study before the clinical study is concluded based on the protocol. The main purposes are to protect the rights and interest of subjects, ensure the quality of research, and avoid unnecessary financial losses.

1) When there are serious safety issues in the study, the study has to be terminated promptly.

2) When poor efficacy is found during the study, there is no need to continue conducting the study.

3) When major errors are found in the clinical study protocol during the study, making it difficult to evaluate the effects of the drug; or when there are major deviations during the implementation of a relatively well-designed protocol, making it difficult to evaluate the effects of the drug if the study were to continue on;

4) The sponsor requests for the study to be terminated (for funding reasons, management reasons, etc.);

5) The administrative authorities revokes the study, etc.

## 4.5 Study process

### 4.5.1 Study drugs

#### 4.5.1.1 Drug specifications

| **Item** | **Study drug** |
| --- | --- |
| Generic name | Sodium Oligomannate capsule (971 capsule) |
| Dose type | Capsule |
| Specifications | 150 mg/capsule |
| Validity | 2 years. |
| Storage conditions | Sealed and stored in a dry place, away from sunlight. |
| Manufacturer | Shanghai Green Valley Pharmaceutical Co., Ltd. |
| Supplier | Shanghai Green Valley Pharmaceutical Co., Ltd. |

The placebo and Sodium Oligomannate capsules were exactly the same in terms of taste, smell and appearance, as well as design, and both had passed the quality standard inspection of China Food and Drug Administration.

#### 4.1.2 Administration and dosage

This study consisted of the low-dose group (Sodium Oligomannate capsule 600mg group), high-dose group (Sodium Oligomannate capsule 900mg group) and placebo group. The study cycle included a 4-week screening/run-in phase and a 24-week treatment period.

| **Run-in phase** | Placebo, 3 capsules/time, taken orally 2 times each day. |
| --- | --- |
| **Treatment period** | **Low-dose group (600mg group)**: 2 Sodium Oligomannate capsules (971 capsule) + 1 placebo capsule to be taken each time;  **High-dose group (900mg group)**: 3 Sodium Oligomannate capsules (971 capsule) to be taken each time;  **Placebo group**: 3 placebo capsules to be taken each time;  All the groups will take the capsules twice a day, in the morning and at night, for 24 weeks in total. |

#### 4.5.1.3 Management and storage of the study drug

The study drug had to be stored in a safe and dry place under room temperature and away from sunlight. There were specialized staff who were responsible for managing the study drugs in all the study centers, and only the investigators, personnel appointed by the investigators and the CRA were granted access to the drugs.

The drugs could only be used on eligible patients who were enrolled into this clinical study. The study drugs were dispensed to the eligible and enrolled patients in strict accordance with the drug numbers specified in the IWRS system. When dispensing drugs, the pharmacist or clinicians had to carefully verify the enrollment number to ensure that the drugs were dispensed according to the specified numbers. The clinical research associate would regularly collect back the remaining drugs and drug packaging. The investigator had to accept, store, dispense and collect back the drugs in accordance with the relevant regulations, and take the corresponding records. The investigator must return the unused drugs to the sponsor. The study center had the obligation to keep the list of drugs and accept the monitoring, audit and inspection of drugs carried out by the CRA, auditors and China Food and Drug Administration (CFDA).

#### 4.5.1.4 Drug packaging

Prior to the start of the clinical study, all the drugs used in the study were packed in the same manner. Three tablets were packed into aluminum and plastic trays, and the following contents had to be specified on the external packaging of the drugs used for the study: clinical study approval number, drug quantity inside the box, drug specifications, storage conditions, instructions for use, validity and drug supplier, and the words "for clinical study use only" had to be indicated clearly.

#### 4.5.1.5 Drug distribution and records

The study drug was sent directly by the sponsor to the study center, and the study drug management staff of the study center dispensed the drug to each clinical study personnel based on the drug number allocated by the Interactive Web Response System (IWRS); the clinical study personnel then dispensed the drugs to the subjects.

After all the patients were found eligible during the visits in the screening period, the investigator logged in to the IWRS to obtain the randomization number and drug number of the respective subjects. The subjects were randomly assigned to each study group based on the ratio of 1:1:1.During each visit, the investigator had to record in detail the receipt and return of study drugs by the subjects, and fill in the drug dispensing registration form in a prompt and accurate manner.

The investigator was responsible for the dispensing, storing and tracking all clinical study drugs, and promptly recorded the dispensing and quantity of all clinical study drugs. After the termination of the study, the investigator had to return all the remaining drugs and inventory records to the CRA.

Dispensing the study drugs to patients who are not a subject of this study was prohibited.

The investigator had to record, on the drug inventory form, the drugs received in each batch and the drugs dispensed to each patient.

#### 4.5.1.6 Drug Inventory

The investigator was responsible for taking stock, checking and recording the study drugs. The investigator or designated personnel must keep record of the quantity of drugs in the entire study process. The clinical research associate would regularly check the drug inventory, returned drugs and dispensing record form of the study center. Upon conclusion of the study, all the remaining drugs, returned drugs and packaging would be counted by the investigator and CRA and collected back by the sponsor if everything was in order.

### 4.5.2 Blinding

#### 4.5.2.1 Creation and keeping of blind codes

This study was a randomized, double-blind, multi-center clinical study. The statistician created the random blind codes for the study drugs and completed the preparation of the random number table and the blind coding of drugs. The random number table made used of the SAS 9.1.3 software and the proc plan process, and it was created using the simple randomization method. The seed number used to create the random numbers and the random number table were kept as the blind codes. When the random table was created, an emergency document corresponding to each random number was created at the same time. The biostatistician then performed packaging based on the aforementioned blind codes. Each subject was allocated a drug box that contained all the drugs used during the study. The corresponding number was written on the label of the drug box, and the same number was written on the label of all drugs inside the box. After the packaging of the drugs was completed, the drug blind codes were sealed and three copies were kept with the Mental Health Center Affiliated with Shanghai Jiao Tong University School of Medicine (Shanghai Mental Health Center), GCP ClinPlus Co., Ltd. and Shanghai Green Valley Pharmaceutical Co., Ltd. During the study, the blind codes could not be opened and read. The emergency document was given to the study unit along with the study drugs with the corresponding number, and the document was kept at the principal investigator's office in the study center.

#### 4.5.2.2 Emergency unblinding

During the occurrence of serious adverse events and situations requiring patient rescue that rendered the need to know the drug that the patient was taking, the person responsible for the study in the center would report to the person responsible for the clinical study, and the decision to perform emergency unblinding could be made after seeking consent. The principal investigator of all centers could obtain the detailed grouping information from the IWRS system or the emergency document that was sent together with the study drugs to the study unit. Once the emergency unblinding had been performed said patient was then handled as a dropout case. The time, location and reason of unblinding, and personnel involved in the unblinding had to be recorded promptly and reported promptly to the sponsor, CFDA and responsible person for the clinical study.

#### 4.5.2.3 Unblinding provisions

As the one-time unblinding approach was adopted in this study, the unblinding was carried out after the blinding status had been verified, the statistical analysis plan had been confirmed and the data had been locked.

### 4.5.3 Randomization

Dynamic randomization was achieved in this study through the IWRS, and the stratification factors of study center, education level (primary school, secondary school and above), whether cerebrospinal fluid lumbar puncture was performed, and whether PET imaging was performed were taken into consideration. All the dispensed study drugs had the corresponding numbers, and the doctors had to register in the IWRS system based on the sequence of consultation of each subject, and dispense the drugs used in the study to the specific subject based on the drug number provided by the system without choosing the drug numbers. The drug number allocated by the IWRS system covered all the drugs used by the subject during the study treatment period (24 weeks). The investigator had to provide the drugs in batches to the specified subject based on the protocol requirements. Each subject would receive the treatment of one of the three treatment groups based on the drug number, and the treatment had to begin within three days after randomization.

### 4.5.4 Study flow chart

| **Step** | **Screening period/**  **Run-in phase** | **Baseline period^1^** | **After treatment**  **Week 2** | **After treatment**  **Week 4** | **After treatment**  **Week 8** | **After treatment**  **Week 12** | **After treatment**  **Week 16** | **After treatment**  **Week 24** | **Unplanned**  **follow-up** |
| --- | --- | --- | --- | --- | --- | --- | --- | --- | --- |
| **Visit date** | **Visit 0**  **(Week -4)** | **Visit 1 (0d)** | **Telephone follow-up 1**  **(Week 2±3d)** | **Visit 2**  **(Week 4±3d)** | **Telephone follow-up 2**  **(Week 8±7d)** | **Visit 3**  **(Week 12±7d)** | **Telephone follow-up 3**  **(Week 16±7d)** | **Visit 4**  **(Week 24±7d)** | **As required in the study or during early withdrawal from study** |
| Informed consent form | **×** |  |  |  |  |  |  |  |  |
| Inclusion/Exclusion criteria | **×** | **×^2^** |  |  |  |  |  |  |  |
| Basic medical history information | **×** |  |  |  |  |  |  |  |  |
| Physical examination | **×** | **×** |  |  |  |  |  | **×** | **×** |
| Vital signs | **×** | **×** |  | **×** |  | **×** |  | **×** | **×** |
| Urine pregnancy test^3^ | **×** |  |  |  |  |  |  | **×** |  |
| Routine blood and urine tests | **×** |  |  | **×** |  | **×** |  | **×** | **×** |
| Blood biochemistry | **×** |  |  | **×** |  | **×** |  | **×** | **×** |
| Coagulation profile^4^ | **×** |  |  | **×** |  | **×** |  | **×** | **×** |
| Folic acid, VitB_12_, thyroid function, syphilis testing | **×** |  |  |  |  |  |  |  |  |
| Collection of samples for APOE genotype test | **×** |  |  |  |  |  |  |  |  |
| Electrocardiogram | **×** |  |  | **×** |  | **×** |  | **×** | **×** |
| Head MRI | **×** |  |  |  |  |  |  |  |  |
| **Study flow chart (continued)** | | | | | | | | | |
| **Step** | **Screening period/**  **Run-in phase** | **Baseline period^1^** | **After treatment**  **Week 2** | **After treatment**  **Week 4** | **After treatment**  **Week 8** | **After treatment**  **Week 12** | **After treatment**  **Week 16** | **After treatment**  **Week 24** | **Unplanned**  **follow-up** |
| **Visit date** | **Visit 0**  **(Week -4)** | **Visit 1 (0d)** | **Telephone follow-up 1**  **(Week 2±3d)** | **Visit 2**  **(Week 4±3d)** | **Telephone follow-up 2**  **(Week 8±7d)** | **Visit 3**  **(Week 12±7d)** | **Telephone follow-up 3**  **(Week 16±7d)** | **Visit 4**  **(Week 24±7d)** | **As required in the study or during early withdrawal from study** |
| PET imaging^5^ |  | **×** |  |  |  |  |  | **×** |  |
| MMSE | **×** | **×** |  |  |  |  |  |  |  |
| HIS | **×** | **×** |  |  |  |  |  |  |  |
| HAMD | **×** | **×** |  |  |  |  |  |  |  |
| ADAS-cog |  | **×** |  | **×** |  | **×** |  | **×** |  |
| CIBIC-plus |  | **×** |  | **×** |  | **×** |  | **×** |  |
| ADCS-ADL |  | **×** |  | **×** |  | **×** |  | **×** |  |
| NPI questionnaire |  | **×** |  | **×** |  | **×** |  | **×** |  |
| Recording of concomitant medication | **×** | **×** | **×** | **×** | **×** | **×** | **×** | **×** | **×** |
| Recording of adverse events |  | **×** | **×** | **×** | **×** | **×** | **×** | **×** | **×** |
| Dispensing/collection of study drug |  | **×** |  | **×** |  | **×** |  | **×** |  |
| Scheduling visit date | **×** | **×** |  | **×** |  | **×** |  |  |  |

Note:

1 The screening period results were accepted as the results for the urine pregnancy test, routine blood and urine tests, biochemical examination, coagulation profile check, ECG and head MRI during the baseline period.

2 At the end of the run-in phase, the inclusion/exclusion criteria had to be verified again.

3 The urine pregnancy test was done on women with childbearing potential.

4 Coagulation profile: including the calculation of prothrombin time, partial thromboplastin time and international normalized ratio.

5 PET imaging was performed in some centers.

### 4.5.5 Study and assessment

#### 4.5.5.1 Assessment during the screening/run-in phase - Visit 0 (Week -4)

The investigator had to record, in the screening form, the status of compliance of each pending patient with the inclusion criteria and whether that patient was eventually enrolled or eliminated, and submit the form to the study application unit. After passing the screening, all the patients then entered the four-week placebo single-blind run-in phase. The following assessment or steps had to be completed during the screening/run-in phase assessment.

1) Signing of the informed consent form: this had to be completed before the screening;

2) Completion of general data;

3) Inquiry about the medical history, including past history, current medical history, treatment history and other history of concomitant diseases and medication;

4) Physical examination and vital signs (temperature, resting heart rate, respiration and blood pressure after resting for 10 minutes);

5) Head MRI examination (exempted for those who could provide results within one year before screening);

6) Scale assessment: including HIS score, HAMD score and MMSE score;

7) Laboratory tests (results within seven days before going through screening):

① Routine blood test：red blood cell count (RBC), hemoglobin (HGB), white blood cell count (WBC), neutrophil percentage, lymphocyte percentage, monocyte percentage, eosinophil percentage, basophil percentage, PLT;

① Routine urine test：urine pH (pH), urinary protein, leukocyte (LEU), red blood cells;

③ Blood biochemistry： alanine aminotransferase (ALT), aspartate aminotransferase (AST), total bilirubin (TBiL), direct bilirubin (DBiL), alkaline phosphatase (ALT), serum albumin (ALB), urea nitrogen (BUN), creatinine (Cr), γ-glutamyl transferase (GGT), immediate blood glucose level;

④ Coagulation profile: including the calculation of prothrombin time, partial thromboplastin time and international normalized ratio;

⑤Folic acid, vitamin B_12_ (VB_12_), thyroid function (triiodothyronine (T_3_), tetraiodothyronine (T_4_), serum free triiodothyronine (FT_3_), serum free tetraiodothyronine (FT_4_), thyroid stimulating hormone (TSH)), syphilis testing;

⑥ Retention of blood specimens: APOEε4 genotype test;

⑦ Urine pregnancy test (women with childbearing potential);

8) ECG examination (results within seven days before going through screening):

9) Recording of adverse events;

10) Recording of drug compliance;

11) Dispensing of drugs for the run-in phase, and informing patients of the method of administration: three tablets taken orally twice a day;

12) Scheduling of next visit date.

#### 4.5.5.2 Baseline assessment - Visit 1 (Day 0)

After the run-in phase, the investigator then assessed the compliance of patients, and conducted the assessment of inclusion/exclusion criteria again on those with better compliance (80%-120% compliance with the taking of placebo). The eligible patients then entered the randomization stage and the study drugs were dispensed to them. The efficacy data during baseline assessment had to be obtained within three days (within 72 hours) before the first administration of study drug. The results in the screening period were accepted as the results of laboratory tests (routine blood and urine tests, blood biochemistry, coagulation profile and urine pregnancy test), ECG and head MRI. The following assessment and steps had to be completed during baseline assessment:

1) Review of the inclusion/exclusion criteria and assess the compliance in the screening period;

2) Vital signs: the temperature, resting heart rate, respiration and blood pressure after resting for 10 minutes;

3) Physical examination

4) Scale assessment: including the HIS score, HAMD score, MMSE assessment, ADAS-cog/12 scale assessment, ADCS-ADL scale assessment, NPI assessment, CIBIC-plus scale;

5) PET imaging examination: observation of the glucose metabolism in the brain (conducted only in some centers);

6) Recording of adverse events;

7) Recording of concomitant medication;

8) Dispensing of drugs for the run-in phase, and informing patients of the method of administration: one tray (three tablets) taken orally twice a day;

9) Scheduling of next visit date.

#### 4.5.5.3 Four weeks after treatment - Visit 2 (Week 4±3 days)

1) Vital signs: the temperature, resting heart rate, respiration and blood pressure after resting for 10 minutes;

2) Scale assessment: including the ADAS-cog scale, CIBIC-plus scale, ADCS-ADL scale and NPI questionnaire;

3) Laboratory tests:

① Routine blood tests: RBC, HGB, WBC, neutrophil percentage, lymphocyte percentage, monocyte percentage, eosinophil percentage, basophil percentage, PLT;

① Routine urine test：urine pH, urinary protein, LEU, red blood cells;

③ Blood biochemistry ALT, AST, TBiL, DBiL, ALP, ALB, BUN, Cr, GGT, immediate blood glucose level;

④ Coagulation profile: including the calculation of prothrombin time, partial thromboplastin time and international normalized ratio;

4) ECG;

5) Recording of adverse events;

6) Recording of concomitant medication;

7) Dispensing/collecting of drugs for the run-in phase, and informing patients of the method of administration: one tray (three tablets) taken orally twice a day;

8) Scheduling of next visit date.

#### 4.5.5.4 Twelve weeks after treatment - Visit 3 (Week 12±7 days)

1) Vital signs: the temperature, resting heart rate, respiration and blood pressure after resting for 10 minutes;

2) Scale assessment: including the ADAS-cog scale assessment, ADCS-ADL scale assessment, NPI questionnaire and CIBIC-plus scale;

3) Laboratory tests:

① Routine blood test： RBC, HGB, WBC, neutrophil percentage, lymphocyte percentage, monocyte percentage, eosinophil percentage, basophil percentage, PLT;

① Routine urine test：urine pH, urinary protein, LEU, red blood cells;

③ Blood biochemistry：ALT, AST, TBiL, DBiL, ALP, ALB, BUN, Cr, GGT, immediate blood glucose level;

④ Coagulation profile: including the calculation of prothrombin time, partial thromboplastin time and international normalized ratio;

4) ECG;

5) Recording of adverse events;

6) Recording of concomitant medication;

7) Dispensing/collecting of drugs for the run-in phase, and informing patients of the method of administration: one tray (three tablets) taken orally twice a day.

#### 4.5.5.5 Twenty-four weeks after treatment - Visit 4 (Week 24±7 days)

1) Vital signs: the temperature, resting heart rate, respiration and blood pressure after resting for 10 minutes;

2) Scale assessment: including the ADAS-cog scale assessment, ADCS-ADL scale assessment, NPI questionnaire and CIBIC-plus scale;

3) Laboratory tests:

① Routine blood test：RBC, HGB, WBC, neutrophil percentage, lymphocyte percentage, monocyte percentage, eosinophil percentage, basophil percentage, PLT;

② Routine urine test：urine pH, urinary protein, LEU, red blood cells;

③ Blood biochemistry：ALT, AST, TBiL, DBiL, ALP, ALB, BUN, Cr, GGT, immediate blood glucose level;

④Coagulation profile: including the calculation of prothrombin time, partial thromboplastin time and international normalized ratio;

4) PET imaging examination: observation of the glucose metabolism situation in the brain (conducted only in some centers);

5) ECG;

6) Recording of adverse events;

7) Recording of concomitant medication;

8) Collection of study drugs.

#### 4.5.5.6 Telephone follow-up

On the 2nd week, 9th week and 16th week after treatment, the investigator had a telephone follow-up with the subjects to find out the occurrence of adverse events, including the patients' mental condition, diet, urine and stool condition, and dry mouth, as well as the concomitant medication situation.

#### 4.5.5.7 Unplanned visits (as required in the study or during early withdrawal from study)

When there was a need for a subject to go for additional visits or withdraw early from the study, the measures taken by the investigator (including the laboratory test results) were recorded in the original medical records and the unplanned visit table in the case report form.

In addition, when the subject withdrew from the study before the completion of the study, safety assessment had to be conducted as much as possible, and the investigator also filled in the study conclusion page.

#### 4.5.5.8 Safety assessment

Safety assessment was carried out at baseline and after drug administration. For patients who withdrew early from the study, safety assessments were conducted before withdrawal. Abnormal laboratory tests with obvious clinical significance were re-measured within one week as much as possible, and the results were recorded on the "unplanned follow-up" section of the CRF; the necessary clinical treatment was also applied until the measurements returned to normal or stabilized.

Safety assessment included adverse events, vital signs and laboratory tests, as well as situations of early withdrawal due to safety or tolerability reasons, and all the adverse events and corresponding treatment were recorded in the CRF.

### 4.5.6 Concomitant medication

Symptomatic medication can be used for other concomitant diseases so long as they did not affect the judgment of the efficacy of the study drug. Apart from treating the cause of disease, concomitant medication was reduced as much as possible.

#### 4.5.6.1 Prohibited drugs for the study observation period

If a subject took any of the listed "prohibited drugs" for treatment during the study period, that subject would have to withdraw from the study. However, if the said treatment lasted <7 days, the investigator could immediately discontinue the use of prohibited drug and continue with the study based on the subject's safety and the need to receive new treatment. The situation of this use of prohibited drug would also be recorded under the "concomitant medication" section in the CRF.

###### Table 1 Prohibited drugs

| 1.Drugs that are currently being taken or taken in the past 30 days or five half-lives (whichever is longer) | |
| --- | --- |
| 1a Within 30 days before Visit 1 or five half-lives (whichever is longer).  2a Taken when necessary (PRN), defined as: taken in the past 8 weeks when necessary, and drug was administered less than four times each week (more frequent medication is defined as chronic short-term or long-term medication.  Long-term medication is defined as a chronic, fixed-dose medication for ≥2 months before the first visit. | |
| Type of prohibited drugs | Explanation |
| Adrenocorticotropic hormone |  |
| Central nervous system stimulants |  |
| Traditional Chinese Medicine (TCM) supplements |  |
| All TCM and Western medicine nootropics that affect memory | Such as hydergine, duxil, piracetam, Kangnaoshuai, aniracetam, etc. |
| NMDA antagonists | Such as amantadine, ketamine and dextromethorphan |
| Antipsychotics | This excludes risperidone, quetiapine and olanzapine that are already stably used before randomization, and the dose was to be kept stable as much as possible during the study. |
| Antidepressants | This excludes sertraline, citalopram and escitalopram that are already stably used for more than four weeks before randomization, and the dose was to be kept stable as much as possible during the study. |
| Sedative-hypnotics | Sedative-hypnotics (when necessary, zopiclone, alprazolam and estazolam could be used temporarily).For those who had been on long-term sedative-hypnotics, the dose stabilized for at least four weeks before randomization, and the dose was to be kept stable as much as possible during the study. |
| Heparin, sodium double-ester alginic acid and propylene glycol mannate sulfate |  |

4.5.6.2 Drugs permitted during the studyAny drugs other than the prohibited drugs stated in Table 1. If drugs that might have a negative impact on cognitive function were used, such as anticholinergic drugs (including drugs with significant anticholinergic activity, such as amitriptyline) and most sedatives (typical or atypical antipsychotics), the dose had to be stabilized for at least four weeks before baseline, and the dose was to be kept unchanged as much as possible during the study.

### 4.5.7 Assessment of medication compliance

The investigator had to emphasize the importance of compliance to the subjects.

During the returned visits, the subjects had to return the packaging of used drugs such as capsule trays, as well as unused drugs and packaging. The investigator or designated personnel then took stock of the quantity of returned drugs, calculated the quantity of used drugs, and compared it with the quantity of drugs that should be taken. Records had to be taken for lost or unreturned drugs.

Medication compliance = Actual quantity of drugs taken / Quantity of drugs that should be taken × 100%.

Criterion for good medication compliance: 80% ≤compliance ≤120%; poor compliance: compliance <80% or >120%.

If a subject had poor compliance, the reasons should be found and recorded.

The investigator had to record the quantity of dispensed and returned drugs in the corresponding portion of the case report form.

## 4.6 Safety evaluation

The safety parameters included adverse events, laboratory tests, ECG, vital signs and physical examination.

All the adverse events that occurred during the drug therapy were recorded in the CRF. The assessment of adverse events included the category, grade, relationship with the drug, treatment measures and outcome.

#### 4.6.1 Adverse events

##### 4.6.1.1 Definition of adverse events

An adverse event is an adverse medical event occurring after a subject takes a certain drug. It may not necessarily be related to the drug, and includes any new event or event of deterioration in terms of severity or frequency as compared to baseline. This includes abnormal results in diagnostic methods such as laboratory tests. The adverse events that occurred in the process of this study were to be recorded in the case report form. For adverse events that occurred during the study, the symptoms, severity, onset time, duration, treatment measures and process were recorded on the observation form, and the correlation with the study drug was evaluated after considering the complications and concomitant medication fully. The doctor then recorded the details accordingly. The adverse events had to be recorded using medical terminology, and the diagnosis of the disease should be given as much as possible instead of providing a list of symptoms and their signs (for example: cough, runny nose, sneezing and sore throat were reported as upper respiratory tract infection).

##### 4.6.1.2 Relationship of adverse event with the study drug

The investigator had to assess the possible correlation between the study drug and concomitant medication and determine the relationship between the adverse events and the treatment based on the five grading criteria below.

**Definitely related**: the reactions occur in a reasonable sequence after medication and are coherent with the type of known reactions suspected for the drug; the condition improves after drug withdrawal, but recur after the drug is administered again;

**Possibly related**: the reactions occur in a reasonable sequence after medication and are coherent with the type of known reactions suspected for the drug; the condition improves after drug withdrawal, but may recur after the drug is administered again, and the patient's clinical condition or other treatment methods may also produce the said reactions;

**Probably related:** the reactions occur in a reasonable sequence after medication and are coherent with the type of known reactions suspected for the drug; the patient's clinical condition or other treatment methods may also produce the said reactions;

**Probably unrelated:** the reactions do not occur in a reasonable sequence after medication and are not very coherent with the type of known reactions suspected for the drug; the patient's clinical condition or other treatment methods may also produce the said reactions;

**Unrelated:** the reactions do not occur in a reasonable sequence after medication and are coherent with the type of known reactions for drugs outside of the study; the patient's clinical condition or other treatment methods may also produce the said reactions, the reactions disappear when the disease condition improves or other treatment methods are discontinued, but recur when the other treatment methods are used again.

##### 4.6.1.3 Grading of the severity of adverse events

1) Mild: cases whereby the adverse reaction was mild, the symptoms did not develop, the patient had slight discomfort, and the adverse reaction usually did not require treatment and drug withdrawal and could subside on its own;

2) Moderate: cases whereby the adverse reaction was obvious, the patient had obvious discomfort, and the major organs or systems were moderately damaged;

3) Severe: cases whereby the major organs or systems are severely damaged and the condition is life-threatening.

#### 4.6.2 Adverse drug reactions

Adverse drug reactions are harmful and unexpected reactions having a causal relationship with the drug application that occur during the normal application of drugs based on the specified dose. In a clinical study on a new drug or new drug application, when its therapeutic dose has not been determined, all the harmful and unexpected reactions having a causal relationship with the drug application should also be considered as adverse drug reactions.

#### 4.6.3 Clinical laboratory test anomalies

When laboratory test anomalies are not accompanied by clinical symptoms and signs, they are not recorded as adverse events, and such changes would be analyzed during data analysis. However, if the investigator deems that the necessary treatment is to be given to address the extent of laboratory test anomalies, such laboratory tests would be recorded as adverse events, and the corresponding treatment has to be recorded too. All the abnormal laboratory tests have to be followed up until remission or stable condition.

For laboratory anomalies that are hard to explain, the test has to be conducted until the results are normal and/or the anomalies can be properly explained.

#### 4.6.4 Serious adverse events

Serious adverse drug reactions/events are any of the following situations:

1) cases that lead to death;

2) cases that lead to genetic malformation, cancer or birth defects;

3) cases that are life-threatening and could cause permanent or significant disability in the human body;

4) cases that lead to the permanent damage of organ functions;

5) cases that require the patient to be hospitalized for treatment or hospitalized for a long time.

If a subject experiences a serious adverse event during the study, the investigator has to immediately take the appropriate treatment measures regardless of whether the event is related to the study drug, so as to protect the subject's safety, and the matter has to be reported to the medical ethics committee of the center, the Drug Registration Department of CFDA within 24 hours of occurrence, the health administration and clinical study project manager, sponsor's contact person, principal investigator, GCP center contact person of the lead unit, at the same time, case report form for serious adverse event has to be completed promptly, and data related to the event has to be completed and recorded in detail as much as possible.

The investigator has to make the following assessment and records on the serious adverse event in the case report form: severity, relationship with the study drug, measures taken in relation to the study drug, and the current conclusion. The sponsor has to inform all the involved hospitals and ensure that the reporting procedures required by all laws and regulations are fulfilled.

# 5. Data quality assurance

## 5.1 Data collection and revision

The data collection interface of the EDC system was designed based on the protocol, and the investigator carried out data collection and recording. The data manager would check the data provided by the CRA and when some queries are found, and he would raise the queries to the investigator in the EDC system. The investigator would then verify, revise and confirm the data, and all data revisions would be recorded in detail in the EDC. The system would set the corresponding rights based on the different roles of the users.

## 5.2 Verification of original records

The investigator must properly handle all data obtained in the clinical study process, so as to protect the rights and privacy of patients participating in the clinical study. The investigator must allow the CRA to view and review all the necessary clinical study data, so as to verify the accuracy of the original records and understand the progress of the study. If the original records could not be verified, the investigator had to agree to assist the CRA/auditor/inspector in further confirming the quality of data.

## 5.3 Blind review and unblinding

On August 15, 2013, the sponsor, data manager and statistician performed the data review work at the conference room of Sheraton Changbaishan Resort. The three parties reviewed the data management report, conducted another review of the data of subjects with protocol deviations, and confirmed the data set for statistical analysis.

A blind review is the verification and evaluation of data in the database after the final case report form has been keyed into the database and before the first unblinding.

After all the case report forms are keyed in and verified to be in order, the data manager would then prepare the database inspection report that contains the study completion situation (including the list of dropout subjects), inclusion/exclusion criteria check, integrity check, logical consistency check, outlier detection, time window check, concomitant medication check, adverse event check, etc.

In the blind review meeting, the principal investigator, sponsor, CRA, data manager and biostatistics professional reviewed the informed consent form signed by subjects, maintenance of blinding in the study process, emergency unblinding during the study process, and other such situations. They also made decisions on the issues raised in the database inspection report, completed the final definition and judgment of the analysis population, and drafted out the blind review report. At the same time, the database was locked.

## 5.4 Data lock

On August 15, 2013, GCP ClinPlus Co., Ltd. locked the data and handed it over to the statistical analysis personnel for analysis. The data would be locked after the following conditions are met:

1) All data have been collected;

2) All queries have been resolved;

3) The analysis population has been defined.

The data lock was completed by the data manager, and the locked data was handed over to the statistical analysis personnel for analysis.

# 6. Statistical processing plan

## 6.1 General principles

The SSA v9.2 statistical analysis software will be used for all statistical analyses.

All the statistical tests will be performed using two-sided test, and when the P value is 0.05 or less, the tested difference is deemed to be statistically significant (unless otherwise specified).

The description of quantitative indicators included the calculation of the mean, standard deviation, median, minimum value, maximum value and interquartile range.

Qualitative indicators are described using different number of cases and percentages. For qualitative indicators with the same significance level (same indicator level), they will be described using quantitative and qualitative variables at the same time.

Definition of baseline: The most recent observation would be before the first administration of the study drug.

Unless otherwise specified, the data list will include all the randomized population.

## 6.2 Data set for statistical analysis

### 6.2.1 FAS

Based on the intention-to-treat (ITT) principle, the subjects were excluded in the smallest and most reasonable manner. The FAS population refers to all patients who have been randomly assigned to the groups, have entered the double-blind treatment phase, and have been administered with at least one drug therapy and assessed for corresponding efficacy. Some of the missing data related to efficacy of the FAS population was added using the last observation carried forward (LOCF) method.

The FAS will be used for the analysis of efficacy indicators and balanced analysis of general information and basic indicators, and it is the primary data set for the efficacy evaluation under this study.

### 6.2.2 PPS

Those who met the following conditions formed the PP population: ①compliance within 80%-120%; ②had not taken any prohibited drugs during the trial; ③met the indicators in the inclusion criteria; ④completed all the planned visits and the CRF had been completed as per requirement. No carryforward will be made for the missing data in this data set, and it will still be treated as missing data.

PPS is the secondary data set for the efficacy evaluation under this study.

### 6.2.3 SS

The safety analysis population is all the populations that have been enrolled into the study and administered medication once, and have suitable subsequent information for safety analysis. All safety information records of subjects would be assessed, including adverse events and laboratory test results.

Safety analysis will be conducted based on the actual groupings that received treatment. SS was the primary data set for the safety evaluation under this study.

## 6.3 Characteristics of subjects

### 6.3.1 Situation of enrollment and completion

The total number of subjects randomly assigned to the respective groups and the number of subjects who entered the respective analysis sets were summarized according to the treatment grouping and centers;

The number of subjects randomly assigned to the respective groups, the number of subjects who completed the study, the number of subjects who did not complete the study and the number of subjects who entered the respective analysis sets were summarized;

The list of subjects who did not enter PPS (including the reasons) and the subjects who dropped out of the study were stated separately.

### 6.3.2 General data

Evaluation using FAS

1) Demographics: gender, age, race, height, weight, education level;

2) Correlation with medical history: whether drug therapy was used for in the past one year, whether there were any comorbid diseases, whether there was any family history, and whether there was any history of drug allergy, drug abuse or alcohol abuse;

3) Vital signs: body temperature, respiration, blood pressure (systolic BP and diastolic BP), pulse;

4) Physical examination: general condition, skin, lymph node, neck (including thyroid), eyes, ear, nose, throat, heart, lungs, abdomen, spine, limbs and nervous system.

Age (years) = (Date of informed consent form - Date of birth)/365.25, round off to a whole number

Corresponding descriptions were made for the aforementioned indicators based on their numerical features, and comparisons were made between the quantitative indicators for the three groups of subjects using the analysis of variance/K-W rank sum test; comparisons were made between the qualitative indicators for the three groups of subjects using the Chi-square test/Fisher's exact probability approach.

## 6.4 Safety evaluation

### 6.4.1 Subject's exposure to the study and receipt of the study drug treatment

Study duration and treatment duration: The duration of the subject's exposure to the study and receipt of the study drug treatment were summarized. Wherein:

Treatment duration (days) = Last date of medication - First date of medication + 1;

Study duration (days) = (Date of completion/Date of termination of study - Date of signing of informed consent form) + 1.

Compliance (%) = Actual amount of drug taken / Amount of drug that should be taken x 100%.Wherein: The actual amount of drug taken by the subject was calculated based on the actual amount of drug to be taken as provided in the CRF; compliance was classified as <80%, 80%-120% and >120%, with medication compliance in the range of 80%-120% being good compliance.

The study duration and treatment duration of the three groups were described. The compliance of the three groups was described, and the number of cases and percentage for the respective categories were calculated.

### 6.4.2 Safety evaluation methods

1) Adverse events

In this study, the classification method in the internationally recognized MEDDRA terminology list was used to code the adverse events. Based on the system names and canonical names of the adverse events after coding, the type and frequency of the adverse events, severity, relationship with the study drug, serious adverse events, and adverse events leading to dropout were described in tabular form. The list of subjects who discontinued the study due to adverse events and experienced serious adverse events were specified.

2) Laboratory tests

For laboratory tests (including routine blood test, routine urine test, blood biochemistry and coagulation profile) conducted before and after treatment, the completed examination items and descriptive statistics were presented in the form of cross tabulation (based on the normal range or the investigator's judgment of clinical significance); missing values were separately categorized as unchecked items. The examination items conducted after treatment that show abnormalities (including abnormalities without clinical significance and abnormalities with clinical significance) will be listed out and their respective measured values before treatment will be attached.

2) ECG examination

A summary was provided with reference to the laboratory examinations.

3) Vital signs

The vital signs at Week 4, Week 12 and Week 24 of treatment (body temperature, blood pressure, respiration and heart rate), and their changes when compared to baseline were described statistically by calculating the total number of cases, missing data, mean, standard deviation, minimum value, maximum value, median and interquartile range, and the differences between groups were compared using variance analysis.

4) Physical examination

A summary was provided with reference to the laboratory examinations.

## 6.5 Concomitant medication

The concomitant medication was coded using the classification in the "Contemporary Drug Trade Names and Aliases Dictionary" (compiled and written by the Chinese Pharmaceutical Association, published by Chemical Industry Press and Modern Biotechnology and Medical Technology Publishing Center, August 2006, 2nd Edition).The calculation of the number of cases, number of times and incidence rate of the respective concomitant medications was carried out according to their system names and canonical names after coding. A tabular description of all the concomitant medications and their specific situation was provided, and the said drugs were arranged in order according to center and drug code.

# 7. Amendments made during the study

## 7.1 Protocol amendments made during the study

In this study, the protocol was revised for a total of four times.

**First protocol amendment**

| **Page number/line number** | **Original protocol content (version 1.1, date: 07-20-2011)** | **Revised protocol content (version 1.2, date: 09-26-2011)** | **Reason for amendment** |
| --- | --- | --- | --- |
| Cover page, first line on page 26, last 11th to 13th lines on page 56 | Shandong Provincial Hospital was not in the list of centers | Shandong Provincial Hospital was added to the participating centers | Added 21 centers |

Second protocol amendment

| **Page number/line number** | **Original protocol content (version 1.2, date: 09-26-2011)** | **Revised protocol content (version 1.3, date: 10-10-2011)** | **Reason for amendment** |
| --- | --- | --- | --- |
| 10th to 14th lines on page 50 | No description of hypothesis testing | Added the description of hypothesis testing | The ethics committee of Center 08 (Ruijin Hospital Affiliated with Shanghai Jiao Tong University School of Medicine) suggested the addition of hypothesis testing. |

Third protocol amendment

| **Page number/line number** | **Original protocol content (version 1.3, date: 10-10-2011)** | **Revised protocol content (version 2.0, date: 11-07-2011)** | **Reason for amendment** |
| --- | --- | --- | --- |
| Cover page, first line of page 26, last 6th line of page 56 and 3rd line of page 57 | Tangdu Hospital, Fourth Military Medical University, People's Liberation Army, Baotou Central Hospital, and Wuxi Mental Health Center were not in the list of participating units | Tangdu Hospital, Fourth Military Medical University, People's Liberation Army, Baotou Central Hospital, and Wuxi Mental Health Center were added to the list of centers | to add more centers |

Fourth protocol amendment

| **Page number/line number** | **Original protocol content (version 2.0, date: 11-07-2011)** | **Revised protocol content (version 2.1, date: 12-12-2011)** | **Reason for amendment** |
| --- | --- | --- | --- |
| 1. First line on the cover page of the protocol, 9th line on page 10,  8th line on page 16,  6th line on page 25 and header on all pages. | Clinical trial approval document number:  2006L02492 | Clinical trial approval document number:  2006L02492, 2011L00942 | The specifications for 2006L02492 approval was  100mg, while the specifications used in this study was 150mg, and 2011L00942 was the approval document for the additional specifications. |
| 2. Cover page of the protocol, 6th line on page 25,  last 5th line on page 54 | The participating units were listed in detail | The detailed name list of participating units was removed and replaced by the general description of “20-30 study centers across the country". | This amendment was made because new centers were added during the study according to the study’s progress, and the protocol version had to be changed each time a new center was added to the list. |

## 7.2 Change of clinical centers

The original plan was to have 26 centers participating in this study. Thereafter, due to the screening failure of Center 03 and early closing of Center 08 as there was no enrollment after the start of study, the number of study centers in this study was 24.

Due to the small number of enrolled patients in some centers, those centers with few patients were merged based on geographical proximity. Centers 1 and 9 merged to form Center 1, Center 10 was changed to Center 2, Centers 7 and 24 merged to form Center 3, Center 14 was changed to Center 4, Centers 4 and 20 merged to form Center 5, Centers 11, 13 and 15 merged to form Center 6, Centers 5 and 19 merged to form Center 7, Centers 22 and 23 merged to form Center 8, Centers 2, 16 and 17 merged to form Center 9, Centers 6, 12 and 18 merged to form Center 10, and Centers 21, 25 and 26 merged to form Center 10.

# 8. Interim analysis

There was no interim analysis in this study.

# 9. Characteristics of subjects

## 9.1 Distribution of subjects

In this study, the plan was to enroll 252 subjects, but 255 subjects were actually enrolled, with 85 subjects, 84 subjects and 86 subjects in the placebo group, 600mg group and 900mg group, respectively. Among the enrolled subjects, 32 (12.55%) of them dropped out and 223 (87.45%) of them completed the trial. See Table 9.1-1 for the distribution of patients in all the study centers. See Table 9.1-2 for the distribution of enrolled patients. See Tables 9.1-3, 4 and 5 for the list of subjects who did not enter PPS.

Among the 85 patients in the placebo group, there were 83, 75 and 85 subjects included in the FAS, PPS and SS, respectively. All the 85 subjects had medication and post-medication safety evaluations, hence all of them entered SS. Post-medication efficacy evaluation was not conducted on two patients, hence they did not enter FAS and PPS, and there were 83 patients in the FAS population. In addition, eight patients (two patients withdrew the informed consent form, two patients exceeded the visit window during follow-up, one patient had poor compliance, one patient was lost to follow-up, one patient developed serious complications or experienced a worsening of condition, and one patient had other reasons) did not enter PPS, hence there were 75 patients in the PPS population.

Among the 84 patients in the 600mg group, there were 76, 67 and 84 subjects included in the FAS, PPS and SS, respectively. All the 84 subjects had medication and post-medication safety evaluations, hence all of them entered SS. Eight patients (three patients withdrew the informed consent form and did not have post-medication efficacy evaluation, one patient was lost to follow-up and did not have post-medication efficacy evaluation, two patients had a serious violation of the inclusion and exclusion criteria, and two patients had other reasons) did not enter the FAS and PPS. Therefore, there were 76 patients in the FAS population. There were also nine patients (one patient withdrew the informed consent form, one patient developed serious complications or experienced a worsening of condition, one patient had poor compliance, two patients had adverse events, one patient did not meet the inclusion criteria and had poor compliance, one patient exceeded the visit window during follow-up, one patient was lost to follow-up, and one patient had a serious violation of the inclusion and exclusion criteria) who did not enter PPS, hence there were 67 patients in the PPS population.

Among the 86 patients in the 900mg group, there were 83, 71 and 86 subjects included in the FAS, PPS and SS, respectively. All the 86 subjects had medication and post-medication safety evaluations, hence all of them entered SS. Three patients (one patient developed serious complications or experienced a worsening of condition during the study, and two patients due to the doctors' deviation in the understanding and implementation of the scale assessment method) did not enter FAS and PPS, hence there were 83 patients in the FAS population. There were also 12 patients (five patients withdrew the informed consent form, one patient was lost to follow-up, one patient had a serious event, one patient exceeded the visit window during follow-up, one patient was allergic to the Sodium Oligomannate capsule, one patient had other reasons, one patient had poor compliance, and one patient took "oxiracetam" concomitantly during the treatment and this might have affected the study drug efficacy evaluation)who did not enter PPS, hence there were 71 patients in the PPS population.

###### Table 9.1-1 Distribution of patients in all the study centers

|  | **Placebo** | | | | **Sodium Oligomannate capsule 600mg** | | | | **Sodium Oligomannate capsule 900mg** | | | | **Total** | | | |
| --- | --- | --- | --- | --- | --- | --- | --- | --- | --- | --- | --- | --- | --- | --- | --- | --- |
| **Center** | **Enrollment** | **FAS** | **PPS** | **SS** | **Enrollment** | **FAS** | **PPS** | **SS** | **Enrollment** | **FAS** | **PPS** | **SS** | **Enrollment** | **FAS** | **PPS** | **SS** |
| 1 | 13 | 13 | 11 | 13 | 15 | 14 | 12 | 15 | 12 | 12 | 11 | 12 | 40 | 39 | 34 | 40 |
| 2 | 3 | 3 | 3 | 3 | 5 | 5 | 4 | 5 | 3 | 3 | 3 | 3 | 11 | 11 | 10 | 11 |
| 4 | 3 | 2 | 1 | 3 | 4 | 3 | 3 | 4 | 2 | 2 | 1 | 2 | 9 | 7 | 5 | 9 |
| 5 | 4 | 4 | 4 | 4 | 3 | 3 | 3 | 3 | 5 | 5 | 4 | 5 | 12 | 12 | 11 | 12 |
| 6 | 0 | 0 | 0 | 0 | 0 | 0 | 0 | 0 | 2 | 0 | 0 | 2 | 2 | 0 | 0 | 2 |
| 7 | 4 | 4 | 4 | 4 | 3 | 2 | 2 | 3 | 5 | 5 | 3 | 5 | 12 | 11 | 9 | 12 |
| 9 | 2 | 2 | 1 | 2 | 3 | 3 | 2 | 3 | 1 | 1 | 0 | 1 | 6 | 6 | 3 | 6 |
| 10 | 10 | 10 | 9 | 10 | 7 | 7 | 7 | 7 | 9 | 9 | 9 | 9 | 26 | 26 | 25 | 26 |
| 11 | 1 | 1 | 1 | 1 | 0 | 0 | 0 | 0 | 2 | 2 | 2 | 2 | 3 | 3 | 3 | 3 |
| 12 | 4 | 4 | 3 | 4 | 3 | 3 | 2 | 3 | 3 | 3 | 3 | 3 | 10 | 10 | 8 | 10 |
| 13 | 2 | 2 | 2 | 2 | 1 | 1 | 1 | 1 | 3 | 3 | 1 | 3 | 6 | 6 | 4 | 6 |
| 14 | 4 | 4 | 4 | 4 | 4 | 4 | 3 | 4 | 6 | 5 | 4 | 6 | 14 | 13 | 11 | 14 |
| 15 | 3 | 3 | 3 | 3 | 5 | 4 | 4 | 5 | 4 | 4 | 4 | 4 | 12 | 11 | 11 | 12 |
| 16 | 1 | 1 | 1 | 1 | 0 | 0 | 0 | 0 | 3 | 3 | 1 | 3 | 4 | 4 | 2 | 4 |
| 17 | 2 | 2 | 1 | 2 | 1 | 0 | 0 | 1 | 2 | 2 | 2 | 2 | 5 | 4 | 3 | 5 |
| 18 | 3 | 3 | 2 | 3 | 3 | 3 | 2 | 3 | 3 | 3 | 3 | 3 | 9 | 9 | 7 | 9 |
| 19 | 2 | 2 | 2 | 2 | 2 | 2 | 2 | 2 | 2 | 2 | 2 | 2 | 6 | 6 | 6 | 6 |
| 20 | 3 | 2 | 2 | 3 | 5 | 4 | 4 | 5 | 4 | 4 | 3 | 4 | 12 | 10 | 9 | 12 |
| 21 | 2 | 2 | 2 | 2 | 4 | 4 | 3 | 4 | 2 | 2 | 2 | 2 | 8 | 8 | 7 | 8 |
| 22 | 3 | 3 | 3 | 3 | 1 | 1 | 1 | 1 | 1 | 1 | 1 | 1 | 5 | 5 | 5 | 5 |
| 23 | 5 | 5 | 5 | 5 | 4 | 3 | 2 | 4 | 2 | 2 | 2 | 2 | 11 | 10 | 9 | 11 |
| 24 | 7 | 7 | 7 | 7 | 5 | 5 | 5 | 5 | 7 | 7 | 7 | 7 | 19 | 19 | 19 | 19 |
| 25 | 0 | 0 | 0 | 0 | 3 | 3 | 3 | 3 | 1 | 1 | 1 | 1 | 4 | 4 | 4 | 4 |
| 26 | 4 | 4 | 4 | 4 | 3 | 2 | 2 | 3 | 2 | 2 | 2 | 2 | 9 | 8 | 8 | 9 |
| **Total** | 85 | 83 | 75 | 85 | 84 | 76 | 67 | 84 | 86 | 83 | 71 | 86 | 255 | 242 | 213 | 255 |

###### Table 9.1-2 Distribution of enrolled patients, n (%)

| **Item** | **Placebo** | **Sodium Oligomannate**  **capsule 600mg** | **Sodium Oligomannate**  **capsule 900mg** | **Total** |
| --- | --- | --- | --- | --- |
| Randomly-assigned | 85(100.00%) | 84(100.00%) | 86(100.00%) | 255(100.00%) |
| Completed | 78(91.76%) | 70(83.33%) | 75(87.21%) | 223(87.45%) |
| Dropouts during the study period | 7(8.24%) | 14(16.67%) | 11(12.79%) | 32(12.55%) |
| Withdrawal of informed consent form by subject | 4(4.71%) | 4(4.76%) | 5(5.81%) | 13(5.10%) |
| Abnormal unblinding during the study period | 0(0.00%) | 0(0.00%) | 0(0.00%) | 0(0.00%) |
| Lost to follow-up | 1(1.18%) | 2(2.38%) | 1(1.16%) | 4(1.57%) |
| Allergic to Sodium Oligomannate capsule | 0(0.00%) | 0(0.00%) | 1(1.16%) | 1(0.39%) |
| Occurrence of adverse events | 0(0.00%) | 2(2.38%) | 1(1.16%) | 3(1.18%) |
| The subject developed serious complications or experienced a worsening of condition, emergency measures had to be taken | 1(1.18%) | 1(1.19%) | 1(1.16%) | 3(1.18%) |
| Serious violation of the inclusion and exclusion criteria | 0(0.00%) | 3(3.57%) | 0(0.00%) | 3(1.18%) |
| Poor compliance with the protocol | 0(0.00%) | 0(0.00%) | 1(1.16%) | 1(0.39%) |
| Use of prohibited drugs during the study period | 0(0.00%) | 0(0.00%) | 0(0.00%) | 0(0.00%) |
| Other reasons | 1(1.18%) | 2(2.38%) | 1(1.16%) | 4(1.57%) |

Note: The percentages of all items were calculated using the number of subjects randomly assigned into groups as the denominator.

Table 9.1-3 List of subjects of the placebo group who did not enter PPS

| **Center** | **Subject**  **Number** | **Completed**  **Study** | **Reason for dropout** | **Reason for non-entry to PPS** | **Whether the subject entered FAS** | **Whether the subject entered SS** |
| --- | --- | --- | --- | --- | --- | --- |
| 1 | 01001 | No | Withdrawal of informed consent form by subject | Did not complete the clinical study: The subject withdrew the informed consent form. | Yes | Yes |
| 1 | 01026 | No | Withdrawal of informed consent form by subject | Did not complete the clinical study: The subject withdrew the informed consent form. | Yes | Yes |
| 4 | 04005 | No | Withdrawal of informed consent form by subject | Did not complete the clinical study: The subject withdrew the informed consent form; medication and post-medication safety evaluations were carried out, but post-medication efficacy evaluation was not carried out. | No | Yes |
| 4 | 04010 | No | Other reasons: we could not rule out the possibility that the subject had taken  the prohibited drug, memantine | Did not complete the clinical study: Other reasons (we could not rule out the possibility that the subject had taken the prohibited drug, memantine) | Yes | Yes |
| 7 | 07005 | No | Withdrawal of informed consent form by subject | Did not complete the clinical study: The subject withdrew the informed consent form. | Yes | Yes |
| 9 | 09006 | Yes |  | There was a gap of 15 days between the final medication date and final visit date, which might have affected the efficacy evaluation of the study drug; this was deemed as a serious violation of the protocol. | Yes | Yes |
| 10 | 10001 | Yes |  | The total compliance during the double-blind treatment phase was <80% (total compliance: 73.09%); this was deemed as a serious violation of the protocol. | Yes | Yes |
| 12 | 12005 | No | Lost to follow-up | Did not complete the clinical study: Lost to follow-up. | Yes | Yes |
| 14 | 14007 | No | Other reasons: Due to the illness,  the subject preferred to stay at home  and did not want to go out; the subject lost his temper easily and did not  listen to the counsel of his family members; there was a decline in his daily living abilities.  -Therefore, the subject was unable to go to the hospital for visits. | Did not complete the clinical study: Other reasons (due to the illness, the subject preferred to stay at home and did not want to go out; the subject lost his temper easily and did not listen to the counsel of his family members; there was a decline in his daily living abilities. Therefore, the subject was unable to go to the hospital for visits.) | Yes | Yes |
| 16 | 16003 | No | Withdrawal of informed consent form by subject | Did not complete the clinical study: The subject withdrew the informed consent form. | Yes | Yes |
| 17 | 17003 | Yes |  | There was a gap of 32 days between the final medication date and final visit date, which might have affected the efficacy evaluation of the study drug; this was deemed as a serious violation of the protocol. | Yes | Yes |
| 18 | 18009 | No | The subject developed serious complications  or experienced a worsening of condition, which required emergency measures to be taken. | Did not complete the clinical study: The subject developed serious complications or experienced a worsening of condition during the study period, which required emergency measures to be taken. | Yes | Yes |
| 20 | 20004 | No | Withdrawal of informed consent form by subject | Did not complete the clinical study: The subject withdrew the informed consent form; medication and post-medication safety evaluations were carried out, but post-medication efficacy evaluation was not carried out. | No | Yes |

Table 9.1-4 List of subjects of the Sodium Oligomannate capsule 600mg group who did not enter PPS

| **Center** | **Subject**  **Number** | **Completed**  **Study** | **Reason for dropout** | **Reason for non-entry to PPS** | **Whether the subject entered FAS** | **Whether the subject entered SS** |
| --- | --- | --- | --- | --- | --- | --- |
| 1 | 01002 | No | Withdrawal of informed consent form by subject | Did not complete the clinical study: The subject withdrew the informed consent form. | Yes | Yes |
| 1 | 01017 | No | Withdrawal of informed consent form by subject | Did not complete the clinical study: The subject withdrew the informed consent form; medication and post-medication safety evaluations were carried out, but post-medication efficacy evaluation was not carried out. | No | Yes |
| 1 | 01028 | No | The subject developed serious complications  or experienced a worsening of condition, which required emergency measures to be taken. | Did not complete the clinical study: The subject developed serious complications or experienced a worsening of condition during the study period, which required emergency measures to be taken. | Yes | Yes |
| 2 | 02016 | Yes |  | The total compliance during the double-blind treatment phase was <80% (total compliance: 52.74%); this was deemed as a serious violation of the protocol. | Yes | Yes |
| 4 | 04004 | No | Withdrawal of informed consent form by subject | Did not complete the clinical study: The subject withdrew the informed consent form; medication and post-medication safety evaluations were carried out, but post-medication efficacy evaluation was not carried out. | No | Yes |
| 7 | 07009 | No | Lost to follow-up | Did not complete the clinical study: Lost to follow-up; medication and post-medication safety evaluations were carried out, but post-medication efficacy evaluation was not carried out. | No | Yes |
| 9 | 09007 | Yes |  | The total MMSE score at baseline was 27 points (total MMSE score: >24 points), and inclusion criterion 4 was not met; the total compliance during the treatment phase was 55.23%; this was deemed as a serious violation of the protocol. | Yes | Yes |
| 12 | 12006 | Yes |  | There was a gap of 15 days between the final medication date and final visit date, which might have affected the efficacy evaluation of the study drug; this was deemed as a serious violation of the protocol. | Yes | Yes |
| 13 | 13002 | No | Allergic to Sodium Oligomannate capsule | Did not complete the clinical study: Allergic to the Sodium Oligomannate capsule. | Yes | Yes |
| 14 | 14004 | No | Occurrence of adverse events | Did not complete the clinical study: Occurrence of adverse events. | Yes | Yes |
| 15 | 15008 | No | Serious violation of the inclusion and exclusion criteria | Did not complete the clinical study: Serious violation of the inclusion and exclusion criteria; medication and post-medication safety evaluations were carried out, but post-medication efficacy evaluation was not carried out. | No | Yes |
| 17 | 17004 | No | Serious violation of the inclusion and exclusion criteria | Did not complete the clinical study: Serious violation of the inclusion and exclusion criteria; medication and post-medication safety evaluations were carried out, but post-medication efficacy evaluation was not carried out. | No | Yes |
| 18 | 18007 | No | Lost to follow-up | Did not complete the clinical study: Lost to follow-up. | Yes | Yes |
| 20 | 20011 | No | Withdrawal of informed consent form by subject | Did not complete the clinical study: The subject withdrew the informed consent form; medication and post-medication safety evaluations were carried out, but post-medication efficacy evaluation was not carried out. | No | Yes |
| 21 | 21006 | No | Serious violation of the inclusion and exclusion criteria | Did not complete the clinical study: Serious violation of the inclusion and exclusion criteria. | Yes | Yes |
| 23 | 23002 | No | Other reasons: the V0-related examination was an external institution's results | Did not complete the clinical study: Other reasons (the V0-related examination was an external institution's results); medication and post-medication safety evaluations were carried out, but post-medication efficacy evaluation was not carried out. | No | Yes |
| 23 | 23007 | No | Occurrence of adverse events | Did not complete the clinical study: Occurrence of adverse events. | Yes | Yes |
| 26 | 26013 | No | Other reasons: The patient discontinued the medication and requested for withdrawal from the study of his own accord  - | Did not complete the clinical study: Other reasons (the patient discontinued the medication and requested for withdrawal from the study of his own accord); medication and post-medication safety evaluations were carried out, but post-medication efficacy evaluation was not carried out. | No | Yes |

###### Table 9.1-5 List of subjects of the Sodium Oligomannate capsule group 900mg who did not enter PPS

| **Center** | **Subject**  **Number** | **Completed the study** | **Reason for dropout** | **Reason for non-entry to PPS** | **Whether the subject entered FAS** | **Whether the subject entered SS** |
| --- | --- | --- | --- | --- | --- | --- |
| 1 | 01035 | No | Withdrawal of informed consent form by subject | Did not complete the clinical study: The subject withdrew the informed consent form. | Yes | Yes |
| 4 | 04007 | No | Lost to follow-up | Did not complete the clinical study: Lost to follow-up. | Yes | Yes |
| 5 | 05005 | No | Occurrence of adverse events | Did not complete the clinical study: Occurrence of adverse events. | Yes | Yes |
| 6 | 06001 | Yes |  | There was a deviation in the understanding and implementation of the scale assessment method by the said center, which led to distortion of scale scores. Therefore, efficacy analysis could not be carried out. | No | Yes |
| 6 | 06002 | Yes |  | There was a deviation in the understanding and implementation of the scale assessment method; amendments were made to the scale scores largely at will, which led to distortion of scale scores. Therefore, efficacy analysis could not be carried out. | No | Yes |
| 7 | 07004 | No | Withdrawal of informed consent form by subject | Did not complete the clinical study: The subject withdrew the informed consent form. | Yes | Yes |
| 7 | 07005 | No | Withdrawal of informed consent form by subject | Did not complete the clinical study: The subject withdrew the informed consent form. | Yes | Yes |
| 9 | 09008 | Yes |  | There was a gap of 15 days between the final medication date and final visit date, which might have affect the efficacy evaluation of the study drug; this was deemed as a serious violation of the protocol. | Yes | Yes |
| 13 | 13001 | No | Withdrawal of informed consent form by subject | Did not complete the clinical study: The subject withdrew the informed consent form. | Yes | Yes |
| 13 | 13002 | No | Allergic to Sodium Oligomannate capsule | Did not complete the clinical study: Allergic to the Sodium Oligomannate capsule. | Yes | Yes |
| 14 | 14002 | No | The subject developed serious complications  or experienced a worsening of condition, which required emergency measures to be taken. | Did not complete the clinical study: The subject developed serious complications or experienced a worsening of condition during the study period, which required emergency measures to be taken; medication and post-medication safety evaluations were carried out, but post-medication efficacy evaluation was not carried out. | No | Yes |
| 14 | 14007 | No | Other reasons: Due to the illness, the subject preferred to  stay at home and did not want to go out; the subject lost his temper easily and did not  listen to the counsel of his family members; there was a decline in his daily life abilities.  Therefore, the subject was unable to go to the hospital for visits. | Did not complete the clinical study: Other reasons (due to the illness, the subject preferred to stay at home and did not want to go out; the subject lost his temper easily and did not listen to the counsel of his family members; there was a decline in his daily living abilities. Therefore, the subject was unable to go to the hospital for visits.) | Yes | Yes |
| 16 | 16001 | No | Poor compliance with the protocol | Did not complete the clinical study: The subject showed poor compliance with the protocol. | Yes | Yes |
| 16 | 16003 | No | Withdrawal of informed consent form by subject | Did not complete the clinical study: The subject withdrew the informed consent form. | Yes | Yes |
| 20 | 20006 | Yes |  | The concomitant use of "oxiracetam" during the double-blind treatment period might have affected the efficacy evaluation of the study drug; this was deemed as a serious violation of the protocol. | Yes | Yes |

## 9.2 Serious deviations from the study protocol

In total, 1 2 subjects had serious deviations from the study protocol during the study. The various cases of deviation are described as follows:

three (02016, 09007 and 10001) subjects had poor compliance to the protocol; this was deemed as a serious violation of the study protocol.

four (09006, 09008, 12006 and 17003) subjects seriously exceeded the visit window for their final visit; this was deemed as a serious violation of the study protocol.

one (09007) subject did not meet the inclusion criteria; this was deemed as a serious violation of the study protocol.

one (20006) subject took concomitant medication during the treatment period, which might have affected the efficacy assessment of the study drug; this was deemed as a serious violation of the study protocol.

three (15008, 17004 and 21006) subjects seriously violated the inclusion and exclusion criteria, and did not complete the study; this was deemed as a serious violation of the study protocol.

one (23002) subject's V0-related examination was an external institution's results and he did not complete the study; this was deemed as a serious violation of the study protocol. See Table 9.2-1 for details.

###### Table 9.2-1 Protocol deviations, N (%a)

| Protocol deviation a | Total population (N=255) | |
| --- | --- | --- |
|  | N | % |
| Poor compliance with the protocol | 3 | 1.18% |
| Deviation in visit time | 4 | 1.57% |
| Violation of inclusion criteria | 1 | 0.39% |
| Concomitant medication | 1 | 0.39% |
| Serious violation of the inclusion and exclusion criteria | 3 | 1.18% |
| V0-related examination was an external institution's results | 1 | 0.39% |

a. The percentages were based on the number of enrolled patients

**9.3 Demographics and other baseline characteristics**

In the general data of subjects in the FAS population at baseline, the data related to demographic characteristics and medical history was distributed evenly among the three groups with good comparability (P>0.05).See Tables 9.3-1, 2 and 3 for details.

The results of vital signs examination of patients in the three groups were consistent; after applying statistical analysis, we could ascertain that the vital signs of patients had no effect on the clinical action of the study drug and placebo.

In the physical examination, there were anomalies in some indicators of a small number of patients, and this was distributed evenly among the three groups with good comparability. The anomalies in the physical examination of subjects in the three groups were as follows: general condition (one subject in the 900mg group), skin (one subject in the placebo group and one subject in the 600mg group), eyes (three subjects in the placebo group), heart (three subjects in the 900mg group), abdomen (one subject in the 600mg group), spine (one subject in the placebo group), and limbs (one subject in the 600mg group).See the "statistical analysis report" in Table 8.1.4 for details.

The clinical features of the respective indicators of ECG examination and laboratory tests conducted on patients in the three groups were essentially consistent, with good comparability. See the“statistical analysis report”in Tables 8.3.4, 5 for details.

###### Table 9.3-1Comparison of the demographics of subjects in the three groups (FAS)

| **Item** | **Placebo**  **N=83** | **Sodium Oligomannate capsule 600mg**  **N=76** | **Sodium Oligomannate capsule 900mg**  **N=83** | **P value** |
| --- | --- | --- | --- | --- |
| Gender |  |  |  | 0.5189 |
| Male | 31(37.35%) | 35(46.05%) | 33(39.76%) |  |
| Female | 52(62.65%) | 41(53.95%) | 50(60.24%) |  |
| Age (years old) |  |  |  | 0.9957 |
| Mean (SD) | 70.34(8.13) | 70.26(8.40) | 70.39(8.51) |  |
| Race |  |  |  | 0.7638 |
| Han | 83(100.00%) | 75(98.68%) | 82(98.80%) |  |
| Minority group | 0(0.00%) | 1(1.32%) | 1(1.20%) |  |
| Height (cm) |  |  |  | 0.5176 |
| Mean (SD) | 161.69(8.78) | 163.12(7.15) | 162.52(7.64) |  |
| Weight (kg) |  |  |  | 0.1986 |
| Mean (SD) | 57.85(10.40) | 60.67(9.88) | 58.64(10.03) |  |
| Education level |  |  |  | 0.8007 |
| Primary school | 31(37.35%) | 22(28.95%) | 27(32.53%) |  |
| Above primary school | 52(62.65% ) | 54(71.06% ) | 56(67.47% ) |  |

###### Table 9.3-2 Comparison of data related to medical history of subjects in the three groups (FAS)

| **Item** | **Placebo**  **N=83** | **Sodium Oligomannate capsule 600mg**  **N=76** | **Sodium Oligomannate capsule 900mg**  **N=83** | **P value** |
| --- | --- | --- | --- | --- |
| Whether drug therapy was used in the past one month |  |  |  | 0.1156 |
| No | 70(84.34%) | 71(93.42%) | 69(83.13%) |  |
| Yes | 13(15.66%) | 5(6.48%) | 14(16.87%) |  |
| Whether there were comorbid diseases |  |  |  | 0.2619 |
| No | 44(53.01%) | 47(61.84%) | 54(65.06%) |  |
| Yes | 39(46.99%) | 29(38.16%) | 29(34.94%) |  |
| Whether there was any family history |  |  |  | 0.4001 |
| No | 77(92.77%) | 71(93.42%) | 73(87.95%) |  |
| Yes | 6(7.23%) | 5(6.48%) | 10(12.05%) |  |
| Whether there was any history of drug allergy |  |  |  | 0.3619 |
| No | 80(96.39%) | 72(94.74%) | 82(98.80%) |  |
| Yes | 3(3.61%) | 4(5.26%) | 1(1.20%) |  |
| Whether there was any history of drug abuse |  |  |  |  |
| No | 83(100.00%) | 76(100.00%) | 83(100.00%) |  |
| Yes | 0(0.00%) | 0(0.00%) | 0(0.00%) |  |
| Whether there was any history of alcohol abuse |  |  |  | 1.0000 |
| No | 83(100.00%) | 76(100.00%) | 82(98.80%) |  |
| Yes | 0(0.00%) | 0(0.00%) | 1(1.20%) |  |

###### Table 9.3-3 Comparison of vital signs of subjects in the three groups (FAS)

| **Item** | **Placebo**  **N=83** | **Sodium Oligomannate capsule 600mg**  **N=76** | **Sodium Oligomannate capsule 900mg**  **N=83** | **P value** |
| --- | --- | --- | --- | --- |
| Body temperature (℃) |  |  |  | 0.7791 |
| Mean (SD) | 36.46(0.25) | 36.49(0.25) | 36.47(0.25) |  |
| Respiration (times/second) |  |  |  | 0.5363 |
| Mean (SD) | 18.35(1.96) | 18.46(2.07) | 18.11(2.10) |  |
| Systolic blood pressure (mmHg) |  |  |  | 0.6489 |
| Mean (SD) | 125.83(12.01) | 124.20(10.82) | 125.53(10.24) |  |
| Diastolic blood pressure (mmHg) |  |  |  | 0.2404 |
| Mean (SD) | 75.67(6.99) | 77.62(7.41) | 76.12(8.14) |  |
| Pulse rate (times/second) |  |  |  | 0.8479 |
| Mean (SD) | 74.40(10.00) | 74.72(8.86) | 73.89(8.74) |  |

## 9.4 Drug exposure situation and medication compliance

The compliance in the study was analyzed using the time of exposure of subjects in the SS population to the study and the receipt of study drug treatment.

In this study, the study cycle was 28 weeks (4-week run-in phase and 24-week treatment period), and four subjects (two in the placebo group, one in the 600mg group and one in the 900mg group) seriously exceeded the window for their visit (there was a gap of 15 days between the final medication date and final visit date).

The average study duration of the placebo group, 600mg group and 900mg group was 196.73 days, 187.92 days and 196.36 days, respectively; the study duration of patients in the three groups was relatively similar, and all of them had good medication compliance (80%-120%). See Table 9.4-1.

###### Table 9.4-1 Subjects' exposure and compliance (SS)

| **Item** |  |  |  |
| --- | --- | --- | --- |
| Study duration (days) | **Placebo** | **Sodium Oligomannate capsule 600mg** | **Sodium Oligomannate capsule 900mg** |
| N (Missing) | 85(0) | 84(0) | 86(0) |
| Mean (SD) | 196.73(34.41) | 187.92(52.61) | 196.36(31.24) |
| Min, Max | 60,254 | 36,337 | 79,271 |
| Md (Q3-Q1) | 203.00(9.00) | 202.50(9.50) | 202.00(10.00) |
| Medication compliance |  |  |  |
| >120% | 0(0.00%) | 0(0.00%) | 0(0.00%) |
| 80%-120% | 77(98.72%) | 68(97.14%) | 75(100.00%) |
| <80% | 1(1.28%) | 2(2.86%) | 0(0.00%) |
| Total | 78 | 70 | 75 |

Note: The medication compliance data of 32 subjects was missing as they did not complete the study. Subjects with subject numbers 10001, 9007 and 2016 were not included into PPS as their compliance was less than 80%.

9.5 Concomitant medication

The analysis of concomitant medication was carried out on all the enrolled patients.

During the study, the incidence rate of concomitant medication existing in the placebo group, 600mg group and 900mg group was 56.47% (48 patients), 46.43% (39 patients) and 46.51% (40 patients), respectively.

There were 11 subjects who took anti-dementia or anti-psychotic drugs concomitantly during the study, and only one of them (patient number 20006 in the 900mg group) took concomitant medication (oxiracetam) during the double-blind treatment period, which might affect the judgment of the efficacy of the study drug. There were seven patients who took concomitant medication during the run-in phase but discontinued the said concomitant medication before randomization. Three patients began taking concomitant medication when they withdrew from the study after experiencing SAEs, hence these were deemed to have no effect on the study drug.

See Table 9.5-1 for the frequency of use of concomitant medication for each body system during the study. See the "statistical analysis report" in Table 9.2.3 for the list of all subjects who took concomitant medication.

###### Table 9.5-1 Frequency of use of different concomitant medication for each body system (SS)

|  | **Placebo**  **(N=85)** | | |  | **Sodium Oligomannate capsule 600mg**  **(N=84)** | | |  | **Sodium Oligomannate capsule**  **900mg**  **(N=86)** | | |
| --- | --- | --- | --- | --- | --- | --- | --- | --- | --- | --- | --- |
| **Item** | Number of cases | Number of incidents | Incidence rate (%) |  | Number of cases | Number of incidents | Incidence rate  (%) |  | Number of cases | Number of incidents | Incidence rate  (%) |
| Total | 48 | 133 | 56.47 |  | 39 | 106 | 46.43 |  | 40 | 130 | 46.51 |
| Central nervous system drugs | 23 | 37 | 27.06 |  | 15 | 21 | 17.86 |  | 20 | 34 | 23.26 |
| Autonomic nervous system drugs | 0 | 0 | 0.00 |  | 0 | 0 | 0.00 |  | 5 | 6 | 5.81 |
| Circulatory system drugs | 24 | 44 | 28.24 |  | 19 | 34 | 22.62 |  | 27 | 41 | 31.40 |
| Respiratory system drugs | 1 | 1 | 1.18 |  | 1 | 1 | 1.19 |  | 1 | 1 | 1.16 |
| Digestive system drugs | 2 | 2 | 2.35 |  | 6 | 7 | 7.14 |  | 1 | 1 | 1.16 |
| Urinary system drugs | 1 | 2 | 1.18 |  | 0 | 0 | 0.00 |  | 1 | 1 | 1.16 |
| Drugs that affect the blood and hematopoietic system | 5 | 5 | 5.88 |  | 1 | 1 | 1.19 |  | 4 | 4 | 4.65 |
| Hormones and related drugs | 6 | 8 | 7.06 |  | 6 | 9 | 7.14 |  | 4 | 6 | 4.65 |
| Antimicrobial drugs | 5 | 6 | 5.88 |  | 6 | 9 | 7.14 |  | 3 | 7 | 3.49 |
| Vitamin drugs | 4 | 5 | 4.71 |  | 2 | 2 | 2.38 |  | 4 | 5 | 4.65 |
| Anti-aging drugs and drugs for some disease of old people | 0 | 0 | 0.00 |  | 1 | 1 | 1.19 |  | 1 | 1 | 1.16 |
| Drugs for water, electrolyte and acid-base balance | 0 | 0 | 0.00 |  | 1 | 1 | 1.19 |  | 1 | 1 | 1.16 |
| Standby drugs of all clinical departments | 0 | 0 | 0.00 |  | 1 | 1 | 1.19 |  | 0 | 0 | 0.00 |
| Drugs for other purposes | 18 | 23 | 21.18 |  | 12 | 19 | 14.29 |  | 13 | 22 | 15.12 |

# 10. Safety evaluation results

The safety analysis was performed on the SS population.

**10.1 Overall safety evaluation results**

**10.1.1 Adverse events**

In this study, the incidence rate of adverse events in the placebo group, 600mg group and 900mg group was 41.18%, 36.90% and 27.91%, respectively. The incidence rate of adverse events related to the study drug was 3.53%, 5.59% and 3.49%, respectively. The incidence rate of adverse events leading to dropouts was 1.18%, 3.57% and 3.49%, respectively. The adverse events in the three groups were mostly mild adverse events.

See Table 10.1.1-1 for the summary of adverse events. See Tables 10.1.1-2, 3 and 4 for the list of adverse events leading to dropouts. See “statistical analysis report” in Table 9.2.2.1 for the list of all cases of adverse events.

###### Table 10.1.1-1 Summary of adverse events (SS)

|  | **Placebo  (N=85)** | | |  | **Sodium Oligomannate capsule 600mg  (N=84)** | | |  | **Sodium Oligomannate capsule 900mg  (N=86)** | | |
| --- | --- | --- | --- | --- | --- | --- | --- | --- | --- | --- | --- |
| **Item** | **Number of cases** | **Number of incidents** | **Incidence rate (%)** |  | **Number of cases** | **Number of incidents** | **Incidence rate (%)** |  | **Number of cases** | **Number of incidents** | **Incidence rate (%)** |
| All adverse events | 35 | 66 | 41.18 |  | 31 | 64 | 36.90 |  | 24 | 51 | 27.91 |
| Adverse events related to the study drug* | 3 | 5 | 3.53 |  | 5 | 12 | 5.95 |  | 3 | 3 | 3.49 |
| Serious adverse events | 6 | 6 | 7.06 |  | 5 | 6 | 5.95 |  | 3 | 3 | 3.49 |
| Serious adverse events related to the study drug* | 0 | 0 | 0.00 |  | 1 | 1 | 1.19 |  | 0 | 0 | 0.00 |
| Adverse events leading to dropouts | 1 | 1 | 1.18 |  | 3 | 5 | 3.57 |  | 3 | 3 | 3.49 |

Note: *Related to the study drug: Includes those who are "definitely related", "possibly related", "probably related" and "suspected to be related" to the study drug.

###### Tables 10.1.1-2 List of adverse events leading to dropouts in the placebo group

| **Center number** | **Subject number** | **System**  **name** | **Canonical**  **name** | **Severity**  **level** | **Relationship with the study drug** | **Measures adopted** | **Outcome** | **Start**  **date** | **End**  **date** | **Whether still present** | **Whether it is SAE** |
| --- | --- | --- | --- | --- | --- | --- | --- | --- | --- | --- | --- |
| 18 | 18009 | All kinds of nervous system disorders | Cerebral infarction | Mild | Probably unrelated | Discontinue study drug + add concomitant medication + hospitalization/extended hospitalization | Subsided | 01/28/13 | 02/11/13 | No | Yes |

###### Table 10.1.1-3 List of adverse events leading to dropouts in the Sodium Oligomannate capsule 600mg group

| **Center number** | **Subject number** | **System name** | **Canonical name** | **Severity level** | **Relationship with the study drug** | **Measures adopted** | **Outcome** | **Start**  **date** | **End**  **date** | **Whether still present** | **Whether it is SAE** |
| --- | --- | --- | --- | --- | --- | --- | --- | --- | --- | --- | --- |
| 1 | 1028 | Psychiatric disorders | Behavioral and psychological symptoms of dementia | Moderate | Probably unrelated | Discontinue study drug + add concomitant medication + hospitalization/extended hospitalization | Subsided | 11/19/12 | 03/15/13 | No | Yes |
| 1 | 1028 | All kinds of injuries, poisoning and complications | Subdural hemorrhage | Serious | Probably unrelated | Discontinue study drug + administered non-drug therapy + hospitalization/extended hospitalization | Subsided | 12/11/12 | 12/19/12 | No | Yes |
| 14 | 14004 | Benign and malignant tumors of unknown nature (including cystic and polypoid tumors) | Lung cancer | Moderate | Unrelated | Discontinue study drug + add concomitant medication + hospitalization/extended hospitalization | Stable | 06/04/12 |  | Yes | Yes |
| 14 | 14002 | Gastrointestinal disorders | Abdomen pain | Moderate | Probably unrelated | Discontinue the study drug | Whether still present | 01/18/12 |  | Yes | No |
| 23 | 23007 | Psychiatric disorders | Irritation | Mild | Possibly related | Discontinue the study drug | Subsided | 07/31/12 | 09/10/12 | No | No |
| 23 | 23007 | Psychiatric disorders | Mid-term insomnia | Mild | Possibly related | Discontinue the study drug | Subsided | 07/31/12 | 09/10/13 | No | No |

###### Table 10.1.1-4 List of adverse events leading to dropouts in the Sodium Oligomannate capsule 900mg group

| **Center number** | **Subject number** | **System name** | **Canonical**  **name** | **Severity level** | **Relationship with the study drug** | **Measures adopted** | **Outcome** | **Start**  **date** | **End**  **date** | **Whether still present** | **Whether it is SAE** |
| --- | --- | --- | --- | --- | --- | --- | --- | --- | --- | --- | --- |
| 5 | 5005 | All kinds of nervous system disorders | Cerebral hemorrhage | Serious | Unrelated | Discontinue study drug + add concomitant medication + hospitalization/extended hospitalization | Stable | 11/06/12 |  | Yes | Yes |
| 13 | 13002 | Skin and subcutaneous tissue diseases | Rash | Moderate | Probably unrelated | Discontinue the study drug | Whether still present | 07/09/12 |  | Yes | No |
| 14 | 14002 | Gastrointestinal disorders | Abdomen pain | Moderate | Probably unrelated | Discontinue the study drug | Whether still present | 01/18/12 |  | Yes | No |

**10.1.2 Drug-related adverse events**

The incidence rate of drug-related adverse events during the study was 3.53% for the placebo group, 5.95% for the 600mg group, and 3.49% for the 900mg group, and they were all presented as mild to moderate adverse events. See Table 10.1.2-1 for the incidence rate of adverse events related to the various systems and drug.

###### Table 10.1.2-1 Incidence rate of adverse events related to the various systems and drug (SS)

| **Item** | **Placebo**  **(N=85)** | | |  | **Sodium Oligomannate capsule 600mg**  **(N=84)** | | |  | **Sodium Oligomannate capsule 900mg**  **(N=86)** | | |
| --- | --- | --- | --- | --- | --- | --- | --- | --- | --- | --- | --- |
|  | **Number of cases** | **Number of incidents** | **Incidence rate (%)** |  | **Number of cases** | **Number of incidents** | **Incidence rate (%)** |  | **Number of cases** | **Number of incidents** | **Incidence rate (%)** |
| Ear and labyrinth disorders | 1 | 1 | 1.18 |  | 1 | 1 | 1.19 |  | 0 | 0 | 0.00 |
| Tinnitus | 1 | 1 | 1.18 |  | 0 | 0 | 0.00 |  | 0 | 0 | 0.00 |
| Vertigo | 0 | 0 | 0.00 |  | 1 | 1 | 1.19 |  | 0 | 0 | 0.00 |
| Gastrointestinal disorders | 3 | 4 | 3.53 |  | 0 | 0 | 0.00 |  | 1 | 1 | 1.16 |
| Diarrhea | 1 | 1 | 1.18 |  | 0 | 0 | 0.00 |  | 0 | 0 | 0.00 |
| Bloating | 1 | 2 | 1.18 |  | 0 | 0 | 0.00 |  | 0 | 0 | 0.00 |
| Dry mouth | 1 | 1 | 1.18 |  | 0 | 0 | 0.00 |  | 1 | 1 | 1.16 |
| All kinds of injuries, poisoning and complications | 0 | 0 | 0.00 |  | 1 | 1 | 1.19 |  | 0 | 0 | 0.00 |
| Scratched | 0 | 0 | 0.00 |  | 1 | 1 | 1.19 |  | 0 | 0 | 0.00 |
| All kinds of musculoskeletal and connective tissue disorders | 0 | 0 | 0.00 |  | 1 | 1 | 1.19 |  | 0 | 0 | 0.00 |
| Weak muscles | 0 | 0 | 0.00 |  | 1 | 1 | 1.19 |  | 0 | 0 | 0.00 |
| All kinds of nervous system disorders | 0 | 0 | 0.00 |  | 1 | 1 | 1.19 |  | 0 | 0 | 0.00 |
| Dizziness | 0 | 0 | 0.00 |  | 1 | 1 | 1.19 |  | 0 | 0 | 0.00 |
| Psychiatric disorders | 0 | 0 | 0.00 |  | 2 | 5 | 2.38 |  | 0 | 0 | 0.00 |
| Mental and behavioral symptoms of dementia | 0 | 0 | 0.00 |  | 1 | 1 | 1.19 |  | 0 | 0 | 0.00 |
| Irritation | 0 | 0 | 0.00 |  | 1 | 2 | 1.19 |  | 0 | 0 | 0.00 |
| Insomnia | 0 | 0 | 0.00 |  | 1 | 2 | 1.19 |  | 0 | 0 | 0.00 |
| Reproductive system and breast disorders | 0 | 0 | 0.00 |  | 0 | 0 | 0.00 |  | 1 | 1 | 1.16 |
| Hyperplasia of mammary glands | 0 | 0 | 0.00 |  | 0 | 0 | 0.00 |  | 1 | 1 | 1.16 |
| Respiratory, thoracic and mediastinal disorders | 0 | 0 | 0.00 |  | 1 | 1 | 1.19 |  | 0 | 0 | 0.00 |
| Stuffy nose | 0 | 0 | 0.00 |  | 1 | 1 | 1.19 |  | 0 | 0 | 0.00 |
| Skin and subcutaneous tissue diseases | 0 | 0 | 0.00 |  | 1 | 2 | 1.19 |  | 1 | 1 | 1.16 |
| Rash | 0 | 0 | 0.00 |  | 0 | 0 | 0.00 |  | 1 | 1 | 1.16 |
| Itching | 0 | 0 | 0.00 |  | 1 | 2 | 1.19 |  | 0 | 0 | 0.00 |

### 10.1.3 Serious adverse events

In the process of this study, there were 14 patients (15 cases) who experienced serious adverse events; six of them were in the placebo group (six cases, 7.06%), five of them were in the 600mg group (six cases, 5.95%) and three of them were in the 900mg group (three cases, 3.49%).Based on the judgment of the clinical investigator, ten of the SAE cases (01009, 05009 and 14004 in the 600mg group; 02005, 05005 and 20014 in the 900mg group; 02009, 05004, 16002 and 22004 in the placebo group) were definitely unrelated to the drug, four of the SAE cases (01028 and 21008 in the 600mg group; 12013 and 18009 in the placebo group) were probably unrelated to the drug, and one SAE case (01028 in the 600mg group) was probably related to the drug.

The SAEs are described in details as follows:

1. Patient number 01009, female, 83 years old, was officially enrolled on December 27, 2011 and took the first dose of the study drug for the first time on that day. On May 25, 2012, she accidentally fell and was sent to the emergency department of the Jingan Branch of Huashan Hospital Affiliated with Fudan University, and was diagnosed with 12 compression fractures of the chest. On May 28, she was admitted to the Orthopedic ward of Huashan Hospital and received surgical treatment. She was later discharged on June 4.This event was reported to the investigator on June 25, and in the clinical investigator's judgment, this adverse event was definitely unrelated to the drug.
2. Patient number 01028, female, 81 years old, was officially enrolled on July 26 2012 and took the first dose of the study drug for the first time on that day. On November 19 2012, she was admitted to the Mental Health Center Affiliated with Mental Health Center, Shanghai Jiao Tong University School of Medicine for mental combined with behavioral disorders. She then discontinued the study drug and withdrew from the study. After receiving treatment using zyprexa 5mg, QN; ebixa 5mg, QD; estazolam 1mg, QN, her condition improved and she was discharged on March 15 2013.This event was reported to the investigator on November 19 2012, and in the clinical investigator's judgment, this adverse event was probably related to the drug. On December 11 2012, the patient suddenly had subdural hemorrhage and was transferred to Xuhui District Central Hospital. Her condition improved after surgery and she was discharged on December 19 2012. This event was reported to the investigator on December 11 2012, and in the clinical investigator's judgment, this adverse event was probably unrelated to the drug. After being discharged, the patient took zyprexa 5mg, QN; aricept 5mg, QD; memantine 5mg, QD every day, and her physical and mental health gradually stabilized.
3. Patient number 02005, male, 81 years old, was officially enrolled on March 26, 2012. He completed V3 (Week 12 after treatment) visit on June 21, and he was found to have a history of left varicose veins for 16 years and had never gone for any treatment. On June 20, the back of his left foot was swollen, and it was diagnosed as cellulitis at the back of his left foot. He was admitted to the Second Hospital Affiliated with Chongqing Medical University, where he received anti-inflammatory treatment using penicillin V potassium and cefathiamidine. He was then diagnosed clinically to have recovered and was discharged. This event was reported to the investigator on June 21, and in the clinical investigator's judgment, this adverse event was definitely unrelated to the drug.
4. Patient number 02009, male, 81 years old, was officially enrolled on May 24, 2012 and took the first dose of the study drug for the first time on that day. On August 13, he was found to have drooping eyelid on the left eye; there was no pain and this did not affect his life, hence he did not pay much attention to it. However, on September 4, his family member noticed the drooping eyelid when he went to the hospital for follow-up visit, and he eventually admitted to the Second Hospital Affiliated with Chongqing Medical University on that day. Upon examination, it was found that the patient did not restrain his diet and his glucose tolerance level was abnormal; diabetic oculomotor nerve palsy was considered, and the patient was given diet control and neurotrophic therapy. The patient was discharged on September 19. His eyelid disease improved subsequently until it became normal again. During this period, he continued to take the study drug. This event was reported to the investigator on September 4, and in the clinical investigator's judgment, this adverse event was definitely unrelated to the drug.
5. Patient number 05004, female, 79 years old, was officially enrolled on July 17, 2012 and took the first dose of the study drug for the first time on that day. On November 28, she was admitted to the First Affiliated Hospital of Harbin Medical University due to "acute gastrointestinal dysfunction", and she was given levofloxacin and inverted sugar electrolyte injection for symptomatic treatment. She then recovered and was discharged on November 30.This event was reported to the investigator on January 10, 2013, and in the clinical investigator's judgment, this adverse event was definitely unrelated to the drug.
6. Patient number 05005, male, 77 years old, was officially enrolled on August 2, 2012 and took the first dose of the study drug for the first time on that day. On November 6, he was admitted to the ICU ward of the First Affiliated Hospital of Harbin Medical University due to "cerebral hemorrhage". He discontinued the study drug and withdrew from the study and received symptomatic treatment. On November 9, he was transferred to the Brain Surgery Department to continue with the treatment, and he was given mannitol, albumin, cephalosporins and oxiracetam for treatment. He was then discharged on December 6, and he was still in a state of narcosis at that time. On January 10, 2013, his family said that his condition was stable with no complications, but there was no improvement in his state of consciousness. The said patient withdrew from the study. This event was reported to the investigator on January 10, 2013, and in the clinical investigator's judgment, this adverse event was definitely unrelated to the drug.
7. Patient number 05009, female, 85 years old, was officially enrolled on September 10, 2012 and took the first dose of the study drug for the first time on that day. During the fourth visit on February 22, 2013, her family said that she was admitted to the Fourth Hospital of Harbin Medical University on January 28, 2013 due to "acute intestinal hernia", and she was discharged on February 2 after getting better. The family also said that the patient had intestinal hernia one year ago. This event was reported to the investigator on February 22, 2013, and in the clinical investigator's judgment, this adverse event was definitely unrelated to the drug.
8. Patient number 12013, female, 82 years old, was officially enrolled on January 22, 2013 and took the first dose of the study drug for the first time on that day. She had been having ongoing knee pain for no reason for the past 20 years. On June 6, she was admitted to Hubei Hospital of Traditional Chinese Medicine due to "acute onset of osteoarthritis in both knees" and received the corresponding treatment. Her condition then improved and she was discharged on June 19. During the period of hospitalization, the dose of the study drug was not adjusted at all, and the study drug was taken as per normal. This event was reported to the investigator on June 28, 2013, and in the clinical investigator's judgment, this adverse event was probably unrelated to the drug.
9. Patient number 14004, female, 78 years old, was officially enrolled on February 13, 2012 and took the first dose of the study drug for the first time on that day. The patient had chest pain for more than three months, but she did not tell her family. On June 4, she was admitted to Sir Run Run Shaw Hospital Affiliated with Zhejiang University School of Medicine due to "chest lump"; she also discontinued the study drug and withdrew from the study. After examination, the possibility of lung cancer was considered. During the telephone follow-up made on June 6 at that time, the patient and her family did not inform about this event. The patient was discharged on June 15, and after repeated examinations by many hospitals, the possibility of lung cancer was still being considered. However, her family clearly rejected further examination to confirm the diagnosis and rejected the relevant treatment such as surgery or chemotherapy due to her old age, and went for TCM treatment instead. We only knew about this event when we contacted her family on July 7. This event was reported to the investigator on July 7, 2012, and in the clinical investigator's judgment, this adverse event was definitely unrelated to the drug.
10. Patient number 16002, male, 68 years old, was officially enrolled on September 24, 2012 and took the first dose of the study drug for the first time on that day. In the second visit held on November 2, the patient informed the investigator that he was admitted to Chongqing Ninth People Hospital on October 20 due to "acute appendicitis attack", and he was discharged on October 27 with no sequelae. During the hospitalization period, he discontinued the study drug. His family said that the patient took cephalosporins during hospitalization, but they do not know the quantity and other drugs that he had taken. Medical documents related to this hospitalization were also not provided, and the patient did not contact the investigator during the hospitalization period. After being discharged, the patient continued taking the study drug. This event was reported to the investigator on November 2, 2012, and in the clinical investigator's judgment, this adverse event was definitely unrelated to the drug.
11. Patient number 18009, male, 77 years old, was officially enrolled on December 18, 2012 and took the first dose of the study drug for the first time on January 21, 2013. On January 28 (Day 8 of taking the study drug), the patient's family called the investigator to inform us that the patient had a weak upper left limb and requested that the patient discontinue the drug and withdraw from the study. The investigator agreed and requested that the patient go to the hospital for re-examination. Due to travel inconvenience, the patient consulted a local hospital (Yingcheng City People's Hospital) on January 28 and was diagnosed with "right cerebral infarction". His symptoms were completely gone after treatment and he was discharged on February 10.During the hospitalization period, the patient was given aspirin tablets, folic acid tablets and Yindan Xinnaotong capsules for treatment. This event was reported to the investigator on January 28, 2013, and in the clinical investigator's judgment, this adverse event was probably unrelated to the drug.
12. Patient number 20014, female, 77 years old, was officially enrolled on December 24, 2012 and took the first dose of the study drug for the first time on that day. On January 9, 2013, the patient experienced dizziness and flushing, and was diagnosed with high blood pressure at a local hospital. Her blood lipids were also abnormal, and she was admitted on that day and given treatment to control blood pressure and regulate blood lipids: Yilunping 162.5mg, QD and Genketong 20mg, three times a day, as well as Crestor 10mg once at night. Her condition then improved and she was discharged on January 16. This event was reported to the investigator on January 10, 2013, and in the clinical investigator's judgment, this adverse event was probably unrelated to the drug.
13. Patient number 21008, female, 69 years old, was officially enrolled on November 30, 2012 and took the first dose of the study drug for the first time on that day. The patient had irregular chest tightness and palpitation 1.5 years ago, especially at night. Several Holter examinations showed atrial fibrillation and long RR time, but no special treatment was given. On March 29, 2013, another Holter examination showed atrial fibrillation and long RR time, and the local hospital recommended the implantation of a pacemaker. On April 1, the patient was admitted to Shandong Provincial Hospital and went for pacemaker implantation surgery on that day. The surgery was successful, and the aforementioned symptoms completely subsided after the surgery. The patient was discharged after one week of hospitalization, and she no longer experienced such palpitations thereafter. This event was reported to the investigator on May 20, 2013, and in the clinical investigator's judgment, this adverse event was probably unrelated to the drug.
14. Patient number 22004, male, 61 years old, signed the informed consent form on December 7, 2012 and entered screening on December 10. He then passed the screening and was given the drug for the run-in phase. The patient had SAE during the run-in phase, and the details are as such: On December 22, 2012, the patient was caught in heavy snow and plunging temperatures while on holiday, and his legs was covered in the snow. He then had relatively severe chills symptoms and consulted Tangdu Hospital of the Fourth Military Medical University. After administering infusion to protect the stomach and other such measures due to his low body temperature, the symptoms disappeared and the patient then returned to his normal self. This event was reported to the investigator on January 8, 2013, and in the clinical investigator's judgment, this adverse event was definitely unrelated to the drug.

###### Table 10.1.3-1 List of serious adverse events in the placebo group

| **Center** | **Subject number** | **System**  **name** | **Canonical**  **name** | **Severity level** | **Relationship with the study drug** | **Measures adopted** | **Outcome** | **Start**  **date** | **End**  **date** | **Whether still present** | **Whether there is withdrawal from the study due to this** |
| --- | --- | --- | --- | --- | --- | --- | --- | --- | --- | --- | --- |
| 2 | 2009 | All kinds of nervous system disorders | Third cranial nerve palsy | Serious | Unrelated | Add concomitant medication + hospitalization/extended hospitalization | Subsided | 08/13/12 | 10/25/12 | No | No |
| 5 | 5004 | Gastrointestinal disorders | Gastrointestinal motility disorders | Moderate | Unrelated | Add concomitant medication + hospitalization/extended hospitalization | Subsided | 11/28/12 | 11/30/12 | No | No |
| 12 | 12013 | All kinds of musculoskeletal and connective tissue disorders | Osteoarthritis | Moderate | Probably unrelated | Add concomitant medication + hospitalization/extended hospitalization | Subsided | 06/06/13 | 06/19/13 | No | No |
| 16 | 16002 | Infections and infectious diseases | Appendicitis | Moderate | Unrelated | Change dose/discontinue study drug + add concomitant medication + hospitalization/extended hospitalization | Subsided | 10/20/12 | 10/27/12 | No | No |
| 18 | 18009 | All kinds of nervous system disorders | Cerebral infarction | Mild | Probably unrelated | Discontinue study drug + add concomitant medication + hospitalization/extended hospitalization | Subsided | 01/28/13 | 02/11/13 | No | Yes |
| 21 | 21008 | Various surgical and medical procedures | Pacemaker implantation | Moderate | Probably unrelated | Hospitalization/extended hospitalization | Subsided | 04/01/13 | 04/08/13 | No | No |
| 22 | 22004 | Systemic diseases and various reactions at the administration site | Chills | Mild | Unrelated | Change dose/discontinue study drug + add concomitant medication + administration of non-drug therapy + hospitalization/extended hospitalization | Subsided | 12/22/12 | 12/23/12 | No | No |

###### Table 10.1.3-2 List of serious adverse events in the Sodium Oligomannate capsule 600mg group

| **Center** | **Subject number** | **System name** | **Canonical name** | **Severity level** | **Relationship with the study drug** | **Measures adopted** | **Outcome** | **Start**  **date** | **End**  **date** | **Whether still present** | **Whether there is withdrawal from the study due to this** |
| --- | --- | --- | --- | --- | --- | --- | --- | --- | --- | --- | --- |
| 1 | 1009 | All kinds of injuries, poisoning and complications | Vertebral compression fractures | Serious | Unrelated | Administration of non-drug therapy + hospitalization/extended hospitalization | Subsided | 05/25/12 | 06/04/12 | No | No |
| 1 | 1028 | Psychiatric disorders | Behavioral and psychological symptoms of dementia | Moderate | Probably related | Discontinue study drug + add concomitant medication + hospitalization/extended hospitalization | Subsided | 11/19/12 | 03/15/13 | No | Yes |
| 1 | 1028 | All kinds of injuries, poisoning and complications | Subdural hemorrhage | Serious | Probably unrelated | Discontinue study drug + administered non-drug therapy + hospitalization/extended hospitalization | Subsided | 12/11/12 | 12/19/12 | No | Yes |
| 5 | 5009 | Gastrointestinal disorders | Intestinal hernia | Serious | Unrelated | Add concomitant medication + hospitalization/extended hospitalization | Subsided | 01/28/13 | 02/06/13 | No | No |
| 14 | 14004 | Benign and malignant tumors of unknown nature (including cystic and polypoid tumors) | Lung cancer | Moderate | Unrelated | Discontinue study drug + add concomitant medication + hospitalization/extended hospitalization | Stable | 06/04/12 |  | Yes | Yes |
| 21 | 21008 | Various surgical and medical procedures | Pacemaker implantation | Moderate | Probably unrelated | Hospitalization/extended hospitalization | Subsided | 04/01/13 | 04/08/13 | No | No |

###### Table 10.1.3-3 List of serious adverse events in the Sodium Oligomannate capsule 900mg group

| **Center** | **Subject number** | **System name** | **Canonical name** | **Severity level** | **Relationship with the study drug** | **Measures adopted** | **Outcome** | **Start**  **date** | **End**  **date** | **Whether still present** | **Whether there is withdrawal from the study due to this** |
| --- | --- | --- | --- | --- | --- | --- | --- | --- | --- | --- | --- |
| 2 | 2005 | Infections and infectious diseases | Cellulitis | Serious | Unrelated | Add concomitant medication + hospitalization/extended hospitalization | Subsided | 06/20/12 | 06/28/12 | No | No |
| 5 | 5005 | All kinds of nervous system disorders | Cerebral hemorrhage | Serious | Unrelated | Discontinue study drug + add concomitant medication + hospitalization/extended hospitalization | Stable | 11/06/12 |  | Yes | Yes |
| 20 | 20014 | Vascular and lymphatic diseases | Hypertension | Serious | Unrelated | Add concomitant medication + hospitalization/extended hospitalization | Subsided | 01/09/13 | 01/21/13 | No | No |

**10.1.4 Vital signs and physical examination**

During the study, the subjects' vital signs (body temperature, respiration, heart rate, systolic blood pressure and diastolic blood pressure) were checked before and after treatment. The results are as follows: As compared with the baselines of the respective groups, there were no abnormal changes in the body temperature, respiration and systolic blood pressure of subjects in the three groups. There were also no intergroup differences. In terms of heart rate, there was a reduction of two beats in the 600mg group at Week 12 as compared to baseline (P<0.05).In terms diastolic blood pressure, there were significant differences (P<0.05) among the three groups at Week 4, as well as significant differences (P<0.05) in the changes as compared to baseline of the three groups at Week 24. The change in the placebo group, 600mg group and 900mg group as compared to baseline was 0.22mmHg, -2.03mmHg and 2.00mmHg, respectively, and there was an increment of 2mmHg (P<0.05) in the 900mg group at Week 24 of treatment as compared to baseline.

The results of the subjects' physical examination (general condition, skin, lymph nodes, neck, eye, ear, nose, throat, heart, lungs, abdomen, spine, limbs and nervous system) conducted before and after treatment in the study are consolidated as follows: There were no abnormal changes in the lymph nodes, neck, ear, nose, throat and lungs of the subjects in the three groups.

The abnormal conditions are summarized as follows:

Placebo group: three cases at the eyes and one case at the heart;

600mg group: one case on the skin, one case at the abdomen and one case at the limbs;

900mg group: one case on the skin and one case at the nervous system.

### 10.1.5 Laboratory tests and ECG examination

The routine blood test (HGB, RBC, WBC, percentage of neutrophils, percentage of lymphocytes, percentage of monocytes, percentage of eosinophils, percentage of basophils, PLT), blood biochemistry (ALT, AST, TBIL, DBiL, ALP, ALB, BUN, Cr, GGT, GLU), blood coagulation function (PT, APTT, INR), and routine urine test (urine pH, urine protein, leukocytes, red blood cells) were conducted on the three groups at baseline and in the last visit (Week 24 of treatment). The results showed that, the subjects in the three groups had similar percentages of indicators of laboratory tests and ECG examination with clinically significant differences as compared to baseline. See Table 10.1.5-1 for details.

###### Table 10.1.5-1 Negative changes in laboratory test results of patients before and after treatment (SS)

| **Item** | **Placebo group** | | | **Sodium Oligomannate capsule 600mg group** | | | **Sodium Oligomannate capsule 900mg group** | | |
| --- | --- | --- | --- | --- | --- | --- | --- | --- | --- |
|  | **Normal/**  **Abnormal -** | **Normal/**  **Abnormal +** | **Abnormal -/**  **Abnormal +** | **Normal/**  **Abnormal -** | **Normal/**  **Abnormal +** | **Abnormal -/**  **Abnormal +** | **Normal/**  **Abnormal -** | **Normal/**  **Abnormal +** | **Abnormal -/**  **Abnormal +** |
| HGB | 2(2.6%) | 0(0.0%) | 0(0.0%) | 7(9.3%) | 0(0.0%) | 0(0.0%) | 4(5.1%) | 0(0.0%) | 0(0.0%) |
| RBC | 2(2.4%) | 0(0.0%) | 0(0.0%) | 5(6.4%) | 0(0.0%) | 0(0.0%) | 3(3.9%) | 0(0.0%) | 0(0.0%) |
| WBC | 5(6.2%) | 0(0.0%) | 0(0.0%) | 4(5.1%) | 1(1.3%) | 0(0.0%) | 7(8.4%) | 0(0.0%) | 0(0.0%) |
| Percentage of neutrophils | 6(10.2%) | 0(0.0%) | 0(0.0%) | 8(13.6%) | 1(1.7%) | 0(0.0%) | 13(20.3%) | 0(0.0%) | 0(0.0%) |
| Percentage of lymphocytes | 7(10.0%) | 0(0.0%) | 0(0.0%) | 9(13.4%) | 0(0.0%) | 0(0.0%) | 14(21.5%) | 0(0.0%) | 0(0.0%) |
| Percentage of monocytes | 6(7.9%) | 0(0.0%) | 0(0.0%) | 4(5.2%) | 0(0.0%) | 0(0.0%) | 5(6.7%) | 0(0.0%) | 0(0.0%) |
| Percentage of eosinophils | 4(5.6%) | 0(0.0%) | 0(0.0%) | 5(6.7%) | 0(0.0%) | 0(0.0%) | 3(4.2%) | 0(0.0%) | 0(0.0%) |
| Percentage of basophils | 3(3.8%) | 0(0.0%) | 0(0.0%) | 0(0.0%) | 0(0.0%) | 0(0.0%) | 1(1.3%) | 0(0.0%) | 0(0.0%) |
| PLT | 4(5.0%) | 0(0.0%) | 0(0.0%) | 2(2.5%) | 1(1.3%) | 0(0.0%) | 2(2.5%) | 0(0.0%) | 0(0.0%) |
| ALB | 1(1.3%) | 0(0.0%) | 0(0.0%) | 2(2.5%) | 0(0.0%) | 0(0.0%) | 3(3.6%) | 0(0.0%) | 0(0.0%) |
| ALT | 4(4.8%) | 0(0.0%) | 0(0.0%) | 2(2.5%) | 1(1.2%) | 0(0.0%) | 3(3.5%) | 0(0.0%) | 0(0.0%) |
| AST | 0(0.0%) | 0(0.0%) | 0(0.0%) | 4(4.8%) | 0(0.0%) | 0(0.0%) | 3(3.6%) | 0(0.0%) | 0(0.0%) |
| TBIL | 3(3.8%) | 0(0.0%) | 0(0.0%) | 5(6.8%) | 0(0.0%) | 0(0.0%) | 4(4.9%) | 0(0.0%) | 0(0.0%) |
| DBiL | 2(2.6%) | 0(0.0%) | 0(0.0%) | 3(4.2%) | 0(0.0%) | 0(0.0%) | 3(3.7%) | 0(0.0%) | 0(0.0%) |
| ALP | 3(4.1%) | 0(0.0%) | 0(0.0%) | 2(2.6%) | 0(0.0%) | 0(0.0%) | 3(4.0%) | 0(0.0%) | 0(0.0%) |
| BUN | 9(10.5%) | 0(0.0%) | 0(0.0%) | 3(4.4%) | 0(0.0%) | 0(0.0%) | 6(8.0%) | 0(0.0%) | 0(0.0%) |
| Cr | 1(1.2%) | 0(0.0%) | 0(0.0%) | 3(3.9%) | 0(0.0%) | 0(0.0%) | 1(1.3%) | 0(0.0%) | 0(0.0%) |
| GGT | 3(4.0%) | 0(0.0%) | 0(0.0%) | 3(4.2%) | 0(0.0%) | 0(0.0%) | 0(0.0%) | 0(0.0%) | 0(0.0%) |
| GLU | 8(13.3%) | 0(0.0%) | 1(4.5%) | 7(12.3%) | 0(0.0%) | 1(5.0%) | 10(15.4%) | 0(0.0%) | 0(0.0%) |
| PT | 4(6.1%) | 0(0.0%) | 0(0.0%) | 6(10.0%) | 0(0.0%) | 0(0.0%) | 6(9.1%) | 0(0.0%) | 0(0.0%) |
| APTT | 3(5.2%) | 0(0.0%) | 0(0.0%) | 6(10.5%) | 0(0.0%) | 0(0.0%) | 8(12.5%) | 0(0.0%) | 0(0.0%) |
| INR | 2(2.8%) | 0(0.0%) | 0(0.0%) | 0(0.0%) | 0(0.0%) | 0(0.0%) | 0(0.0%) | 0(0.0%) | 0(0.0%) |
| Urine pH | 1(1.2%) | 0(0.0%) | 0(0.0%) | 1(1.4%) | 0(0.0%) | 0(0.0%) | 3(3.9%) | 0(0.0%) | 0(0.0%) |
| Urine protein | 4(5.4%) | 0(0.0%) | 0(0.0%) | 7(9.2%) | 0(0.0%) | 0(0.0%) | 7(8.8%) | 0(0.0%) | 0(0.0%) |
| White blood cell | 9(12.9%) | 0(0.0%) | 0(0.0%) | 3(4.2%) | 0(0.0%) | 0(0.0%) | 7(10.1%) | 0(0.0%) | 0(0.0%) |
| Red blood cell | 8(12.1%) | 0(0.0%) | 0(0.0%) | 5(7.7%) | 0(0.0%) | 0(0.0%) | 2(3.0%) | 0(0.0%) | 0(0.0%) |
| Electrocardiogram | 11(24.4%) | 1(2.2%) | 0(0.0%) | 4(8.9%) | 1(2.2%) | 1(2.6%) | 7(15.9%) | 1(2.3%) | 0(0.0%) |

## 10.2 Sub-group safety analysis

Considering that the baseline characteristics (including demographics at enrollment) can possibly affect the safety results, sub-group analysis was conducted on the safety results of the three groups using the subjects' baseline characteristics as the sub-group. See Table 10.2-1 for the results. The results of the sub-group analysis showed that, the overall incidence rate of adverse events was spread evenly among the sub-group of the three groups.

1) Gender: Adverse events occurred more in female subjects than in male subjects in all the three groups; the number of female subjects was double the number of male subjects in the placebo group and 600mg group, and the number of female subjects was slightly higher than the number of male subjects in the 900mg group.

2) Age: The number of patients aged above 65 years was 3-4 times the number of patients aged 65 years or below in all three groups.

3) Education level: This was evenly distributed in the three groups, and there were more patients with an education level above primary school than patients with primary school education level.

4) BMI: The number of patients with BMI of (18.5-24) was higher than the number of patients with the other two BMI levels in the three groups, and there were more patients with BMI of (≥24) than patients with BMI of (<18.5) in the three groups.

5) Family history: This was evenly distributed in the three groups, and there were significantly more patients without family history than patients with family history.

6) Drug therapy history: This was evenly distributed in the three groups, and there were significantly more patients without drug therapy history than patients with drug therapy history.

7) MMSE: The number of patients with MMSE of (≥20 points) was the highest in the three groups, while the number of patients with MMSE

of (15-20 points) was similar to the number of patients with MMSE of (<15 points) in the 600mg group and 900mg group; in the placebo group, the number of patients with MMSE of (15-20 points) was close to the number of patients with MMSE of (≥20 points) and higher than the number of patients with MMSE of (<15 points).

###### Table 10.2-1 Summary of the overall sub-group analysis of adverse events (SS)

| **Indicator** | **Category** | **Placebo**  **(N=85)** | **Sodium Oligomannate capsule 600mg**  **(N=84)** | **Sodium Oligomannate capsule**  **900mg**  **(N=86)** |
| --- | --- | --- | --- | --- |
| Gender | Male | 12(14.12%) | 11(13.10%) | 11(12.79%) |
|  | Female | 23(27.06%) | 20(23.81%) | 13(15.12%) |
| Age | >65 years old | 28(32.94%) | 23(27.38%) | 18(20.93%) |
|  | ≤65 years old | 7(8.24%) | 8(9.52%) | 6(6.98%) |
| Education level | Primary school | 16(18.82%) | 6(7.14%) | 8(9.30%) |
|  | Above primary school | 19(22.36%) | 25(29.77%) | 16(18.61%) |
| BMI | ≥24 | 8(9.41%) | 13(15.48%) | 6(6.98%) |
|  | 18.5-24 | 23(27.06%) | 16(19.05%) | 13(15.12%) |
|  | ＜18.5 | 4(4.71%) | 2(2.38%) | 5(5.81%) |
| Family history | Yes | 3(3.53%) | 2(2.38%) | 2(2.33%) |
|  | No | 32(37.65%) | 29(34.52%) | 22(25.58%) |
| Drug therapy history | Yes | 4(4.71%) | 2(2.38%) | 4(4.65%) |
|  | No | 31(36.47%) | 29(34.52%) | 20(23.26%) |
| MMSE | ≥20 points | 14(16.47%) | 17(20.24%) | 12(13.95%) |
|  | 15-20 points | 13(15.29%) | 5(5.95%) | 6(6.98%) |
|  | <15 points | 8(9.41%) | 9(10.71%) | 6(6.98%) |

###### Table 10.2-2 Baseline characteristics of the subjects in the three groups (SS)

| **Item** | **Placebo** | **Sodium Oligomannate capsule 600mg** | **Sodium Oligomannate capsule 900mg** |
| --- | --- | --- | --- |
| Gender |  |  |  |
| Male | 32(37.65%) | 39(46.43%) | 35(40.70%) |
| Female | 53(62.35%) | 45(53.57%) | 51(59.30%) |
| Total | 85 | 84 | 86 |
| Age |  |  |  |
| ＞65 | 59(69.41%) | 56(66.67%) | 58(69.05%) |
| ≤65 | 26(30.59%) | 28(33.33%) | 26(30.95%) |
| Total | 85 | 84 | 84 |
| Education level |  |  |  |
| Primary school | 31(36.47%) | 26(30.95%) | 27(32.14%) |
| Above primary school | 54(6353%) | 58(69.04%) | 57(67.85%) |
| Total | 85 | 84 | 84 |
| BMI |  |  |  |
| ≥24 | 18(21.18%) | 27(32.14%) | 27(32.14%) |
| 18.5-24 | 57(67.06%) | 51(60.71%) | 42(50.00%) |
| ＜18.5 | 10(10.76%) | 6(7.14%) | 15(17.86%) |
| Total | 85 | 84 | 84 |
| Family history |  |  |  |
| No | 79(92.94%) | 78(92.86%) | 74(88.10%) |
| Yes | 6(7.06%) | 6(7.14%) | 10(10.90%) |
| Total | 85 | 84 | 84 |
| Drug therapy history |  |  |  |
| No | 71(83.53%) | 77(91.67%) | 70(83.33%) |
| Yes | 14(16.47%) | 7(8.33%) | 14(16.67%) |
| Total | 85 | 84 | 84 |
| MMSE |  |  |  |
| ≥20 points | 31(36.47%) | 36(42.86%) | 44(52.38%) |
| 15-20 points | 28(32.94%) | 25(29.76%) | 21(25.00%) |
| <15 points | 26(30.59%) | 23(27.38%) | 19(22.62%) |
| Total | 85 | 84 | 84 |

## 10.3 Safety analysis of exploratory indicators

The results of the logistic regression analysis with gender, age, education level and MMSE score taken as prognostic factors showed that, the difference in the incidence rate of adverse events between the Sodium Oligomannate capsule 900mg group and placebo group was statistically significant, and the OR and the 95% CI of the incidence rate between the Sodium Oligomannate capsule 900mg group and placebo group was 2.036 (1.042, 3.978). This is a post-hoc analysis.

###### Table 10.3-1 Results of logistic regression analysis built using the incidence rate of all adverse events as the dependent variable (SS)

|  |  | | | | | | | | | | | | **OR 95% confidence interval** | | | |
| --- | --- | --- | --- | --- | --- | --- | --- | --- | --- | --- | --- | --- | --- | --- | --- | --- |
| **Indicator** | | **Type** |  | **Parameter estimation** |  | **Standard error** |  | **Wald-Chisq** |  | **P value** |  | **OR estimates** |  | **Lower limit** |  | **Upper limit** |
| Constant | |  |  | -0.4609 |  | 0.2626 |  | 3.0799 |  | 0.0793 |  | . |  | . |  | . |
| Group | | Sodium Oligomannate capsule 900mg vs placebo |  | -0.6446 |  | 0.3364 |  | 3.6718 |  | 0.0553 |  | 0.525 |  | 0.271 |  | 1.015 |
| Gender | | Male vs female |  | 0.1553 |  | 0.1832 |  | 0.7182 |  | 0.3967 |  | 1.364 |  | 0.665 |  | 2.797 |
| Age group | | >65 years old vs ≤65 years old |  | 0.3129 |  | 0.1915 |  | 2.6693 |  | 0.1023 |  | 1.870 |  | 0.883 |  | 3.961 |
| Education level | | Above primary school vs primary school |  | -0.2562 |  | 0.1854 |  | 1.9095 |  | 0.1670 |  | 0.599 |  | 0.290 |  | 1.239 |
| MMSE score | | <15 points vs ≥15 points |  | -0.1856 |  | 0.1935 |  | 0.9198 |  | 0.3375 |  | 0.690 |  | 0.323 |  | 1.473 |

###### Table 10.3-2 Results of logistic regression analysis built using the incidence rate of all adverse events as the dependent variable (SS)

| **Indicator** | **Type** |  | **Parameter**  **estimation** |  | **Standard**  **error** |  | **Wald-**  **Chisq** |  | **P value** |  | **OR**  **estimates** | **OR 95% confidence interval lower limit upper limit** | | | |
| --- | --- | --- | --- | --- | --- | --- | --- | --- | --- | --- | --- | --- | --- | --- | --- |
| Constant |  |  | -0.4900 |  | 0.2382 |  | 4.2314 |  | 0.0397 |  | . |  | . |  | . |
| Group | Sodium Oligomannate capsule 900mg vs placebo |  | -0.6275 |  | 0.3323 |  | 3.5645 |  | 0.0590 |  | 0.534 |  | 0.278 |  | 1.024 |
| Age group | >65 years old vs ≤65 years old |  | 0.3129 |  | 0.1915 |  | 2.6693 |  | 0.1023 |  | 1.870 |  | 0.883 |  | 3.961 |

**11. Discussion and conclusion**

**Discussion**

Sodium Oligomannate is an oligosaccharide obtained from the degradation and fractionation of sodium alginate, which is the raw material. It is a low-molecular-weight acidic oligosaccharide compound with a specific molecular skeleton that can possibly bind at high affinity with Aβ, and it is produced based on the structure-activity relationship pattern of the interaction between Aβ molecules and carbohydrates, and via rational drug design, specific degradation technologies and Aβ biochip screening. The results of past pharmacodynamic, safety and pharmacokinetic tests showed that, Sodium Oligomannate's high specificity for Aβ, low toxicity and easy penetration of the blood-brain barrier have laid a good research foundation for the development of ideal therapeutic drugs for AD. Summarizing the results of past studies, the drug is recommended for application on mild to moderate Alzheimer's disease in clinical trials. In this study, the Alzheimer's Disease Assessment Scale - cognitive component (ADAS-cog/12) was used to evaluate the improvement in cognitive functions as the primary efficacy endpoint. The objective was to investigate the optimal therapeutic dose and perform an initial evaluation of the efficacy and safety of Sodium Oligomannate capsule (971 capsule) in the treatment of mild to moderate Alzheimer's disease, so as to provide a basis for the phase III clinical study.

This study enrolled 255 patients with mild to moderate Alzheimer's disease in 24 centers in total. The number of subjects who entered FAS in the placebo group, 600mg group and 900mg group was 83, 76 and 83, respectively; the number of subjects who entered PPS was 75, 67 and 71, respectively; and the number of subjects who entered SS was 85, 84 and 86, respectively. At baseline, the demographic characteristics, vital signs, physical examination, Alzheimer's disease history, past history or comorbid diseases, history of drug allergy and abuse, history of alcohol abuse and other such clinical data of the three groups of subjects were not statistically significant. The clinical features of all indicators of laboratory tests and ECG examination were essentially balanced, and there was good comparability. The subjects in all three groups had good drug compliance during the study.

The safety analysis of the 600mg group and 900mg group in this study showed that, the overall incidence rate of adverse events in the placebo group, 600mg group and 900mg group was 41.18%, 36.90% and 27.91%, respectively. The incidence rate of adverse events related to the study drug was 3.53%, 5.59% and 3.49%, respectively. The incidence rate of adverse events leading to dropouts was 1.18%, 3.57% and 3.49%, respectively. The drug-related adverse events were mainly presented as mild to moderate psychiatric symptoms and skin symptoms.

There were 14 cases (15 incidents) of serious adverse events (SAEs) in the entire study. In the investigator's judgment, ten of the SAEs were definitely unrelated to the drug, four SAEs were probably unrelated to the drug, and one SAE (600mg group, 01028) was probably related to the drug. The safety results showed that the use of Sodium Oligomannate capsule 600mg and 900mg for the treatment of patients with mild to moderate Alzheimer's disease was safe with good tolerability, which is consistent with the results of preclinical study.

**Conclusion**

The use of Sodium Oligomannate capsule 600mg and 900mg for the treatment of patients with mild to moderate Alzheimer's disease for 24 weeks is safe with good tolerability.
